# Individualized metabolic profiling stratifies pancreatic and biliary tract cancer: a useful tool for innovative screening programs and predictive strategies in healthcare

**DOI:** 10.1007/s13167-018-0147-5

**Published:** 2018-08-17

**Authors:** Jun Hwa Lee, Seung Eun Yu, Kyung-Hee Kim, Myung Hyun Yu, In-Hye Jeong, Jae Youl Cho, Sang-Jae Park, Woo Jin Lee, Sung-Sik Han, Tae Hyun Kim, Eun Kyung Hong, Sang Myung Woo, Byong Chul Yoo

**Affiliations:** 1 Biomarker Branch, Research Institute, National Cancer Center, Goyang, 10408 Republic of Korea; 2 Omics Core Laboratory, Research Institute, National Cancer Center, Goyang, 10408 Republic of Korea; 3 Department of Genetic Engineering, Sungkyunkwan University, Suwon, 16419 Republic of Korea; 4 Center for Liver Cancer, Hospital, National Cancer Center, Goyang, 10408 Republic of Korea; 5 Department of Cancer Biomedical Science, Graduate School of Cancer Science and Policy, National Cancer Center, Goyang, 10408 Republic of Korea

**Keywords:** Predictive preventive personalized medicine, Pancreatic cancer, Biliary tract cancer, Biomarkers, Metabolomics, Bioinformatics

## Abstract

**Background:**

Pancreatic cancer (PC) and biliary tract cancer (BTC) are highly aggressive cancers, characterized by their rarity, difficulty in diagnosis, and overall poor prognosis. Diagnosis of PC and BTC is complex and is made using a combination of appropriate clinical suspicion, imaging and endoscopic techniques, and cytopathological examination. However, the late-stage detection and poor prognosis of this tumor have led to an urgent need for biomarkers for early and/or predictive diagnosis and improved personalized treatments.

**Working hypothesis:**

There are two hypotheses for focusing on low-mass metabolites in the blood. First, valuable information can be obtained from the masses and relative amounts of such metabolites, which present as low-mass ions (LMIs) in mass spectra. Second, metabolic profiling of individuals may provide important information regarding biological changes in disease states that is useful for the early diagnosis of PC and BTC.

**Materials and methods:**

To assess whether profiling metabolites in serum can serve as a non-invasive screening tool for PC and BTC, 320 serum samples were obtained from patients with PC (*n* = 51), BTC (*n* = 39), colorectal cancer (CRC) (*n* = 100), and ovarian cancer (OVC) (*n* = 30), and from healthy control subjects (control) (n = 100). We obtained information on the relative amounts of metabolites, as LMIs, via triple time-of-flight mass spectrometry. All data were analyzed according to the peak area ratios of discriminative LMIs.

**Results and conclusions:**

The levels of the 14 discriminative LMIs were higher in the PC and BTC groups than in the control, CRC and OVC groups, but only two LMIs discriminated between PC and BTC: lysophosphatidylcholine (LysoPC) (16:0) and LysoPC(20:4). The levels of these two LysoPCs were also slightly lower in the PC/BTC/CRC/OVC groups compared with the control group. Taken together, the data showed that metabolic profiling can precisely denote the status of cancer, and, thus, could be useful for screening. This study not only details efficient methods to identify discriminative LMIs for cancer screening but also provides an example of metabolic profiling for distinguishing PC from BTC. Furthermore, the two metabolites [LysoPC(16:0), LysoPC(20:4)] shown to discriminate these diseases are potentially useful when combined with other, previously identified protein or metabolic biomarkers for predictive, preventive and personalized medical approach.

**Electronic supplementary material:**

The online version of this article (10.1007/s13167-018-0147-5) contains supplementary material, which is available to authorized users.

## Introduction

Pancreatic cancer (PC) and biliary tract cancer (BTC) are highly aggressive cancers, for which the mortality rates closely parallel the incidence rates [1]. Most PC and BTC cases are not accompanied by clinical symptoms until the disease reaches an advanced stage [2, 3]. A minority of PC and BTC patients present with surgically resectable disease, but the relapse rate is high [4, 5]. PC is the fifth-most deadly cancer, and only approximately 8% of patients with PC survive for 5 years; thus, it has the worst survival rate among all 22 common cancers [6, 7]. Meanwhile, the overall 5-year survival time of advanced BTC is less than 1 year [8]. BTCs are generally divided into intrahepatic cholangiocarcinomas, perihilar or extrahepatic cholangiocarcinomas, and gallbladder tumors [9]. The incidence of cholangiocarcinoma remains significantly higher (by up to 40-fold) in China and Korea than in Western countries, and, thus, poses a significant public health problem [10]. Therefore, diagnosing and classifying PC and BTC at the early stage is urgently needed to increase the likelihood of cure.

Concerning early detection of PC or BTC, imaging studies and biomarkers have both been used in the clinic. Imaging modalities, such as endoscopic ultrasound, computed tomography, and magnetic resonance imaging have difficulty in differentiating non-malignant and malignant tissue [11, 12]. In addition, the high cost of these procedures limits their use in the follow-up of asymptomatic cases [13]. The most well-validated and useful biomarker for PC and BTC is carbohydrate antigen (CA19-9); however, CA19-9 is not recommended for general screening because it is upregulated in other inflammatory conditions such as chronic pancreatitis and cholangitis [14, 15]. Other molecular markers of PC and BTC have also been used, such as circulating tumor cells, epigenetic markers, and microRNAs. Liquid biopsy with circulating tumor DNA (ctDNA) is an emerging technology to detect actionable alterations [16]. A recent study found that low-level PIK3CA mutations can be detected in serum using ctDNA, indicating the usefulness of ctDNA to detect cancer-derived mutations in metastatic BTC [17]. *KRAS* mutations have been detected by digital polymerase chain reaction in ctDNA [2], and it has been suggested that serum miRNA is more useful than CA19-9 for diagnosis of PC and BTC [18]. Recently, methylation-on-beads technology, which can detect methylation changes in DNA circulating in serum, showed potential for PC diagnosis [19–21]. Several novel biomarkers for early diagnosis of PC and BTC have been suggested over the last few years; however, no molecular biomarkers are currently suitable for clinical use.

Recently, several metabolites have been reported as potential biomarkers for various cancers [20]. Some new metabolites have been validated for diagnostic purposes; choline was consistently elevated in breast cancer biopsy samples, for example, compared to levels in normal tissue, and the diagnostic accuracy using this marker was 100% [22]. Increased citrate and decreased spermine levels in prostatic fluid showed potential utility in screening for prostate cancer [23, 24]. Furthermore, a metabolomics-based urine screening test to detect adenomatous polyps has been reported [25]. However, only a few metabolic studies have screened for PC and BTC. In the present metabolic profiling study, we enrolled not only patients with PC and BTC but also those with other types of cancer such as colorectal cancer (CRC), and ovarian cancer (OVC) as a positive control. This was intended to rule out common metabolic factors in cancers such as fibrinogen peptide alpha chain and to select more reliable metabolic candidates relevant to PC or BTC.

**Scheme 1:**
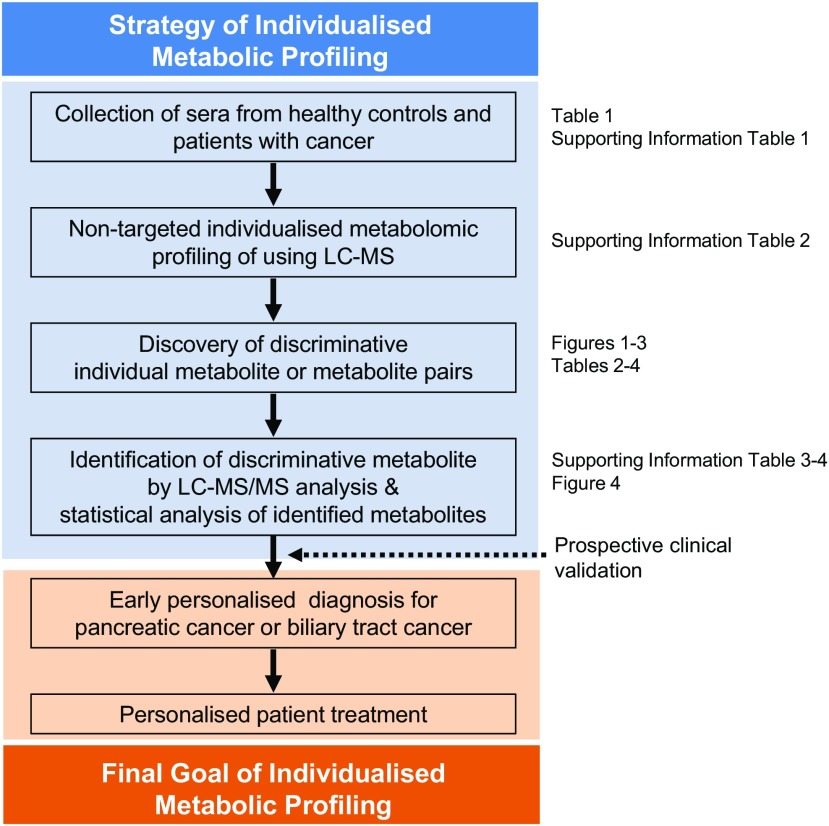
Strategy for individualized non-target metabolic profiling of PC and BTC patients based on serum analysis as well as our goal of predictive preventive personalized medicine (PPPM). Non-target metabolic profiling was performed using liquid chromatography-mass spectrometry (LC-MS). The retrospective findings at each step are shown at left; these should be validated in a prospective clinical study

Scheme 1 summarizes our research strategy for the metabolic profiling of PC and BTC patients based on serum analysis for predictive diagnosis, targeted prevention, and personalized treatment. Here, we report the metabolic profiles and discuss their clinical utility.

## Materials and methods

### Study population

In total, 320 serum samples were collected from healthy individuals (control) and patients with PC, BTC, CRC, and OVC (Table 1 and Supporting Information Table 1). Eligible control subjects were selected from individuals in the cancer screening cohort, who were subjected to routine health examinations at the Center for Cancer Prevention and Detection of the National Cancer Center, Korea, between 2001 and 2017. In total, 320 healthy subjects were included. Control patients were matched for age, sex, and date of admission or visit. The PC and BTC groups included high-risk individuals (HRIs). The majority of HRIs for pancreatic cancer (*n* = 20, 95%) were patients who presented with intraductal papillary mucinous neoplasms of the pancreas, but one had chronic pancreatitis. The study included 25 BTC HRIs, of whom 20 (80%) were infected with hepatitis B virus (HBV) and 1 (4%) with hepatitis C virus (HCV). The others had liver cirrhosis (*n* = 1, 4%), gallstone (*n* = 1, 4%), and biliary tract stones (*n* = 2, 8%). Samples were gathered before surgery or chemotherapy to prevent any effects of anesthetic or anticancer agents on low-mass ions (LMIs) in sera. Informed consent was obtained from all participants, and the institutional review board of each participating institution approved the research protocol (IRB numbers: NCC2015-0173, NCC2015-0248, and NCC2018-0005). This research was part-funded by the Korea Gynecologic Cancer Bank through the Bio & Medical Technology Development Program of the MSIP, Korea.

**Table 1:** Information of the study participants

	Number				Stage					Age (years)		
	Total	M	F	HRG	0	I	II	III	IV	Mean	SD	Range
PC	51	30	21	11		1	10	3	26	63.7	10.6	33–82
BTC	39	25	14	25			1	2	11	62.3	11.3	36–84
Control	100	70	30							56.8	7.5	37–70
CRC	100	58	42		1	26	22	44	7	61.1	11.2	33–82
OVC	30		30			11	2	12	4	53.7	10.6	37–78

Supplementary information on individual participants is tabulated in Supporting Information Table 1, which also shows the sample collection sites. For the OVC group, only 29 samples with available data were considered. M male, F female, HRG high risk group, SD standard deviation, PC pancreatic cancer, BTC biliary tract cancer, CRC colorectal cancer, OVC ovarian cancer

### Serum extraction for metabolite profiling

Fifty microliters of serum were added to 1 mL water. After vortexing, 2 mL MeOH and 0.9 mL dichloromethane were added. After vortexing and placing on ice for 30 min, 1 mL water and 0.9 mL dichloromethane were added, and the mixture was centrifuged (1500 rpm, 10 min, room temperature). The supernatant was dried under an N_2_ stream and subjected to MS analysis.

### Serum metabolite analysis

Dried samples were reconstituted in 0.1% (*v*/*v*) formic acid and subjected to liquid chromatography-tandem mass spectrometry (LC-MS/MS) using a Nexera X2 system (Shimadzu, Tokyo, Japan) coupled to a triple time-of-flight (TOF) 5600+ system (Sciex, Tokyo, Japan) equipped at the front end with a DuoSpray ion source (Sciex). For ultra-high-performance LC, the samples were loaded onto Atlantis T3 sentry guard cartridges (3 μm; 2.1 × 10 mm; Waters, Milford, MA, USA), and separation proceeded via an Atlantis T3 column (3 μm, 2.1 × 100 mm; Waters). The MS system was set to perform one full scan (50 to 1200 *m*/*z*, mass-to-charge ratio) followed by MS/MS of the ten most-abundant parent ions (mass tolerance, 50 mDa; collision energy, 35%).

### Individual LMIs that enabled discrimination of the PC and BTC groups and the control, CRC, and OVC groups

Lists of LC-MS peaks (.peaks files) were created from a corresponding file (.wiff) for every sample using MarkerView software (Sciex). The parameters for this process were as follows: minimum retention time (RT), 0.00 min; subtraction offset, 10 scans; subtraction multiplication factor, 1.3; noise threshold, 10; minimum spectral peak width, 10 ppm; and minimum RT peak width, 5 scans. Next, a table of peaks was created by importing the .peaks files into the MarkerView software, for all samples simultaneously, using the following parameters: RT tolerance, 0.01 min; mass tolerance, 10.0 ppm; intensity threshold, 10; maximum number of peaks, 20,000; and area reporting using the “area integrated from the raw data, not from original peak finding.” The table of peaks contains data on the mass value (*m*/*z*), RT (min), and peak area.

The data in the peak table were converted to logarithms. A peak area of 0 was set to 1 because log_10_(0) is not defined and log_10_(1) is zero again. LMIs having outstanding discriminative ability (i.e., distinguishing PC and BTC from control, CRC and OVC groups) were identified using the logarithmic peak table. The methods for assessing single ions were as follows: 1) For each LMI, a discrimination threshold was determined, with an increment of 0.01, whereby the sum of the sensitivity and specificity was highest. When more than one threshold showed the same discrimination performance, the thresholds were averaged. Furthermore, in cases of perfect discrimination, discrimination ability was given by the difference between the maximum and minimum thresholds. 2) A few discriminative LMIs were determined comparing the discrimination performance of all *N* LMIs.

### LMI pairs that enabled discrimination of the PC and BTC groups, and between the PC and BTC groups and PC and BTC high-risk groups

To discriminate the PC and BTC groups, dual-ion methods based on the ratio of logarithmic peak areas, or the difference between peaks, were devised. Mathematically, the latter is the logarithm of the ratio of peak areas, whereas the former is the ratio of the logarithm of peak areas. The dual-ion methods were executed as follows: 1) _*N*_C_2_ combinations were arranged as a list. Each pair of LMIs on the list was examined twice. One of the two LMIs in each pair was arbitrarily chosen as the numerator (or minuend) LMI initially, and was then used as the denominator (or subtrahend) LMI. 2) All LMI pairs in the list were investigated in sequence. The ratio of the common logarithm of the two LMIs, or their difference, was calculated as a discriminant score for all samples. 3) Thresholds were determined with an increment of 0.01 such that the sum of the sensitivity and specificity was highest. When more than one threshold showed the same discrimination performance, the thresholds were averaged. 4) The two ordered pairs, thresholds, and their summed sensitivity and specificity values were tabulated for later comparison. 5) Several discriminative pairs were identified by comparing the discrimination performance of all ordered pairs.

### Identification of metabolite ions

The MS and MS/MS spectra were analyzed by the Formula Finder tool (SCIEX) to determine probable elemental compositions within a specified mass tolerance of a given mass value (*m*/*z*), using PeakView software (SCIEX). By interrogating the Human Metabolome Database (HMDB), the compounds producing the observed *m*/*z* ions were identified and listed in rank order based on the MS and MS/MS data.

### Statistical analyses

Differences in means between any two groups in Supporting Information Table 4 were evaluated by an independent sample *t* test for normally distributed data. When data were not normally distributed, the Mann–Whitney *U* test was used to compare means. Normality was verified by Shapiro–Wilk tests. Statistical analyses were performed using R version 3.5.0 and a *p*-value of less than 0.05 was considered indicative of statistical significance.

## Results

### Individual LMIs that enabled discrimination of the PC and BTC groups and the control, CRC and OVC groups

**Fig. 1:**
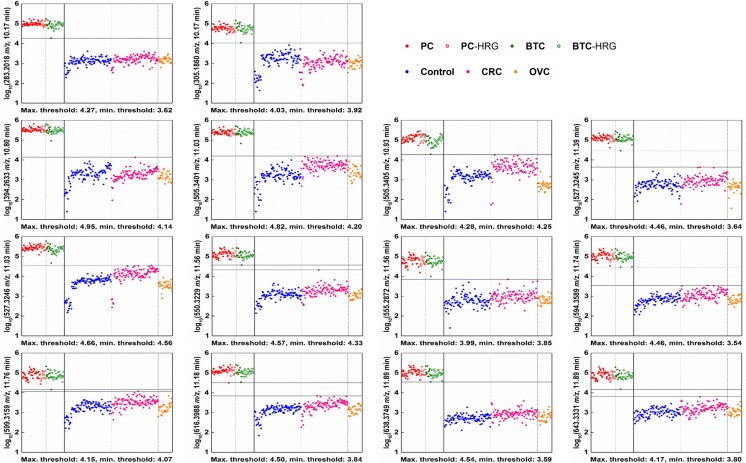
Individual LMIs discriminating the PC and BTC groups from the control, CRC and OVC groups. The mass peak areas of the selected LMIs were converted to logarithms. The 14 discriminative LMIs independently showed perfect discrimination. Horizontal lines denote the maximum and minimum thresholds. The results should be validated using a large number of new samples and including more LMIs with the next highest performance. LMI, low-mass ion; PC, pancreatic cancer; BTC, biliary tract cancer; CRC, colorectal cancer; OVC, ovarian cancer; HRG, high-risk group

The peak table consisted of 6724 LMIs (Supporting Information Table 2). The single-ion method identified 14 discriminative LMIs (Fig. 1), each showing perfect discrimination between the PC and BTC groups and the control, CRC and OVC groups. Table 2 shows the discrimination performance of the LMIs in terms of the difference between the maximum and minimum thresholds, and Fisher’s discriminant ratio values. The logarithmic peaks of all 14 LMIs were higher in the PC and BTC groups than in the control, CRC and OVC groups. No LMIs that showed perfect discrimination were higher in the control, CRC and OVC groups than in the PC and BTC groups.

**Table 2:** Discrimination performance of individual discriminative LMIs (PC/BTC vs. control/CRC/OVC groups)

Mass ion		Separating threshold			Fisher’s discriminant ratio
Mass value (*m*/*z*)	Retention time (min)	Maximum	Minimum	Difference	
283.2018	10.17	4.27	3.62	0.65	53.50
305.1860	10.17	4.03	3.92	0.11	16.95
394.2633	10.80	4.95	4.14	0.81	39.89
505.3401	11.03	4.82	4.20	0.62	18.78
505.3405	10.93	4.28	4.25	0.03	8.40
527.3245	11.39	4.46	3.64	0.82	48.98
527.3246	11.03	4.66	4.56	0.10	10.38
550.3229	11.56	4.57	4.33	0.24	26.70
555.2872	11.56	3.99	3.85	0.14	26.65
594.3589	11.74	4.46	3.54	0.92	41.05
599.3159	11.76	4.15	4.07	0.08	17.40
616.3988	11.18	4.50	3.84	0.66	31.55
638.3749	11.89	4.54	3.59	0.95	56.24
643.3331	11.89	4.17	3.80	0.37	35.64

LMI low-mass ion, PC pancreatic cancer, BTC biliary tract cancer, CRC colorectal cancer, OVC ovarian cancer

### LMI pairs that enabled discrimination of the PC and BTC groups

No single LMI showed perfect discrimination between the PC and BTC groups. The highest performance attained by a single LMI was a sensitivity of 80.39% (10 false-negatives) and a specificity of 94.87% (2 false-positives), achieved by a mass ion of 271.1904 *m*/*z* at an RT of 16.37 min (Supporting Information Fig. 1a). Its logarithmic peaks were higher in the BTC group than in the PC group.

**Fig. 2:**
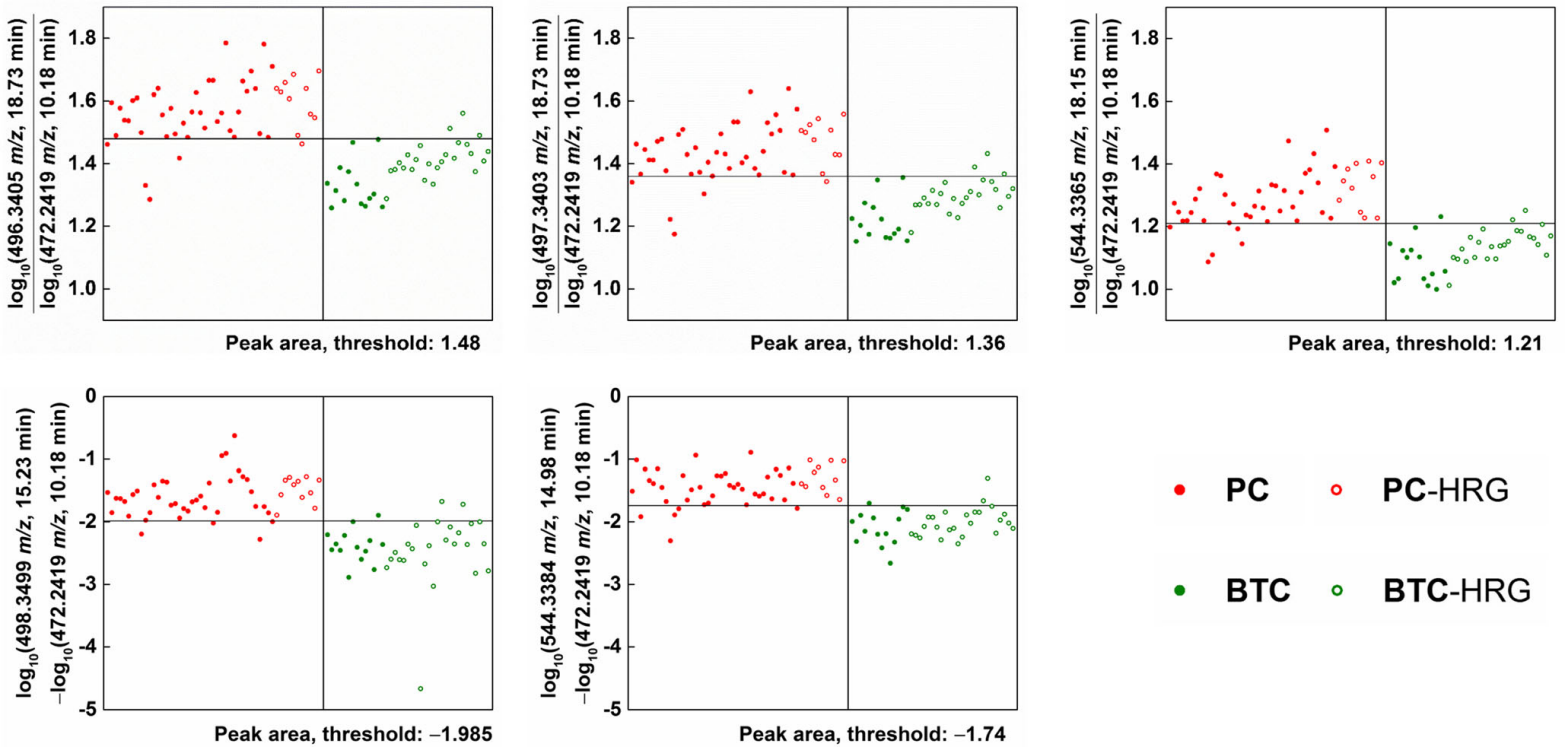
LMI pairs discriminating the PC from BTC groups. The mass peak areas of the selected LMIs were converted to logarithms, and the relative ratio or difference of each LMI pair showed great performance (combined sensitivity / specificity > 90%) in discriminating between PC and BTC. The horizontal line denotes the corresponding discrimination threshold. Overall, the logarithmic peak areas of the numerator or minuend LMIs were higher in the PC group than in the BTC group and the reverse was true for denominator or subtrahend LMIs. LMI, low-mass ion; PC, pancreatic cancer; BTC, biliary tract cancer; HRG, high-risk group

The dual-ion methods revealed five discriminative LMI pairs (Fig. 2), each showing a combined sensitivity/specificity > 90%. Table 3 shows their discrimination performance. The first three pairs achieved their high performance based on the ratio of the logarithm of peak areas, while the remaining two pairs achieved their performance based on the logarithm of the ratio of peak areas. A mass ion of 472.2419 *m/z* at an RT of 10.18 min was used as the denominator or subtrahend LMI. Overall, the logarithmic peak areas of numerator or minuend LMIs were higher in PC than in BTC and the reverse was true for denominator or subtrahend LMIs.

**Table 3:** Discrimination performance of discriminative LMI pairs (PC versus BTC)

Mass value (*m*/*z*)	Retention time (min)	Mass value (*m*/*z*)	Retention time (min)	Sensitivity	Specificity
Numerator LMI		Denominator LMI			
496.3405	18.73	472.2419	10.18	90.20%	92.31%
497.3403	18.73			90.20%	92.31%
544.3365	18.15			90.20%	92.31%
Minuend LMI		Subtrahend LMI			
498.3499	15.23	472.2419	10.18	92.16%	92.31%
544.3384	14.98			90.20%	92.31%

LMI low-mass ion, PC pancreatic cancer, BTC biliary tract cancer

### LMI pairs that enabled discrimination of the PC and BTC groups and the PC and BTC high-risk groups

As with the PC vs. BTC groups, no single LMI showed perfect discrimination between the PC and BTC groups and the PC and BTC high-risk groups. The highest performance was a sensitivity of 68.52% (17 false-negatives) and a specificity of 97.22% (1 false-positive), achieved by a mass ion of 1030.6515 *m*/*z* at an RT of 11.15 min (Supporting Information Fig. 1b). Its logarithmic peaks were higher in the PC and BTC groups than in the PC and BTC high-risk groups.

**Fig. 3:**
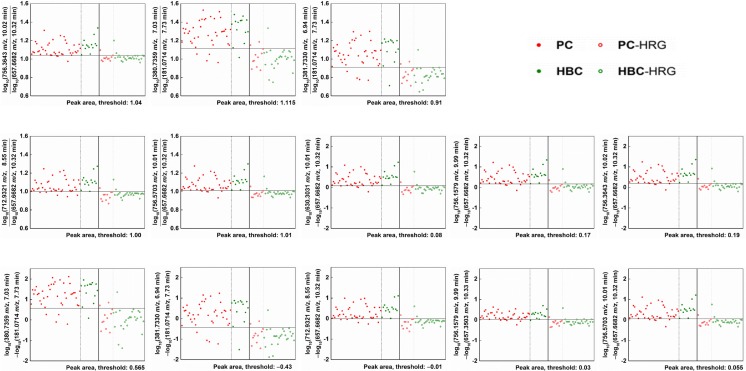
LMI pairs discriminating the PC and BTC groups from PC and BTC high-risk groups. The mass peak areas of the selected LMIs were converted to logarithms, and the relative ratio or difference of each LMI pair showed a high level of performance (combined sensitivity/specificity > 90%) in discriminating between the PC and BTC groups and the PC and BTC high-risk groups. The horizontal line denotes the corresponding discrimination threshold. Overall, the logarithmic peak areas of the numerator or minuend LMIs were higher in the cancer group than in the high-risk group and the reverse was true for denominator or subtrahend LMIs. The same five LMI pairs were selected using the relative ratio or difference. LMI; low-mass ion, PC, pancreatic cancer; BTC, biliary tract cancer; HRG, high-risk group

The dual-ion methods revealed eight discriminative LMI pairs (Fig. 3), each showing a combined sensitivity/specificity of > 90%. Table 4 shows their discrimination performance. The first five pairs achieved their performance based on the ratio of the logarithm of peak areas, and the remaining eight pairs achieved their performance based on the logarithm of the ratio of peak areas. The latter contained the former.

**Table 4:** Discrimination performance of discriminative LMI pairs (PC/BTC versus PC/BTC HRG)

Mass value (*m*/*z*)	Retention time (min)	Mass value (*m*/*z*)	Retention time (min)	Sensitivity	Specificity
Numerator LMI		Denominator LMI			
756.3643	10.02	657.6682	10.32	94.44%	91.67%
380.7359	7.03	181.0714	7.73	90.74%	91.67%
381.7330	6.94	181.0714	7.73	90.74%	91.67%
712.9321	8.55	657.6682	10.32	90.74%	91.67%
756.5703	10.01	657.6682	10.32	90.74%	91.67%
Minuend LMI		Subtrahend LMI			
630.3031	10.01	657.6682	10.32	92.59%	91.67%
756.1579	9.99	657.6682	10.32	92.59%	91.67%
756.3643	10.02	657.6682	10.32	92.59%	91.67%
380.7359	7.03	181.0714	7.73	90.74%	91.67%
381.7330	6.94	181.0714	7.73	90.74%	91.67%
712.9321	8.55	657.6682	10.32	90.74%	91.67%
756.1579	9.99	657.3503	10.33	90.74%	91.67%
756.5703	10.01	657.6682	10.32	90.74%	91.67%

LMI low-mass ion, PC pancreatic cancer, BTC biliary tract cancer, HRG high risk group

### Candidate metabolites for individual LMIs and LMI pairs

**Fig. 4:**
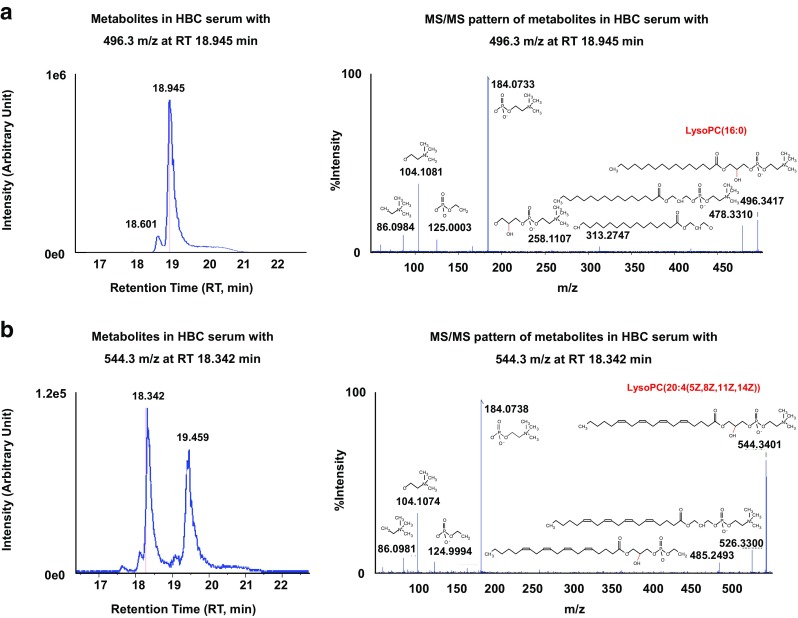
LMIs discriminating between the PC and BTC groups. Two LMIs (496.3 *m*/*z*, retention time (RT) of 18.9 min **a** and 544.3 *m*/*z*, RT of 18.3 min **b** were identified as LysoPC(16,0) and LysoPC(20:4(5Z,8Z,11Z,14Z)) according to the tandem mass spectrometry (MS/MS) patterns of serum samples of BTC patients

**Fig. 5:**
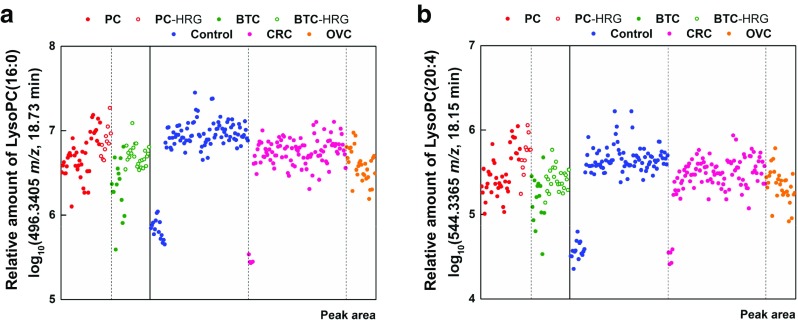
Amounts of LysoPC(16:0) and LysoPC(20:4(5Z,8Z,11Z,14Z)) in the serum of PC and BTC patients. The amounts of the two metabolites (unit:logarithm of the mass peak area) were slightly lower in the PC, BTC, CRC and OVC groups than in the control group, and no single metabolite could discriminate between the cancer groups and the control group

Supporting Information Table 3 shows candidate metabolites for LMIs. Nine LMIs were not matched with any metabolites in the HMDB and, in many cases, the mass information of single LMIs did not reduce the number of candidate metabolites. However, two LMIs (496.3 *m/z* at an RT of 18.9 min and 544.3 *m*/*z* at an RT of 18.3 min), lysophosphatidylcholine (LysoPC) (16:0) and LysoPC(20:4(5Z,8Z,11Z,14Z)), identified according to their MS/MS patterns in the analyses of the sera of BTC patients, could discriminate between PC and BTC (Figs. 2 and 4, Table 3). The relative amounts of LysoPC(16:0) and LysoPC(20:4(5Z,8Z,11Z,14Z)) were significantly lower in the PC, BTC, CRC, and OVC groups than in the control group (Fig. 5, Supporting Information Table 4). LMI alone did not allow discrimination of the cancer and control groups (Fig. 5). However, statistical analyses confirmed the differential levels of the two metabolites in the PC/BTC vs. control/CRC/OVC and PC vs. BTC groups (Supporting Information Table 4).

## Discussion

The rapid development of mass analysis for metabolomics facilitates discovery of reliable cancer-screening biomarkers. Such information will enable predictive, preventive, and personalized medicine (PPPM) for cancer patients [26–28]. Recently, we analyzed metabolites detected as LMIs by mass spectrometry (MS) and reported the metabolic profiles of cancer patients based on serum analyses. These metabolic profiles were useful for predicting, diagnosing, and predicting the prognosis, of cancer [29–31]. In the present study, we obtained metabolic profiles based on MS analysis of serum from PC and BTC patients, and found that the profiles showed potential for diagnosing cancer.

Fourteen discriminative LMIs were identified, and each showed perfect discrimination between the PC and BTC groups and the control, CRC, and OVC groups (Fig. 1 and Table 2). The logarithmic peaks of all 14 LMIs were higher in the PC and BTC groups (Fig. 1). When the data in the peak table were normalized according to the “total area sums” method, two additional individual LMIs (527.3218 *m*/*z*, 10.95 min RT and 749.4577 *m*/*z*, 11.33 min RT, (Supporting Information Fig. 2A, C) showed perfect discrimination between the PC and BTC groups and the control, CRC and OVC groups. The normalization yielded the same total peak areas for each sample in the peak table, via multiplication by a scaling factor. However, they yielded the next highest (15th and 16th) discrimination performance with the unnormalized peak table (Supporting Information Fig. 2B, D). Because the normalization process made a minor difference, it was not considered in this article.

No single LMI discriminated between the PC and BTC groups, but five LMI pairs were discriminative (Fig. 2 and Table 3). These LMI pairs showed a combined sensitivity/specificity > 90%. The mass ion of 472.2419 *m*/*z* at an RT of 10.18 min was used as the denominator or subtrahend LMI, and may be an important metabolite for discriminating between PC and BTC. The HMDB suggested two metabolites, hydroxydesmthyl doxepin glucuronide and (E)-2-hydroxydoxepin glucuronide, as candidates for LMI with 472.2419 *m*/*z* (Supporting Information Table 2). Both are metabolites of the psychotropic agent doxepin. Other endogenous metabolites should be sought that respond to the LMI of 472.2419 *m*/*z*. Similar to the situation for discriminating between the PC and BTC groups, no single LMI discriminated between the PC and BTC and PC and BTC high risk groups. However, eight LMI pairs were discriminative (Fig. 3 and Table 4) and showed high sensitivity and specificity.

Metabolic profiling provided useful information regarding screening for PC and BTC, and also showed a shortage in the identification of LMIs selected. Two LMIs (496.3 *m*/*z* at an RT of 18.9 min and 544.3 *m/z* at an RT of 18.3 min), identified as LysoPC(16:0) and LysoPC(20:4(5Z,8Z,11Z,14Z)), discriminated between PC and BTC, Figs. 2 and 4, Table 3). However, the relative amounts of LysoPC(16:0) and LysoPC(20:4(5Z,8Z,11Z,14Z)) were also slightly decreased in the PC/BTC/CRC/OVC groups compared with those in the control group (Fig. 5).

LysoPC is a product of the phosphatidylcholine hydrolysis precipitated by phospholipase A activity; in tumor tissue, LysoPC is usually generated by saturated PC after the accumulation of liposomes, and has been shown to activate cells from several lineages [32, 33]. Higher LysoPC levels were associated with lower risks of breast, prostate, and colorectal cancers [34], response to chemoradiotherapy in esophageal squamous cell carcinoma [35], and reduced melanoma cell adhesion and metastasis [32, 36]. However, decreased levels of LysoPC in blood or tissue precede the diagnosis of cancer by several years. Decreased LysoPC levels have been reported in the blood and tissue samples of patients with many types of cancer [30, 37, 38], e.g., blood samples of colorectal [39–41] and cervical cancer patients [40] and tissue samples of gastric [42], prostate [43], and liver [44] cancer patients. Considering these results across many types of cancer, metabolic changes in lipid metabolism may drive tumorigenesis.

Recent studies have shown that LysoPC can cause cholangiocyte senescence, which potentially contributes to the pathogenesis of BTC [45]. Furthermore, LysoPC can inhibit cholangiocyte apoptosis by inducing COX-2 expression via a Raf-1-dependent mechanism, and such anti-apoptotic effects might be important in biliary tract carcinogenesis in patients with compromised pancreaticobiliary ductal junctions [46]. Similar to other types of cancer, we found a reduced level of LysoPC in the serum of BTC patients [47], as well as significantly decreased levels of LysoPC in the bile of these patients compared with those with benign biliary tract disease [10]. These findings strongly imply that LysoPC may be useful for BTC diagnosis and prognosis [47, 48].

## Conclusions and expert recommendations

Targeted metabolomic profiling uses a mixture of standard metabolites and so allows identification and quantification of metabolites in samples exactly matched to standard metabolites. Targeted metabolomic profiling provides information on metabolites of interest, whereas non-targeted metabolomic profiling harvests information on the mass-to-charge ratio (*m*/*z*) and relative amounts of metabolites in a sample. Compared to targeted metabolomic profiling, non-targeted metabolomic profiling is limited in its ability to identify metabolites. In non-targeted metabolomic profiling, metabolites are present as LMIs in mass spectra, the *m*/*z* of each of which can match multiple metabolites. For identification of LMIs by non-targeted metabolomics, candidate metabolites must be listed in HMDB using only their *m*/*z*, and the MS/MS patterns of the LMIs must be compared with those of candidate metabolites obtained from commercial sources. Therefore, unlike targeted metabolomics, identification of metabolites is laborious as it depends on the *m*/*z*. Furthermore, if standard metabolites are not commercially available, they must be synthesized. Therefore, two types of metabolomic profiling can be performed, depending on the goal of our research. Our data indicate that non-targeted metabolic profiling based on blood samples can be done using information on the mass and relative amounts of LMIs. Metabolic profiling can precisely denote the status of diseases such as cancer and, thus, can be used for cancer screening. The present study not only described efficient methods for selecting discriminative (between PC and BTC) LMIs for the purposes of cancer screening but also provided an example of non-targeted metabolic profiling for screening these diseases. Information on all individual metabolites obtained in this retrospective, non-targeted metabolic profiling will facilitate validation of our results in a prospective study involving a large number of patients. No screening test has been shown to lower the risk of dying from PC and BTC. Two metabolites [LysoPC(16:0), LysoPC(20:4)] have potential utility for distinguishing PC from BTC when combined with other, previously identified proteins or metabolic biomarkers for predictive preventive personalized medicine to identify individuals at high risk for PC and BTC.

## Electronic supplementary material

ESM 1 (DOCX 388 kb)

ESM 2 (DOCX 554 kb)

## References

[1] Siegel RL, Miller KD, Jemal A (2016). Cancer statistics. CA Cancer J Clin.

[2] Kim MK, Woo SM, Park B, Yoon KA, Kim YH, Joo J, Lee WJ, Han SS, Park SJ, Kong SY (2018). Prognostic implications of multiplex detection of KRAS mutations in cell-FreeDNA from patients with pancreatic ductal adenocarcinoma. Clin Chem.

[3] Xu X, Cheng S, Ding C (2015). Identification of bile biomarkers of biliary tract cancer through a liquid chromatography/mass spectrometry-based metabolomic method. Mol Med Rep.

[4] Wang SJ, Lemieux A, Kalpathy-Cramer J, Ord CB, Walker GV, Fuller CD, Kim JS, Thomas CR (2011). Nomogram for predicting the benefit of adjuvant chemoradiotherapy for resected gallbladder cancer. J Clin Oncol.

[5] Wang Y, Li J, Xia Y, Gong R, Wang K, Yan Z, Wan X, Liu G, Wu D, Shi L, Lau W, Wu M, Shen F (2013). Prognostic nomogram for intrahepatic cholangiocarcinoma after partial hepatectomy. J Clin Oncol.

[6] Cancer Research UK. The 20 most common causes of cancer death in 2014. http://www.cancerresearchuk.org/sites/default/files/cstream-node/mort_20common_mf_M14_2.pdf.

[7] Kamisawa T, Wood LD, Itoi T, Takaori K (2016). Pancreatic cancer. Lancet.

[8] Valle J, Wasan H, Palmer DH, Cunningham D, Anthoney A, Maraveyas A, Madhusudan S, Iveson T, Hughes S, Pereira SP, Roughton M, Bridgewater J (2010). Cisplatin plus gemcitabine versus gemcitabine for biliary tract cancer. N Engl J Med.

[9] Imperatori M, D’Onofrio L, Marrucci E, Pantano F, Zoccoli A, Tonini G (2015). Neoadjuvant treatment of biliary tract cancer: state-of-the-art and new perspectives. Hepatic Oncol.

[10] Hughes T, O’Connor T, Techasen A (2017). Opisthorchiasis and cholangiocarcinoma in Southeast Asia: an unresolved problem. Int J Gen Med.

[11] Lennon AM, Wolfgang CL, Canto MI, Klein AP, Herman JM, Goggins M, Fishman EK, Kamel I, Weiss MJ, Diaz LA, Papadopoulos N, Kinzler KW, Vogelstein B, Hruban RH (2014). The early detection of pancreatic cancer: what will it take to diagnose and treat curable pancreatic neoplasia?. Cancer Res.

[12] Pavlovic Markovic A, Rosch T, Alempijevic T (2012). Endoscopic ultrasound for differential diagnosis of duodenal lesions. Ultraschall Med.

[13] Kaur S, Baine MJ, Jain M, Sasson AR, Batra SK (2012). Early diagnosis of pancreatic cancer: challenges and new developments. Biomark Med.

[14] Morris-Stiff G, Taylor MA (2012). Ca19-9 and pancreatic cancer: is it really that good?. J Gastrointest Oncol.

[15] Loosen SH, Roderburg C, Kauertz KL, Koch A, Vucur M, Schneider AT, Binnebösel M, Ulmer TF, Lurje G, Schoening W, Tacke F, Trautwein C, Longerich T, Dejong CH, Neumann UP, Luedde T (2017). CEA but not CA19-9 is an independent prognostic factor in patients undergoing resection of cholangiocarcinoma. Sci Rep.

[16] Volik S, Alcaide M, Morin RD, Collins C (2016). Cell-free DNA (cfDNA): clinical significance and utility in cancer shaped by emerging technologies. Mol Cancer Res.

[17] Kim ST, Lira M, Deng S (2015). PIK3CA mutation detection in metastatic biliary cancer using cell-free DNA. Oncotarget.

[18] Kojima M, Sudo H, Kawauchi J (2015). MicroRNA markers for the diagnosis of pancreatic and biliary-tract cancers. PLoS One.

[19] Guzzetta AA, Pisanic Ii TR, Sharma P (2014). The promise of methylation on beads for cancer detection and treatment. Expert Rev Mol Diagn.

[20] Wang X, Chen S, Jia W (2016). Metabolomics in cancer biomarker research. Curr Pharmacol Reports.

[21] Yi JM, Guzzetta AA, Bailey VJ, Downing SR, van Neste L, Chiappinelli KB, Keeley BP, Stark A, Herrera A, Wolfgang C, Pappou EP, Iacobuzio-Donahue CA, Goggins MG, Herman JG, Wang TH, Baylin SB, Ahuja N (2013). Novel methylation biomarker panel for the early detection of pancreatic cancer. Clin Cancer Res.

[22] Sharma U, Baek HM, Su MY, Jagannathan NR (2011). In vivo (1)H MRS in the assessment of the therapeutic response of breast cancer patients. NMR Biomed.

[23] Serkova NJ, Spratlin JL, Eckhardt SG (2007). NMR-based metabolomics: translational application and treatment of cancer. Curr Opin Mol Ther.

[24] Kline EE, Treat EG, Averna TA, Davis MS, Smith AY, Sillerud LO (2006). Citrate concentrations in human seminal fluid and expressed prostatic fluid determined via 1H nuclear magnetic resonance spectroscopy outperform prostate specific antigen in prostate cancer detection. J Urol.

[25] Wang H, Tso V, Wong C, Sadowski D, Fedorak RN (2014). Development and validation of a highly sensitive urine-based test to identify patients with colonic adenomatous polyps. Clin Transl Gastroenterol.

[26] Lu M, Zhan X (2018). The crucial role of multiomic approach in cancer research and clinically relevant outcomes. EPMA J.

[27] Cheng T, Zhan X (2017). Pattern recognition for predictive, preventive, and personalized medicine in cancer. EPMA J.

[28] Grech G, Zhan X, Yoo BC, Bubnov R, Hagan S, Danesi R, Vittadini G, Desiderio DM (2015). EPMA position paper in cancer: current overview and future perspectives. EPMA J.

[29] Lee JH, Kim KH, Park JW, Chang HJ, Kim BC, Kim SY, Kim KG, Lee ES, Kim DY, Oh JH, Yoo BC, Kim IH (2014). Low-mass-ion discriminant equation: a new concept for colorectal cancer screening. Int J Cancer.

[30] Kim SC, Kim MK, Kim YH, Ahn SA, Kim KH, Kim K, Kim WK, Lee JH, Cho JY, Yoo BC (2014). Differential levels of l-homocysteic acid and lysophosphatidylcholine (16,0) in sera of patients with ovarian cancer. Oncol Lett.

[31] Lee JH, Yoo BC, Kim YH, Ahn SA, Yeo SG, Cho JY, Kim KH, Kim SC (2016). Low-mass-ion discriminant equation (LOME) for ovarian cancer screening. BioData Min.

[32] Jantscheff P, Schlesinger M, Fritzsche J, Taylor LA, Graeser R, Kirfel G, Furst DO, Massing U, Bendas G (2011). Lysophosphatidylcholine pretreatment reduces VLA-4 and P-Selectin-mediated b16.f10 melanoma cell adhesion in vitro and inhibits metastasis-like lung invasion in vivo. Mol Cancer Ther.

[33] Okita M, Gaudette DC, Mills GB, Holub BJ (1997). Elevated levels and altered fatty acid composition of plasma lysophosphatidylcholine(lysoPC) in ovarian cancer patients. Int J Cancer.

[34] Kühn T, Floegel A, Sookthai D, Johnson T, Rolle-Kampczyk U, Otto W, von Bergen M, Boeing H, Kaaks R (2016). Higher plasma levels of lysophosphatidylcholine 18:0 are related to a lower risk of common cancers in a prospective metabolomics study. BMC Med.

[35] Xu J, Chen Y, Zhang R, Song Y, Cao J, Bi N, Wang J, He J, Bai J, Dong L, Wang L, Zhan Q, Abliz Z (2013). Global and targeted metabolomics of esophageal squamous cell carcinoma discovers potential diagnostic and therapeutic biomarkers. Mol Cell Proteomics.

[36] Thomas R, Bastian J, Manuel G (2016). The molecular mechanism by which saturated lysophosphatidylcholine attenuates the metastatic capacity of melanoma cells. FEBS Open Bio.

[37] Zhang T, Wu X, Yin M, Fan L, Zhang H, Zhao F, Zhang W, Ke C, Zhang G, Hou Y, Zhou X, Lou G, Li K (2012). Discrimination between malignant and benign ovarian tumors by plasma metabolomic profiling using ultra performance liquid chromatography/mass spectrometry. Clin Chim Acta.

[38] Zhang H, Ge T, Cui X, Hou Y, Ke C, Yang M, Yang K, Wang J, Guo B, Zhang F, Lou G, Li K (2015). Prediction of advanced ovarian cancer recurrence by plasma metabolic profiling. Mol BioSyst.

[39] Del Boccio P, Perrotti F, Rossi C (2017). Serum lipidomic study reveals potential early biomarkers for predicting response to chemoradiation therapy in advanced rectal cancer: a pilot study. Adv Radiat Oncol.

[40] Yin M-Z, Tan S, Li X, Hou Y, Cao G, Li K, Kou J, Lou G (2016). Identification of phosphatidylcholine and lysophosphatidylcholine as novel biomarkers for cervical cancers in a prospective cohort study. Tumour Biol.

[41] Zhao Z, Xiao Y, Elson P, Tan H, Plummer SJ, Berk M, Aung PP, Lavery IC, Achkar JP, Li L, Casey G, Xu Y (2007). Plasma lysophosphatidylcholine levels: potential biomarkers for colorectal cancer. J Clin Oncol.

[42] Hoekstra HJ, Wobbes T, Heineman E, Haryono S, Aryandono T, Balch CM (2016). Fighting global disparities in cancer care: a surgical oncology view. Ann Surg Oncol.

[43] Takayuki G, Naoki T, Takahiro I (2015). Decreased expression of lysophosphatidylcholine (16,0/OH) in high resolution imaging mass spectrometry independently predicts biochemical recurrence after surgical treatment for prostate cancer. Prostate.

[44] Rodriguez-Peralvarez M, Tsochatzis E, Naveas MC (2013). Reduced exposure to calcineurin inhibitors early after liver transplantation prevents recurrence of hepatocellular carcinoma. J Hepatol.

[45] Miyazaki M, Ohtsuka M, Miyakawa S (2015). Classification of biliary tract cancers established by the Japanese Society of Hepato-Biliary-Pancreatic Surgery: 3(rd) English edition. J Biliary Pancreat Sci.

[46] Ponisch W, Mitrou PS, Merkle K (2006). Treatment of bendamustine and prednisone in patients with newly diagnosed multiple myeloma results in superior complete response rate, prolonged time to treatment failure and improved quality of life compared to treatment with melphalan and prednisone—a randomized phase III study of the East German Study Group of Hematology and Oncology (OSHO). J Cancer Res Clin Oncol.

[47] Kim K-H, Joo J, Park B (2017). Reduced levels of N′-methyl-2-pyridone-5-carboxamide and lysophosphatidylcholine 16:0 in the serum of patients with intrahepatic cholangiocarcinoma, and the correlation with recurrence-free survival. Oncotarget.

[48] Barbayianni E, Kaffe E, Aidinis V, Kokotos G (2015). Autotaxin, a secreted lysophospholipase D, as a promising therapeutic target in chronic inflammation and cancer. Prog Lipid Res.

