# Supplementary material for: Individualized metabolic profiling stratifies pancreatic and biliary tract cancer: a useful tool for innovative screening programs and predictive strategies in healthcare
Source: EPMA J. 2018 Aug 17;9(3):287–97. doi: 10.1007/s13167-018-0147-5 (PMC6107458; doi:10.1007/s13167-018-0147-5)
Supplement: Supplementary file 1 — (DOCX 388 kb) [file 13167_2018_147_MOESM1_ESM.docx]

**Supporting Information Table 1** List of subjects with serum samples for deriving LMIs.

| A. Patients with PC | | | | |
| --- | --- | --- | --- | --- |
| Sample  identifier | Sex | Age  (years) | Stage | Status |
| PC001 | F | 82 | II | Resectable |
| PC002 | M | 42 | IV | Metastatic |
| PC003 | M | 54 | IV | Metastatic |
| PC004 | M | 55 | IV | Metastatic |
| PC005 | M | 74 | II | Resectable |
| PC006 | F | 76 | IV | Metastatic |
| PC007 | F | 68 | II | Resectable |
| PC008 | F | 68 | IV | Metastatic |
| PC009 | F | 49 | HRG | Branch duct IPMN |
| PC010 | F | 73 | III | Local |
| PC011 | F | 69 | IV | Metastatic |
| PC012 | M | 52 | IV | Metastatic |
| PC013 | M | 33 | IV | Metastatic |
| PC014 | M | 74 | I | Resectable |
| PC015 | F | 75 | IV | Metastatic |
| PC016 | M | 69 | IV | Metastatic |
| PC017 | F | 51 | HRG | Hypercholesterolemia, IPMN |
| PC018 | M | 80 | HRG | Branch duct IPMN |
| PC019 | M | 40 | II | Resectable |
| PC020 | M | 61 | IV | Metastatic |
| PC021 | F | 65 | IV | Metastatic |
| PC022 | M | 63 | IV | Metastatic |
| PC023 | F | 72 | II | Resectable |
| PC024 | F | 65 | III | Local |
| PC025 | M | 50 | III | Local |
| PC026 | F | 48 | HRG | Branch duct IPMN |
| PC027 | M | 55 | IV | Metastatic |
| PC028 | F | 64 | IV | Metastatic |
| PC029 | F | 58 | IV | Metastatic |
| PC030 | M | 69 | II | Resectable |
| PC031 | F | 49 | IV | Metastatic |
| PC032 | F | 74 | IV | Metastatic |
| PC033 | M | 62 | IV | Metastatic |
| PC034 | M | 72 | HRG | Acute pancreatitis |
| PC035 | F | 66 | II | Resectable |
| PC036 | M | 62 | IV | Metastatic |
| PC037 | M | 74 | II | Resectable |
| PC038 | M | 69 | HRG | Branch duct IPMN |
| PC039 | M | 74 | HRG | IPMN, uncinate |
| PC040 | F | 65 | HRG | Branch duct IPMN |
| PC041 | M | 67 | HRG | Combined type IPMN |
| PC042 | M | 63 | HRG | IPMN, branch duct type |
| PC043 | F | 59 | IV | Metastatic |
| PC044 | M | 61 | HRG | Chronic pancreatitis |
| PC045 | M | 64 | IV | Metastatic |
| PC046 | M | 71 | IV | Metastatic |
| PC047 | F | 68 | IV | Metastatic |
| PC048 | M | 63 | IV | Metastatic |
| PC049 | M | 63 | IV | Metastatic |
| PC050 | M | 79 | II | Resectable |
| PC051 | M | 69 | II | Resectable |

LMI low-mass ion, PC pancreatic cancer, M male, F female, HRG high risk group, IPMN intraductal papillary mucinous neoplasm

| B. Patients with BTC | | | | |
| --- | --- | --- | --- | --- |
| Sample  identifier | Sex | Age  (years) | Stage | Status |
| BTC001 | F | 82 | HRG | Multiple IHD stones |
| BTC002 | M | 53 | HRG | Chronic hepatitis B |
| BTC003 | F | 45 | HRG | Chronic hepatitis B |
| BTC004 | F | 79 | HRG | Chronic hepatitis B |
| BTC005 | M | 57 | HRG | Chronic hepatitis B |
| BTC006 | M | 63 | HRG | Liver cirrhosis |
| BTC007 | M | 68 | HRG | Chronic hepatitis B |
| BTC008 | M | 36 | HRG | Chronic hepatitis B |
| BTC009 | F | 47 | HRG | Chronic hepatitis B |
| BTC010 | M | 53 | HRG | Chronic hepatitis B |
| BTC011 | F | 59 | HRG | Chronic hepatitis B |
| BTC012 | F | 50 | HRG | Chronic hepatitis B |
| BTC013 | M | 60 | HRG | Chronic hepatitis B |
| BTC014 | F | 54 | HRG | Chronic hepatitis B |
| BTC015 | M | 54 | HRG | Chronic hepatitis B |
| BTC016 | M | 57 | HRG | Chronic hepatitis B |
| BTC017 | M | 78 | III | Local |
| BTC018 | F | 61 | IV | Metastatic |
| BTC019 | M | 61 | HRG | Chronic hepatitis B |
| BTC020 | M | 62 | IV | Metastatic |
| BTC021 | M | 64 | HRG | Chronic hepatitis B |
| BTC022 | M | 63 | HRG | Chronic hepatitis B |
| BTC023 | M | 73 | IV | Metastatic |
| BTC024 | M | 64 | IV | Metastatic |
| BTC025 | M | 75 | IV | Metastatic |
| BTC026 | M | 68 | IV | Metastatic |
| BTC027 | F | 79 | III | Local |
| BTC028 | M | 59 | HRG | Chronic hepatitis B |
| BTC029 | M | 75 | IV | Metastatic |
| BTC030 | F | 77 | IVA | Local |
| BTC031 | F | 53 | HRG | Focal adenomyomatosis at GB fundus |
| BTC032 | M | 56 | HRG | Chronic hepatitis B |
| BTC033 | M | 58 | HRG | Chronic hepatitis B |
| BTC034 | F | 84 | IV | Metastatic |
| BTC035 | F | 74 | II | Resectable |
| BTC036 | M | 55 | HRG | Chronic hepatitis C |
| BTC037 | F | 46 | HRG | GB polyp |
| BTC038 | M | 58 | IV | metastatic |
| BTC039 | M | 71 | IV | metastatic |

BTC biliary tract cancer, M male, F female, HRG high risk group, IHD intra-hepatic duct, GB gallbladder

| C. Healthy individuals | | | | | |
| --- | --- | --- | --- | --- | --- |
| Sample identifier | Sex | Age  (years) | Sample identifier | Sex | Age  (years) |
| Control001 | M | 63 | Control051 | F | 49 |
| Control002 | M | 62 | Control052 | F | 52 |
| Control003 | M | 59 | Control053 | M | 69 |
| Control004 | M | 52 | Control054 | M | 56 |
| Control005 | M | 47 | Control055 | F | 52 |
| Control006 | F | 59 | Control056 | F | 55 |
| Control007 | M | 46 | Control057 | M | 53 |
| Control008 | F | 49 | Control058 | M | 58 |
| Control009 | F | 55 | Control059 | M | 51 |
| Control010 | M | 51 | Control060 | F | 70 |
| Control011 | M | 63 | Control061 | M | 65 |
| Control012 | M | 59 | Control062 | M | 51 |
| Control013 | M | 56 | Control063 | M | 52 |
| Control014 | M | 52 | Control064 | M | 51 |
| Control015 | M | 66 | Control065 | M | 60 |
| Control016 | F | 60 | Control066 | M | 63 |
| Control017 | M | 64 | Control067 | M | 59 |
| Control018 | F | 49 | Control068 | M | 56 |
| Control019 | M | 61 | Control069 | M | 52 |
| Control020 | M | 68 | Control070 | M | 66 |
| Control021 | M | 64 | Control071 | M | 64 |
| Control022 | M | 64 | Control072 | F | 49 |
| Control023 | F | 61 | Control073 | M | 61 |
| Control024 | M | 57 | Control074 | M | 68 |
| Control025 | F | 69 | Control075 | M | 64 |
| Control026 | M | 48 | Control076 | M | 64 |
| Control027 | M | 69 | Control077 | F | 61 |
| Control028 | M | 50 | Control078 | M | 57 |
| Control029 | M | 57 | Control079 | F | 69 |
| Control030 | M | 52 | Control080 | M | 48 |
| Control031 | M | 59 | Control081 | M | 58 |
| Control032 | F | 54 | Control082 | F | 70 |
| Control033 | M | 42 | Control083 | F | 50 |
| Control034 | F | 50 | Control084 | M | 66 |
| Control035 | F | 56 | Control085 | F | 37 |
| Control036 | F | 54 | Control086 | F | 38 |
| Control037 | M | 63 | Control087 | M | 64 |
| Control038 | M | 47 | Control088 | M | 59 |
| Control039 | M | 60 | Control089 | M | 49 |
| Control040 | M | 66 | Control090 | M | 69 |
| Control041 | M | 62 | Control091 | M | 50 |
| Control042 | F | 57 | Control092 | M | 57 |
| Control043 | F | 69 | Control093 | M | 51 |
| Control044 | M | 59 | Control094 | M | 59 |
| Control045 | M | 52 | Control095 | F | 54 |
| Control046 | M | 47 | Control096 | M | 42 |
| Control047 | F | 59 | Control097 | F | 56 |
| Control048 | M | 58 | Control098 | F | 54 |
| Control049 | M | 46 | Control099 | M | 63 |
| Control050 | F | 54 | Control100 | M | 47 |

M male, F female

| D. Patients with CRC | | | | | | | |
| --- | --- | --- | --- | --- | --- | --- | --- |
| Sample identifier | Sex | Age  (years) | Stage | Sample identifier | Sex | Age  (years) | Stage |
| CRC001 | F | 79 | 0 | CRC051 | M | 52 | I |
| CRC002 | M | 71 | I | CRC052 | F | 70 | I |
| CRC003 | F | 71 | I | CRC053 | M | 65 | I |
| CRC004 | M | 73 | I | CRC054 | M | 76 | I |
| CRC005 | M | 69 | I | CRC055 | F | 58 | I |
| CRC006 | M | 52 | IIA | CRC056 | M | 52 | IIA |
| CRC007 | F | 67 | IIA | CRC057 | M | 42 | IIIB |
| CRC008 | F | 76 | IIA | CRC058 | M | 64 | IIA |
| CRC009 | F | 70 | IIA | CRC059 | M | 42 | IIA |
| CRC010 | M | 48 | IIIA | CRC060 | M | 68 | IIIA |
| CRC011 | F | 47 | IIIB | CRC061 | F | 53 | IIIB |
| CRC012 | M | 63 | IIIB | CRC062 | F | 82 | IIIB |
| CRC013 | M | 71 | IIIB | CRC063 | M | 62 | IIIB |
| CRC014 | M | 68 | IIIB | CRC064 | F | 79 | IIIB |
| CRC015 | M | 59 | IIIC | CRC065 | M | 70 | IIIC |
| CRC016 | F | 65 | IV | CRC066 | M | 54 | IIIB |
| CRC017 | M | 45 | IV | CRC067 | M | 48 | I |
| CRC018 | M | 64 | I | CRC068 | F | 58 | I |
| CRC019 | F | 68 | I | CRC069 | M | 64 | I |
| CRC020 | F | 50 | I | CRC070 | M | 48 | I |
| CRC021 | M | 49 | I | CRC071 | M | 68 | I |
| CRC022 | F | 46 | IIA | CRC072 | M | 73 | IIA |
| CRC023 | M | 77 | IIA | CRC073 | M | 40 | IIA |
| CRC024 | M | 78 | IIA | CRC074 | F | 66 | IIA |
| CRC025 | M | 58 | IIA | CRC075 | F | 48 | IIB |
| CRC026 | F | 50 | IIIA | CRC076 | F | 66 | IIIA |
| CRC027 | M | 80 | IIIB | CRC077 | F | 44 | IIIB |
| CRC028 | F | 36 | IIIB | CRC078 | F | 70 | IIIB |
| CRC029 | F | 71 | IIIB | CRC079 | M | 56 | IIIB |
| CRC030 | F | 55 | IIIB | CRC080 | M | 50 | IIIB |
| CRC031 | M | 60 | IIIB | CRC081 | M | 68 | IIIC |
| CRC032 | F | 49 | IIIC | CRC082 | F | 52 | IIIC |
| CRC033 | M | 81 | IVA | CRC083 | M | 74 | IVB |
| CRC034 | M | 53 | IVA | CRC084 | M | 70 | IIIB |
| CRC035 | F | 68 | IV | CRC085 | M | 49 | I |
| CRC036 | F | 70 | I | CRC086 | F | 53 | I |
| CRC037 | M | 51 | I | CRC087 | M | 57 | I |
| CRC038 | M | 61 | I | CRC088 | F | 70 | I |
| CRC039 | F | 49 | I | CRC089 | M | 56 | IIA |
| CRC040 | M | 64 | IIA | CRC090 | F | 49 | IIA |
| CRC041 | M | 67 | IIA | CRC091 | M | 68 | IIA |
| CRC042 | M | 73 | IIA | CRC092 | F | 48 | IIIB |
| CRC043 | M | 69 | IIA | CRC093 | F | 59 | IIIB |
| CRC044 | F | 73 | IIIA | CRC094 | F | 73 | IIIB |
| CRC045 | M | 73 | IIIB | CRC095 | M | 71 | IIIB |
| CRC046 | F | 33 | IIIB | CRC096 | F | 70 | IIIB |
| CRC047 | M | 56 | IIIB | CRC097 | F | 72 | IIIB |
| CRC048 | M | 62 | IIIB | CRC098 | F | 45 | IIIC |
| CRC049 | M | 56 | IIIB | CRC099 | M | 66 | IIIC |
| CRC050 | M | 54 | IIIC | CRC100 | F | 54 | IVB |

CRC colorectal cancer, M male, F female

| E. Patients with OVC | | | |
| --- | --- | --- | --- |
| Sample identifier | Age  (years) | Stage | Pathology |
| OVC001 | 70 | IIIA1 | High grade serous carcinoma |
| OVC002 | 52 | IVA | High grade serous carcinoma |
| OVC003 | 45 | IA | Mucinous borderline tumor |
| OVC004 | 56 | IIIB | High grade serous carcinoma |
| OVC005 | 51 | IVB | High grade serous carcinoma |
| OVC006 | 51 | IIIC | High grade serous carcinoma |
| OVC007 | 46 | IIIA1 | Serous adenocarcinoma |
| OVC008 | 58 | IC3 | Endometrioid adenocarcinoma |
| OVC009 | 52 | IA | Hemorrhagic corpus luteum |
| OVC010 | 66 | IIB | Serous adenocarcinoma |
| OVC011 | 54 | IIA | Serous adenocarcinoma, malignant |
| OVC012 | 46 | IC3 | Strumal carcinoid |
| OVC013 | 63 | IIIC | Serous carcinoma, malignant |
| OVC014 | 56 | IA | High grade serous carcinoma |
| OVC015 |  | IVB | Reactive cellular changes associated with atrophy |
| OVC016 | 76 | IB | Mucinous carcinoma, malignant |
| OVC017 | 37 | IA | Clear cell carcinoma |
| OVC018 | 44 | IC3 | Low grade serous carcinoma |
| OVC019 | 46 | IIIC | High grade serous carcinoma |
| OVC020 | 40 | IA | Clear cell carcinoma |
| OVC021 | 41 |  | Metastatic mucinous adenocarcinoma |
| OVC022 | 54 | IB | High grade serous carcinoma, malignant |
| OVC023 | 68 | IIIC | Adenosquamous carcinoma |
| OVC024 | 49 | IIIC | Clear cell carcinoma |
| OVC025 | 54 | IIIB | Mucinous carcinoma |
| OVC026 | 46 | IIIB | High grade serous carcinoma |
| OVC027 | 78 | IIIC | Serous carcinoma |
| OVC028 | 63 | IIIC | High grade serous carcinoma |
| OVC029 | 53 | IVA | Metastatic adenocarcinoma |
| OVC030 | 42 | IA |  |

OVC ovarian cancer

| F. Institutions from which samples were gathered | |  |
| --- | --- | --- |
|  | Address | Sample groups |
| National Cancer Center Hospital | 323 Ilsan-ro, Ilsandong-gu, Goyang-si, Gyeonggi-do, 10408, Korea | PC/BTC/Control/CRC |
| Korea University Guro Hospital | 148 Gurodong-ro, Guro-gu, Seoul, 08308, Korea | OVC |

**Supporting Information Table 2** Mass values and retention times of all 6,724 LMIs

| No. | Mass value (*m/z*) | Ret. time  (min) | No. | Mass value (*m/z*) | Ret. time  (min) | No. | Mass value (*m/z*) | Ret. time  (min) | No. | Mass value (*m/z*) | Ret. time  (min) |
| --- | --- | --- | --- | --- | --- | --- | --- | --- | --- | --- | --- |
| 1 | 53.0030 | 9.96 | 1682 | 327.2277 | 19.43 | 3363 | 504.3058 | 18.05 | 5044 | 623.5933 | 8.30 |
| 2 | 53.0032 | 9.26 | 1683 | 327.2299 | 19.35 | 3364 | 504.3060 | 14.97 | 5045 | 623.5978 | 10.06 |
| 3 | 53.0033 | 15.37 | 1684 | 327.6834 | 1.24 | 3365 | 504.3145 | 21.20 | 5046 | 623.5980 | 8.47 |
| 4 | 53.0035 | 15.61 | 1685 | 328.2319 | 8.36 | 3366 | 504.3370 | 19.54 | 5047 | 623.8295 | 8.31 |
| 5 | 53.0039 | 1.02 | 1686 | 328.2340 | 8.53 | 3367 | 504.3370 | 8.94 | 5048 | 623.8340 | 8.47 |
| 6 | 53.0039 | 10.05 | 1687 | 328.9040 | 0.66 | 3368 | 504.3375 | 16.09 | 5049 | 623.8382 | 10.19 |
| 7 | 53.0040 | 1.01 | 1688 | 329.0024 | 13.88 | 3369 | 504.3390 | 10.61 | 5050 | 623.8389 | 8.76 |
| 8 | 53.0041 | 17.70 | 1689 | 329.0027 | 14.04 | 3370 | 504.3404 | 9.22 | 5051 | 623.8416 | 8.15 |
| 9 | 53.0041 | 19.20 | 1690 | 329.0027 | 13.94 | 3371 | 504.6112 | 9.41 | 5052 | 623.8416 | 8.08 |
| 10 | 53.0042 | 10.23 | 1691 | 329.0032 | 17.06 | 3372 | 504.6117 | 7.83 | 5053 | 623.8549 | 10.04 |
| 11 | 53.0042 | 1.03 | 1692 | 329.0041 | 17.06 | 3373 | 504.6152 | 9.66 | 5054 | 624.0794 | 8.30 |
| 12 | 53.0042 | 16.31 | 1693 | 329.0048 | 14.00 | 3374 | 505.2780 | 19.25 | 5055 | 624.0832 | 10.09 |
| 13 | 53.0043 | 19.15 | 1694 | 329.0056 | 17.09 | 3375 | 505.2811 | 19.30 | 5056 | 624.0849 | 10.19 |
| 14 | 53.0043 | 10.06 | 1695 | 329.0088 | 14.23 | 3376 | 505.3401 | 11.03 | 5057 | 624.0875 | 10.06 |
| 15 | 53.0044 | 18.41 | 1696 | 329.0095 | 13.77 | 3377 | 505.3405 | 10.93 | 5058 | 624.0882 | 8.06 |
| 16 | 53.0044 | 18.32 | 1697 | 329.0278 | 0.87 | 3378 | 505.4559 | 9.90 | 5059 | 624.0916 | 8.49 |
| 17 | 53.0044 | 19.32 | 1698 | 329.2276 | 14.07 | 3379 | 505.6622 | 9.87 | 5060 | 624.3343 | 10.03 |
| 18 | 53.0045 | 19.08 | 1699 | 329.2300 | 17.33 | 3380 | 506.2349 | 17.39 | 5061 | 624.3451 | 8.05 |
| 19 | 53.0045 | 15.28 | 1700 | 329.2303 | 14.15 | 3381 | 506.2703 | 10.40 | 5062 | 624.6020 | 10.17 |
| 20 | 53.0048 | 0.62 | 1701 | 329.2312 | 17.39 | 3382 | 506.2960 | 10.63 | 5063 | 625.3138 | 10.97 |
| 21 | 53.0049 | 0.86 | 1702 | 329.2313 | 14.42 | 3383 | 506.2995 | 11.35 | 5064 | 625.4495 | 19.29 |
| 22 | 53.0051 | 19.37 | 1703 | 329.2326 | 14.03 | 3384 | 506.5211 | 10.40 | 5065 | 625.6957 | 8.61 |
| 23 | 53.0051 | 1.24 | 1704 | 329.2490 | 17.85 | 3385 | 506.7719 | 10.40 | 5066 | 626.5858 | 8.04 |
| 24 | 53.0051 | 22.08 | 1705 | 331.0043 | 17.10 | 3386 | 507.2833 | 8.53 | 5067 | 626.5872 | 10.04 |
| 25 | 53.0052 | 5.97 | 1706 | 331.1075 | 15.90 | 3387 | 507.2837 | 10.62 | 5068 | 626.5884 | 8.48 |
| 26 | 53.0052 | 6.15 | 1707 | 331.2336 | 1.72 | 3388 | 507.2845 | 10.50 | 5069 | 626.5905 | 10.01 |
| 27 | 53.0052 | 6.22 | 1708 | 331.2351 | 1.41 | 3389 | 507.2870 | 8.74 | 5070 | 626.5916 | 9.83 |
| 28 | 53.0052 | 15.67 | 1709 | 332.0672 | 15.23 | 3390 | 508.2832 | 10.61 | 5071 | 626.5918 | 10.18 |
| 29 | 53.0053 | 18.45 | 1710 | 332.1354 | 8.91 | 3391 | 508.3334 | 18.54 | 5072 | 626.5929 | 8.38 |
| 30 | 53.0053 | 17.84 | 1711 | 332.3301 | 15.85 | 3392 | 508.3365 | 15.38 | 5073 | 626.5933 | 8.18 |
| 31 | 53.0053 | 17.77 | 1712 | 332.8791 | 2.62 | 3393 | 508.3401 | 15.34 | 5074 | 626.5941 | 10.06 |
| 32 | 53.0054 | 7.35 | 1713 | 332.8865 | 2.71 | 3394 | 508.3717 | 19.99 | 5075 | 626.5945 | 8.70 |
| 33 | 53.0054 | 19.22 | 1714 | 334.1445 | 6.03 | 3395 | 508.3746 | 16.43 | 5076 | 626.5951 | 9.87 |
| 34 | 53.0055 | 16.26 | 1715 | 334.6931 | 7.60 | 3396 | 508.3765 | 16.34 | 5077 | 626.5963 | 8.94 |
| 35 | 53.0055 | 17.73 | 1716 | 334.9153 | 0.70 | 3397 | 508.5759 | 10.05 | 5078 | 626.6081 | 10.01 |
| 36 | 53.0055 | 16.34 | 1717 | 336.0131 | 0.83 | 3398 | 508.8529 | 0.75 | 5079 | 626.8381 | 10.04 |
| 37 | 53.0062 | 1.10 | 1718 | 336.2193 | 21.58 | 3399 | 508.9070 | 10.02 | 5080 | 626.8381 | 8.70 |
| 38 | 53.0062 | 1.19 | 1719 | 337.1650 | 19.22 | 3400 | 508.9298 | 8.64 | 5081 | 626.8404 | 8.18 |
| 39 | 53.0063 | 0.07 | 1720 | 337.1653 | 19.07 | 3401 | 508.9331 | 7.45 | 5082 | 626.8415 | 9.98 |
| 40 | 53.0077 | 10.12 | 1721 | 337.1660 | 18.18 | 3402 | 508.9354 | 8.94 | 5083 | 626.8425 | 9.84 |
| 41 | 53.0077 | 1.20 | 1722 | 337.1683 | 18.22 | 3403 | 508.9369 | 7.33 | 5084 | 626.8448 | 9.96 |
| 42 | 53.0096 | 19.25 | 1723 | 337.1698 | 18.44 | 3404 | 508.9394 | 8.51 | 5085 | 626.8458 | 9.83 |
| 43 | 53.0100 | 0.07 | 1724 | 337.2261 | 21.57 | 3405 | 508.9398 | 7.68 | 5086 | 626.8474 | 8.21 |
| 44 | 55.0559 | 1.28 | 1725 | 338.0523 | 5.87 | 3406 | 508.9413 | 8.92 | 5087 | 626.8487 | 10.00 |
| 45 | 56.0510 | 1.44 | 1726 | 338.1411 | 8.91 | 3407 | 508.9415 | 7.87 | 5088 | 626.8497 | 8.48 |
| 46 | 56.9663 | 21.70 | 1727 | 338.1492 | 8.77 | 3408 | 509.0611 | 8.93 | 5089 | 626.8497 | 8.51 |
| 47 | 56.9678 | 0.70 | 1728 | 338.1868 | 6.12 | 3409 | 509.0613 | 9.01 | 5090 | 626.8505 | 8.94 |
| 48 | 58.0289 | 6.09 | 1729 | 339.1782 | 17.67 | 3410 | 509.0635 | 8.64 | 5091 | 626.8506 | 8.14 |
| 49 | 58.0313 | 5.89 | 1730 | 339.2291 | 19.02 | 3411 | 509.0643 | 7.63 | 5092 | 627.0052 | 0.83 |
| 50 | 58.0317 | 6.37 | 1731 | 339.2343 | 17.96 | 3412 | 509.0667 | 7.45 | 5093 | 627.0734 | 8.96 |
| 51 | 58.0680 | 0.82 | 1732 | 339.2368 | 19.09 | 3413 | 509.0670 | 8.61 | 5094 | 627.0920 | 9.97 |
| 52 | 59.0485 | 18.36 | 1733 | 340.1659 | 9.98 | 3414 | 509.0679 | 7.60 | 5095 | 627.0933 | 10.07 |
| 53 | 59.0490 | 19.22 | 1734 | 340.1730 | 9.95 | 3415 | 509.0680 | 9.62 | 5096 | 627.0933 | 8.48 |
| 54 | 59.0495 | 10.01 | 1735 | 340.6947 | 7.18 | 3416 | 509.0691 | 8.34 | 5097 | 627.0943 | 8.14 |
| 55 | 59.0496 | 18.88 | 1736 | 340.6973 | 7.44 | 3417 | 509.0701 | 7.42 | 5098 | 627.0946 | 8.21 |
| 56 | 59.0502 | 19.26 | 1737 | 340.6984 | 7.66 | 3418 | 509.0708 | 8.95 | 5099 | 627.0947 | 8.18 |
| 57 | 59.0506 | 1.10 | 1738 | 341.1075 | 11.87 | 3419 | 509.0709 | 8.99 | 5100 | 627.0954 | 10.01 |
| 58 | 59.0515 | 0.68 | 1739 | 341.1681 | 9.99 | 3420 | 509.2558 | 9.64 | 5101 | 627.0959 | 10.04 |
| 59 | 59.0515 | 0.06 | 1740 | 341.2057 | 21.57 | 3421 | 509.2619 | 8.99 | 5102 | 627.0969 | 8.51 |
| 60 | 59.0515 | 7.91 | 1741 | 342.0537 | 0.87 | 3422 | 509.2644 | 8.61 | 5103 | 627.0972 | 10.00 |
| 61 | 59.0516 | 19.55 | 1742 | 342.2625 | 11.39 | 3423 | 509.2646 | 7.31 | 5104 | 627.1001 | 9.83 |
| 62 | 59.0516 | 1.18 | 1743 | 342.2627 | 13.94 | 3424 | 509.2651 | 9.04 | 5105 | 627.1002 | 9.99 |
| 63 | 59.0516 | 16.22 | 1744 | 342.2638 | 11.63 | 3425 | 509.2661 | 10.06 | 5106 | 627.1030 | 8.70 |
| 64 | 59.0516 | 5.25 | 1745 | 343.1418 | 15.69 | 3426 | 509.2676 | 7.65 | 5107 | 627.3405 | 9.84 |
| 65 | 59.0516 | 3.37 | 1746 | 343.2115 | 15.63 | 3427 | 509.2679 | 8.96 | 5108 | 627.3415 | 8.14 |
| 66 | 59.0516 | 11.49 | 1747 | 343.2236 | 19.18 | 3428 | 509.2680 | 8.93 | 5109 | 627.3416 | 8.38 |
| 67 | 59.0516 | 0.66 | 1748 | 343.2272 | 18.68 | 3429 | 509.2684 | 7.60 | 5110 | 627.3425 | 10.02 |
| 68 | 59.0516 | 10.30 | 1749 | 343.2970 | 15.68 | 3430 | 509.2693 | 7.87 | 5111 | 627.3432 | 8.70 |
| 69 | 59.0516 | 1.17 | 1750 | 344.0415 | 0.86 | 3431 | 509.2697 | 8.35 | 5112 | 627.3454 | 8.96 |
| 70 | 59.0516 | 7.04 | 1751 | 344.1064 | 9.98 | 3432 | 509.2703 | 8.64 | 5113 | 627.3457 | 8.17 |
| 71 | 59.0516 | 1.04 | 1752 | 344.2271 | 7.94 | 3433 | 509.2708 | 7.70 | 5114 | 627.3467 | 10.00 |
| 72 | 59.0516 | 1.10 | 1753 | 344.2274 | 8.10 | 3434 | 509.2740 | 7.47 | 5115 | 627.3497 | 10.01 |
| 73 | 59.0516 | 8.42 | 1754 | 344.2275 | 7.97 | 3435 | 509.2804 | 8.00 | 5116 | 627.3502 | 10.04 |
| 74 | 59.0517 | 1.25 | 1755 | 344.2287 | 9.16 | 3436 | 509.2811 | 7.95 | 5117 | 627.4788 | 20.91 |
| 75 | 59.0517 | 18.21 | 1756 | 344.2535 | 9.83 | 3437 | 509.2834 | 7.13 | 5118 | 627.4815 | 17.24 |
| 76 | 59.0517 | 19.11 | 1757 | 344.2539 | 10.11 | 3438 | 509.2840 | 7.96 | 5119 | 627.5842 | 10.18 |
| 77 | 59.0517 | 18.43 | 1758 | 344.2572 | 10.03 | 3439 | 509.4635 | 10.06 | 5120 | 627.5864 | 10.01 |
| 78 | 59.0517 | 17.85 | 1759 | 344.2584 | 8.47 | 3440 | 509.4655 | 9.02 | 5121 | 627.5877 | 9.99 |
| 79 | 59.0517 | 0.87 | 1760 | 344.2586 | 10.23 | 3441 | 509.4674 | 8.37 | 5122 | 627.5888 | 8.94 |
| 80 | 59.0518 | 27.49 | 1761 | 344.2593 | 9.99 | 3442 | 509.4676 | 7.41 | 5123 | 627.5901 | 10.06 |
| 81 | 59.0520 | 15.26 | 1762 | 344.2774 | 11.76 | 3443 | 509.4678 | 7.45 | 5124 | 627.5915 | 8.48 |
| 82 | 59.0526 | 22.13 | 1763 | 344.2775 | 14.38 | 3444 | 509.4681 | 8.61 | 5125 | 627.5940 | 10.04 |
| 83 | 59.0526 | 10.21 | 1764 | 344.2787 | 11.99 | 3445 | 509.4687 | 7.61 | 5126 | 627.5963 | 8.18 |
| 84 | 59.0527 | 16.28 | 1765 | 344.2789 | 11.67 | 3446 | 509.4748 | 8.96 | 5127 | 627.5982 | 9.83 |
| 85 | 59.0527 | 6.32 | 1766 | 344.8769 | 21.69 | 3447 | 509.5959 | 8.82 | 5128 | 627.6001 | 8.17 |
| 86 | 59.0527 | 1.04 | 1767 | 344.8774 | 0.65 | 3448 | 509.6651 | 8.64 | 5129 | 627.8234 | 9.96 |
| 87 | 59.0537 | 10.21 | 1768 | 345.1367 | 14.35 | 3449 | 509.6656 | 8.92 | 5130 | 627.8331 | 8.18 |
| 88 | 59.0546 | 19.25 | 1769 | 345.2575 | 10.00 | 3450 | 509.6663 | 7.60 | 5131 | 627.8363 | 8.81 |
| 89 | 59.0546 | 19.19 | 1770 | 345.2592 | 9.81 | 3451 | 509.6683 | 7.45 | 5132 | 627.8422 | 8.64 |
| 90 | 59.0549 | 16.48 | 1771 | 345.2602 | 10.03 | 3452 | 509.6690 | 8.96 | 5133 | 627.8435 | 8.97 |
| 91 | 59.0556 | 18.97 | 1772 | 345.7298 | 10.29 | 3453 | 509.6719 | 8.61 | 5134 | 627.8485 | 10.04 |
| 92 | 59.0568 | 19.19 | 1773 | 345.7324 | 10.28 | 3454 | 509.7807 | 7.96 | 5135 | 627.8633 | 9.83 |
| 93 | 59.0570 | 0.06 | 1774 | 345.7331 | 10.46 | 3455 | 509.7865 | 7.13 | 5136 | 628.1027 | 10.06 |
| 94 | 59.0570 | 10.13 | 1775 | 345.7374 | 10.31 | 3456 | 509.8634 | 8.93 | 5137 | 628.3335 | 8.62 |
| 95 | 59.0571 | 25.67 | 1776 | 346.1231 | 13.77 | 3457 | 509.8701 | 7.60 | 5138 | 628.8601 | 0.74 |
| 96 | 61.0096 | 21.64 | 1777 | 346.1231 | 16.68 | 3458 | 509.8721 | 8.64 | 5139 | 629.2958 | 5.00 |
| 97 | 61.0100 | 0.70 | 1778 | 346.2290 | 9.23 | 3459 | 509.8723 | 8.62 | 5140 | 629.2990 | 5.52 |
| 98 | 61.0106 | 21.70 | 1779 | 347.0896 | 15.06 | 3460 | 510.0583 | 8.98 | 5141 | 629.7210 | 10.96 |
| 99 | 61.0147 | 1.44 | 1780 | 347.1236 | 16.68 | 3461 | 510.0634 | 8.62 | 5142 | 630.1223 | 10.02 |
| 100 | 61.0307 | 1.09 | 1781 | 347.2173 | 16.39 | 3462 | 510.2061 | 0.96 | 5143 | 630.1355 | 8.04 |
| 101 | 61.0426 | 1.02 | 1782 | 347.2182 | 13.30 | 3463 | 510.2639 | 8.64 | 5144 | 630.2989 | 8.02 |
| 102 | 68.9834 | 0.67 | 1783 | 347.2186 | 13.53 | 3464 | 510.2997 | 7.26 | 5145 | 630.3031 | 10.01 |
| 103 | 69.0721 | 17.85 | 1784 | 347.2187 | 14.17 | 3465 | 510.3501 | 16.26 | 5146 | 630.4687 | 9.99 |
| 104 | 69.9948 | 21.70 | 1785 | 347.2194 | 16.75 | 3466 | 510.3535 | 19.87 | 5147 | 630.4689 | 8.02 |
| 105 | 70.0675 | 1.05 | 1786 | 347.2198 | 13.43 | 3467 | 510.3537 | 19.68 | 5148 | 630.4757 | 10.20 |
| 106 | 70.0676 | 1.07 | 1787 | 347.2211 | 13.28 | 3468 | 510.3540 | 16.14 | 5149 | 630.6348 | 8.04 |
| 107 | 71.9530 | 21.69 | 1788 | 347.2218 | 16.80 | 3469 | 510.3549 | 16.36 | 5150 | 630.6431 | 10.01 |
| 108 | 71.9531 | 0.70 | 1789 | 347.2235 | 13.69 | 3470 | 510.3562 | 16.16 | 5151 | 630.8018 | 8.02 |
| 109 | 71.9539 | 0.67 | 1790 | 347.8508 | 2.74 | 3471 | 510.3563 | 15.99 | 5152 | 631.5737 | 9.87 |
| 110 | 72.0817 | 1.25 | 1791 | 348.2206 | 13.28 | 3472 | 510.3569 | 19.90 | 5153 | 631.5795 | 9.99 |
| 111 | 72.0821 | 1.20 | 1792 | 348.2208 | 16.44 | 3473 | 510.3571 | 19.64 | 5154 | 631.8254 | 8.94 |
| 112 | 76.0769 | 0.82 | 1793 | 348.2232 | 13.53 | 3474 | 510.3572 | 16.40 | 5155 | 631.8290 | 9.87 |
| 113 | 78.0234 | 0.68 | 1794 | 348.9882 | 14.00 | 3475 | 510.3585 | 16.37 | 5156 | 631.8307 | 10.07 |
| 114 | 80.0446 | 18.88 | 1795 | 348.9914 | 17.07 | 3476 | 510.3603 | 16.19 | 5157 | 631.8362 | 8.20 |
| 115 | 80.0449 | 18.98 | 1796 | 349.1802 | 8.05 | 3477 | 510.7614 | 9.37 | 5158 | 632.0807 | 9.87 |
| 116 | 80.0457 | 15.06 | 1797 | 349.1844 | 7.85 | 3478 | 511.2588 | 9.37 | 5159 | 632.0834 | 10.01 |
| 117 | 80.0460 | 19.09 | 1798 | 349.1845 | 9.12 | 3479 | 511.3555 | 19.87 | 5160 | 632.0840 | 9.89 |
| 118 | 80.0469 | 18.28 | 1799 | 349.1846 | 9.17 | 3480 | 511.4668 | 19.00 | 5161 | 632.3324 | 10.03 |
| 119 | 80.0470 | 18.79 | 1800 | 349.1859 | 8.09 | 3481 | 511.4693 | 19.83 | 5162 | 632.3396 | 9.87 |
| 120 | 80.0470 | 19.00 | 1801 | 349.1869 | 9.01 | 3482 | 511.4701 | 2.92 | 5163 | 634.2831 | 12.14 |
| 121 | 80.0470 | 18.89 | 1802 | 350.0824 | 6.91 | 3483 | 511.4726 | 19.42 | 5164 | 634.8733 | 1.02 |
| 122 | 80.0471 | 18.94 | 1803 | 350.0860 | 7.19 | 3484 | 511.4734 | 19.19 | 5165 | 634.8761 | 0.76 |
| 123 | 80.0471 | 19.12 | 1804 | 350.2033 | 8.70 | 3485 | 511.4743 | 8.65 | 5166 | 634.8792 | 0.80 |
| 124 | 80.0480 | 13.16 | 1805 | 350.2036 | 8.31 | 3486 | 511.4743 | 19.97 | 5167 | 635.2959 | 12.14 |
| 125 | 80.0483 | 18.89 | 1806 | 350.2039 | 8.46 | 3487 | 511.4750 | 5.14 | 5168 | 635.3910 | 21.60 |
| 126 | 80.0483 | 17.69 | 1807 | 350.2049 | 8.55 | 3488 | 511.4754 | 20.19 | 5169 | 635.8749 | 0.79 |
| 127 | 80.0484 | 19.37 | 1808 | 350.2051 | 9.77 | 3489 | 511.4786 | 17.55 | 5170 | 636.0716 | 8.62 |
| 128 | 80.0484 | 19.10 | 1809 | 350.2055 | 8.08 | 3490 | 511.4791 | 18.33 | 5171 | 636.0756 | 8.93 |
| 129 | 80.0484 | 19.01 | 1810 | 350.2064 | 10.26 | 3491 | 511.4815 | 6.03 | 5172 | 636.0787 | 8.59 |
| 130 | 80.0485 | 15.25 | 1811 | 350.2065 | 8.28 | 3492 | 511.5073 | 7.71 | 5173 | 636.0795 | 8.88 |
| 131 | 80.0485 | 9.94 | 1812 | 350.2079 | 9.78 | 3493 | 511.6406 | 8.33 | 5174 | 636.0806 | 7.60 |
| 132 | 80.0485 | 19.15 | 1813 | 350.2083 | 9.70 | 3494 | 511.6485 | 8.01 | 5175 | 636.0858 | 8.64 |
| 133 | 80.0485 | 14.88 | 1814 | 350.2086 | 9.73 | 3495 | 511.7461 | 7.69 | 5176 | 636.0859 | 8.95 |
| 134 | 80.0488 | 5.16 | 1815 | 350.2092 | 9.64 | 3496 | 511.9756 | 8.33 | 5177 | 636.0862 | 7.45 |
| 135 | 80.0492 | 15.67 | 1816 | 350.2099 | 10.05 | 3497 | 512.0110 | 7.69 | 5178 | 636.0897 | 8.61 |
| 136 | 80.0497 | 16.38 | 1817 | 350.2106 | 8.82 | 3498 | 512.2730 | 20.62 | 5179 | 636.3247 | 8.91 |
| 137 | 80.0497 | 19.01 | 1818 | 350.2118 | 8.34 | 3499 | 512.3113 | 8.34 | 5180 | 636.3277 | 8.64 |
| 138 | 80.0504 | 0.89 | 1819 | 350.2118 | 9.46 | 3500 | 512.3279 | 12.50 | 5181 | 636.3296 | 7.60 |
| 139 | 80.0504 | 0.65 | 1820 | 350.2144 | 8.43 | 3501 | 512.3281 | 14.57 | 5182 | 636.3303 | 10.14 |
| 140 | 80.0505 | 10.27 | 1821 | 350.8912 | 0.68 | 3502 | 512.3327 | 15.40 | 5183 | 636.3312 | 8.92 |
| 141 | 80.0505 | 6.72 | 1822 | 350.8917 | 0.69 | 3503 | 512.3395 | 17.64 | 5184 | 636.3317 | 7.45 |
| 142 | 80.0505 | 1.14 | 1823 | 350.9857 | 14.00 | 3504 | 512.4802 | 8.93 | 5185 | 636.3318 | 8.96 |
| 143 | 80.0505 | 5.30 | 1824 | 350.9865 | 17.06 | 3505 | 514.2777 | 7.95 | 5186 | 636.3384 | 8.62 |
| 144 | 80.0505 | 6.24 | 1825 | 350.9876 | 17.09 | 3506 | 514.2803 | 8.87 | 5187 | 636.3384 | 8.59 |
| 145 | 80.0505 | 5.98 | 1826 | 351.0990 | 2.43 | 3507 | 514.2814 | 9.64 | 5188 | 636.5769 | 8.58 |
| 146 | 80.0506 | 1.56 | 1827 | 351.2110 | 10.05 | 3508 | 514.2846 | 9.68 | 5189 | 636.5772 | 7.45 |
| 147 | 80.0506 | 6.50 | 1828 | 351.2116 | 9.78 | 3509 | 514.2894 | 9.40 | 5190 | 636.5783 | 7.61 |
| 148 | 80.0506 | 6.18 | 1829 | 351.2129 | 9.46 | 3510 | 514.2895 | 7.80 | 5191 | 636.5839 | 8.64 |
| 149 | 80.0506 | 10.29 | 1830 | 351.2146 | 9.70 | 3511 | 514.4751 | 7.79 | 5192 | 636.5847 | 8.90 |
| 150 | 80.0506 | 6.55 | 1831 | 351.7044 | 7.69 | 3512 | 514.4765 | 9.68 | 5193 | 636.5875 | 8.95 |
| 151 | 80.0506 | 6.08 | 1832 | 352.0426 | 17.17 | 3513 | 514.4792 | 7.92 | 5194 | 636.8188 | 8.62 |
| 152 | 80.0506 | 21.81 | 1833 | 352.1631 | 10.38 | 3514 | 514.4818 | 8.87 | 5195 | 636.8189 | 8.95 |
| 153 | 80.0506 | 1.15 | 1834 | 352.1652 | 8.67 | 3515 | 514.4830 | 9.64 | 5196 | 636.8295 | 8.64 |
| 154 | 80.0506 | 1.05 | 1835 | 352.1940 | 7.53 | 3516 | 514.4871 | 9.57 | 5197 | 636.8375 | 8.72 |
| 155 | 80.0506 | 21.75 | 1836 | 352.1964 | 6.98 | 3517 | 514.4889 | 9.52 | 5198 | 636.8442 | 8.96 |
| 156 | 80.0507 | 21.84 | 1837 | 352.1966 | 7.69 | 3518 | 514.4891 | 8.14 | 5199 | 636.8857 | 0.86 |
| 157 | 80.0507 | 6.62 | 1838 | 352.2114 | 9.77 | 3519 | 514.6768 | 7.77 | 5200 | 637.0577 | 8.93 |
| 158 | 80.0507 | 5.89 | 1839 | 352.6959 | 7.76 | 3520 | 514.6781 | 9.68 | 5201 | 637.0727 | 8.66 |
| 159 | 80.0507 | 5.92 | 1840 | 353.2251 | 21.33 | 3521 | 514.6802 | 8.87 | 5202 | 637.0929 | 8.62 |
| 160 | 80.0507 | 21.79 | 1841 | 354.1841 | 21.64 | 3522 | 514.6805 | 7.95 | 5203 | 638.0173 | 11.23 |
| 161 | 80.0507 | 10.40 | 1842 | 354.1894 | 21.24 | 3523 | 514.6829 | 9.38 | 5204 | 638.3749 | 11.89 |
| 162 | 80.0507 | 6.19 | 1843 | 354.2264 | 21.63 | 3524 | 514.6897 | 9.63 | 5205 | 638.3885 | 11.02 |
| 163 | 80.0507 | 6.75 | 1844 | 355.0533 | 0.88 | 3525 | 514.8734 | 0.78 | 5206 | 638.3959 | 11.21 |
| 164 | 80.0507 | 10.17 | 1845 | 355.0588 | 16.32 | 3526 | 514.8751 | 0.83 | 5207 | 640.0762 | 10.07 |
| 165 | 80.0508 | 21.87 | 1846 | 355.2257 | 16.84 | 3527 | 514.8819 | 9.85 | 5208 | 641.3294 | 10.40 |
| 166 | 80.0508 | 6.81 | 1847 | 355.2260 | 16.96 | 3528 | 514.8913 | 9.63 | 5209 | 641.3337 | 10.28 |
| 167 | 80.0508 | 6.61 | 1848 | 355.2270 | 20.24 | 3529 | 514.8918 | 8.85 | 5210 | 642.6064 | 9.64 |
| 168 | 80.0508 | 19.00 | 1849 | 355.2274 | 16.70 | 3530 | 515.2460 | 13.36 | 5211 | 642.8481 | 9.63 |
| 169 | 80.0508 | 21.74 | 1850 | 355.2285 | 16.83 | 3531 | 515.3008 | 8.62 | 5212 | 642.8564 | 8.85 |
| 170 | 80.0508 | 6.43 | 1851 | 355.2491 | 21.63 | 3532 | 515.3050 | 8.92 | 5213 | 642.8684 | 0.75 |
| 171 | 80.0508 | 1.07 | 1852 | 355.2546 | 21.60 | 3533 | 515.3119 | 15.63 | 5214 | 643.1068 | 9.65 |
| 172 | 80.0508 | 5.36 | 1853 | 355.2607 | 16.41 | 3534 | 515.3205 | 15.52 | 5215 | 643.3331 | 11.89 |
| 173 | 80.0508 | 6.02 | 1854 | 355.2636 | 16.45 | 3535 | 515.3208 | 15.62 | 5216 | 643.8082 | 8.20 |
| 174 | 80.0509 | 19.27 | 1855 | 356.0746 | 0.87 | 3536 | 515.5600 | 8.64 | 5217 | 643.8180 | 9.86 |
| 175 | 80.0509 | 17.88 | 1856 | 356.1711 | 7.94 | 3537 | 515.5612 | 8.92 | 5218 | 643.8292 | 10.05 |
| 176 | 80.0509 | 17.99 | 1857 | 356.2689 | 16.41 | 3538 | 515.8102 | 8.61 | 5219 | 644.3234 | 9.87 |
| 177 | 80.0509 | 15.76 | 1858 | 356.2780 | 11.92 | 3539 | 516.2486 | 13.36 | 5220 | 644.8372 | 0.75 |
| 178 | 80.0509 | 19.12 | 1859 | 356.2828 | 14.36 | 3540 | 516.2954 | 17.68 | 5221 | 648.3870 | 9.72 |
| 179 | 80.0518 | 10.27 | 1860 | 356.6883 | 10.29 | 3541 | 516.3052 | 14.71 | 5222 | 648.3895 | 7.74 |
| 180 | 80.0520 | 19.00 | 1861 | 356.6891 | 10.45 | 3542 | 516.3064 | 17.78 | 5223 | 648.3932 | 9.38 |
| 181 | 80.0522 | 19.12 | 1862 | 356.6906 | 10.31 | 3543 | 516.3083 | 14.83 | 5224 | 648.3975 | 7.86 |
| 182 | 80.0522 | 21.14 | 1863 | 356.6908 | 10.32 | 3544 | 516.3125 | 14.64 | 5225 | 648.4036 | 8.79 |
| 183 | 80.0523 | 15.37 | 1864 | 356.9046 | 0.76 | 3545 | 516.3146 | 14.68 | 5226 | 648.7060 | 8.50 |
| 184 | 80.0532 | 8.38 | 1865 | 357.1268 | 11.73 | 3546 | 516.7531 | 8.44 | 5227 | 648.7270 | 9.37 |
| 185 | 80.0533 | 19.07 | 1866 | 357.1744 | 9.27 | 3547 | 516.7548 | 8.28 | 5228 | 648.7309 | 9.40 |
| 186 | 80.0533 | 19.23 | 1867 | 357.1892 | 10.29 | 3548 | 516.7561 | 8.47 | 5229 | 648.7344 | 7.85 |
| 187 | 80.0534 | 17.87 | 1868 | 357.2761 | 14.84 | 3549 | 516.7589 | 8.35 | 5230 | 648.7355 | 9.72 |
| 188 | 80.0536 | 19.08 | 1869 | 357.2771 | 16.29 | 3550 | 517.0019 | 8.31 | 5231 | 648.7415 | 8.81 |
| 189 | 80.0544 | 21.90 | 1870 | 357.2788 | 17.12 | 3551 | 517.0032 | 8.44 | 5232 | 649.0366 | 8.50 |
| 190 | 80.0545 | 0.07 | 1871 | 357.2789 | 17.96 | 3552 | 517.0047 | 0.79 | 5233 | 649.0576 | 8.82 |
| 191 | 80.0557 | 10.24 | 1872 | 357.2790 | 18.11 | 3553 | 517.0098 | 8.46 | 5234 | 649.0650 | 9.40 |
| 192 | 80.0561 | 19.15 | 1873 | 357.2801 | 16.58 | 3554 | 517.2457 | 13.36 | 5235 | 649.3921 | 9.38 |
| 193 | 80.0562 | 0.08 | 1874 | 357.2816 | 18.04 | 3555 | 517.2471 | 8.46 | 5236 | 649.3952 | 8.79 |
| 194 | 80.0577 | 6.26 | 1875 | 358.0575 | 0.87 | 3556 | 517.2490 | 8.29 | 5237 | 650.8466 | 0.80 |
| 195 | 80.0583 | 1.09 | 1876 | 358.2593 | 13.09 | 3557 | 517.3027 | 14.74 | 5238 | 650.8479 | 0.79 |
| 196 | 81.5212 | 21.70 | 1877 | 358.2820 | 17.96 | 3558 | 517.3055 | 14.83 | 5239 | 651.8965 | 11.72 |
| 197 | 81.5216 | 21.65 | 1878 | 358.2856 | 16.59 | 3559 | 517.3070 | 17.77 | 5240 | 651.9476 | 5.53 |
| 198 | 83.0623 | 0.83 | 1879 | 358.2932 | 12.11 | 3560 | 517.3112 | 14.69 | 5241 | 651.9625 | 6.46 |
| 199 | 83.0623 | 0.84 | 1880 | 358.2953 | 14.66 | 3561 | 517.3152 | 14.75 | 5242 | 651.9636 | 4.95 |
| 200 | 83.9987 | 21.67 | 1881 | 358.2960 | 12.10 | 3562 | 517.4970 | 8.47 | 5243 | 651.9670 | 5.62 |
| 201 | 84.0448 | 0.95 | 1882 | 358.2963 | 12.19 | 3563 | 517.9939 | 0.90 | 5244 | 652.3966 | 11.72 |
| 202 | 84.0667 | 0.83 | 1883 | 358.2967 | 11.96 | 3564 | 518.2668 | 5.00 | 5245 | 652.8910 | 11.73 |
| 203 | 84.9605 | 0.65 | 1884 | 358.2992 | 12.37 | 3565 | 518.2676 | 5.05 | 5246 | 653.6972 | 11.09 |
| 204 | 84.9608 | 21.69 | 1885 | 359.0920 | 0.91 | 3566 | 518.2714 | 5.36 | 5247 | 654.0218 | 11.09 |
| 205 | 85.0294 | 1.01 | 1886 | 359.0932 | 0.91 | 3567 | 518.2717 | 4.88 | 5248 | 654.3681 | 11.09 |
| 206 | 85.9414 | 21.72 | 1887 | 359.1505 | 15.27 | 3568 | 518.2719 | 7.76 | 5249 | 654.8601 | 11.25 |
| 207 | 86.0967 | 2.01 | 1888 | 360.2085 | 7.48 | 3569 | 518.2726 | 5.53 | 5250 | 654.8605 | 11.23 |
| 208 | 86.0976 | 2.42 | 1889 | 360.2113 | 8.32 | 3570 | 518.2763 | 5.77 | 5251 | 654.8632 | 11.13 |
| 209 | 86.0976 | 2.45 | 1890 | 360.2138 | 8.49 | 3571 | 518.2765 | 6.98 | 5252 | 654.8640 | 11.30 |
| 210 | 86.0977 | 2.16 | 1891 | 360.2142 | 7.70 | 3572 | 518.2789 | 15.76 | 5253 | 654.8662 | 9.46 |
| 211 | 86.0977 | 2.26 | 1892 | 360.2185 | 7.34 | 3573 | 518.2795 | 18.93 | 5254 | 654.8676 | 9.28 |
| 212 | 86.0977 | 2.39 | 1893 | 360.7060 | 6.66 | 3574 | 518.2828 | 6.61 | 5255 | 654.8683 | 10.96 |
| 213 | 86.0978 | 2.44 | 1894 | 360.7068 | 6.98 | 3575 | 518.3031 | 20.98 | 5256 | 655.3551 | 11.23 |
| 214 | 86.0980 | 2.56 | 1895 | 360.7069 | 7.69 | 3576 | 518.3075 | 20.96 | 5257 | 655.3585 | 9.07 |
| 215 | 86.0981 | 2.53 | 1896 | 360.7070 | 7.17 | 3577 | 518.3079 | 21.05 | 5258 | 655.3585 | 9.28 |
| 216 | 86.9929 | 0.66 | 1897 | 360.7070 | 7.80 | 3578 | 518.3112 | 20.90 | 5259 | 655.3644 | 9.46 |
| 217 | 87.9363 | 21.72 | 1898 | 360.7071 | 7.82 | 3579 | 518.3114 | 20.87 | 5260 | 655.3658 | 11.28 |
| 218 | 88.0214 | 12.56 | 1899 | 360.7071 | 7.68 | 3580 | 518.3156 | 15.42 | 5261 | 655.3665 | 10.96 |
| 219 | 88.0222 | 12.40 | 1900 | 360.7073 | 7.50 | 3581 | 518.3178 | 21.25 | 5262 | 655.8711 | 11.25 |
| 220 | 88.0226 | 12.80 | 1901 | 360.7073 | 7.52 | 3582 | 518.3181 | 18.54 | 5263 | 655.9036 | 11.25 |
| 221 | 88.0227 | 12.55 | 1902 | 360.7081 | 6.82 | 3583 | 518.3184 | 15.37 | 5264 | 655.9429 | 11.22 |
| 222 | 88.0234 | 15.23 | 1903 | 360.7099 | 7.77 | 3584 | 518.3184 | 21.33 | 5265 | 656.1488 | 11.22 |
| 223 | 88.0234 | 12.38 | 1904 | 360.7104 | 7.99 | 3585 | 518.3197 | 14.51 | 5266 | 656.2772 | 12.14 |
| 224 | 90.0559 | 0.89 | 1905 | 360.7110 | 7.42 | 3586 | 518.3205 | 18.80 | 5267 | 656.3239 | 8.98 |
| 225 | 90.9763 | 0.84 | 1906 | 360.7127 | 7.49 | 3587 | 518.3206 | 20.98 | 5268 | 656.3295 | 8.77 |
| 226 | 90.9770 | 0.73 | 1907 | 361.0087 | 0.97 | 3588 | 518.3209 | 21.18 | 5269 | 656.3306 | 8.59 |
| 227 | 90.9770 | 0.80 | 1908 | 361.2052 | 7.66 | 3589 | 518.3210 | 18.84 | 5270 | 656.3397 | 10.06 |
| 228 | 95.0170 | 1.43 | 1909 | 361.2054 | 7.17 | 3590 | 518.3219 | 15.43 | 5271 | 656.3398 | 9.90 |
| 229 | 97.0113 | 1.43 | 1910 | 361.2065 | 6.82 | 3591 | 518.3219 | 15.48 | 5272 | 656.3665 | 11.23 |
| 230 | 97.0273 | 1.00 | 1911 | 361.2079 | 6.98 | 3592 | 518.3219 | 17.63 | 5273 | 656.5896 | 9.87 |
| 231 | 97.0770 | 1.06 | 1912 | 361.2084 | 7.52 | 3593 | 518.3224 | 18.51 | 5274 | 656.5986 | 10.06 |
| 232 | 98.9617 | 21.70 | 1913 | 361.2085 | 8.00 | 3594 | 518.3224 | 15.68 | 5275 | 656.6044 | 8.20 |
| 233 | 98.9846 | 2.61 | 1914 | 361.2107 | 7.69 | 3595 | 518.3228 | 18.73 | 5276 | 656.6584 | 8.77 |
| 234 | 98.9849 | 2.68 | 1915 | 361.2109 | 7.53 | 3596 | 518.3229 | 18.68 | 5277 | 656.6598 | 8.97 |
| 235 | 98.9849 | 2.75 | 1916 | 361.2720 | 21.54 | 3597 | 518.3243 | 21.02 | 5278 | 656.8426 | 9.87 |
| 236 | 98.9850 | 2.70 | 1917 | 361.7068 | 7.69 | 3598 | 518.3244 | 21.24 | 5279 | 656.9960 | 8.97 |
| 237 | 99.7702 | 6.43 | 1918 | 362.6992 | 8.02 | 3599 | 518.3248 | 20.89 | 5280 | 657.0920 | 9.87 |
| 238 | 100.0751 | 6.67 | 1919 | 362.7059 | 8.25 | 3600 | 518.3252 | 21.10 | 5281 | 657.2320 | 11.21 |
| 239 | 100.0755 | 5.72 | 1920 | 362.7060 | 8.34 | 3601 | 518.3259 | 18.72 | 5282 | 657.2335 | 15.22 |
| 240 | 100.0755 | 6.38 | 1921 | 362.7215 | 7.04 | 3602 | 518.3260 | 15.23 | 5283 | 657.2352 | 10.92 |
| 241 | 100.0755 | 5.70 | 1922 | 362.9204 | 1.04 | 3603 | 518.3269 | 15.70 | 5284 | 657.2355 | 15.26 |
| 242 | 100.0756 | 5.77 | 1923 | 362.9253 | 0.76 | 3604 | 518.3271 | 20.86 | 5285 | 657.2390 | 11.18 |
| 243 | 100.0757 | 6.11 | 1924 | 362.9261 | 0.86 | 3605 | 518.3291 | 14.66 | 5286 | 657.3423 | 8.22 |
| 244 | 100.0761 | 6.83 | 1925 | 363.0356 | 18.22 | 3606 | 518.3343 | 21.30 | 5287 | 657.3503 | 10.33 |
| 245 | 100.0761 | 6.43 | 1926 | 363.9264 | 0.79 | 3607 | 518.3372 | 21.05 | 5288 | 657.3630 | 11.58 |
| 246 | 100.0762 | 6.35 | 1927 | 364.1525 | 7.37 | 3608 | 519.1523 | 15.31 | 5289 | 657.6682 | 10.32 |
| 247 | 100.0762 | 6.95 | 1928 | 364.1542 | 7.21 | 3609 | 519.2562 | 5.00 | 5290 | 657.6840 | 9.83 |
| 248 | 100.0762 | 6.51 | 1929 | 365.1055 | 1.16 | 3610 | 519.2580 | 4.88 | 5291 | 657.6958 | 11.19 |
| 249 | 100.0763 | 6.80 | 1930 | 365.1067 | 1.11 | 3611 | 519.2684 | 7.69 | 5292 | 658.0092 | 9.82 |
| 250 | 100.0764 | 5.54 | 1931 | 365.1068 | 1.22 | 3612 | 519.2685 | 5.53 | 5293 | 658.0362 | 11.21 |
| 251 | 100.0764 | 6.27 | 1932 | 365.1071 | 1.24 | 3613 | 519.3199 | 15.26 | 5294 | 658.2376 | 11.14 |
| 252 | 100.0764 | 6.62 | 1933 | 365.1788 | 5.26 | 3614 | 519.3210 | 15.43 | 5295 | 658.3615 | 11.19 |
| 253 | 100.0764 | 6.23 | 1934 | 365.1810 | 6.22 | 3615 | 519.3239 | 15.37 | 5296 | 661.1014 | 9.48 |
| 254 | 100.0764 | 5.53 | 1935 | 365.1816 | 6.43 | 3616 | 519.3246 | 15.65 | 5297 | 661.3450 | 9.52 |
| 255 | 100.0765 | 6.64 | 1936 | 365.1816 | 6.06 | 3617 | 519.3261 | 15.70 | 5298 | 662.1059 | 9.17 |
| 256 | 100.0765 | 5.58 | 1937 | 365.1825 | 5.60 | 3618 | 519.3266 | 18.84 | 5299 | 662.3531 | 9.18 |
| 257 | 100.0766 | 6.59 | 1938 | 365.1827 | 5.58 | 3619 | 519.3267 | 15.62 | 5300 | 662.3563 | 9.17 |
| 258 | 100.0766 | 6.60 | 1939 | 365.1834 | 5.42 | 3620 | 519.3270 | 18.90 | 5301 | 662.6068 | 9.17 |
| 259 | 100.0766 | 5.14 | 1940 | 365.1838 | 5.83 | 3621 | 519.3273 | 18.56 | 5302 | 662.6117 | 9.31 |
| 260 | 100.0766 | 5.19 | 1941 | 365.1861 | 5.29 | 3622 | 519.3279 | 17.60 | 5303 | 664.3290 | 8.32 |
| 261 | 100.0769 | 6.12 | 1942 | 365.2659 | 19.46 | 3623 | 519.3280 | 18.51 | 5304 | 664.3291 | 8.47 |
| 262 | 100.1105 | 2.47 | 1943 | 365.2783 | 15.71 | 3624 | 519.3283 | 18.73 | 5305 | 664.3337 | 8.62 |
| 263 | 100.1122 | 2.25 | 1944 | 366.1850 | 6.06 | 3625 | 519.3301 | 21.35 | 5306 | 666.1324 | 8.08 |
| 264 | 100.9572 | 21.72 | 1945 | 366.1858 | 5.60 | 3626 | 519.3320 | 18.70 | 5307 | 666.1412 | 10.10 |
| 265 | 101.0774 | 5.49 | 1946 | 366.1868 | 5.44 | 3627 | 519.6398 | 8.98 | 5308 | 666.1491 | 8.31 |
| 266 | 101.0777 | 6.39 | 1947 | 366.1870 | 6.22 | 3628 | 519.8349 | 8.61 | 5309 | 666.1513 | 10.17 |
| 267 | 101.0779 | 6.64 | 1948 | 366.1872 | 5.83 | 3629 | 519.8451 | 8.95 | 5310 | 666.1514 | 10.06 |
| 268 | 101.0782 | 5.76 | 1949 | 366.2007 | 8.62 | 3630 | 520.0502 | 8.62 | 5311 | 666.1584 | 8.94 |
| 269 | 101.0791 | 6.37 | 1950 | 366.2024 | 8.41 | 3631 | 520.2986 | 18.80 | 5312 | 666.3457 | 8.28 |
| 270 | 101.0792 | 5.42 | 1951 | 366.2032 | 10.43 | 3632 | 520.2994 | 18.52 | 5313 | 666.3472 | 8.08 |
| 271 | 101.0792 | 6.49 | 1952 | 366.2033 | 10.42 | 3633 | 520.3082 | 18.74 | 5314 | 666.3479 | 8.96 |
| 272 | 101.0792 | 5.54 | 1953 | 366.2035 | 10.20 | 3634 | 520.3159 | 18.85 | 5315 | 666.3513 | 10.14 |
| 273 | 101.0793 | 6.26 | 1954 | 366.2060 | 10.21 | 3635 | 520.3188 | 18.94 | 5316 | 666.3523 | 10.19 |
| 274 | 101.0794 | 5.58 | 1955 | 366.2299 | 10.08 | 3636 | 520.3219 | 18.70 | 5317 | 666.3545 | 8.46 |
| 275 | 101.0795 | 5.20 | 1956 | 366.2382 | 9.83 | 3637 | 520.3242 | 18.82 | 5318 | 666.3633 | 10.10 |
| 276 | 101.0796 | 5.73 | 1957 | 366.2393 | 10.07 | 3638 | 520.3263 | 18.87 | 5319 | 666.5450 | 10.09 |
| 277 | 101.0797 | 6.01 | 1958 | 366.2412 | 9.99 | 3639 | 520.3275 | 18.92 | 5320 | 666.5472 | 8.54 |
| 278 | 101.0798 | 5.72 | 1959 | 367.1222 | 1.07 | 3640 | 520.3283 | 18.70 | 5321 | 666.5494 | 8.30 |
| 279 | 101.0798 | 6.12 | 1960 | 367.2053 | 10.21 | 3641 | 520.3293 | 10.93 | 5322 | 666.5511 | 8.08 |
| 280 | 101.0799 | 5.77 | 1961 | 367.2054 | 10.39 | 3642 | 520.3303 | 18.85 | 5323 | 666.5516 | 10.14 |
| 281 | 101.0799 | 5.52 | 1962 | 367.2080 | 10.43 | 3643 | 520.3326 | 8.99 | 5324 | 666.5517 | 8.97 |
| 282 | 102.0327 | 21.70 | 1963 | 367.5280 | 8.25 | 3644 | 520.3333 | 15.44 | 5325 | 666.7410 | 10.14 |
| 283 | 102.9695 | 0.68 | 1964 | 367.5314 | 8.49 | 3645 | 520.3340 | 10.88 | 5326 | 666.7452 | 8.27 |
| 284 | 103.0522 | 5.09 | 1965 | 368.1432 | 5.46 | 3646 | 520.3341 | 9.40 | 5327 | 666.7485 | 10.06 |
| 285 | 103.0534 | 5.41 | 1966 | 368.1436 | 6.07 | 3647 | 520.3342 | 9.05 | 5328 | 666.7534 | 8.30 |
| 286 | 103.0535 | 5.51 | 1967 | 368.1447 | 5.62 | 3648 | 520.3344 | 18.77 | 5329 | 666.7555 | 8.94 |
| 287 | 103.0538 | 5.90 | 1968 | 368.1450 | 5.01 | 3649 | 520.3344 | 18.72 | 5330 | 666.7583 | 8.05 |
| 288 | 103.0538 | 5.11 | 1969 | 368.1464 | 5.19 | 3650 | 520.3345 | 18.67 | 5331 | 666.8198 | 0.83 |
| 289 | 103.0542 | 6.14 | 1970 | 368.1470 | 5.55 | 3651 | 520.3346 | 18.71 | 5332 | 666.9478 | 8.44 |
| 290 | 103.0543 | 4.85 | 1971 | 368.1576 | 8.00 | 3652 | 520.3348 | 11.00 | 5333 | 666.9486 | 8.94 |
| 291 | 103.0560 | 5.72 | 1972 | 368.1614 | 7.80 | 3653 | 520.3348 | 15.13 | 5334 | 666.9496 | 10.10 |
| 292 | 104.0522 | 1.44 | 1973 | 368.1618 | 7.63 | 3654 | 520.3348 | 15.60 | 5335 | 666.9568 | 10.19 |
| 293 | 104.1069 | 0.86 | 1974 | 368.2766 | 11.73 | 3655 | 520.3352 | 18.92 | 5336 | 667.1345 | 8.97 |
| 294 | 105.1105 | 0.86 | 1975 | 368.2796 | 14.36 | 3656 | 520.3354 | 15.49 | 5337 | 667.1535 | 10.19 |
| 295 | 106.9507 | 0.81 | 1976 | 368.5315 | 5.73 | 3657 | 520.3369 | 8.85 | 5338 | 667.3566 | 10.14 |
| 296 | 107.0861 | 1.04 | 1977 | 368.5353 | 5.98 | 3658 | 520.3369 | 18.01 | 5339 | 667.7896 | 8.89 |
| 297 | 107.0861 | 0.07 | 1978 | 368.5359 | 5.33 | 3659 | 520.3372 | 9.37 | 5340 | 667.7962 | 8.66 |
| 298 | 110.0093 | 0.63 | 1979 | 368.5360 | 5.57 | 3660 | 520.3374 | 18.26 | 5341 | 667.7990 | 8.86 |
| 299 | 110.0601 | 7.23 | 1980 | 368.5361 | 5.68 | 3661 | 520.3374 | 15.02 | 5342 | 668.3038 | 8.91 |
| 300 | 110.9751 | 1.82 | 1981 | 368.5365 | 5.03 | 3662 | 520.3377 | 18.74 | 5343 | 668.3106 | 8.65 |
| 301 | 111.0100 | 0.68 | 1982 | 368.5378 | 5.48 | 3663 | 520.3378 | 8.98 | 5344 | 668.7986 | 8.92 |
| 302 | 111.0929 | 1.77 | 1983 | 368.5378 | 4.91 | 3664 | 520.3378 | 15.29 | 5345 | 668.8675 | 0.75 |
| 303 | 111.1164 | 17.73 | 1984 | 368.8663 | 5.33 | 3665 | 520.3378 | 9.17 | 5346 | 668.8775 | 0.82 |
| 304 | 111.1165 | 17.76 | 1985 | 368.8682 | 5.48 | 3666 | 520.3383 | 15.18 | 5347 | 669.3720 | 0.86 |
| 305 | 111.1166 | 10.11 | 1986 | 368.8684 | 5.98 | 3667 | 520.3388 | 18.81 | 5348 | 672.9808 | 9.74 |
| 306 | 111.1176 | 17.84 | 1987 | 368.8692 | 5.57 | 3668 | 520.3390 | 15.35 | 5349 | 673.5402 | 6.49 |
| 307 | 111.1193 | 17.63 | 1988 | 368.8746 | 5.68 | 3669 | 520.3397 | 15.42 | 5350 | 673.8002 | 6.49 |
| 308 | 112.1132 | 2.53 | 1989 | 369.0738 | 16.23 | 3670 | 520.3398 | 15.07 | 5351 | 674.0528 | 6.49 |
| 309 | 112.9767 | 21.70 | 1990 | 369.1647 | 8.02 | 3671 | 520.3400 | 14.98 | 5352 | 675.8165 | 9.41 |
| 310 | 113.9460 | 21.68 | 1991 | 369.1659 | 7.79 | 3672 | 520.3401 | 18.24 | 5353 | 676.3199 | 9.44 |
| 311 | 113.9622 | 21.89 | 1992 | 369.1992 | 21.34 | 3673 | 520.3402 | 18.21 | 5354 | 676.8661 | 0.77 |
| 312 | 113.9636 | 22.14 | 1993 | 369.3524 | 19.45 | 3674 | 520.3405 | 14.80 | 5355 | 680.8561 | 0.69 |
| 313 | 113.9894 | 1.43 | 1994 | 369.3546 | 0.47 | 3675 | 520.3405 | 18.22 | 5356 | 681.0119 | 10.56 |
| 314 | 114.0646 | 0.88 | 1995 | 370.0614 | 0.90 | 3676 | 520.3408 | 18.77 | 5357 | 681.3579 | 10.56 |
| 315 | 114.1273 | 8.71 | 1996 | 370.2473 | 21.37 | 3677 | 520.3412 | 18.75 | 5358 | 681.7137 | 11.16 |
| 316 | 114.9550 | 21.65 | 1997 | 370.2915 | 12.27 | 3678 | 520.3414 | 15.16 | 5359 | 682.0524 | 11.14 |
| 317 | 115.9632 | 21.72 | 1998 | 370.2982 | 12.11 | 3679 | 520.3420 | 18.17 | 5360 | 684.3083 | 10.91 |
| 318 | 116.0702 | 1.07 | 1999 | 370.2985 | 14.79 | 3680 | 520.3421 | 18.11 | 5361 | 684.8499 | 0.81 |
| 319 | 116.0715 | 1.05 | 2000 | 370.7177 | 10.92 | 3681 | 520.3423 | 18.16 | 5362 | 689.1057 | 8.39 |
| 320 | 116.9707 | 21.69 | 2001 | 371.0954 | 19.04 | 3682 | 520.3427 | 14.90 | 5363 | 689.1115 | 10.03 |
| 321 | 117.0751 | 1.05 | 2002 | 371.1006 | 19.72 | 3683 | 520.3431 | 18.13 | 5364 | 689.1124 | 8.96 |
| 322 | 117.9587 | 21.69 | 2003 | 371.1007 | 18.52 | 3684 | 520.3434 | 14.94 | 5365 | 689.1155 | 9.87 |
| 323 | 118.0860 | 1.25 | 2004 | 371.1010 | 17.20 | 3685 | 520.3434 | 18.20 | 5366 | 689.1163 | 8.18 |
| 324 | 118.0861 | 1.27 | 2005 | 371.1010 | 17.43 | 3686 | 520.3462 | 18.14 | 5367 | 689.1182 | 8.69 |
| 325 | 118.0863 | 0.95 | 2006 | 371.1011 | 18.38 | 3687 | 521.2534 | 6.98 | 5368 | 689.1189 | 10.03 |
| 326 | 118.0866 | 1.20 | 2007 | 371.1011 | 17.38 | 3688 | 521.3336 | 10.93 | 5369 | 689.1201 | 10.12 |
| 327 | 119.0900 | 0.96 | 2008 | 371.1027 | 19.64 | 3689 | 521.3356 | 15.00 | 5370 | 689.1201 | 8.20 |
| 328 | 120.0024 | 0.90 | 2009 | 371.1032 | 19.43 | 3690 | 521.3399 | 18.17 | 5371 | 689.1217 | 9.81 |
| 329 | 120.0670 | 0.92 | 2010 | 371.1035 | 19.04 | 3691 | 521.3408 | 18.14 | 5372 | 689.1226 | 8.04 |
| 330 | 120.0792 | 6.65 | 2011 | 371.1037 | 1.00 | 3692 | 521.3412 | 18.01 | 5373 | 689.3257 | 10.62 |
| 331 | 120.0799 | 5.13 | 2012 | 371.1040 | 17.25 | 3693 | 521.3424 | 15.16 | 5374 | 689.3661 | 9.81 |
| 332 | 120.0801 | 6.22 | 2013 | 371.1063 | 17.34 | 3694 | 521.3439 | 14.95 | 5375 | 689.3663 | 8.69 |
| 333 | 120.0801 | 6.24 | 2014 | 371.2270 | 8.42 | 3695 | 521.3445 | 18.20 | 5376 | 689.3668 | 10.17 |
| 334 | 120.0802 | 4.51 | 2015 | 371.2284 | 10.01 | 3696 | 521.3449 | 18.17 | 5377 | 689.3705 | 9.90 |
| 335 | 120.0803 | 5.95 | 2016 | 371.2287 | 9.74 | 3697 | 521.3450 | 18.23 | 5378 | 689.3718 | 8.18 |
| 336 | 120.0803 | 4.86 | 2017 | 371.2288 | 9.72 | 3698 | 521.3452 | 18.13 | 5379 | 689.3723 | 8.39 |
| 337 | 120.0803 | 4.03 | 2018 | 371.2295 | 8.12 | 3699 | 521.3453 | 15.29 | 5380 | 689.3744 | 8.04 |
| 338 | 120.0803 | 4.88 | 2019 | 371.2413 | 21.37 | 3700 | 521.3466 | 14.84 | 5381 | 689.3788 | 8.94 |
| 339 | 120.0804 | 5.98 | 2020 | 371.2423 | 18.16 | 3701 | 521.3470 | 14.90 | 5382 | 689.3794 | 8.20 |
| 340 | 120.0804 | 8.29 | 2021 | 371.7242 | 7.06 | 3702 | 521.3481 | 14.80 | 5383 | 689.6151 | 8.04 |
| 341 | 120.0805 | 6.14 | 2022 | 372.0674 | 0.87 | 3703 | 521.3484 | 18.24 | 5384 | 689.6180 | 9.81 |
| 342 | 120.0805 | 6.20 | 2023 | 372.1851 | 9.83 | 3704 | 521.3487 | 15.19 | 5385 | 689.6194 | 8.18 |
| 343 | 120.0806 | 6.15 | 2024 | 372.2553 | 8.09 | 3705 | 521.3488 | 15.13 | 5386 | 689.6214 | 8.70 |
| 344 | 120.0806 | 4.99 | 2025 | 372.2579 | 7.97 | 3706 | 521.5054 | 6.95 | 5387 | 689.6228 | 9.87 |
| 345 | 120.0807 | 6.48 | 2026 | 372.2593 | 9.17 | 3707 | 522.2664 | 8.53 | 5388 | 689.6236 | 8.16 |
| 346 | 120.0807 | 5.72 | 2027 | 372.2598 | 9.11 | 3708 | 522.2689 | 8.35 | 5389 | 689.6242 | 8.39 |
| 347 | 120.0807 | 6.31 | 2028 | 372.3449 | 15.85 | 3709 | 522.2707 | 8.17 | 5390 | 689.6265 | 10.11 |
| 348 | 120.0808 | 6.66 | 2029 | 373.1224 | 9.18 | 3710 | 522.2734 | 8.26 | 5391 | 689.6269 | 8.94 |
| 349 | 120.0808 | 5.43 | 2030 | 373.1236 | 10.52 | 3711 | 522.2754 | 7.62 | 5392 | 689.6271 | 8.20 |
| 350 | 120.0809 | 5.40 | 2031 | 373.1645 | 6.89 | 3712 | 522.2757 | 7.20 | 5393 | 689.8301 | 10.53 |
| 351 | 120.0809 | 5.07 | 2032 | 373.2073 | 17.88 | 3713 | 522.2774 | 8.41 | 5394 | 689.8536 | 8.41 |
| 352 | 120.0810 | 6.92 | 2033 | 373.2169 | 15.25 | 3714 | 522.2788 | 7.44 | 5395 | 689.8651 | 8.05 |
| 353 | 120.0811 | 7.08 | 2034 | 373.2730 | 16.38 | 3715 | 522.3437 | 15.02 | 5396 | 689.8662 | 8.72 |
| 354 | 120.0812 | 4.13 | 2035 | 374.0478 | 0.86 | 3716 | 522.3464 | 15.62 | 5397 | 689.8673 | 9.87 |
| 355 | 120.0812 | 5.51 | 2036 | 374.1132 | 14.13 | 3717 | 522.3472 | 18.24 | 5398 | 689.8706 | 10.09 |
| 356 | 120.0812 | 5.57 | 2037 | 374.1959 | 12.25 | 3718 | 522.3474 | 15.29 | 5399 | 689.8714 | 8.94 |
| 357 | 120.0813 | 8.42 | 2038 | 374.2774 | 16.40 | 3719 | 522.3488 | 15.14 | 5400 | 689.8718 | 8.16 |
| 358 | 120.0815 | 5.11 | 2039 | 375.1945 | 10.04 | 3720 | 522.3497 | 18.95 | 5401 | 689.8739 | 9.83 |
| 359 | 120.0816 | 5.90 | 2040 | 375.1945 | 10.01 | 3721 | 522.3508 | 15.19 | 5402 | 690.0712 | 10.55 |
| 360 | 120.0816 | 6.82 | 2041 | 375.6602 | 10.28 | 3722 | 522.3522 | 15.71 | 5403 | 690.1044 | 9.87 |
| 361 | 120.0817 | 4.82 | 2042 | 376.1896 | 10.04 | 3723 | 522.3528 | 15.82 | 5404 | 690.1110 | 9.83 |
| 362 | 120.0820 | 4.66 | 2043 | 376.1909 | 10.17 | 3724 | 522.3534 | 21.37 | 5405 | 690.1159 | 8.94 |
| 363 | 121.0823 | 5.95 | 2044 | 377.2481 | 21.60 | 3725 | 522.3558 | 15.80 | 5406 | 690.1219 | 8.69 |
| 364 | 121.0825 | 6.14 | 2045 | 378.2350 | 10.84 | 3726 | 522.3560 | 15.89 | 5407 | 690.1262 | 9.90 |
| 365 | 121.0836 | 5.13 | 2046 | 378.2377 | 8.48 | 3727 | 522.3562 | 15.90 | 5408 | 690.1275 | 8.18 |
| 366 | 121.0836 | 5.90 | 2047 | 378.8988 | 0.76 | 3728 | 522.3564 | 15.83 | 5409 | 690.1301 | 8.04 |
| 367 | 121.0839 | 4.86 | 2048 | 378.8996 | 0.84 | 3729 | 522.3565 | 19.16 | 5410 | 690.3306 | 10.55 |
| 368 | 121.0842 | 4.64 | 2049 | 379.6824 | 7.69 | 3730 | 522.3569 | 21.34 | 5411 | 690.3675 | 9.87 |
| 369 | 121.0842 | 5.74 | 2050 | 379.7095 | 6.06 | 3731 | 522.3571 | 16.00 | 5412 | 690.3681 | 8.96 |
| 370 | 121.0843 | 5.72 | 2051 | 379.7122 | 5.96 | 3732 | 522.3571 | 19.28 | 5413 | 690.3683 | 8.18 |
| 371 | 121.0845 | 5.44 | 2052 | 379.7144 | 5.64 | 3733 | 522.3573 | 16.06 | 5414 | 690.3688 | 10.16 |
| 372 | 121.0845 | 5.38 | 2053 | 379.7146 | 5.84 | 3734 | 522.3574 | 16.05 | 5415 | 690.5789 | 10.55 |
| 373 | 121.0845 | 5.40 | 2054 | 379.7176 | 5.93 | 3735 | 522.3588 | 15.74 | 5416 | 690.6336 | 9.91 |
| 374 | 121.0848 | 5.51 | 2055 | 379.7178 | 5.95 | 3736 | 522.3590 | 15.70 | 5417 | 690.8251 | 0.71 |
| 375 | 121.0851 | 5.11 | 2056 | 380.2180 | 5.93 | 3737 | 522.3594 | 19.15 | 5418 | 691.3680 | 11.03 |
| 376 | 122.0548 | 5.67 | 2057 | 380.2477 | 14.34 | 3738 | 522.7789 | 7.60 | 5419 | 691.3692 | 10.84 |
| 377 | 122.0564 | 5.59 | 2058 | 380.2527 | 14.49 | 3739 | 522.7790 | 8.53 | 5420 | 694.8692 | 9.94 |
| 378 | 122.0565 | 6.12 | 2059 | 380.2529 | 14.66 | 3740 | 522.7803 | 8.41 | 5421 | 696.8477 | 0.74 |
| 379 | 122.0568 | 6.56 | 2060 | 380.2551 | 14.51 | 3741 | 523.3435 | 18.27 | 5422 | 698.6019 | 10.11 |
| 380 | 122.0568 | 5.24 | 2061 | 380.2576 | 14.48 | 3742 | 523.3500 | 19.04 | 5423 | 698.6184 | 9.84 |
| 381 | 122.0573 | 5.98 | 2062 | 380.2607 | 17.70 | 3743 | 523.3504 | 15.15 | 5424 | 698.8379 | 10.01 |
| 382 | 122.0573 | 5.87 | 2063 | 380.3034 | 21.54 | 3744 | 523.3540 | 16.05 | 5425 | 698.8548 | 7.27 |
| 383 | 122.0578 | 6.41 | 2064 | 380.7301 | 6.58 | 3745 | 523.3590 | 15.89 | 5426 | 698.8577 | 9.87 |
| 384 | 122.0580 | 6.15 | 2065 | 380.7304 | 6.90 | 3746 | 523.3591 | 15.75 | 5427 | 698.8645 | 8.85 |
| 385 | 122.0580 | 6.19 | 2066 | 380.7313 | 7.10 | 3747 | 523.3596 | 19.34 | 5428 | 698.8840 | 12.24 |
| 386 | 122.0581 | 5.92 | 2067 | 380.7324 | 6.72 | 3748 | 523.3599 | 16.07 | 5429 | 699.1033 | 9.90 |
| 387 | 122.0597 | 5.50 | 2068 | 380.7326 | 7.27 | 3749 | 523.3601 | 16.00 | 5430 | 699.1044 | 10.11 |
| 388 | 122.9258 | 0.81 | 2069 | 380.7330 | 7.08 | 3750 | 523.3626 | 15.83 | 5431 | 699.1307 | 12.19 |
| 389 | 122.9628 | 21.76 | 2070 | 380.7331 | 7.15 | 3751 | 523.3657 | 19.15 | 5432 | 699.3607 | 9.89 |
| 390 | 123.0426 | 9.77 | 2071 | 380.7332 | 6.70 | 3752 | 523.3663 | 18.97 | 5433 | 699.3806 | 12.26 |
| 391 | 123.0432 | 2.46 | 2072 | 380.7333 | 6.94 | 3753 | 523.3665 | 15.59 | 5434 | 700.3877 | 9.41 |
| 392 | 123.0434 | 2.61 | 2073 | 380.7335 | 6.54 | 3754 | 524.2762 | 18.32 | 5435 | 700.3881 | 11.23 |
| 393 | 123.0437 | 2.27 | 2074 | 380.7338 | 7.13 | 3755 | 524.2946 | 18.87 | 5436 | 700.3882 | 9.08 |
| 394 | 124.0821 | 19.12 | 2075 | 380.7344 | 6.84 | 3756 | 524.2962 | 15.67 | 5437 | 700.3913 | 11.20 |
| 395 | 124.0837 | 17.37 | 2076 | 380.7349 | 6.43 | 3757 | 524.2966 | 15.64 | 5438 | 700.3921 | 10.93 |
| 396 | 124.0837 | 19.18 | 2077 | 380.7356 | 6.88 | 3758 | 524.3058 | 15.76 | 5439 | 700.3921 | 9.70 |
| 397 | 124.0850 | 5.67 | 2078 | 380.7358 | 7.04 | 3759 | 524.3091 | 15.49 | 5440 | 700.3951 | 11.25 |
| 398 | 124.0850 | 6.36 | 2079 | 380.7359 | 7.03 | 3760 | 524.3133 | 15.75 | 5441 | 700.7131 | 10.93 |
| 399 | 124.0851 | 19.07 | 2080 | 380.7388 | 6.59 | 3761 | 524.3158 | 15.74 | 5442 | 700.7162 | 11.25 |
| 400 | 124.0855 | 15.68 | 2081 | 381.1246 | 15.24 | 3762 | 524.3546 | 16.06 | 5443 | 700.7204 | 11.23 |
| 401 | 124.0866 | 5.40 | 2082 | 381.1306 | 12.46 | 3763 | 524.3601 | 15.92 | 5444 | 700.7208 | 11.19 |
| 402 | 124.0866 | 15.77 | 2083 | 381.1351 | 12.62 | 3764 | 524.3637 | 19.29 | 5445 | 700.7233 | 9.43 |
| 403 | 124.0866 | 5.93 | 2084 | 381.1801 | 5.43 | 3765 | 524.3663 | 17.08 | 5446 | 700.7238 | 9.10 |
| 404 | 124.0866 | 21.16 | 2085 | 381.2001 | 6.07 | 3766 | 524.3668 | 19.34 | 5447 | 700.7274 | 9.41 |
| 405 | 124.0866 | 17.16 | 2086 | 381.2010 | 5.80 | 3767 | 524.3682 | 17.17 | 5448 | 700.7281 | 9.70 |
| 406 | 124.0868 | 15.93 | 2087 | 381.2036 | 6.36 | 3768 | 524.3692 | 16.71 | 5449 | 700.7309 | 9.86 |
| 407 | 124.0868 | 19.15 | 2088 | 381.2051 | 6.62 | 3769 | 524.3692 | 16.63 | 5450 | 700.7378 | 9.20 |
| 408 | 124.0868 | 19.02 | 2089 | 381.2076 | 6.16 | 3770 | 524.3704 | 16.91 | 5451 | 701.0422 | 9.08 |
| 409 | 124.0868 | 18.38 | 2090 | 381.2087 | 6.38 | 3771 | 524.3708 | 17.18 | 5452 | 701.0521 | 11.20 |
| 410 | 124.0868 | 18.42 | 2091 | 381.2093 | 6.26 | 3772 | 524.3709 | 17.13 | 5453 | 701.0527 | 11.23 |
| 411 | 124.0869 | 19.72 | 2092 | 381.2129 | 6.52 | 3773 | 524.3712 | 16.81 | 5454 | 701.0556 | 9.43 |
| 412 | 124.0869 | 18.95 | 2093 | 381.2334 | 6.72 | 3774 | 524.3726 | 16.94 | 5455 | 701.0567 | 10.93 |
| 413 | 124.0869 | 18.18 | 2094 | 381.2336 | 6.71 | 3775 | 524.3730 | 21.33 | 5456 | 701.0636 | 9.10 |
| 414 | 124.0869 | 5.81 | 2095 | 381.2339 | 6.88 | 3776 | 524.3731 | 16.99 | 5457 | 701.3888 | 11.23 |
| 415 | 124.0869 | 15.65 | 2096 | 381.2341 | 7.04 | 3777 | 524.3739 | 16.92 | 5458 | 701.3968 | 10.96 |
| 416 | 124.0869 | 17.88 | 2097 | 381.2344 | 6.94 | 3778 | 524.3743 | 17.24 | 5459 | 702.3627 | 9.90 |
| 417 | 124.0870 | 6.14 | 2098 | 381.2348 | 6.92 | 3779 | 524.3754 | 20.88 | 5460 | 702.3853 | 8.90 |
| 418 | 124.0870 | 17.42 | 2099 | 381.2365 | 7.29 | 3780 | 524.3756 | 20.49 | 5461 | 702.3910 | 9.40 |
| 419 | 124.0871 | 19.21 | 2100 | 381.2366 | 7.06 | 3781 | 524.3761 | 17.05 | 5462 | 702.5882 | 8.20 |
| 420 | 124.0871 | 6.15 | 2101 | 381.2596 | 14.54 | 3782 | 524.3788 | 20.50 | 5463 | 702.5917 | 8.94 |
| 421 | 124.0883 | 18.25 | 2102 | 381.7022 | 6.36 | 3783 | 524.7970 | 15.52 | 5464 | 702.5953 | 10.14 |
| 422 | 124.0883 | 19.09 | 2103 | 381.7032 | 6.52 | 3784 | 524.8004 | 15.60 | 5465 | 702.6062 | 9.87 |
| 423 | 124.0884 | 19.14 | 2104 | 381.7320 | 6.72 | 3785 | 524.8032 | 15.76 | 5466 | 702.8326 | 9.93 |
| 424 | 124.0888 | 19.03 | 2105 | 381.7325 | 7.06 | 3786 | 524.8038 | 15.64 | 5467 | 702.8514 | 9.99 |
| 425 | 124.0900 | 19.27 | 2106 | 381.7330 | 6.94 | 3787 | 524.8060 | 18.86 | 5468 | 702.8631 | 1.02 |
| 426 | 124.0900 | 18.95 | 2107 | 381.7340 | 6.84 | 3788 | 525.2610 | 8.04 | 5469 | 702.8638 | 0.78 |
| 427 | 124.0915 | 18.39 | 2108 | 381.7355 | 7.04 | 3789 | 525.2685 | 9.87 | 5470 | 702.8649 | 8.96 |
| 428 | 124.0929 | 17.30 | 2109 | 381.7383 | 7.03 | 3790 | 525.2762 | 10.04 | 5471 | 702.8670 | 0.76 |
| 429 | 124.9633 | 21.67 | 2110 | 382.0562 | 1.19 | 3791 | 525.2815 | 8.17 | 5472 | 702.8685 | 10.11 |
| 430 | 125.9854 | 21.70 | 2111 | 382.1605 | 6.67 | 3792 | 525.2824 | 10.20 | 5473 | 703.1007 | 8.18 |
| 431 | 125.9854 | 0.68 | 2112 | 382.1606 | 7.03 | 3793 | 525.2845 | 9.05 | 5474 | 703.1067 | 9.90 |
| 432 | 125.9857 | 21.75 | 2113 | 382.1606 | 7.37 | 3794 | 525.2871 | 8.94 | 5475 | 703.3538 | 0.78 |
| 433 | 126.0218 | 0.99 | 2114 | 382.1608 | 7.39 | 3795 | 525.2875 | 9.37 | 5476 | 703.5757 | 0.47 |
| 434 | 126.9596 | 21.72 | 2115 | 382.1617 | 7.20 | 3796 | 525.2912 | 9.16 | 5477 | 703.5757 | 21.31 |
| 435 | 127.0231 | 0.68 | 2116 | 382.1618 | 7.13 | 3797 | 525.2945 | 15.76 | 5478 | 703.8550 | 0.81 |
| 436 | 127.0362 | 0.97 | 2117 | 382.1621 | 7.19 | 3798 | 525.2951 | 10.93 | 5479 | 704.7056 | 10.66 |
| 437 | 128.0265 | 0.68 | 2118 | 382.1626 | 6.92 | 3799 | 525.2966 | 15.53 | 5480 | 704.8601 | 0.79 |
| 438 | 128.9632 | 21.70 | 2119 | 382.1627 | 7.45 | 3800 | 525.2978 | 18.95 | 5481 | 704.8763 | 0.84 |
| 439 | 129.0390 | 0.62 | 2120 | 382.1629 | 7.46 | 3801 | 525.3013 | 18.90 | 5482 | 705.0351 | 10.66 |
| 440 | 129.0635 | 0.96 | 2121 | 382.1633 | 7.03 | 3802 | 525.3045 | 15.63 | 5483 | 705.3760 | 10.66 |
| 441 | 129.1276 | 17.60 | 2122 | 382.1639 | 7.14 | 3803 | 525.3070 | 15.64 | 5484 | 706.0135 | 9.59 |
| 442 | 130.0391 | 0.62 | 2123 | 382.1656 | 6.89 | 3804 | 525.3710 | 19.29 | 5485 | 706.0265 | 11.48 |
| 443 | 130.0480 | 3.29 | 2124 | 382.1728 | 7.20 | 3805 | 525.3711 | 16.94 | 5486 | 706.3528 | 11.49 |
| 444 | 130.0485 | 0.83 | 2125 | 382.1952 | 7.97 | 3806 | 525.3728 | 17.24 | 5487 | 706.3545 | 9.59 |
| 445 | 130.0487 | 0.81 | 2126 | 382.1954 | 7.88 | 3807 | 525.3741 | 16.71 | 5488 | 706.6825 | 11.48 |
| 446 | 130.0491 | 0.86 | 2127 | 382.1958 | 7.98 | 3808 | 525.3744 | 17.06 | 5489 | 706.6882 | 9.59 |
| 447 | 130.0498 | 2.64 | 2128 | 382.1966 | 8.11 | 3809 | 525.3744 | 20.91 | 5490 | 710.8394 | 0.81 |
| 448 | 130.0498 | 2.69 | 2129 | 382.1978 | 8.00 | 3810 | 525.3745 | 17.08 | 5491 | 710.8414 | 0.77 |
| 449 | 130.0498 | 2.63 | 2130 | 382.1981 | 7.71 | 3811 | 525.3748 | 16.99 | 5492 | 712.3271 | 7.15 |
| 450 | 130.0499 | 0.95 | 2131 | 382.1990 | 8.68 | 3812 | 525.3757 | 17.18 | 5493 | 712.3311 | 8.57 |
| 451 | 130.0507 | 2.50 | 2132 | 382.1995 | 8.10 | 3813 | 525.3759 | 17.23 | 5494 | 712.3348 | 7.41 |
| 452 | 130.0507 | 3.09 | 2133 | 382.3183 | 18.18 | 3814 | 525.3761 | 16.81 | 5495 | 712.3413 | 8.52 |
| 453 | 130.0526 | 1.10 | 2134 | 383.1150 | 0.98 | 3815 | 525.3777 | 17.03 | 5496 | 712.3636 | 10.28 |
| 454 | 130.0649 | 14.03 | 2135 | 383.1167 | 1.01 | 3816 | 525.3778 | 17.05 | 5497 | 712.3671 | 10.29 |
| 455 | 130.0849 | 1.57 | 2136 | 383.1175 | 1.39 | 3817 | 525.3806 | 20.50 | 5498 | 712.3708 | 10.31 |
| 456 | 130.0851 | 1.37 | 2137 | 383.1631 | 7.11 | 3818 | 525.3818 | 16.91 | 5499 | 712.3735 | 10.45 |
| 457 | 130.0866 | 1.54 | 2138 | 383.1634 | 7.23 | 3819 | 525.3824 | 17.15 | 5500 | 712.5306 | 8.57 |
| 458 | 130.1077 | 0.91 | 2139 | 383.1647 | 6.91 | 3820 | 525.4689 | 9.87 | 5501 | 712.5310 | 7.62 |
| 459 | 130.1094 | 0.85 | 2140 | 383.1649 | 7.37 | 3821 | 525.4746 | 8.94 | 5502 | 712.5313 | 8.39 |
| 460 | 131.0375 | 0.67 | 2141 | 383.1660 | 7.20 | 3822 | 525.4755 | 10.05 | 5503 | 712.5318 | 8.50 |
| 461 | 131.0480 | 5.72 | 2142 | 383.2022 | 7.99 | 3823 | 525.4815 | 8.05 | 5504 | 712.5366 | 7.24 |
| 462 | 131.0503 | 5.51 | 2143 | 383.2071 | 19.60 | 3824 | 525.4819 | 8.17 | 5505 | 712.5371 | 8.53 |
| 463 | 132.0767 | 0.96 | 2144 | 383.9810 | 8.01 | 3825 | 525.5396 | 9.41 | 5506 | 712.5417 | 7.15 |
| 464 | 132.0784 | 1.01 | 2145 | 383.9847 | 8.34 | 3826 | 525.5404 | 11.20 | 5507 | 712.5425 | 7.42 |
| 465 | 132.0994 | 1.35 | 2146 | 384.1166 | 1.00 | 3827 | 525.5423 | 9.68 | 5508 | 712.5426 | 8.50 |
| 466 | 132.1002 | 7.76 | 2147 | 384.1883 | 8.80 | 3828 | 525.5424 | 9.43 | 5509 | 712.7213 | 8.40 |
| 467 | 132.1007 | 9.87 | 2148 | 384.1915 | 8.63 | 3829 | 525.5428 | 11.22 | 5510 | 712.7343 | 7.62 |
| 468 | 132.1013 | 2.38 | 2149 | 384.2326 | 8.01 | 3830 | 525.5453 | 11.36 | 5511 | 712.7346 | 7.42 |
| 469 | 132.1014 | 2.42 | 2150 | 384.2390 | 8.34 | 3831 | 525.5457 | 9.08 | 5512 | 712.7377 | 8.57 |
| 470 | 132.1014 | 2.45 | 2151 | 384.4824 | 8.34 | 3832 | 525.5482 | 10.93 | 5513 | 712.7387 | 7.17 |
| 471 | 132.1014 | 2.16 | 2152 | 385.1123 | 1.00 | 3833 | 525.5486 | 9.20 | 5514 | 712.7389 | 8.50 |
| 472 | 132.1014 | 8.52 | 2153 | 385.1136 | 0.98 | 3834 | 525.6755 | 10.11 | 5515 | 712.7414 | 7.14 |
| 473 | 132.1015 | 2.39 | 2154 | 385.2125 | 9.96 | 3835 | 525.6756 | 8.18 | 5516 | 712.8288 | 0.75 |
| 474 | 132.1015 | 2.14 | 2155 | 386.0402 | 0.90 | 3836 | 525.6758 | 9.87 | 5517 | 712.9321 | 8.55 |
| 475 | 132.1017 | 2.56 | 2156 | 387.2347 | 15.72 | 3837 | 525.6820 | 8.96 | 5518 | 712.9408 | 7.15 |
| 476 | 132.1019 | 2.11 | 2157 | 387.2687 | 18.13 | 3838 | 525.6825 | 10.01 | 5519 | 712.9417 | 8.50 |
| 477 | 132.1022 | 2.03 | 2158 | 387.2710 | 18.20 | 3839 | 525.6855 | 9.87 | 5520 | 712.9448 | 7.41 |
| 478 | 132.1031 | 2.44 | 2159 | 387.2714 | 14.93 | 3840 | 525.7881 | 9.21 | 5521 | 713.3690 | 10.28 |
| 479 | 132.1032 | 9.91 | 2160 | 387.2717 | 14.72 | 3841 | 525.7882 | 9.08 | 5522 | 713.3763 | 10.42 |
| 480 | 133.0302 | 1.44 | 2161 | 387.2723 | 3.78 | 3842 | 525.7910 | 11.36 | 5523 | 715.3317 | 9.48 |
| 481 | 133.0307 | 1.57 | 2162 | 387.2736 | 2.44 | 3843 | 525.7913 | 9.68 | 5524 | 715.5015 | 9.48 |
| 482 | 133.0319 | 1.45 | 2163 | 387.2757 | 18.19 | 3844 | 525.7918 | 9.41 | 5525 | 715.6864 | 9.48 |
| 483 | 134.0201 | 0.89 | 2164 | 387.2763 | 7.07 | 3845 | 525.7929 | 11.25 | 5526 | 715.8905 | 11.05 |
| 484 | 134.0375 | 1.44 | 2165 | 388.2487 | 10.01 | 3846 | 525.7940 | 10.93 | 5527 | 718.8357 | 0.79 |
| 485 | 134.0476 | 1.05 | 2166 | 388.2527 | 9.44 | 3847 | 525.7957 | 9.67 | 5528 | 719.8351 | 0.79 |
| 486 | 135.0021 | 0.96 | 2167 | 388.2528 | 9.65 | 3848 | 525.7983 | 9.40 | 5529 | 719.8491 | 8.20 |
| 487 | 136.0464 | 0.85 | 2168 | 388.2532 | 8.43 | 3849 | 525.7991 | 11.20 | 5530 | 719.8596 | 8.38 |
| 488 | 136.0466 | 0.84 | 2169 | 388.2545 | 9.74 | 3850 | 525.8043 | 9.43 | 5531 | 720.4032 | 7.68 |
| 489 | 136.0743 | 8.67 | 2170 | 388.2554 | 8.73 | 3851 | 525.8068 | 18.89 | 5532 | 720.4041 | 7.52 |
| 490 | 136.0748 | 2.47 | 2171 | 388.2561 | 8.33 | 3852 | 525.8080 | 15.52 | 5533 | 720.4045 | 7.99 |
| 491 | 136.0749 | 2.16 | 2172 | 388.2587 | 8.57 | 3853 | 525.8118 | 15.67 | 5534 | 720.4056 | 6.66 |
| 492 | 136.0750 | 2.46 | 2173 | 388.2604 | 8.15 | 3854 | 525.8761 | 8.18 | 5535 | 720.4062 | 7.17 |
| 493 | 136.0752 | 2.24 | 2174 | 389.1630 | 13.67 | 3855 | 525.8762 | 9.89 | 5536 | 720.4065 | 7.69 |
| 494 | 136.0753 | 2.61 | 2175 | 389.2400 | 8.75 | 3856 | 525.8786 | 10.06 | 5537 | 720.4073 | 7.64 |
| 495 | 136.0769 | 2.26 | 2176 | 389.2612 | 9.74 | 3857 | 526.0424 | 11.23 | 5538 | 720.4085 | 6.82 |
| 496 | 136.9312 | 2.63 | 2177 | 391.9494 | 0.84 | 3858 | 526.0463 | 10.93 | 5539 | 720.4087 | 6.98 |
| 497 | 137.0448 | 5.02 | 2178 | 392.2269 | 21.33 | 3859 | 526.0466 | 9.10 | 5540 | 721.4063 | 7.69 |
| 498 | 137.0455 | 2.06 | 2179 | 393.0777 | 0.74 | 3860 | 526.0468 | 9.68 | 5541 | 721.4160 | 6.98 |
| 499 | 137.0455 | 2.04 | 2180 | 393.1305 | 0.95 | 3861 | 526.0474 | 9.41 | 5542 | 722.4143 | 7.69 |
| 500 | 137.0456 | 5.72 | 2181 | 393.2025 | 10.04 | 3862 | 526.0482 | 11.20 | 5543 | 726.8353 | 11.15 |
| 501 | 137.0456 | 2.46 | 2182 | 393.2043 | 8.42 | 3863 | 526.0484 | 11.25 | 5544 | 727.3617 | 8.66 |
| 502 | 137.0456 | 2.47 | 2183 | 393.2084 | 9.63 | 3864 | 526.0501 | 9.43 | 5545 | 727.3685 | 10.60 |
| 503 | 137.0456 | 2.42 | 2184 | 393.2103 | 9.67 | 3865 | 526.0767 | 9.89 | 5546 | 727.3736 | 8.51 |
| 504 | 137.0457 | 5.37 | 2185 | 393.2111 | 8.20 | 3866 | 526.2844 | 15.30 | 5547 | 728.3705 | 8.51 |
| 505 | 137.0458 | 5.78 | 2186 | 393.2134 | 9.68 | 3867 | 526.2864 | 14.98 | 5548 | 728.3854 | 9.67 |
| 506 | 137.0458 | 5.40 | 2187 | 393.2153 | 9.44 | 3868 | 526.2875 | 15.18 | 5549 | 728.4522 | 11.23 |
| 507 | 137.0458 | 5.38 | 2188 | 393.6937 | 7.78 | 3869 | 526.2913 | 15.12 | 5550 | 728.6395 | 9.65 |
| 508 | 137.0462 | 5.13 | 2189 | 393.6979 | 7.84 | 3870 | 526.2922 | 10.93 | 5551 | 728.8984 | 9.64 |
| 509 | 137.0463 | 5.11 | 2190 | 393.6991 | 7.76 | 3871 | 526.2928 | 9.43 | 5552 | 729.1370 | 9.65 |
| 510 | 137.0473 | 5.62 | 2191 | 393.7042 | 7.68 | 3872 | 526.2934 | 18.22 | 5553 | 729.8798 | 9.86 |
| 511 | 137.0475 | 6.01 | 2192 | 393.7049 | 7.53 | 3873 | 526.2974 | 15.16 | 5554 | 730.7284 | 11.55 |
| 512 | 137.0662 | 0.82 | 2193 | 393.7086 | 8.02 | 3874 | 526.2997 | 18.16 | 5555 | 730.7306 | 9.92 |
| 513 | 138.0479 | 2.06 | 2194 | 393.7175 | 5.13 | 3875 | 526.2997 | 11.22 | 5556 | 730.7311 | 11.44 |
| 514 | 138.0482 | 5.40 | 2195 | 393.7196 | 5.46 | 3876 | 526.3006 | 11.26 | 5557 | 730.7320 | 9.71 |
| 515 | 138.0540 | 1.20 | 2196 | 393.7207 | 5.59 | 3877 | 526.3500 | 18.70 | 5558 | 730.7343 | 9.49 |
| 516 | 138.0547 | 1.05 | 2197 | 393.7218 | 6.16 | 3878 | 526.3765 | 20.94 | 5559 | 730.7346 | 9.65 |
| 517 | 138.0555 | 1.18 | 2198 | 393.7226 | 6.00 | 3879 | 526.3771 | 17.08 | 5560 | 730.7351 | 9.52 |
| 518 | 139.0364 | 19.15 | 2199 | 393.7232 | 5.33 | 3880 | 526.3787 | 17.24 | 5561 | 730.7355 | 11.17 |
| 519 | 139.9883 | 21.70 | 2200 | 393.7246 | 5.63 | 3881 | 526.3790 | 17.17 | 5562 | 731.0604 | 11.11 |
| 520 | 140.0649 | 0.96 | 2201 | 393.7265 | 5.86 | 3882 | 526.3865 | 17.09 | 5563 | 731.0625 | 9.65 |
| 521 | 141.0683 | 11.87 | 2202 | 393.8694 | 7.68 | 3883 | 527.1383 | 0.93 | 5564 | 731.0662 | 9.92 |
| 522 | 141.0732 | 0.96 | 2203 | 393.8709 | 8.02 | 3884 | 527.1570 | 1.20 | 5565 | 731.0676 | 9.71 |
| 523 | 141.9572 | 10.12 | 2204 | 393.8710 | 7.85 | 3885 | 527.2978 | 15.16 | 5566 | 731.0699 | 9.49 |
| 524 | 141.9577 | 21.88 | 2205 | 393.8725 | 7.75 | 3886 | 527.3002 | 18.22 | 5567 | 731.0703 | 9.50 |
| 525 | 141.9595 | 21.91 | 2206 | 393.8731 | 7.51 | 3887 | 527.3040 | 15.18 | 5568 | 731.0719 | 11.56 |
| 526 | 141.9810 | 21.70 | 2207 | 393.8755 | 7.79 | 3888 | 527.3062 | 14.98 | 5569 | 731.0743 | 11.44 |
| 527 | 143.0014 | 0.68 | 2208 | 394.0322 | 7.76 | 3889 | 527.3218 | 10.95 | 5570 | 731.3940 | 9.49 |
| 528 | 143.0020 | 21.63 | 2209 | 394.0384 | 7.50 | 3890 | 527.3245 | 11.39 | 5571 | 731.3958 | 11.55 |
| 529 | 143.9577 | 21.69 | 2210 | 394.0688 | 0.97 | 3891 | 527.3246 | 11.03 | 5572 | 731.3989 | 9.53 |
| 530 | 144.0793 | 7.62 | 2211 | 394.1959 | 10.95 | 3892 | 527.3826 | 20.98 | 5573 | 731.3991 | 9.67 |
| 531 | 144.0799 | 8.15 | 2212 | 394.2120 | 12.65 | 3893 | 527.3853 | 17.27 | 5574 | 731.3994 | 11.20 |
| 532 | 144.0805 | 7.91 | 2213 | 394.2237 | 6.00 | 3894 | 527.3891 | 17.17 | 5575 | 731.4018 | 9.92 |
| 533 | 144.0807 | 7.78 | 2214 | 394.2238 | 5.14 | 3895 | 527.9247 | 0.89 | 5576 | 731.4022 | 9.50 |
| 534 | 144.0807 | 6.86 | 2215 | 394.2249 | 5.60 | 3896 | 528.2435 | 8.21 | 5577 | 731.4033 | 11.16 |
| 535 | 144.0808 | 8.43 | 2216 | 394.2271 | 5.33 | 3897 | 528.2453 | 8.04 | 5578 | 731.7221 | 9.49 |
| 536 | 144.0809 | 8.31 | 2217 | 394.2633 | 10.80 | 3898 | 528.2558 | 8.24 | 5579 | 731.7302 | 9.50 |
| 537 | 144.0809 | 8.44 | 2218 | 394.2639 | 10.82 | 3899 | 528.2785 | 10.81 | 5580 | 731.7318 | 11.56 |
| 538 | 144.1024 | 1.14 | 2219 | 394.8703 | 0.83 | 3900 | 528.2983 | 18.26 | 5581 | 731.7351 | 11.13 |
| 539 | 144.1026 | 1.15 | 2220 | 395.2086 | 21.33 | 3901 | 528.3047 | 18.61 | 5582 | 731.7415 | 9.65 |
| 540 | 144.1026 | 1.17 | 2221 | 395.2169 | 19.15 | 3902 | 528.7844 | 10.81 | 5583 | 731.8967 | 0.85 |
| 541 | 145.0173 | 0.66 | 2222 | 395.2183 | 19.36 | 3903 | 528.8833 | 9.49 | 5584 | 732.0704 | 11.54 |
| 542 | 145.0175 | 21.65 | 2223 | 395.2199 | 21.64 | 3904 | 529.0774 | 9.51 | 5585 | 732.4070 | 11.55 |
| 543 | 145.0504 | 1.02 | 2224 | 395.2217 | 19.46 | 3905 | 529.2605 | 8.54 | 5586 | 733.3276 | 8.22 |
| 544 | 145.1049 | 1.12 | 2225 | 395.2278 | 19.07 | 3906 | 529.2615 | 10.57 | 5587 | 733.3382 | 9.94 |
| 545 | 145.1227 | 13.17 | 2226 | 395.2433 | 10.78 | 3907 | 529.2619 | 9.07 | 5588 | 733.8351 | 9.92 |
| 546 | 145.1244 | 16.25 | 2227 | 395.6998 | 10.97 | 3908 | 529.2644 | 8.74 | 5589 | 733.8530 | 0.76 |
| 547 | 145.9547 | 21.72 | 2228 | 396.0450 | 8.72 | 3909 | 529.2647 | 8.75 | 5590 | 734.3363 | 9.94 |
| 548 | 146.0186 | 0.68 | 2229 | 396.1897 | 10.11 | 3910 | 529.2669 | 8.57 | 5591 | 734.3371 | 9.95 |
| 549 | 146.0601 | 7.09 | 2230 | 396.1967 | 10.06 | 3911 | 529.2676 | 8.89 | 5592 | 734.3447 | 10.43 |
| 550 | 146.0604 | 7.15 | 2231 | 396.2020 | 10.98 | 3912 | 529.2678 | 9.22 | 5593 | 734.3532 | 10.28 |
| 551 | 146.0605 | 6.67 | 2232 | 396.2748 | 21.61 | 3913 | 529.2680 | 10.64 | 5594 | 734.8109 | 0.84 |
| 552 | 146.0615 | 6.86 | 2233 | 397.1236 | 21.62 | 3914 | 529.2851 | 9.51 | 5595 | 735.8486 | 11.20 |
| 553 | 146.0805 | 0.95 | 2234 | 397.1733 | 10.17 | 3915 | 529.3095 | 8.33 | 5596 | 736.1050 | 11.20 |
| 554 | 146.1161 | 0.87 | 2235 | 397.1774 | 10.06 | 3916 | 529.3130 | 7.16 | 5597 | 736.3577 | 11.18 |
| 555 | 146.1176 | 1.49 | 2236 | 397.1775 | 10.01 | 3917 | 529.3133 | 8.40 | 5598 | 736.6063 | 11.20 |
| 556 | 146.9956 | 0.65 | 2237 | 397.2722 | 17.83 | 3918 | 529.4830 | 9.51 | 5599 | 736.8477 | 0.80 |
| 557 | 147.0762 | 0.95 | 2238 | 397.2724 | 17.81 | 3919 | 529.6035 | 10.81 | 5600 | 737.3677 | 0.83 |
| 558 | 147.0775 | 0.93 | 2239 | 397.2747 | 17.67 | 3920 | 529.6411 | 8.40 | 5601 | 737.3705 | 10.03 |
| 559 | 147.1127 | 0.68 | 2240 | 399.0829 | 0.87 | 3921 | 529.6437 | 8.33 | 5602 | 741.3686 | 10.46 |
| 560 | 148.0029 | 0.94 | 2241 | 399.0835 | 1.01 | 3922 | 529.6473 | 7.16 | 5603 | 741.8639 | 10.45 |
| 561 | 148.0619 | 0.98 | 2242 | 399.1805 | 13.85 | 3923 | 529.9378 | 10.81 | 5604 | 741.9454 | 8.05 |
| 562 | 149.0218 | 19.74 | 2243 | 399.2365 | 6.38 | 3924 | 529.9650 | 8.34 | 5605 | 742.1413 | 8.05 |
| 563 | 149.0224 | 19.69 | 2244 | 399.2434 | 19.27 | 3925 | 529.9820 | 8.41 | 5606 | 742.3411 | 8.05 |
| 564 | 149.0226 | 16.36 | 2245 | 399.2462 | 16.10 | 3926 | 530.2643 | 8.89 | 5607 | 742.3845 | 7.69 |
| 565 | 149.0227 | 16.27 | 2246 | 399.2467 | 16.19 | 3927 | 530.2678 | 8.75 | 5608 | 743.3917 | 7.69 |
| 566 | 149.0228 | 16.68 | 2247 | 399.2481 | 16.00 | 3928 | 530.2678 | 10.57 | 5609 | 743.6856 | 8.46 |
| 567 | 149.0228 | 16.61 | 2248 | 399.2483 | 19.23 | 3929 | 530.2683 | 9.07 | 5610 | 743.6884 | 8.14 |
| 568 | 149.0234 | 19.72 | 2249 | 399.7110 | 7.04 | 3930 | 530.2684 | 10.62 | 5611 | 743.6890 | 8.22 |
| 569 | 149.0239 | 16.52 | 2250 | 400.0693 | 0.90 | 3931 | 530.2686 | 9.09 | 5612 | 743.6926 | 8.29 |
| 570 | 149.0240 | 19.71 | 2251 | 400.1832 | 8.99 | 3932 | 530.2711 | 10.64 | 5613 | 743.6986 | 7.90 |
| 571 | 149.0243 | 13.52 | 2252 | 400.1878 | 10.62 | 3933 | 530.2734 | 8.54 | 5614 | 744.0274 | 8.14 |
| 572 | 149.0244 | 16.64 | 2253 | 400.3450 | 15.93 | 3934 | 530.3154 | 10.71 | 5615 | 744.0350 | 8.31 |
| 573 | 149.0246 | 16.62 | 2254 | 400.3799 | 16.53 | 3935 | 530.3162 | 10.71 | 5616 | 744.0356 | 8.46 |
| 574 | 149.0592 | 5.40 | 2255 | 401.1929 | 10.62 | 3936 | 530.8104 | 8.75 | 5617 | 744.0358 | 8.47 |
| 575 | 149.9501 | 0.66 | 2256 | 401.2332 | 17.56 | 3937 | 530.8198 | 10.72 | 5618 | 744.0390 | 8.22 |
| 576 | 150.0137 | 0.88 | 2257 | 401.2362 | 17.47 | 3938 | 530.8450 | 0.81 | 5619 | 744.0410 | 7.90 |
| 577 | 150.0139 | 0.86 | 2258 | 401.7046 | 10.97 | 3939 | 530.8483 | 0.79 | 5620 | 744.3620 | 8.29 |
| 578 | 150.0246 | 16.40 | 2259 | 402.1828 | 10.70 | 3940 | 530.9784 | 0.92 | 5621 | 744.3665 | 8.46 |
| 579 | 150.0246 | 16.35 | 2260 | 402.2369 | 17.57 | 3941 | 531.0173 | 0.77 | 5622 | 744.3719 | 7.90 |
| 580 | 150.0261 | 16.31 | 2261 | 402.3551 | 12.91 | 3942 | 531.2974 | 11.77 | 5623 | 744.7084 | 8.29 |
| 581 | 150.0270 | 19.78 | 2262 | 402.3630 | 15.71 | 3943 | 531.2981 | 10.28 | 5624 | 744.7206 | 8.46 |
| 582 | 150.0288 | 16.49 | 2263 | 402.9049 | 0.68 | 3944 | 531.3139 | 10.66 | 5625 | 744.8428 | 0.77 |
| 583 | 150.0578 | 1.44 | 2264 | 403.1847 | 10.70 | 3945 | 531.7746 | 10.07 | 5626 | 745.0285 | 8.46 |
| 584 | 150.0582 | 1.58 | 2265 | 403.2473 | 8.81 | 3946 | 531.7766 | 8.37 | 5627 | 745.3420 | 0.80 |
| 585 | 150.0595 | 1.47 | 2266 | 404.2287 | 7.76 | 3947 | 531.7769 | 9.89 | 5628 | 747.3692 | 9.96 |
| 586 | 150.0595 | 1.53 | 2267 | 404.2297 | 5.53 | 3948 | 531.7772 | 10.10 | 5629 | 747.3828 | 10.15 |
| 587 | 150.0597 | 1.51 | 2268 | 404.2299 | 4.99 | 3949 | 531.7786 | 8.16 | 5630 | 747.3849 | 9.83 |
| 588 | 151.0598 | 1.44 | 2269 | 404.2320 | 5.36 | 3950 | 531.7790 | 10.14 | 5631 | 747.9034 | 8.75 |
| 589 | 151.1436 | 0.84 | 2270 | 404.2334 | 5.72 | 3951 | 531.7794 | 9.97 | 5632 | 747.9132 | 9.15 |
| 590 | 152.0547 | 1.44 | 2271 | 405.0998 | 0.96 | 3952 | 531.7803 | 10.12 | 5633 | 748.3744 | 10.17 |
| 591 | 152.0696 | 6.85 | 2272 | 405.1209 | 1.88 | 3953 | 531.7834 | 10.04 | 5634 | 748.3760 | 9.85 |
| 592 | 152.0709 | 7.23 | 2273 | 405.1212 | 1.44 | 3954 | 532.2747 | 10.04 | 5635 | 748.3777 | 10.31 |
| 593 | 152.9458 | 0.71 | 2274 | 405.5379 | 7.99 | 3955 | 532.2764 | 9.89 | 5636 | 748.3836 | 9.96 |
| 594 | 153.0635 | 4.84 | 2275 | 405.5449 | 8.24 | 3956 | 532.2800 | 9.96 | 5637 | 748.4224 | 9.15 |
| 595 | 153.0643 | 5.46 | 2276 | 405.7102 | 8.21 | 3957 | 532.2836 | 9.97 | 5638 | 748.8780 | 10.33 |
| 596 | 153.0644 | 5.72 | 2277 | 405.7113 | 8.08 | 3958 | 532.3258 | 19.83 | 5639 | 749.3712 | 10.15 |
| 597 | 153.0646 | 4.46 | 2278 | 405.7115 | 8.01 | 3959 | 532.3334 | 16.22 | 5640 | 749.3752 | 10.01 |
| 598 | 153.0648 | 5.21 | 2279 | 405.8744 | 8.24 | 3960 | 532.5848 | 10.19 | 5641 | 749.3831 | 9.77 |
| 599 | 153.0663 | 4.78 | 2280 | 407.2207 | 7.29 | 3961 | 532.7783 | 10.15 | 5642 | 749.3876 | 8.22 |
| 600 | 153.0665 | 5.16 | 2281 | 408.2252 | 17.82 | 3962 | 532.9233 | 10.19 | 5643 | 749.4577 | 11.33 |
| 601 | 153.0665 | 5.18 | 2282 | 408.2286 | 7.30 | 3963 | 534.2887 | 18.94 | 5644 | 750.0566 | 10.20 |
| 602 | 153.0669 | 4.91 | 2283 | 408.4008 | 6.41 | 3964 | 534.2966 | 15.69 | 5645 | 750.0637 | 8.10 |
| 603 | 154.0857 | 1.09 | 2284 | 408.4089 | 5.61 | 3965 | 534.2974 | 15.24 | 5646 | 750.3832 | 10.01 |
| 604 | 154.1232 | 6.59 | 2285 | 408.4091 | 5.01 | 3966 | 534.2996 | 15.43 | 5647 | 750.3908 | 10.15 |
| 605 | 155.1548 | 6.58 | 2286 | 408.4093 | 6.72 | 3967 | 534.3000 | 15.71 | 5648 | 750.3955 | 8.11 |
| 606 | 155.1552 | 6.29 | 2287 | 408.8993 | 4.93 | 3968 | 534.3039 | 15.55 | 5649 | 750.7437 | 8.10 |
| 607 | 156.0406 | 0.99 | 2288 | 408.9113 | 6.37 | 3969 | 535.2658 | 9.39 | 5650 | 751.0679 | 8.11 |
| 608 | 156.0409 | 0.92 | 2289 | 408.9169 | 5.53 | 3970 | 535.2693 | 8.02 | 5651 | 752.3493 | 10.17 |
| 609 | 156.0759 | 1.38 | 2290 | 408.9199 | 0.73 | 3971 | 535.2711 | 9.31 | 5652 | 752.8350 | 0.75 |
| 610 | 156.0764 | 1.45 | 2291 | 409.2320 | 17.92 | 3972 | 535.2711 | 8.87 | 5653 | 752.8417 | 0.81 |
| 611 | 157.0163 | 0.62 | 2292 | 409.2920 | 19.42 | 3973 | 535.2712 | 9.34 | 5654 | 753.4399 | 10.02 |
| 612 | 158.1528 | 12.94 | 2293 | 412.0519 | 16.14 | 3974 | 535.2714 | 7.68 | 5655 | 753.7425 | 9.03 |
| 613 | 158.1529 | 1.43 | 2294 | 412.2839 | 15.89 | 3975 | 535.2724 | 9.77 | 5656 | 754.7255 | 8.05 |
| 614 | 158.1534 | 13.43 | 2295 | 412.2888 | 8.45 | 3976 | 535.2747 | 8.34 | 5657 | 754.7276 | 8.16 |
| 615 | 158.1541 | 1.47 | 2296 | 412.2894 | 8.39 | 3977 | 535.2748 | 8.32 | 5658 | 754.7316 | 10.10 |
| 616 | 158.1542 | 16.19 | 2297 | 412.2921 | 7.66 | 3978 | 535.2758 | 9.38 | 5659 | 755.0507 | 8.23 |
| 617 | 158.1542 | 13.13 | 2298 | 412.2922 | 8.54 | 3979 | 535.2765 | 8.51 | 5660 | 755.0513 | 10.06 |
| 618 | 158.1543 | 1.33 | 2299 | 412.2930 | 8.62 | 3980 | 535.2781 | 9.25 | 5661 | 755.0584 | 8.07 |
| 619 | 158.1546 | 1.38 | 2300 | 412.2935 | 8.30 | 3981 | 535.2785 | 7.91 | 5662 | 755.0769 | 8.96 |
| 620 | 158.1546 | 1.42 | 2301 | 412.2963 | 8.63 | 3982 | 535.2946 | 15.56 | 5663 | 755.3881 | 8.22 |
| 621 | 158.1550 | 13.26 | 2302 | 412.8573 | 0.69 | 3983 | 535.2947 | 15.69 | 5664 | 755.3942 | 8.16 |
| 622 | 158.1556 | 16.14 | 2303 | 413.1027 | 0.89 | 3984 | 535.2981 | 18.89 | 5665 | 755.4129 | 9.63 |
| 623 | 158.9646 | 0.82 | 2304 | 413.2328 | 11.74 | 3985 | 535.7652 | 9.39 | 5666 | 755.4204 | 7.82 |
| 624 | 159.0270 | 2.46 | 2305 | 413.2869 | 15.89 | 3986 | 535.7674 | 7.69 | 5667 | 755.7294 | 8.05 |
| 625 | 159.0284 | 2.09 | 2306 | 413.2953 | 8.52 | 3987 | 535.7700 | 9.41 | 5668 | 755.9167 | 7.83 |
| 626 | 159.0790 | 0.96 | 2307 | 413.2959 | 8.65 | 3988 | 535.7705 | 8.87 | 5669 | 755.9170 | 9.63 |
| 627 | 159.0883 | 6.86 | 2308 | 413.7376 | 11.74 | 3989 | 535.7706 | 9.33 | 5670 | 755.9579 | 8.02 |
| 628 | 160.0335 | 1.04 | 2309 | 414.0419 | 1.06 | 3990 | 535.7712 | 7.92 | 5671 | 756.1536 | 8.12 |
| 629 | 160.0347 | 1.00 | 2310 | 414.0420 | 0.89 | 3991 | 535.7718 | 9.77 | 5672 | 756.1562 | 9.97 |
| 630 | 160.0985 | 1.03 | 2311 | 414.0437 | 1.01 | 3992 | 535.7719 | 9.38 | 5673 | 756.1579 | 9.99 |
| 631 | 160.1312 | 1.10 | 2312 | 414.0449 | 1.40 | 3993 | 535.7740 | 7.68 | 5674 | 756.1590 | 8.04 |
| 632 | 160.1318 | 8.71 | 2313 | 414.0546 | 9.52 | 3994 | 535.7771 | 9.34 | 5675 | 756.3356 | 10.28 |
| 633 | 162.1114 | 0.87 | 2314 | 414.2984 | 17.86 | 3995 | 535.7772 | 9.09 | 5676 | 756.3475 | 8.12 |
| 634 | 162.1118 | 0.86 | 2315 | 414.2985 | 14.14 | 3996 | 535.7791 | 8.51 | 5677 | 756.3607 | 8.04 |
| 635 | 162.1119 | 1.09 | 2316 | 414.2991 | 17.72 | 3997 | 536.2736 | 7.68 | 5678 | 756.3623 | 9.84 |
| 636 | 162.1122 | 0.80 | 2317 | 414.3020 | 17.40 | 3998 | 536.2738 | 8.85 | 5679 | 756.3643 | 10.02 |
| 637 | 162.1125 | 0.83 | 2318 | 414.3030 | 15.82 | 3999 | 536.2779 | 9.39 | 5680 | 756.5585 | 8.04 |
| 638 | 162.9732 | 21.62 | 2319 | 415.0448 | 1.02 | 4000 | 536.3002 | 15.69 | 5681 | 756.5703 | 10.01 |
| 639 | 163.0560 | 1.00 | 2320 | 415.0808 | 0.87 | 4001 | 536.4118 | 19.40 | 5682 | 756.7642 | 8.04 |
| 640 | 163.1154 | 0.86 | 2321 | 415.2439 | 10.22 | 4002 | 538.8695 | 0.70 | 5683 | 757.2373 | 0.99 |
| 641 | 164.0276 | 0.90 | 2322 | 415.2881 | 8.94 | 4003 | 539.2340 | 6.49 | 5684 | 758.8184 | 0.71 |
| 642 | 164.0924 | 0.95 | 2323 | 415.2890 | 9.16 | 4004 | 539.9701 | 0.83 | 5685 | 760.4543 | 6.84 |
| 643 | 165.0139 | 0.98 | 2324 | 415.2895 | 7.82 | 4005 | 539.9730 | 12.06 | 5686 | 760.4559 | 7.04 |
| 644 | 165.0545 | 2.26 | 2325 | 415.2903 | 8.92 | 4006 | 539.9733 | 12.03 | 5687 | 760.4567 | 6.92 |
| 645 | 165.0558 | 2.47 | 2326 | 415.2903 | 10.57 | 4007 | 540.2906 | 14.54 | 5688 | 760.4604 | 7.03 |
| 646 | 165.0558 | 2.44 | 2327 | 415.2905 | 8.95 | 4008 | 540.2989 | 17.62 | 5689 | 760.4663 | 6.74 |
| 647 | 165.0559 | 2.16 | 2328 | 415.2905 | 10.53 | 4009 | 540.3084 | 12.05 | 5690 | 760.8179 | 0.83 |
| 648 | 165.0565 | 2.61 | 2329 | 415.2905 | 8.01 | 4010 | 541.3659 | 19.37 | 5691 | 761.4558 | 7.04 |
| 649 | 165.0931 | 0.95 | 2330 | 415.2906 | 10.54 | 4011 | 542.3173 | 18.13 | 5692 | 761.6858 | 10.91 |
| 650 | 165.1135 | 21.31 | 2331 | 415.2912 | 9.62 | 4012 | 542.3174 | 14.69 | 5693 | 762.0222 | 10.89 |
| 651 | 166.0835 | 1.14 | 2332 | 415.2913 | 9.28 | 4013 | 542.3187 | 18.16 | 5694 | 762.0246 | 8.76 |
| 652 | 166.0836 | 1.11 | 2333 | 415.2915 | 9.69 | 4014 | 542.3192 | 14.90 | 5695 | 762.3644 | 10.94 |
| 653 | 166.0836 | 1.12 | 2334 | 415.2917 | 9.60 | 4015 | 542.3195 | 18.14 | 5696 | 762.9060 | 8.72 |
| 654 | 166.0842 | 4.50 | 2335 | 415.2921 | 8.57 | 4016 | 542.3198 | 18.21 | 5697 | 762.9067 | 8.94 |
| 655 | 166.0849 | 5.40 | 2336 | 415.2931 | 8.03 | 4017 | 542.3205 | 18.18 | 5698 | 762.9778 | 15.63 |
| 656 | 166.0855 | 5.13 | 2337 | 415.2933 | 8.05 | 4018 | 542.3205 | 15.02 | 5699 | 763.2551 | 0.96 |
| 657 | 166.0855 | 4.13 | 2338 | 415.2933 | 8.37 | 4019 | 542.3213 | 18.27 | 5700 | 763.3839 | 8.64 |
| 658 | 166.0859 | 4.82 | 2339 | 415.2935 | 8.73 | 4020 | 542.3213 | 14.70 | 5701 | 763.4050 | 8.92 |
| 659 | 166.0860 | 5.11 | 2340 | 415.2937 | 8.90 | 4021 | 542.3215 | 17.50 | 5702 | 763.8887 | 8.94 |
| 660 | 166.0860 | 4.86 | 2341 | 415.2939 | 8.14 | 4022 | 542.3218 | 17.60 | 5703 | 764.3660 | 7.71 |
| 661 | 166.0861 | 5.02 | 2342 | 415.2939 | 10.62 | 4023 | 542.3224 | 18.15 | 5704 | 764.8367 | 0.73 |
| 662 | 166.0862 | 5.90 | 2343 | 415.2942 | 9.29 | 4024 | 542.3231 | 17.98 | 5705 | 765.3381 | 9.77 |
| 663 | 166.0863 | 4.64 | 2344 | 415.2951 | 8.62 | 4025 | 542.3231 | 18.19 | 5706 | 765.9750 | 18.83 |
| 664 | 166.0866 | 5.72 | 2345 | 415.2963 | 8.79 | 4026 | 542.3232 | 14.94 | 5707 | 765.9879 | 15.53 |
| 665 | 166.0867 | 5.98 | 2346 | 415.2980 | 8.60 | 4027 | 542.3241 | 15.29 | 5708 | 765.9901 | 15.49 |
| 666 | 166.0867 | 6.20 | 2347 | 415.3013 | 17.85 | 4028 | 542.3243 | 15.21 | 5709 | 765.9961 | 15.58 |
| 667 | 166.0868 | 5.07 | 2348 | 415.3026 | 15.82 | 4029 | 542.3243 | 17.63 | 5710 | 765.9991 | 15.81 |
| 668 | 166.0873 | 4.09 | 2349 | 415.3046 | 17.40 | 4030 | 542.3245 | 15.16 | 5711 | 766.0051 | 15.67 |
| 669 | 166.0875 | 5.51 | 2350 | 415.3055 | 15.79 | 4031 | 542.3246 | 18.30 | 5712 | 766.4798 | 15.53 |
| 670 | 166.0877 | 5.10 | 2351 | 415.4152 | 7.59 | 4032 | 542.3251 | 14.54 | 5713 | 766.4917 | 18.82 |
| 671 | 166.0885 | 6.14 | 2352 | 415.4188 | 7.50 | 4033 | 542.3254 | 14.47 | 5714 | 766.4926 | 18.90 |
| 672 | 166.1043 | 0.82 | 2353 | 415.4191 | 7.76 | 4034 | 542.3259 | 14.42 | 5715 | 767.8414 | 0.75 |
| 673 | 167.0107 | 21.70 | 2354 | 415.9161 | 7.56 | 4035 | 542.3267 | 17.45 | 5716 | 768.4178 | 9.85 |
| 674 | 167.0872 | 16.59 | 2355 | 416.0176 | 18.25 | 4036 | 542.3285 | 19.09 | 5717 | 768.4198 | 10.61 |
| 675 | 167.0872 | 5.16 | 2356 | 416.1970 | 10.40 | 4037 | 542.3309 | 18.19 | 5718 | 768.4239 | 10.09 |
| 676 | 167.0880 | 5.41 | 2357 | 416.2812 | 9.74 | 4038 | 542.7585 | 10.02 | 5719 | 768.4295 | 11.89 |
| 677 | 167.0893 | 5.92 | 2358 | 416.2897 | 9.60 | 4039 | 543.2546 | 14.49 | 5720 | 768.4329 | 9.71 |
| 678 | 167.0896 | 5.72 | 2359 | 416.2898 | 9.16 | 4040 | 543.2571 | 17.73 | 5721 | 768.7541 | 9.85 |
| 679 | 167.0898 | 5.43 | 2360 | 416.2913 | 8.95 | 4041 | 543.2572 | 14.69 | 5722 | 768.7580 | 11.89 |
| 680 | 167.0898 | 5.40 | 2361 | 416.2914 | 7.91 | 4042 | 543.2598 | 17.66 | 5723 | 768.7602 | 10.09 |
| 681 | 167.0899 | 5.13 | 2362 | 416.2915 | 8.73 | 4043 | 543.2616 | 14.54 | 5724 | 768.7616 | 10.24 |
| 682 | 167.0915 | 5.44 | 2363 | 416.2919 | 8.12 | 4044 | 543.2623 | 14.34 | 5725 | 768.7639 | 10.61 |
| 683 | 168.5825 | 5.75 | 2364 | 416.2931 | 8.60 | 4045 | 543.3200 | 15.30 | 5726 | 768.7653 | 9.71 |
| 684 | 168.5870 | 5.96 | 2365 | 416.2933 | 7.82 | 4046 | 543.3201 | 15.13 | 5727 | 769.0885 | 10.61 |
| 685 | 169.0345 | 1.93 | 2366 | 416.2939 | 10.55 | 4047 | 543.3201 | 15.16 | 5728 | 769.0887 | 10.09 |
| 686 | 169.0347 | 1.98 | 2367 | 416.2940 | 8.38 | 4048 | 543.3221 | 14.98 | 5729 | 769.0904 | 11.89 |
| 687 | 169.0358 | 2.31 | 2368 | 416.2942 | 8.37 | 4049 | 543.3225 | 18.17 | 5730 | 769.0916 | 9.72 |
| 688 | 169.0360 | 2.20 | 2369 | 416.2942 | 10.57 | 4050 | 543.3231 | 15.20 | 5731 | 769.0941 | 10.24 |
| 689 | 169.0360 | 2.05 | 2370 | 416.2945 | 8.90 | 4051 | 543.3233 | 18.19 | 5732 | 769.0983 | 9.85 |
| 690 | 169.0367 | 2.22 | 2371 | 416.2947 | 8.14 | 4052 | 543.3243 | 14.45 | 5733 | 769.1543 | 7.03 |
| 691 | 169.0369 | 2.55 | 2372 | 416.2949 | 9.62 | 4053 | 543.3250 | 18.14 | 5734 | 769.4190 | 11.89 |
| 692 | 169.0592 | 0.90 | 2373 | 416.2957 | 8.21 | 4054 | 543.3262 | 15.22 | 5735 | 769.4212 | 10.09 |
| 693 | 169.9875 | 0.93 | 2374 | 416.2968 | 8.03 | 4055 | 543.3266 | 14.55 | 5736 | 769.4264 | 9.71 |
| 694 | 170.0368 | 2.59 | 2375 | 416.2971 | 10.53 | 4056 | 543.3277 | 14.47 | 5737 | 769.4308 | 9.85 |
| 695 | 170.0415 | 0.98 | 2376 | 416.2972 | 10.54 | 4057 | 543.3293 | 18.24 | 5738 | 769.7460 | 10.09 |
| 696 | 170.0640 | 0.66 | 2377 | 416.2976 | 10.58 | 4058 | 543.3358 | 17.61 | 5739 | 769.7667 | 11.86 |
| 697 | 171.1483 | 10.86 | 2378 | 416.2979 | 9.29 | 4059 | 543.3842 | 8.54 | 5740 | 770.0960 | 11.89 |
| 698 | 171.1483 | 10.99 | 2379 | 416.3016 | 8.63 | 4060 | 543.3867 | 8.45 | 5741 | 770.8532 | 1.04 |
| 699 | 171.1484 | 10.88 | 2380 | 416.3022 | 17.39 | 4061 | 543.3880 | 7.66 | 5742 | 770.8597 | 0.76 |
| 700 | 171.1484 | 10.95 | 2381 | 416.3064 | 15.82 | 4062 | 543.3939 | 7.45 | 5743 | 771.3509 | 0.79 |
| 701 | 171.1488 | 13.11 | 2382 | 417.2327 | 10.47 | 4063 | 544.2600 | 14.75 | 5744 | 771.8458 | 0.78 |
| 702 | 171.1489 | 11.29 | 2383 | 417.2388 | 8.97 | 4064 | 544.2672 | 14.60 | 5745 | 771.9429 | 15.75 |
| 703 | 171.1490 | 13.06 | 2384 | 417.2455 | 9.39 | 4065 | 544.3167 | 19.11 | 5746 | 771.9687 | 15.63 |
| 704 | 171.1501 | 10.89 | 2385 | 417.2461 | 9.39 | 4066 | 544.3319 | 15.77 | 5747 | 771.9707 | 18.89 |
| 705 | 171.1501 | 10.77 | 2386 | 417.2461 | 8.18 | 4067 | 544.3319 | 15.89 | 5748 | 771.9736 | 15.67 |
| 706 | 171.1502 | 10.94 | 2387 | 417.2471 | 8.92 | 4068 | 544.3324 | 17.61 | 5749 | 771.9773 | 15.48 |
| 707 | 172.0390 | 14.17 | 2388 | 417.2482 | 9.79 | 4069 | 544.3357 | 19.29 | 5750 | 772.4594 | 15.64 |
| 708 | 172.1319 | 6.57 | 2389 | 417.2487 | 9.40 | 4070 | 544.3357 | 19.12 | 5751 | 772.4656 | 18.93 |
| 709 | 172.1498 | 10.86 | 2390 | 417.2495 | 7.82 | 4071 | 544.3363 | 21.46 | 5752 | 772.4663 | 15.43 |
| 710 | 172.1504 | 13.09 | 2391 | 417.2496 | 7.80 | 4072 | 544.3363 | 15.31 | 5753 | 772.4675 | 15.72 |
| 711 | 172.1509 | 11.06 | 2392 | 417.2499 | 9.10 | 4073 | 544.3365 | 16.06 | 5754 | 772.4756 | 15.62 |
| 712 | 172.1516 | 10.91 | 2393 | 417.2500 | 8.38 | 4074 | 544.3365 | 18.15 | 5755 | 772.8618 | 0.78 |
| 713 | 172.1523 | 11.29 | 2394 | 417.2501 | 8.05 | 4075 | 544.3366 | 16.05 | 5756 | 772.9526 | 15.76 |
| 714 | 172.1536 | 10.99 | 2395 | 417.2503 | 7.97 | 4076 | 544.3379 | 14.91 | 5757 | 772.9598 | 15.62 |
| 715 | 173.0113 | 0.67 | 2396 | 417.2516 | 9.30 | 4077 | 544.3384 | 14.98 | 5758 | 772.9660 | 15.49 |
| 716 | 173.0408 | 1.06 | 2397 | 417.2517 | 8.56 | 4078 | 544.3385 | 18.20 | 5759 | 772.9698 | 18.84 |
| 717 | 173.1302 | 20.40 | 2398 | 417.2528 | 9.36 | 4079 | 544.3388 | 15.92 | 5760 | 772.9850 | 15.67 |
| 718 | 173.1348 | 16.82 | 2399 | 417.2530 | 9.37 | 4080 | 544.3389 | 15.83 | 5761 | 773.2430 | 11.15 |
| 719 | 173.1359 | 16.84 | 2400 | 417.3000 | 9.29 | 4081 | 544.3390 | 18.11 | 5762 | 773.4753 | 15.67 |
| 720 | 173.1382 | 6.60 | 2401 | 417.7469 | 9.39 | 4082 | 544.3391 | 18.23 | 5763 | 775.4329 | 6.51 |
| 721 | 174.9387 | 0.78 | 2402 | 417.7482 | 7.80 | 4083 | 544.3393 | 19.01 | 5764 | 777.4125 | 17.62 |
| 722 | 174.9909 | 1.77 | 2403 | 417.7487 | 8.91 | 4084 | 544.3398 | 15.16 | 5765 | 777.8581 | 6.51 |
| 723 | 175.0504 | 1.40 | 2404 | 417.7501 | 9.43 | 4085 | 544.3404 | 15.08 | 5766 | 777.8594 | 5.52 |
| 724 | 175.0590 | 1.01 | 2405 | 417.7518 | 8.97 | 4086 | 544.3415 | 15.07 | 5767 | 777.8596 | 6.56 |
| 725 | 175.1202 | 0.82 | 2406 | 417.7530 | 9.40 | 4087 | 544.3417 | 18.17 | 5768 | 777.8631 | 6.39 |
| 726 | 176.1210 | 0.89 | 2407 | 417.7545 | 7.95 | 4088 | 544.3417 | 18.19 | 5769 | 777.8647 | 5.11 |
| 727 | 177.0238 | 0.90 | 2408 | 418.1903 | 5.20 | 4089 | 544.3419 | 19.14 | 5770 | 777.8663 | 4.98 |
| 728 | 177.0606 | 0.79 | 2409 | 418.1917 | 5.39 | 4090 | 544.3421 | 18.22 | 5771 | 777.8667 | 7.04 |
| 729 | 177.1048 | 2.10 | 2410 | 418.1921 | 4.49 | 4091 | 544.3455 | 19.14 | 5772 | 778.8453 | 0.78 |
| 730 | 177.1256 | 21.35 | 2411 | 418.1925 | 4.99 | 4092 | 544.3457 | 18.97 | 5773 | 778.8613 | 4.95 |
| 731 | 178.1090 | 0.83 | 2412 | 418.1928 | 5.21 | 4093 | 544.3476 | 15.71 | 5774 | 778.8615 | 7.01 |
| 732 | 178.1317 | 6.59 | 2413 | 418.1929 | 4.89 | 4094 | 544.3907 | 8.54 | 5775 | 778.8663 | 6.49 |
| 733 | 178.1324 | 6.71 | 2414 | 418.1935 | 5.70 | 4095 | 544.7926 | 7.69 | 5776 | 778.8667 | 5.52 |
| 734 | 178.1338 | 6.61 | 2415 | 418.1944 | 3.06 | 4096 | 544.9000 | 0.76 | 5777 | 779.8538 | 6.51 |
| 735 | 178.1344 | 6.90 | 2416 | 418.1947 | 5.75 | 4097 | 545.2533 | 17.69 | 5778 | 779.8584 | 7.08 |
| 736 | 178.1357 | 6.62 | 2417 | 418.1959 | 4.37 | 4098 | 545.2575 | 14.76 | 5779 | 779.8598 | 5.53 |
| 737 | 179.1057 | 16.84 | 2418 | 418.1970 | 4.85 | 4099 | 545.2634 | 14.38 | 5780 | 779.8706 | 4.97 |
| 738 | 180.0801 | 11.82 | 2419 | 418.1975 | 4.64 | 4100 | 545.2643 | 14.65 | 5781 | 780.8638 | 4.94 |
| 739 | 181.0319 | 1.06 | 2420 | 418.1976 | 5.92 | 4101 | 545.2653 | 17.66 | 5782 | 780.8676 | 6.38 |
| 740 | 181.0713 | 6.90 | 2421 | 418.1976 | 4.77 | 4102 | 545.2684 | 14.50 | 5783 | 781.3704 | 8.25 |
| 741 | 181.0714 | 7.73 | 2422 | 418.2226 | 17.89 | 4103 | 545.3306 | 15.19 | 5784 | 781.3747 | 8.08 |
| 742 | 181.0722 | 7.08 | 2423 | 418.2461 | 9.40 | 4104 | 545.3347 | 15.08 | 5785 | 781.6192 | 8.08 |
| 743 | 181.0853 | 15.30 | 2424 | 418.2568 | 8.94 | 4105 | 545.3362 | 15.90 | 5786 | 781.6227 | 8.25 |
| 744 | 181.0859 | 12.47 | 2425 | 418.5494 | 6.50 | 4106 | 545.3375 | 16.10 | 5787 | 781.8597 | 8.08 |
| 745 | 181.0869 | 12.86 | 2426 | 418.5522 | 6.36 | 4107 | 545.3384 | 18.18 | 5788 | 781.8633 | 8.25 |
| 746 | 181.0875 | 12.46 | 2427 | 418.5528 | 6.86 | 4108 | 545.3394 | 18.16 | 5789 | 782.1082 | 8.07 |
| 747 | 181.0878 | 12.70 | 2428 | 418.5569 | 6.52 | 4109 | 545.3395 | 15.86 | 5790 | 782.4412 | 7.00 |
| 748 | 181.1000 | 17.24 | 2429 | 418.8782 | 0.65 | 4110 | 545.3397 | 15.92 | 5791 | 786.4009 | 7.76 |
| 749 | 181.1022 | 14.36 | 2430 | 419.2001 | 4.89 | 4111 | 545.3402 | 16.07 | 5792 | 786.7293 | 7.76 |
| 750 | 182.0790 | 2.16 | 2431 | 419.2244 | 17.84 | 4112 | 545.3405 | 15.17 | 5793 | 786.8232 | 0.81 |
| 751 | 182.0797 | 2.11 | 2432 | 419.7133 | 9.67 | 4113 | 545.3429 | 18.22 | 5794 | 787.0620 | 7.78 |
| 752 | 182.0798 | 2.61 | 2433 | 419.9646 | 9.70 | 4114 | 545.3440 | 16.05 | 5795 | 787.7591 | 8.44 |
| 753 | 182.0808 | 2.47 | 2434 | 420.2133 | 9.67 | 4115 | 545.3494 | 19.31 | 5796 | 787.8230 | 0.83 |
| 754 | 182.0810 | 2.48 | 2435 | 421.2288 | 15.97 | 4116 | 545.3503 | 15.31 | 5797 | 787.9302 | 11.00 |
| 755 | 182.0828 | 2.44 | 2436 | 421.2319 | 19.31 | 4117 | 546.3241 | 7.03 | 5798 | 788.0874 | 8.45 |
| 756 | 182.0832 | 2.26 | 2437 | 421.2331 | 16.19 | 4118 | 546.3267 | 7.06 | 5799 | 788.4240 | 8.45 |
| 757 | 182.0889 | 15.31 | 2438 | 421.2336 | 16.07 | 4119 | 546.3355 | 16.09 | 5800 | 788.4410 | 11.00 |
| 758 | 182.8995 | 0.64 | 2439 | 421.2544 | 15.59 | 4120 | 546.3389 | 15.17 | 5801 | 788.9401 | 11.00 |
| 759 | 184.0263 | 6.33 | 2440 | 421.2555 | 7.26 | 4121 | 546.3459 | 15.18 | 5802 | 789.4754 | 10.78 |
| 760 | 184.0266 | 5.98 | 2441 | 423.1628 | 15.47 | 4122 | 546.3477 | 19.26 | 5803 | 789.8437 | 4.97 |
| 761 | 184.0282 | 5.72 | 2442 | 423.2034 | 6.53 | 4123 | 546.3480 | 15.43 | 5804 | 790.3938 | 10.97 |
| 762 | 184.0287 | 6.03 | 2443 | 424.2021 | 6.53 | 4124 | 546.3491 | 17.13 | 5805 | 790.4784 | 10.78 |
| 763 | 184.0293 | 6.57 | 2444 | 424.3908 | 8.61 | 4125 | 546.3493 | 17.21 | 5806 | 791.8723 | 7.62 |
| 764 | 184.0911 | 0.85 | 2445 | 424.3920 | 8.95 | 4126 | 546.3493 | 17.24 | 5807 | 791.8755 | 7.50 |
| 765 | 184.0956 | 1.04 | 2446 | 424.5593 | 8.72 | 4127 | 546.3496 | 15.08 | 5808 | 791.8758 | 7.52 |
| 766 | 184.0960 | 0.82 | 2447 | 424.5594 | 8.62 | 4128 | 546.3511 | 17.05 | 5809 | 791.8799 | 7.48 |
| 767 | 185.0633 | 0.68 | 2448 | 424.5607 | 8.98 | 4129 | 546.3514 | 19.29 | 5810 | 791.8801 | 7.73 |
| 768 | 185.0973 | 16.23 | 2449 | 424.5658 | 8.92 | 4130 | 546.3529 | 17.17 | 5811 | 791.8813 | 7.66 |
| 769 | 185.1283 | 9.74 | 2450 | 424.7262 | 8.95 | 4131 | 546.3541 | 17.02 | 5812 | 792.4187 | 8.45 |
| 770 | 185.1289 | 7.10 | 2451 | 424.7280 | 8.61 | 4132 | 546.3542 | 18.76 | 5813 | 792.4192 | 8.71 |
| 771 | 186.0736 | 0.95 | 2452 | 424.8978 | 0.70 | 4133 | 546.3543 | 16.75 | 5814 | 792.4252 | 8.53 |
| 772 | 186.0984 | 16.19 | 2453 | 425.1574 | 21.66 | 4134 | 546.3544 | 17.03 | 5815 | 792.7496 | 9.35 |
| 773 | 186.2032 | 19.01 | 2454 | 425.1613 | 15.47 | 4135 | 546.3549 | 18.24 | 5816 | 792.7596 | 9.42 |
| 774 | 186.2032 | 18.82 | 2455 | 425.5055 | 8.46 | 4136 | 546.3551 | 15.76 | 5817 | 792.7647 | 8.71 |
| 775 | 186.2037 | 19.07 | 2456 | 425.5072 | 7.14 | 4137 | 546.3556 | 17.29 | 5818 | 792.7658 | 9.51 |
| 776 | 186.2076 | 20.88 | 2457 | 425.5100 | 8.34 | 4138 | 546.3560 | 16.81 | 5819 | 792.7678 | 8.84 |
| 777 | 186.2077 | 19.02 | 2458 | 425.5102 | 8.33 | 4139 | 546.3574 | 20.91 | 5820 | 792.8494 | 7.65 |
| 778 | 186.2087 | 18.77 | 2459 | 425.5109 | 8.40 | 4140 | 546.3584 | 16.91 | 5821 | 792.8690 | 7.86 |
| 779 | 186.2088 | 18.95 | 2460 | 425.7573 | 8.34 | 4141 | 546.3588 | 15.48 | 5822 | 792.8732 | 7.65 |
| 780 | 186.2090 | 18.90 | 2461 | 425.7574 | 7.16 | 4142 | 546.3597 | 15.96 | 5823 | 792.8763 | 7.52 |
| 781 | 186.2092 | 19.19 | 2462 | 425.7612 | 8.40 | 4143 | 546.3604 | 20.50 | 5824 | 792.8804 | 8.39 |
| 782 | 186.2097 | 20.94 | 2463 | 426.0057 | 8.40 | 4144 | 546.3618 | 15.68 | 5825 | 792.8839 | 7.50 |
| 783 | 186.2097 | 9.23 | 2464 | 426.0721 | 17.26 | 4145 | 546.6286 | 7.94 | 5826 | 792.8884 | 7.77 |
| 784 | 186.2108 | 7.88 | 2465 | 426.2064 | 6.95 | 4146 | 546.6299 | 9.41 | 5827 | 793.0832 | 9.35 |
| 785 | 186.2110 | 7.49 | 2466 | 426.3028 | 15.72 | 4147 | 546.6301 | 8.42 | 5828 | 793.0887 | 9.43 |
| 786 | 186.2113 | 20.89 | 2467 | 426.3280 | 16.36 | 4148 | 546.6303 | 8.18 | 5829 | 793.0905 | 7.74 |
| 787 | 186.2119 | 19.12 | 2468 | 427.0764 | 0.91 | 4149 | 546.6304 | 8.56 | 5830 | 793.0916 | 7.89 |
| 788 | 186.2129 | 17.67 | 2469 | 427.0812 | 0.94 | 4150 | 546.6305 | 7.82 | 5831 | 793.0931 | 7.76 |
| 789 | 186.2130 | 18.93 | 2470 | 427.3999 | 4.97 | 4151 | 546.6310 | 7.98 | 5832 | 793.0939 | 8.47 |
| 790 | 186.2132 | 19.00 | 2471 | 429.0965 | 0.87 | 4152 | 546.6315 | 7.95 | 5833 | 793.0943 | 8.71 |
| 791 | 186.2132 | 18.82 | 2472 | 429.2409 | 14.63 | 4153 | 546.6321 | 8.23 | 5834 | 793.0964 | 8.53 |
| 792 | 186.2135 | 19.13 | 2473 | 430.0031 | 8.22 | 4154 | 546.6325 | 9.87 | 5835 | 793.0994 | 9.51 |
| 793 | 186.2136 | 10.01 | 2474 | 430.0031 | 8.47 | 4155 | 546.6328 | 9.63 | 5836 | 793.1063 | 8.85 |
| 794 | 186.2142 | 8.57 | 2475 | 430.0055 | 7.56 | 4156 | 546.6336 | 9.16 | 5837 | 793.4181 | 8.53 |
| 795 | 186.2144 | 19.18 | 2476 | 430.0067 | 8.01 | 4157 | 546.6340 | 8.92 | 5838 | 793.4216 | 9.49 |
| 796 | 186.2145 | 1.04 | 2477 | 430.0091 | 8.24 | 4158 | 546.6354 | 8.22 | 5839 | 793.4218 | 9.27 |
| 797 | 186.2146 | 18.76 | 2478 | 430.0091 | 8.40 | 4159 | 546.6362 | 9.67 | 5840 | 793.4229 | 7.76 |
| 798 | 186.2148 | 18.86 | 2479 | 430.0111 | 8.34 | 4160 | 546.9634 | 8.18 | 5841 | 793.4242 | 7.74 |
| 799 | 186.2149 | 9.94 | 2480 | 430.0111 | 7.16 | 4161 | 546.9646 | 7.95 | 5842 | 793.4252 | 9.51 |
| 800 | 186.2150 | 18.80 | 2481 | 430.0113 | 8.33 | 4162 | 546.9654 | 9.64 | 5843 | 793.4253 | 7.89 |
| 801 | 186.2151 | 20.89 | 2482 | 430.0116 | 8.37 | 4163 | 546.9660 | 9.67 | 5844 | 793.4278 | 8.82 |
| 802 | 186.2162 | 10.10 | 2483 | 430.2435 | 14.64 | 4164 | 546.9663 | 9.41 | 5845 | 793.7357 | 9.27 |
| 803 | 186.2164 | 9.90 | 2484 | 430.2544 | 8.37 | 4165 | 546.9665 | 8.42 | 5846 | 793.7510 | 9.51 |
| 804 | 186.2165 | 10.22 | 2485 | 430.2585 | 8.49 | 4166 | 546.9676 | 8.00 | 5847 | 793.7610 | 8.84 |
| 805 | 186.2167 | 18.92 | 2486 | 430.2599 | 7.14 | 4167 | 546.9686 | 8.23 | 5848 | 793.7633 | 9.49 |
| 806 | 186.2168 | 18.24 | 2487 | 430.2617 | 8.24 | 4168 | 546.9688 | 9.88 | 5849 | 793.7689 | 8.84 |
| 807 | 186.2168 | 10.02 | 2488 | 430.2629 | 8.29 | 4169 | 546.9696 | 8.19 | 5850 | 793.8465 | 7.65 |
| 808 | 186.2169 | 17.57 | 2489 | 430.2630 | 7.56 | 4170 | 546.9696 | 8.55 | 5851 | 793.8660 | 7.62 |
| 809 | 186.2170 | 9.93 | 2490 | 430.2641 | 8.01 | 4171 | 546.9701 | 9.17 | 5852 | 793.8742 | 7.49 |
| 810 | 186.2170 | 19.07 | 2491 | 430.2656 | 8.34 | 4172 | 546.9704 | 7.80 | 5853 | 793.8946 | 10.81 |
| 811 | 186.2173 | 15.22 | 2492 | 430.2657 | 9.10 | 4173 | 546.9710 | 8.90 | 5854 | 793.9014 | 10.72 |
| 812 | 186.2182 | 19.03 | 2493 | 430.2686 | 9.44 | 4174 | 547.2572 | 11.81 | 5855 | 793.9084 | 11.06 |
| 813 | 186.2183 | 0.79 | 2494 | 430.2982 | 15.87 | 4175 | 547.2910 | 8.90 | 5856 | 794.1034 | 8.82 |
| 814 | 186.2184 | 6.52 | 2495 | 430.5055 | 8.34 | 4176 | 547.2966 | 8.18 | 5857 | 794.3997 | 10.82 |
| 815 | 186.2186 | 18.22 | 2496 | 430.5087 | 8.33 | 4177 | 547.2974 | 8.02 | 5858 | 794.4053 | 11.06 |
| 816 | 186.2186 | 0.93 | 2497 | 430.5093 | 8.24 | 4178 | 547.2978 | 7.95 | 5859 | 794.4188 | 8.87 |
| 817 | 186.2187 | 17.61 | 2498 | 430.5094 | 8.40 | 4179 | 547.2985 | 8.22 | 5860 | 794.8787 | 7.35 |
| 818 | 186.2189 | 19.04 | 2499 | 430.5097 | 8.46 | 4180 | 547.2995 | 8.19 | 5861 | 794.8880 | 7.06 |
| 819 | 186.2189 | 15.20 | 2500 | 430.5099 | 8.01 | 4181 | 547.3002 | 7.82 | 5862 | 794.8887 | 6.44 |
| 820 | 186.2193 | 17.62 | 2501 | 430.5114 | 7.16 | 4182 | 547.3004 | 8.92 | 5863 | 794.8890 | 4.98 |
| 821 | 186.2199 | 1.06 | 2502 | 430.5119 | 8.37 | 4183 | 547.3015 | 9.62 | 5864 | 794.8909 | 5.53 |
| 822 | 186.2203 | 0.86 | 2503 | 430.7357 | 10.88 | 4184 | 547.3025 | 9.71 | 5865 | 794.9043 | 10.81 |
| 823 | 186.2204 | 16.19 | 2504 | 430.7578 | 8.40 | 4185 | 547.3028 | 9.41 | 5866 | 795.8800 | 7.04 |
| 824 | 186.2205 | 15.20 | 2505 | 430.7631 | 8.34 | 4186 | 547.3030 | 8.42 | 5867 | 795.8875 | 6.45 |
| 825 | 186.2206 | 8.94 | 2506 | 430.8571 | 2.64 | 4187 | 547.3033 | 8.56 | 5868 | 795.8922 | 4.97 |
| 826 | 186.2207 | 9.06 | 2507 | 430.8636 | 2.61 | 4188 | 547.3059 | 9.67 | 5869 | 795.9004 | 5.52 |
| 827 | 186.2207 | 8.86 | 2508 | 430.8846 | 18.32 | 4189 | 547.3230 | 11.19 | 5870 | 796.8193 | 7.04 |
| 828 | 186.2210 | 21.24 | 2509 | 430.8890 | 15.06 | 4190 | 547.3506 | 17.16 | 5871 | 796.8218 | 6.35 |
| 829 | 186.2211 | 18.31 | 2510 | 430.8955 | 18.29 | 4191 | 547.3523 | 15.72 | 5872 | 796.8406 | 5.53 |
| 830 | 186.2217 | 1.11 | 2511 | 430.8960 | 15.33 | 4192 | 547.3537 | 18.77 | 5873 | 796.8420 | 5.00 |
| 831 | 186.2220 | 1.18 | 2512 | 430.9121 | 1.02 | 4193 | 547.3543 | 15.69 | 5874 | 797.3330 | 7.04 |
| 832 | 186.2220 | 1.26 | 2513 | 430.9137 | 0.78 | 4194 | 547.3544 | 17.00 | 5875 | 797.3335 | 5.01 |
| 833 | 186.2221 | 1.07 | 2514 | 430.9153 | 0.78 | 4195 | 547.3547 | 15.67 | 5876 | 797.3379 | 5.55 |
| 834 | 186.2221 | 0.88 | 2515 | 430.9156 | 0.86 | 4196 | 547.3569 | 18.76 | 5877 | 797.3405 | 6.45 |
| 835 | 186.2221 | 0.97 | 2516 | 431.1423 | 14.37 | 4197 | 547.3583 | 17.27 | 5878 | 797.4105 | 7.93 |
| 836 | 186.2221 | 0.66 | 2517 | 431.2790 | 16.36 | 4198 | 547.3585 | 17.15 | 5879 | 797.4141 | 9.11 |
| 837 | 186.2221 | 0.83 | 2518 | 431.3033 | 15.84 | 4199 | 547.3594 | 20.90 | 5880 | 797.4189 | 9.72 |
| 838 | 186.2222 | 9.19 | 2519 | 431.3053 | 15.87 | 4200 | 547.3604 | 17.05 | 5881 | 797.4197 | 9.37 |
| 839 | 186.2222 | 9.40 | 2520 | 431.3114 | 18.96 | 4201 | 547.3666 | 15.53 | 5882 | 797.4273 | 9.18 |
| 840 | 186.2222 | 10.85 | 2521 | 431.3202 | 18.98 | 4202 | 547.6303 | 7.80 | 5883 | 797.4284 | 9.47 |
| 841 | 186.2222 | 1.03 | 2522 | 431.9161 | 0.80 | 4203 | 547.6319 | 9.90 | 5884 | 797.4327 | 9.29 |
| 842 | 186.2223 | 0.29 | 2523 | 432.2752 | 9.07 | 4204 | 547.6326 | 9.67 | 5885 | 797.4354 | 9.27 |
| 843 | 186.2224 | 18.43 | 2524 | 432.2754 | 8.76 | 4205 | 547.6331 | 8.42 | 5886 | 797.4375 | 8.82 |
| 844 | 186.2225 | 19.10 | 2525 | 432.2768 | 10.09 | 4206 | 547.6349 | 9.62 | 5887 | 797.7411 | 9.74 |
| 845 | 186.2226 | 9.79 | 2526 | 432.2777 | 8.41 | 4207 | 547.6351 | 8.23 | 5888 | 797.7487 | 9.11 |
| 846 | 186.2226 | 9.61 | 2527 | 432.2780 | 9.01 | 4208 | 547.6394 | 9.41 | 5889 | 797.7513 | 8.47 |
| 847 | 186.2227 | 11.00 | 2528 | 432.2782 | 8.59 | 4209 | 547.6406 | 7.98 | 5890 | 797.7577 | 7.84 |
| 848 | 186.2236 | 1.15 | 2529 | 432.2794 | 10.21 | 4210 | 547.9660 | 9.67 | 5891 | 797.7602 | 8.81 |
| 849 | 186.2266 | 16.23 | 2530 | 432.2803 | 10.22 | 4211 | 548.0866 | 9.84 | 5892 | 797.7643 | 9.34 |
| 850 | 186.2300 | 18.99 | 2531 | 432.2812 | 8.68 | 4212 | 548.1037 | 10.17 | 5893 | 797.7650 | 8.19 |
| 851 | 186.2308 | 17.61 | 2532 | 432.2821 | 10.34 | 4213 | 548.2761 | 15.16 | 5894 | 797.7659 | 9.18 |
| 852 | 186.2313 | 19.30 | 2533 | 432.2854 | 8.53 | 4214 | 548.2817 | 18.26 | 5895 | 797.7772 | 9.37 |
| 853 | 186.2361 | 19.04 | 2534 | 432.3096 | 14.14 | 4215 | 548.2980 | 9.84 | 5896 | 797.9071 | 11.05 |
| 854 | 186.2397 | 10.19 | 2535 | 432.3115 | 16.40 | 4216 | 548.3456 | 17.23 | 5897 | 798.0846 | 9.18 |
| 855 | 186.2413 | 10.18 | 2536 | 432.3122 | 17.82 | 4217 | 548.3586 | 17.29 | 5898 | 798.0894 | 9.39 |
| 856 | 187.0582 | 1.03 | 2537 | 432.3128 | 17.44 | 4218 | 548.3606 | 20.87 | 5899 | 798.0940 | 9.29 |
| 857 | 187.0585 | 1.16 | 2538 | 432.3129 | 17.40 | 4219 | 548.3607 | 17.14 | 5900 | 798.0990 | 9.34 |
| 858 | 187.0590 | 1.13 | 2539 | 432.3140 | 15.82 | 4220 | 548.3652 | 10.93 | 5901 | 798.1001 | 9.72 |
| 859 | 187.0796 | 0.68 | 2540 | 432.7040 | 11.20 | 4221 | 548.3774 | 16.46 | 5902 | 798.1108 | 8.81 |
| 860 | 187.1223 | 16.32 | 2541 | 432.7622 | 10.91 | 4222 | 548.4935 | 10.17 | 5903 | 798.4114 | 9.18 |
| 861 | 187.1243 | 16.30 | 2542 | 432.8866 | 18.32 | 4223 | 548.8392 | 0.65 | 5904 | 798.4132 | 11.05 |
| 862 | 187.1251 | 12.64 | 2543 | 432.9246 | 0.83 | 4224 | 549.2827 | 18.22 | 5905 | 798.4216 | 8.82 |
| 863 | 187.1256 | 16.28 | 2544 | 433.2828 | 10.23 | 4225 | 549.3027 | 7.89 | 5906 | 798.4396 | 9.37 |
| 864 | 187.1259 | 12.43 | 2545 | 433.3089 | 15.80 | 4226 | 549.3046 | 9.01 | 5907 | 798.4403 | 9.38 |
| 865 | 187.1259 | 12.44 | 2546 | 433.3164 | 17.44 | 4227 | 549.3055 | 8.82 | 5908 | 798.4424 | 9.74 |
| 866 | 187.1260 | 15.24 | 2547 | 434.1239 | 1.04 | 4228 | 549.3087 | 9.06 | 5909 | 798.7564 | 8.82 |
| 867 | 187.1260 | 16.38 | 2548 | 434.3125 | 17.43 | 4229 | 549.3089 | 11.09 | 5910 | 798.7657 | 9.72 |
| 868 | 187.1261 | 12.38 | 2549 | 434.3194 | 15.80 | 4230 | 549.3095 | 9.05 | 5911 | 798.7734 | 9.37 |
| 869 | 187.1263 | 15.27 | 2550 | 435.1637 | 12.56 | 4231 | 549.6396 | 11.11 | 5912 | 798.9124 | 10.68 |
| 870 | 187.1274 | 12.55 | 2551 | 435.1660 | 12.40 | 4232 | 549.7919 | 9.89 | 5913 | 799.4148 | 10.71 |
| 871 | 187.1277 | 12.54 | 2552 | 435.1735 | 12.83 | 4233 | 549.7921 | 9.87 | 5914 | 799.8396 | 6.45 |
| 872 | 187.1281 | 16.29 | 2553 | 436.2354 | 17.87 | 4234 | 549.9735 | 11.09 | 5915 | 799.8440 | 5.51 |
| 873 | 188.0686 | 7.07 | 2554 | 436.9165 | 11.25 | 4235 | 549.9768 | 10.08 | 5916 | 799.8442 | 4.97 |
| 874 | 188.0692 | 6.67 | 2555 | 437.1444 | 15.64 | 4236 | 549.9770 | 9.84 | 5917 | 800.8451 | 5.58 |
| 875 | 188.0697 | 7.33 | 2556 | 437.1487 | 20.58 | 4237 | 550.1888 | 9.90 | 5918 | 800.8494 | 5.04 |
| 876 | 188.0707 | 6.55 | 2557 | 437.1726 | 12.55 | 4238 | 550.2910 | 8.82 | 5919 | 800.8501 | 6.38 |
| 877 | 188.0708 | 7.15 | 2558 | 437.2343 | 10.19 | 4239 | 550.3054 | 9.08 | 5920 | 801.8318 | 0.74 |
| 878 | 188.0714 | 6.81 | 2559 | 437.2405 | 10.23 | 4240 | 550.3150 | 19.32 | 5921 | 802.4031 | 10.97 |
| 879 | 188.0721 | 6.86 | 2560 | 437.2405 | 17.86 | 4241 | 550.3229 | 11.56 | 5922 | 802.7981 | 0.84 |
| 880 | 188.0724 | 6.62 | 2561 | 437.2421 | 10.01 | 4242 | 550.3873 | 9.91 | 5923 | 802.8050 | 0.79 |
| 881 | 188.0727 | 7.10 | 2562 | 437.2438 | 11.25 | 4243 | 551.4927 | 9.86 | 5924 | 803.4250 | 11.04 |
| 882 | 188.0899 | 0.82 | 2563 | 437.2659 | 9.30 | 4244 | 551.4928 | 8.38 | 5925 | 804.2903 | 4.94 |
| 883 | 188.1275 | 12.81 | 2564 | 437.2682 | 7.83 | 4245 | 551.4957 | 8.04 | 5926 | 804.3263 | 5.52 |
| 884 | 188.1285 | 12.54 | 2565 | 437.2700 | 9.20 | 4246 | 551.4964 | 9.90 | 5927 | 804.3299 | 7.04 |
| 885 | 188.1290 | 12.29 | 2566 | 437.2706 | 9.34 | 4247 | 551.4965 | 8.18 | 5928 | 804.3304 | 6.38 |
| 886 | 188.1291 | 15.18 | 2567 | 437.2715 | 8.13 | 4248 | 551.4980 | 8.69 | 5929 | 804.8105 | 6.38 |
| 887 | 188.1297 | 12.64 | 2568 | 437.2716 | 8.92 | 4249 | 551.4993 | 8.94 | 5930 | 804.8227 | 7.00 |
| 888 | 188.1301 | 12.56 | 2569 | 437.2717 | 10.53 | 4250 | 551.6917 | 9.84 | 5931 | 804.8247 | 5.00 |
| 889 | 188.1304 | 12.38 | 2570 | 437.2718 | 9.31 | 4251 | 551.6918 | 9.90 | 5932 | 804.8317 | 0.82 |
| 890 | 188.1306 | 12.44 | 2571 | 437.2719 | 8.30 | 4252 | 551.6947 | 8.94 | 5933 | 804.9216 | 10.96 |
| 891 | 188.1307 | 15.22 | 2572 | 437.2721 | 8.90 | 4253 | 551.6949 | 8.38 | 5934 | 805.0738 | 9.39 |
| 892 | 188.9183 | 21.62 | 2573 | 437.2722 | 8.94 | 4254 | 551.6972 | 8.48 | 5935 | 805.3422 | 0.84 |
| 893 | 189.0702 | 7.15 | 2574 | 437.2731 | 9.18 | 4255 | 551.6986 | 8.16 | 5936 | 806.4469 | 12.07 |
| 894 | 189.0723 | 7.23 | 2575 | 437.2732 | 8.67 | 4256 | 551.6986 | 8.18 | 5937 | 808.8988 | 7.79 |
| 895 | 189.0735 | 6.86 | 2576 | 437.2738 | 8.19 | 4257 | 551.7010 | 8.04 | 5938 | 808.9107 | 7.53 |
| 896 | 189.0738 | 7.07 | 2577 | 437.2744 | 8.53 | 4258 | 551.7095 | 8.70 | 5939 | 809.4534 | 9.85 |
| 897 | 189.0739 | 6.54 | 2578 | 437.2744 | 8.50 | 4259 | 551.8903 | 8.38 | 5940 | 809.4546 | 12.04 |
| 898 | 189.0744 | 6.67 | 2579 | 437.2744 | 7.62 | 4260 | 551.8935 | 8.94 | 5941 | 809.4561 | 10.32 |
| 899 | 189.0744 | 1.48 | 2580 | 437.2745 | 8.37 | 4261 | 551.8975 | 8.43 | 5942 | 809.4607 | 9.94 |
| 900 | 189.0751 | 7.17 | 2581 | 437.2747 | 8.77 | 4262 | 551.8984 | 8.70 | 5943 | 809.9064 | 7.59 |
| 901 | 189.0776 | 7.09 | 2582 | 437.2748 | 8.45 | 4263 | 551.9002 | 9.86 | 5944 | 809.9564 | 12.05 |
| 902 | 189.0970 | 0.66 | 2583 | 437.2773 | 10.55 | 4264 | 551.9005 | 9.90 | 5945 | 809.9627 | 9.95 |
| 903 | 189.1199 | 12.39 | 2584 | 437.2776 | 7.92 | 4265 | 551.9007 | 8.16 | 5946 | 809.9631 | 9.85 |
| 904 | 189.1204 | 12.44 | 2585 | 437.2778 | 7.30 | 4266 | 551.9007 | 8.18 | 5947 | 809.9643 | 12.03 |
| 905 | 189.1208 | 12.29 | 2586 | 437.2780 | 8.44 | 4267 | 551.9031 | 8.04 | 5948 | 809.9658 | 10.32 |
| 906 | 189.1229 | 15.16 | 2587 | 437.2783 | 8.62 | 4268 | 552.0930 | 8.41 | 5949 | 810.4583 | 12.05 |
| 907 | 189.1234 | 12.80 | 2588 | 437.2785 | 9.87 | 4269 | 552.0958 | 8.38 | 5950 | 810.4636 | 10.30 |
| 908 | 189.1257 | 12.55 | 2589 | 437.2809 | 7.29 | 4270 | 552.0975 | 8.72 | 5951 | 810.8523 | 7.50 |
| 909 | 190.0843 | 14.01 | 2590 | 437.7759 | 8.50 | 4271 | 552.0987 | 8.04 | 5952 | 810.9599 | 12.07 |
| 910 | 190.0865 | 14.04 | 2591 | 437.7783 | 8.19 | 4272 | 552.0990 | 8.94 | 5953 | 811.3516 | 7.49 |
| 911 | 190.0972 | 0.66 | 2592 | 437.7789 | 7.62 | 4273 | 552.0992 | 9.84 | 5954 | 811.4537 | 10.79 |
| 912 | 191.0326 | 0.66 | 2593 | 437.7795 | 7.29 | 4274 | 552.0995 | 8.18 | 5955 | 811.8574 | 7.46 |
| 913 | 191.0402 | 0.90 | 2594 | 437.7796 | 7.32 | 4275 | 552.1027 | 9.90 | 5956 | 812.8322 | 0.83 |
| 914 | 191.0414 | 0.88 | 2595 | 437.7817 | 8.40 | 4276 | 552.1028 | 8.16 | 5957 | 812.8327 | 0.78 |
| 915 | 191.0418 | 0.91 | 2596 | 438.2318 | 10.26 | 4277 | 552.2103 | 17.07 | 5958 | 813.3491 | 0.84 |
| 916 | 191.0446 | 1.03 | 2597 | 438.2321 | 17.86 | 4278 | 552.2810 | 8.04 | 5959 | 813.8552 | 7.50 |
| 917 | 191.0453 | 0.86 | 2598 | 438.2692 | 9.31 | 4279 | 552.2914 | 8.53 | 5960 | 813.8596 | 7.52 |
| 918 | 191.0454 | 0.89 | 2599 | 438.2748 | 8.50 | 4280 | 552.2948 | 9.84 | 5961 | 813.8683 | 7.76 |
| 919 | 191.0736 | 0.82 | 2600 | 438.2806 | 10.55 | 4281 | 552.2982 | 9.90 | 5962 | 814.8736 | 7.50 |
| 920 | 192.0239 | 0.98 | 2601 | 440.3160 | 17.24 | 4282 | 552.2984 | 8.18 | 5963 | 817.7412 | 8.23 |
| 921 | 192.1583 | 1.10 | 2602 | 440.8676 | 0.75 | 4283 | 552.2987 | 8.39 | 5964 | 817.7491 | 8.07 |
| 922 | 193.1340 | 13.12 | 2603 | 441.1673 | 16.59 | 4284 | 552.2991 | 5.98 | 5965 | 818.0680 | 8.23 |
| 923 | 194.1152 | 5.82 | 2604 | 441.2056 | 1.64 | 4285 | 552.3049 | 8.96 | 5966 | 818.0759 | 8.07 |
| 924 | 194.1152 | 5.05 | 2605 | 443.2163 | 8.22 | 4286 | 552.3060 | 8.70 | 5967 | 818.3321 | 7.52 |
| 925 | 194.1155 | 5.45 | 2606 | 443.2165 | 8.29 | 4287 | 552.4940 | 9.87 | 5968 | 818.4028 | 8.23 |
| 926 | 194.1158 | 2.85 | 2607 | 443.2173 | 8.37 | 4288 | 552.4997 | 8.04 | 5969 | 818.4067 | 8.07 |
| 927 | 194.1160 | 4.93 | 2608 | 443.2181 | 8.46 | 4289 | 552.5002 | 10.08 | 5970 | 818.7297 | 8.23 |
| 928 | 194.1162 | 5.57 | 2609 | 443.3258 | 10.91 | 4290 | 552.5004 | 9.91 | 5971 | 818.7457 | 8.07 |
| 929 | 194.1163 | 2.42 | 2610 | 443.3263 | 10.97 | 4291 | 552.5033 | 8.94 | 5972 | 818.8491 | 7.62 |
| 930 | 194.1168 | 5.30 | 2611 | 443.4170 | 8.44 | 4292 | 552.5068 | 8.17 | 5973 | 819.4405 | 9.57 |
| 931 | 194.1168 | 5.39 | 2612 | 443.4212 | 8.31 | 4293 | 552.5131 | 9.81 | 5974 | 819.4411 | 8.90 |
| 932 | 194.1169 | 4.52 | 2613 | 443.6161 | 8.46 | 4294 | 552.6323 | 8.54 | 5975 | 819.4413 | 7.80 |
| 933 | 194.1170 | 5.85 | 2614 | 443.6242 | 8.37 | 4295 | 552.6923 | 8.94 | 5976 | 819.4417 | 8.42 |
| 934 | 194.1174 | 5.81 | 2615 | 443.8270 | 8.46 | 4296 | 552.6929 | 9.87 | 5977 | 819.4422 | 9.62 |
| 935 | 194.1185 | 4.79 | 2616 | 444.2806 | 10.01 | 4297 | 552.6965 | 8.17 | 5978 | 819.4430 | 7.97 |
| 936 | 194.1187 | 6.13 | 2617 | 444.3276 | 10.97 | 4298 | 552.7060 | 9.91 | 5979 | 819.4437 | 8.02 |
| 937 | 194.1188 | 5.25 | 2618 | 445.0841 | 0.99 | 4299 | 552.7089 | 10.01 | 5980 | 819.4446 | 8.22 |
| 938 | 194.1188 | 4.99 | 2619 | 445.0866 | 0.94 | 4300 | 552.9118 | 9.87 | 5981 | 819.4467 | 7.95 |
| 939 | 194.1189 | 5.66 | 2620 | 445.1164 | 17.22 | 4301 | 553.3124 | 9.04 | 5982 | 819.4467 | 9.67 |
| 940 | 194.1189 | 3.41 | 2621 | 445.1214 | 19.04 | 4302 | 553.3141 | 9.15 | 5983 | 819.4508 | 9.41 |
| 941 | 194.1189 | 5.11 | 2622 | 445.2570 | 7.40 | 4303 | 553.6421 | 9.12 | 5984 | 819.4553 | 9.88 |
| 942 | 194.1189 | 5.36 | 2623 | 445.2607 | 9.64 | 4304 | 553.6459 | 9.15 | 5985 | 819.8338 | 7.48 |
| 943 | 194.1193 | 4.70 | 2624 | 445.2654 | 8.21 | 4305 | 553.6478 | 9.02 | 5986 | 819.9453 | 8.22 |
| 944 | 194.1196 | 5.70 | 2625 | 445.2813 | 17.24 | 4306 | 553.9730 | 9.02 | 5987 | 819.9455 | 8.95 |
| 945 | 194.1198 | 4.84 | 2626 | 445.2951 | 10.17 | 4307 | 553.9773 | 9.12 | 5988 | 819.9458 | 7.82 |
| 946 | 194.1202 | 4.91 | 2627 | 445.3031 | 10.02 | 4308 | 553.9845 | 9.15 | 5989 | 819.9463 | 9.65 |
| 947 | 195.0888 | 9.10 | 2628 | 445.8258 | 2.70 | 4309 | 554.2958 | 6.53 | 5990 | 819.9465 | 8.42 |
| 948 | 195.1243 | 6.60 | 2629 | 445.8318 | 2.73 | 4310 | 554.3198 | 9.15 | 5991 | 819.9469 | 9.62 |
| 949 | 196.0767 | 13.80 | 2630 | 446.2360 | 10.49 | 4311 | 554.8558 | 0.70 | 5992 | 819.9474 | 7.95 |
| 950 | 196.1179 | 0.83 | 2631 | 446.2415 | 10.47 | 4312 | 554.8596 | 0.70 | 5993 | 819.9479 | 9.88 |
| 951 | 197.1186 | 0.83 | 2632 | 446.2449 | 10.29 | 4313 | 555.2797 | 8.30 | 5994 | 819.9493 | 9.57 |
| 952 | 198.0798 | 0.98 | 2633 | 446.6198 | 8.28 | 4314 | 555.2867 | 10.10 | 5995 | 819.9544 | 8.18 |
| 953 | 198.9380 | 0.65 | 2634 | 446.6220 | 8.12 | 4315 | 555.2872 | 11.56 | 5996 | 819.9555 | 9.41 |
| 954 | 198.9411 | 21.69 | 2635 | 446.6222 | 8.19 | 4316 | 555.2882 | 8.06 | 5997 | 819.9555 | 9.67 |
| 955 | 199.1689 | 17.82 | 2636 | 446.6227 | 8.22 | 4317 | 555.2889 | 8.96 | 5998 | 820.4308 | 7.80 |
| 956 | 200.0443 | 0.91 | 2637 | 446.6229 | 8.29 | 4318 | 555.2929 | 10.17 | 5999 | 820.4361 | 11.99 |
| 957 | 200.0444 | 1.02 | 2638 | 446.6236 | 7.56 | 4319 | 555.2944 | 8.44 | 6000 | 820.4483 | 9.67 |
| 958 | 200.1966 | 14.58 | 2639 | 446.6244 | 8.44 | 4320 | 555.2991 | 6.53 | 6001 | 820.4516 | 7.94 |
| 959 | 200.1983 | 14.74 | 2640 | 446.6253 | 8.21 | 4321 | 555.4496 | 10.10 | 6002 | 820.4564 | 9.41 |
| 960 | 200.2006 | 14.53 | 2641 | 446.6270 | 8.47 | 4322 | 555.4542 | 8.46 | 6003 | 820.4569 | 9.88 |
| 961 | 200.2011 | 14.68 | 2642 | 446.6275 | 8.46 | 4323 | 555.4563 | 10.19 | 6004 | 820.8234 | 0.76 |
| 962 | 200.2016 | 14.71 | 2643 | 446.6278 | 7.90 | 4324 | 555.4592 | 8.30 | 6005 | 820.9413 | 9.67 |
| 963 | 200.2031 | 17.83 | 2644 | 446.8197 | 8.29 | 4325 | 555.4594 | 8.28 | 6006 | 821.8959 | 6.23 |
| 964 | 200.5402 | 1.01 | 2645 | 446.8215 | 7.90 | 4326 | 555.4607 | 8.05 | 6007 | 822.9052 | 5.56 |
| 965 | 200.9367 | 0.69 | 2646 | 446.8220 | 8.19 | 4327 | 555.4617 | 8.96 | 6008 | 822.9196 | 4.98 |
| 966 | 201.2035 | 17.84 | 2647 | 446.8242 | 8.44 | 4328 | 555.4629 | 10.22 | 6009 | 822.9236 | 6.51 |
| 967 | 202.1786 | 1.51 | 2648 | 446.8250 | 8.24 | 4329 | 555.4656 | 10.14 | 6010 | 823.4546 | 11.08 |
| 968 | 202.1800 | 1.41 | 2649 | 446.8263 | 7.56 | 4330 | 555.6219 | 10.17 | 6011 | 823.4625 | 11.09 |
| 969 | 202.4261 | 1.00 | 2650 | 446.8272 | 8.46 | 4331 | 555.6223 | 8.28 | 6012 | 823.9202 | 6.49 |
| 970 | 203.0527 | 1.39 | 2651 | 446.8277 | 8.12 | 4332 | 555.6225 | 10.19 | 6013 | 823.9284 | 7.11 |
| 971 | 203.0531 | 0.97 | 2652 | 446.8290 | 8.14 | 4333 | 555.6258 | 10.10 | 6014 | 823.9307 | 5.00 |
| 972 | 203.0531 | 1.31 | 2653 | 446.8873 | 0.76 | 4334 | 555.6339 | 8.06 | 6015 | 823.9605 | 11.11 |
| 973 | 203.1091 | 19.28 | 2654 | 447.0209 | 8.16 | 4335 | 555.6341 | 8.94 | 6016 | 824.4625 | 11.09 |
| 974 | 203.1380 | 2.14 | 2655 | 447.0213 | 7.90 | 4336 | 555.7870 | 8.94 | 6017 | 824.9274 | 6.44 |
| 975 | 203.1384 | 2.45 | 2656 | 447.0245 | 8.12 | 4337 | 555.7921 | 10.13 | 6018 | 824.9317 | 4.98 |
| 976 | 203.1386 | 9.31 | 2657 | 447.0247 | 8.19 | 4338 | 555.7933 | 8.49 | 6019 | 825.4547 | 11.74 |
| 977 | 203.1392 | 2.65 | 2658 | 447.0252 | 8.22 | 4339 | 555.7954 | 10.10 | 6020 | 826.4600 | 11.74 |
| 978 | 203.1400 | 2.15 | 2659 | 447.0254 | 8.08 | 4340 | 555.7985 | 8.28 | 6021 | 826.7961 | 0.75 |
| 979 | 203.1481 | 0.90 | 2660 | 447.0254 | 8.29 | 4341 | 555.9578 | 10.17 | 6022 | 826.9303 | 8.53 |
| 980 | 203.1488 | 0.86 | 2661 | 447.0265 | 8.47 | 4342 | 555.9650 | 10.13 | 6023 | 827.4252 | 8.53 |
| 981 | 203.1506 | 0.88 | 2662 | 447.0282 | 8.28 | 4343 | 555.9672 | 8.97 | 6024 | 828.8011 | 0.81 |
| 982 | 204.0552 | 0.97 | 2663 | 447.0329 | 8.46 | 4344 | 556.1209 | 10.20 | 6025 | 829.4674 | 9.12 |
| 983 | 204.0570 | 1.01 | 2664 | 447.2149 | 8.46 | 4345 | 556.1296 | 8.94 | 6026 | 829.4682 | 9.15 |
| 984 | 204.1213 | 1.24 | 2665 | 447.2153 | 13.18 | 4346 | 557.0970 | 7.13 | 6027 | 829.9623 | 9.04 |
| 985 | 204.1224 | 1.22 | 2666 | 447.2161 | 8.22 | 4347 | 558.0240 | 8.47 | 6028 | 829.9679 | 9.15 |
| 986 | 204.1251 | 1.25 | 2667 | 447.2227 | 8.14 | 4348 | 558.0253 | 8.12 | 6029 | 830.4367 | 9.72 |
| 987 | 204.1385 | 18.96 | 2668 | 447.2271 | 7.90 | 4349 | 558.0258 | 8.21 | 6030 | 830.4438 | 10.19 |
| 988 | 205.0665 | 0.99 | 2669 | 447.2282 | 8.29 | 4350 | 558.0262 | 8.31 | 6031 | 830.4440 | 8.11 |
| 989 | 205.0768 | 0.66 | 2670 | 447.2297 | 8.44 | 4351 | 558.0263 | 8.29 | 6032 | 830.4511 | 10.06 |
| 990 | 205.0850 | 19.68 | 2671 | 447.4177 | 8.46 | 4352 | 558.0265 | 7.91 | 6033 | 830.4540 | 10.04 |
| 991 | 205.0850 | 19.69 | 2672 | 447.4206 | 8.44 | 4353 | 558.0276 | 7.54 | 6034 | 830.4549 | 10.14 |
| 992 | 205.0855 | 19.69 | 2673 | 447.4311 | 8.29 | 4354 | 558.0293 | 8.28 | 6035 | 830.4637 | 9.15 |
| 993 | 205.0857 | 16.25 | 2674 | 448.2876 | 10.97 | 4355 | 558.0296 | 8.22 | 6036 | 830.7589 | 10.09 |
| 994 | 205.0866 | 16.52 | 2675 | 448.3067 | 15.86 | 4356 | 558.0303 | 7.90 | 6037 | 830.7680 | 6.73 |
| 995 | 205.0870 | 19.73 | 2676 | 448.3612 | 19.39 | 4357 | 558.0309 | 8.46 | 6038 | 830.7706 | 6.35 |
| 996 | 205.0883 | 16.40 | 2677 | 448.8822 | 0.81 | 4358 | 558.0995 | 7.13 | 6039 | 830.7741 | 9.72 |
| 997 | 205.0883 | 16.41 | 2678 | 448.9544 | 6.49 | 4359 | 558.2703 | 7.90 | 6040 | 830.7774 | 8.50 |
| 998 | 205.0959 | 6.67 | 2679 | 449.3136 | 15.86 | 4360 | 558.2726 | 8.28 | 6041 | 830.7777 | 9.83 |
| 999 | 205.0960 | 6.81 | 2680 | 449.3843 | 16.23 | 4361 | 558.2729 | 8.29 | 6042 | 830.7807 | 8.31 |
| 1000 | 205.0964 | 7.17 | 2681 | 449.3851 | 13.13 | 4362 | 558.2739 | 8.47 | 6043 | 830.7821 | 10.15 |
| 1001 | 205.0970 | 6.62 | 2682 | 449.3898 | 13.54 | 4363 | 558.2761 | 8.08 | 6044 | 830.7897 | 8.06 |
| 1002 | 205.0973 | 6.55 | 2683 | 449.3900 | 13.20 | 4364 | 558.2762 | 8.22 | 6045 | 830.7950 | 9.93 |
| 1003 | 205.0983 | 7.33 | 2684 | 449.3916 | 13.38 | 4365 | 558.2799 | 8.14 | 6046 | 830.7954 | 10.04 |
| 1004 | 205.0988 | 6.86 | 2685 | 450.3222 | 15.80 | 4366 | 558.2808 | 7.54 | 6047 | 831.1029 | 9.83 |
| 1005 | 205.0992 | 7.07 | 2686 | 450.3236 | 17.85 | 4367 | 558.2809 | 8.46 | 6048 | 831.1055 | 10.11 |
| 1006 | 205.1268 | 1.30 | 2687 | 450.3240 | 17.43 | 4368 | 558.2883 | 15.30 | 6049 | 831.1075 | 9.72 |
| 1007 | 205.1278 | 1.25 | 2688 | 450.3832 | 13.20 | 4369 | 558.2931 | 16.81 | 6050 | 831.1150 | 8.06 |
| 1008 | 205.9864 | 1.93 | 2689 | 450.3865 | 16.23 | 4370 | 558.2953 | 15.13 | 6051 | 831.1151 | 10.18 |
| 1009 | 205.9868 | 1.75 | 2690 | 450.3870 | 16.26 | 4371 | 558.2978 | 15.12 | 6052 | 831.1162 | 9.93 |
| 1010 | 205.9874 | 1.85 | 2691 | 450.3956 | 13.39 | 4372 | 558.3038 | 14.98 | 6053 | 831.1247 | 10.04 |
| 1011 | 205.9883 | 1.82 | 2692 | 451.2119 | 14.66 | 4373 | 558.5175 | 7.54 | 6054 | 831.1431 | 10.19 |
| 1012 | 206.0694 | 0.97 | 2693 | 451.3183 | 17.43 | 4374 | 558.5272 | 8.47 | 6055 | 831.2531 | 12.37 |
| 1013 | 207.0117 | 0.90 | 2694 | 451.3254 | 15.80 | 4375 | 558.5290 | 8.21 | 6056 | 831.4495 | 10.48 |
| 1014 | 208.0544 | 0.95 | 2695 | 452.2840 | 17.84 | 4376 | 558.5296 | 8.29 | 6057 | 831.4523 | 12.36 |
| 1015 | 209.0586 | 0.95 | 2696 | 452.7336 | 10.34 | 4377 | 558.5299 | 8.14 | 6058 | 831.4591 | 10.14 |
| 1016 | 209.1185 | 16.96 | 2697 | 452.7433 | 10.18 | 4378 | 558.5302 | 7.90 | 6059 | 831.4604 | 8.08 |
| 1017 | 209.1557 | 17.73 | 2698 | 453.2326 | 10.18 | 4379 | 558.5330 | 7.91 | 6060 | 831.4623 | 10.04 |
| 1018 | 209.2004 | 8.97 | 2699 | 453.2353 | 7.36 | 4380 | 558.5342 | 8.46 | 6061 | 831.6475 | 12.39 |
| 1019 | 209.2004 | 8.95 | 2700 | 453.2371 | 7.14 | 4381 | 558.7695 | 8.22 | 6062 | 831.6513 | 10.21 |
| 1020 | 209.2007 | 9.41 | 2701 | 453.2432 | 7.99 | 4382 | 558.7709 | 8.46 | 6063 | 831.7789 | 10.16 |
| 1021 | 209.2015 | 10.95 | 2702 | 453.3206 | 19.43 | 4383 | 558.7729 | 8.29 | 6064 | 831.8550 | 12.39 |
| 1022 | 209.2016 | 9.28 | 2703 | 453.7782 | 8.59 | 4384 | 558.7794 | 8.08 | 6065 | 832.4453 | 10.22 |
| 1023 | 209.2022 | 9.63 | 2704 | 454.0192 | 8.61 | 4385 | 558.7802 | 7.90 | 6066 | 832.6769 | 10.17 |
| 1024 | 209.2023 | 9.04 | 2705 | 454.0225 | 8.68 | 4386 | 559.0149 | 0.88 | 6067 | 832.6944 | 8.96 |
| 1025 | 209.2027 | 11.02 | 2706 | 454.2741 | 8.59 | 4387 | 559.0172 | 8.37 | 6068 | 832.8183 | 0.79 |
| 1026 | 209.2027 | 8.93 | 2707 | 454.2848 | 15.72 | 4388 | 559.0230 | 8.29 | 6069 | 832.8213 | 0.75 |
| 1027 | 209.2036 | 9.17 | 2708 | 454.2874 | 19.04 | 4389 | 559.0276 | 8.46 | 6070 | 832.9292 | 10.17 |
| 1028 | 210.0802 | 1.12 | 2709 | 454.2907 | 19.01 | 4390 | 559.0877 | 9.87 | 6071 | 832.9304 | 10.12 |
| 1029 | 210.0902 | 11.82 | 2710 | 454.2945 | 15.66 | 4391 | 559.0941 | 10.11 | 6072 | 832.9345 | 8.96 |
| 1030 | 210.1315 | 0.88 | 2711 | 454.2945 | 15.59 | 4392 | 559.0995 | 7.13 | 6073 | 833.1738 | 10.16 |
| 1031 | 210.1357 | 0.81 | 2712 | 454.2960 | 19.07 | 4393 | 559.1426 | 5.40 | 6074 | 833.1789 | 8.94 |
| 1032 | 210.2035 | 8.93 | 2713 | 454.2964 | 18.64 | 4394 | 559.1477 | 5.13 | 6075 | 833.4460 | 10.14 |
| 1033 | 210.2055 | 11.02 | 2714 | 454.2981 | 15.49 | 4395 | 559.2750 | 9.68 | 6076 | 833.4801 | 9.42 |
| 1034 | 211.0760 | 13.36 | 2715 | 454.2987 | 15.74 | 4396 | 559.2808 | 9.84 | 6077 | 833.4825 | 8.90 |
| 1035 | 212.0730 | 13.36 | 2716 | 455.2217 | 12.41 | 4397 | 559.2842 | 9.90 | 6078 | 833.5031 | 7.80 |
| 1036 | 213.1599 | 10.08 | 2717 | 455.2256 | 15.33 | 4398 | 559.2881 | 8.39 | 6079 | 834.4872 | 9.42 |
| 1037 | 213.1612 | 10.02 | 2718 | 455.2910 | 19.02 | 4399 | 559.2887 | 10.12 | 6080 | 835.1033 | 10.04 |
| 1038 | 213.1613 | 9.80 | 2719 | 455.3016 | 15.73 | 4400 | 559.3085 | 15.15 | 6081 | 835.1064 | 10.00 |
| 1039 | 214.0071 | 0.97 | 2720 | 455.3025 | 15.50 | 4401 | 559.4876 | 9.84 | 6082 | 835.1099 | 8.21 |
| 1040 | 214.9146 | 0.65 | 2721 | 456.0618 | 13.26 | 4402 | 559.4911 | 9.90 | 6083 | 835.1145 | 9.99 |
| 1041 | 214.9199 | 21.69 | 2722 | 456.2501 | 7.36 | 4403 | 559.6093 | 9.67 | 6084 | 835.1152 | 9.83 |
| 1042 | 215.0304 | 9.82 | 2723 | 456.7560 | 9.87 | 4404 | 560.1009 | 7.15 | 6085 | 835.1154 | 10.18 |
| 1043 | 215.1227 | 0.70 | 2724 | 456.9095 | 9.86 | 4405 | 560.1436 | 5.40 | 6086 | 835.1162 | 8.49 |
| 1044 | 215.1244 | 8.50 | 2725 | 457.0008 | 9.84 | 4406 | 560.3000 | 15.13 | 6087 | 835.1197 | 9.94 |
| 1045 | 215.1252 | 8.27 | 2726 | 457.0654 | 13.25 | 4407 | 560.3098 | 16.25 | 6088 | 835.1261 | 8.94 |
| 1046 | 215.1255 | 8.70 | 2727 | 457.0786 | 9.87 | 4408 | 560.8718 | 0.73 | 6089 | 835.1264 | 8.17 |
| 1047 | 217.0677 | 1.43 | 2728 | 457.2481 | 9.84 | 4409 | 561.1616 | 0.95 | 6090 | 835.4481 | 8.94 |
| 1048 | 217.0960 | 8.44 | 2729 | 457.2672 | 5.53 | 4410 | 561.2806 | 10.58 | 6091 | 835.4484 | 8.17 |
| 1049 | 217.0969 | 8.25 | 2730 | 457.2750 | 5.13 | 4411 | 561.2833 | 8.65 | 6092 | 835.4523 | 10.01 |
| 1050 | 217.0974 | 8.29 | 2731 | 457.2753 | 4.88 | 4412 | 561.6148 | 10.58 | 6093 | 835.4553 | 8.51 |
| 1051 | 217.0981 | 7.90 | 2732 | 457.2764 | 5.99 | 4413 | 561.6149 | 8.64 | 6094 | 835.4561 | 8.20 |
| 1052 | 217.0982 | 7.78 | 2733 | 457.2786 | 5.27 | 4414 | 561.8094 | 10.94 | 6095 | 835.4562 | 10.05 |
| 1053 | 217.0982 | 8.46 | 2734 | 457.2787 | 5.25 | 4415 | 561.9524 | 10.58 | 6096 | 835.4576 | 9.83 |
| 1054 | 217.0988 | 7.62 | 2735 | 457.2797 | 5.00 | 4416 | 562.0798 | 8.18 | 6097 | 835.4579 | 10.04 |
| 1055 | 217.1034 | 6.53 | 2736 | 457.2798 | 5.47 | 4417 | 562.0831 | 9.87 | 6098 | 835.4603 | 10.04 |
| 1056 | 217.1036 | 6.72 | 2737 | 457.4984 | 9.84 | 4418 | 562.0886 | 10.10 | 6099 | 835.4659 | 10.18 |
| 1057 | 217.1038 | 6.73 | 2738 | 457.7775 | 5.00 | 4419 | 562.1017 | 7.13 | 6100 | 835.4692 | 8.04 |
| 1058 | 217.1044 | 6.45 | 2739 | 457.7807 | 6.01 | 4420 | 562.2704 | 8.18 | 6101 | 835.4730 | 9.88 |
| 1059 | 217.1050 | 6.32 | 2740 | 457.7863 | 4.99 | 4421 | 562.2832 | 9.86 | 6102 | 835.4733 | 10.00 |
| 1060 | 217.1050 | 9.80 | 2741 | 458.2135 | 17.86 | 4422 | 562.2868 | 8.93 | 6103 | 835.7685 | 8.49 |
| 1061 | 217.1053 | 6.98 | 2742 | 458.2746 | 8.43 | 4423 | 562.3071 | 10.90 | 6104 | 835.7838 | 9.83 |
| 1062 | 217.1054 | 6.62 | 2743 | 458.2750 | 7.41 | 4424 | 562.3200 | 17.10 | 6105 | 835.7839 | 10.18 |
| 1063 | 217.1055 | 7.02 | 2744 | 458.2758 | 8.56 | 4425 | 562.3358 | 17.21 | 6106 | 835.7863 | 9.97 |
| 1064 | 217.1058 | 11.80 | 2745 | 458.2761 | 7.32 | 4426 | 562.3434 | 11.97 | 6107 | 835.7905 | 8.20 |
| 1065 | 217.1073 | 6.60 | 2746 | 458.2779 | 8.46 | 4427 | 562.3461 | 9.80 | 6108 | 835.7921 | 10.03 |
| 1066 | 218.1009 | 0.83 | 2747 | 458.2784 | 7.27 | 4428 | 562.3465 | 9.82 | 6109 | 835.7986 | 9.98 |
| 1067 | 218.1391 | 2.58 | 2748 | 458.2785 | 8.77 | 4429 | 562.3487 | 11.94 | 6110 | 835.7987 | 10.04 |
| 1068 | 218.1393 | 2.61 | 2749 | 458.2789 | 7.60 | 4430 | 562.3505 | 10.21 | 6111 | 835.8027 | 8.38 |
| 1069 | 218.1399 | 2.10 | 2750 | 458.2790 | 8.52 | 4431 | 562.4739 | 8.94 | 6112 | 835.8110 | 8.94 |
| 1070 | 219.0169 | 1.89 | 2751 | 458.2790 | 8.53 | 4432 | 562.4778 | 9.87 | 6113 | 835.8297 | 0.75 |
| 1071 | 219.0171 | 1.91 | 2752 | 458.2808 | 7.95 | 4433 | 562.4781 | 8.17 | 6114 | 835.9103 | 6.48 |
| 1072 | 219.0192 | 1.85 | 2753 | 458.2810 | 8.59 | 4434 | 562.4842 | 10.14 | 6115 | 836.1087 | 10.04 |
| 1073 | 219.0275 | 0.97 | 2754 | 458.2817 | 8.61 | 4435 | 562.6713 | 8.94 | 6116 | 836.1208 | 8.20 |
| 1074 | 219.0458 | 1.24 | 2755 | 458.2818 | 7.78 | 4436 | 562.6816 | 9.90 | 6117 | 836.1215 | 9.99 |
| 1075 | 219.0790 | 0.88 | 2756 | 458.2819 | 8.73 | 4437 | 562.6819 | 8.18 | 6118 | 836.1250 | 8.94 |
| 1076 | 219.0814 | 0.85 | 2757 | 458.2821 | 8.75 | 4438 | 562.6821 | 10.07 | 6119 | 836.1265 | 10.18 |
| 1077 | 219.0838 | 0.82 | 2758 | 458.2822 | 8.66 | 4439 | 562.8446 | 9.80 | 6120 | 836.1293 | 9.97 |
| 1078 | 220.1333 | 6.52 | 2759 | 458.5263 | 8.61 | 4440 | 562.8484 | 9.83 | 6121 | 836.4528 | 10.18 |
| 1079 | 220.1333 | 6.50 | 2760 | 458.5264 | 8.48 | 4441 | 562.8488 | 11.98 | 6122 | 836.4635 | 10.04 |
| 1080 | 220.1344 | 6.76 | 2761 | 458.5287 | 8.79 | 4442 | 562.8557 | 10.21 | 6123 | 836.7940 | 10.04 |
| 1081 | 220.9347 | 0.70 | 2762 | 458.5300 | 8.51 | 4443 | 563.1945 | 11.82 | 6124 | 836.9319 | 7.48 |
| 1082 | 223.0968 | 12.46 | 2763 | 458.5313 | 8.44 | 4444 | 563.2394 | 16.75 | 6125 | 836.9326 | 7.58 |
| 1083 | 223.0970 | 12.49 | 2764 | 458.5314 | 7.95 | 4445 | 563.3506 | 11.97 | 6126 | 836.9365 | 7.76 |
| 1084 | 223.0971 | 12.70 | 2765 | 458.5321 | 8.58 | 4446 | 563.4300 | 19.60 | 6127 | 837.9185 | 6.38 |
| 1085 | 223.0971 | 12.47 | 2766 | 458.5321 | 7.80 | 4447 | 564.3009 | 14.47 | 6128 | 837.9207 | 4.95 |
| 1086 | 223.1003 | 15.33 | 2767 | 458.5325 | 7.60 | 4448 | 564.3016 | 14.35 | 6129 | 837.9362 | 7.73 |
| 1087 | 223.1005 | 12.83 | 2768 | 458.5328 | 8.66 | 4449 | 564.3033 | 11.49 | 6130 | 837.9488 | 7.53 |
| 1088 | 224.0200 | 16.43 | 2769 | 458.5347 | 7.41 | 4450 | 564.3041 | 14.45 | 6131 | 838.8328 | 0.78 |
| 1089 | 224.0215 | 13.24 | 2770 | 458.5349 | 7.18 | 4451 | 564.3056 | 17.59 | 6132 | 838.8453 | 1.04 |
| 1090 | 224.0930 | 8.67 | 2771 | 458.5353 | 7.44 | 4452 | 564.3091 | 17.61 | 6133 | 838.9286 | 7.46 |
| 1091 | 224.0980 | 15.35 | 2772 | 458.5639 | 9.65 | 4453 | 564.3121 | 14.57 | 6134 | 839.3418 | 0.82 |
| 1092 | 225.0342 | 0.99 | 2773 | 458.7768 | 7.62 | 4454 | 564.3559 | 9.34 | 6135 | 839.8356 | 0.78 |
| 1093 | 225.1945 | 13.13 | 2774 | 458.7776 | 8.69 | 4455 | 564.3565 | 9.51 | 6136 | 840.3644 | 16.25 |
| 1094 | 225.1952 | 11.26 | 2775 | 458.7794 | 7.41 | 4456 | 564.3578 | 9.20 | 6137 | 840.8247 | 0.78 |
| 1095 | 225.1970 | 9.14 | 2776 | 458.7803 | 8.46 | 4457 | 564.3587 | 11.16 | 6138 | 840.8435 | 0.88 |
| 1096 | 225.1970 | 16.25 | 2777 | 458.7824 | 8.59 | 4458 | 564.3590 | 9.07 | 6139 | 841.3659 | 16.31 |
| 1097 | 225.1970 | 13.56 | 2778 | 458.7833 | 8.53 | 4459 | 564.3598 | 11.22 | 6140 | 841.3827 | 16.33 |
| 1098 | 225.1971 | 13.36 | 2779 | 458.7835 | 8.75 | 4460 | 564.3610 | 11.19 | 6141 | 842.4441 | 10.07 |
| 1099 | 226.1023 | 1.17 | 2780 | 458.7835 | 8.66 | 4461 | 564.3621 | 9.28 | 6142 | 842.7752 | 10.18 |
| 1100 | 226.1956 | 13.39 | 2781 | 458.7859 | 8.62 | 4462 | 564.3630 | 9.18 | 6143 | 842.7791 | 10.09 |
| 1101 | 226.1984 | 13.34 | 2782 | 458.8562 | 0.65 | 4463 | 564.6112 | 9.68 | 6144 | 843.1156 | 10.04 |
| 1102 | 226.1991 | 16.27 | 2783 | 458.8917 | 9.65 | 4464 | 564.6277 | 11.50 | 6145 | 844.7893 | 7.49 |
| 1103 | 226.1996 | 13.54 | 2784 | 459.0252 | 8.75 | 4465 | 565.3013 | 17.61 | 6146 | 845.6253 | 21.58 |
| 1104 | 226.1996 | 13.38 | 2785 | 459.0302 | 8.59 | 4466 | 565.3020 | 14.55 | 6147 | 846.8187 | 0.78 |
| 1105 | 226.2008 | 13.20 | 2786 | 459.0999 | 0.89 | 4467 | 565.3621 | 11.22 | 6148 | 846.8224 | 0.76 |
| 1106 | 226.2009 | 13.13 | 2787 | 459.2251 | 17.89 | 4468 | 565.3765 | 8.50 | 6149 | 846.8284 | 0.78 |
| 1107 | 226.9463 | 1.01 | 2788 | 459.2515 | 9.20 | 4469 | 565.5567 | 8.89 | 6150 | 846.8306 | 0.84 |
| 1108 | 226.9525 | 0.76 | 2789 | 459.2521 | 9.32 | 4470 | 565.5649 | 9.04 | 6151 | 847.4250 | 11.76 |
| 1109 | 226.9527 | 1.04 | 2790 | 459.2753 | 10.61 | 4471 | 565.5721 | 8.71 | 6152 | 847.7497 | 8.61 |
| 1110 | 227.1989 | 13.20 | 2791 | 459.2877 | 8.61 | 4472 | 565.8131 | 9.01 | 6153 | 847.7658 | 8.64 |
| 1111 | 227.2022 | 13.41 | 2792 | 459.7386 | 8.39 | 4473 | 565.8271 | 8.75 | 6154 | 847.7695 | 8.93 |
| 1112 | 227.2038 | 13.13 | 2793 | 459.7439 | 8.94 | 4474 | 566.0614 | 9.01 | 6155 | 847.7765 | 8.87 |
| 1113 | 227.2041 | 16.27 | 2794 | 459.7448 | 9.98 | 4475 | 566.0633 | 8.89 | 6156 | 848.0898 | 8.62 |
| 1114 | 227.9514 | 0.74 | 2795 | 459.7486 | 9.80 | 4476 | 566.0790 | 8.69 | 6157 | 848.0952 | 8.65 |
| 1115 | 228.9568 | 0.75 | 2796 | 459.7498 | 8.04 | 4477 | 566.3031 | 9.04 | 6158 | 848.1027 | 8.96 |
| 1116 | 229.1541 | 1.28 | 2797 | 459.7501 | 8.38 | 4478 | 566.3142 | 18.22 | 6159 | 848.1124 | 8.72 |
| 1117 | 229.1548 | 1.42 | 2798 | 459.7535 | 8.18 | 4479 | 566.3172 | 15.07 | 6160 | 848.1235 | 8.61 |
| 1118 | 230.0945 | 1.21 | 2799 | 459.7552 | 8.73 | 4480 | 566.3178 | 15.20 | 6161 | 848.4418 | 8.89 |
| 1119 | 230.1424 | 21.36 | 2800 | 459.7571 | 9.90 | 4481 | 566.3203 | 15.09 | 6162 | 848.4436 | 11.76 |
| 1120 | 230.8899 | 21.81 | 2801 | 459.9071 | 8.04 | 4482 | 566.3205 | 18.17 | 6163 | 848.4472 | 8.62 |
| 1121 | 231.0859 | 2.31 | 2802 | 459.9108 | 8.18 | 4483 | 566.3263 | 15.28 | 6164 | 848.4542 | 8.95 |
| 1122 | 231.1010 | 16.19 | 2803 | 459.9114 | 9.87 | 4484 | 566.8774 | 1.04 | 6165 | 848.4559 | 8.64 |
| 1123 | 231.1710 | 6.68 | 2804 | 459.9121 | 8.69 | 4485 | 566.8860 | 0.78 | 6166 | 848.7644 | 8.61 |
| 1124 | 232.1034 | 16.23 | 2805 | 459.9132 | 8.94 | 4486 | 566.8900 | 0.76 | 6167 | 848.7706 | 8.95 |
| 1125 | 232.1175 | 0.81 | 2806 | 459.9138 | 8.21 | 4487 | 566.8910 | 0.86 | 6168 | 849.1088 | 8.62 |
| 1126 | 232.1545 | 5.96 | 2807 | 459.9139 | 8.41 | 4488 | 567.0021 | 7.62 | 6169 | 851.5389 | 10.54 |
| 1127 | 232.1554 | 5.51 | 2808 | 459.9152 | 10.17 | 4489 | 567.0115 | 8.59 | 6170 | 851.5866 | 10.18 |
| 1128 | 232.1554 | 6.23 | 2809 | 459.9170 | 9.83 | 4490 | 567.3167 | 15.08 | 6171 | 854.8081 | 0.81 |
| 1129 | 232.1557 | 5.81 | 2810 | 459.9173 | 9.84 | 4491 | 567.3283 | 18.22 | 6172 | 854.8100 | 0.79 |
| 1130 | 232.8913 | 21.70 | 2811 | 459.9407 | 0.89 | 4492 | 567.3293 | 15.30 | 6173 | 855.8146 | 0.81 |
| 1131 | 233.0401 | 18.25 | 2812 | 460.0826 | 8.04 | 4493 | 567.3294 | 15.17 | 6174 | 856.3358 | 17.61 |
| 1132 | 233.9819 | 1.88 | 2813 | 460.0832 | 8.18 | 4494 | 567.3378 | 8.61 | 6175 | 856.3442 | 14.54 |
| 1133 | 233.9833 | 1.95 | 2814 | 460.0833 | 8.96 | 4495 | 567.3386 | 7.60 | 6176 | 856.9101 | 4.98 |
| 1134 | 233.9835 | 1.75 | 2815 | 460.0833 | 9.81 | 4496 | 567.3413 | 8.68 | 6177 | 857.3325 | 17.59 |
| 1135 | 233.9838 | 1.72 | 2816 | 460.0837 | 9.84 | 4497 | 567.6733 | 8.59 | 6178 | 858.1957 | 9.49 |
| 1136 | 233.9842 | 1.07 | 2817 | 460.0837 | 9.89 | 4498 | 567.7787 | 8.46 | 6179 | 858.3470 | 17.60 |
| 1137 | 234.0740 | 0.83 | 2818 | 460.0840 | 9.91 | 4499 | 567.8856 | 0.76 | 6180 | 858.4109 | 9.48 |
| 1138 | 234.9105 | 2.64 | 2819 | 460.0870 | 8.70 | 4500 | 568.3352 | 15.04 | 6181 | 858.6089 | 9.49 |
| 1139 | 235.0295 | 18.25 | 2820 | 460.0894 | 8.41 | 4501 | 568.3370 | 18.73 | 6182 | 859.0059 | 8.46 |
| 1140 | 235.1677 | 15.80 | 2821 | 460.2383 | 9.91 | 4502 | 568.3374 | 18.70 | 6183 | 859.0062 | 8.37 |
| 1141 | 235.1683 | 15.60 | 2822 | 460.2411 | 9.89 | 4503 | 568.3384 | 15.11 | 6184 | 859.0095 | 8.24 |
| 1142 | 235.1685 | 15.77 | 2823 | 460.2429 | 8.04 | 4504 | 568.3391 | 18.14 | 6185 | 859.0117 | 7.16 |
| 1143 | 235.1687 | 15.94 | 2824 | 460.2497 | 8.18 | 4505 | 568.3393 | 14.95 | 6186 | 859.0148 | 8.39 |
| 1144 | 235.1689 | 18.97 | 2825 | 460.2518 | 9.80 | 4506 | 568.8832 | 0.80 | 6187 | 859.4901 | 8.47 |
| 1145 | 237.1029 | 14.57 | 2826 | 460.2530 | 8.97 | 4507 | 569.3098 | 11.15 | 6188 | 859.4976 | 8.33 |
| 1146 | 237.2204 | 18.11 | 2827 | 460.2564 | 10.14 | 4508 | 569.3127 | 9.18 | 6189 | 859.4986 | 8.39 |
| 1147 | 237.2236 | 17.97 | 2828 | 460.2648 | 10.14 | 4509 | 569.3132 | 11.20 | 6190 | 859.5064 | 8.37 |
| 1148 | 238.8852 | 0.69 | 2829 | 460.2711 | 10.11 | 4510 | 569.3157 | 9.35 | 6191 | 859.5101 | 8.22 |
| 1149 | 239.0899 | 11.92 | 2830 | 460.3101 | 8.70 | 4511 | 569.3158 | 11.19 | 6192 | 859.5122 | 7.14 |
| 1150 | 239.0911 | 9.76 | 2831 | 460.3126 | 10.23 | 4512 | 569.3230 | 9.51 | 6193 | 860.0048 | 8.29 |
| 1151 | 239.0917 | 10.32 | 2832 | 460.3135 | 9.06 | 4513 | 569.3384 | 14.95 | 6194 | 860.0066 | 8.46 |
| 1152 | 239.0920 | 10.00 | 2833 | 460.3205 | 10.42 | 4514 | 569.3400 | 15.15 | 6195 | 860.0151 | 8.37 |
| 1153 | 239.0921 | 11.92 | 2834 | 460.4074 | 10.01 | 4515 | 569.3421 | 18.16 | 6196 | 860.0153 | 8.47 |
| 1154 | 239.0931 | 10.20 | 2835 | 460.4125 | 8.17 | 4516 | 569.3444 | 15.04 | 6197 | 861.8081 | 5.11 |
| 1155 | 239.0934 | 10.51 | 2836 | 460.4155 | 8.94 | 4517 | 570.3347 | 18.14 | 6198 | 861.8147 | 6.73 |
| 1156 | 239.1475 | 7.75 | 2837 | 460.4161 | 8.18 | 4518 | 570.3398 | 18.54 | 6199 | 861.8190 | 6.83 |
| 1157 | 239.1625 | 14.31 | 2838 | 460.4196 | 9.89 | 4519 | 570.3480 | 15.14 | 6200 | 861.8233 | 4.91 |
| 1158 | 239.1650 | 17.60 | 2839 | 460.4227 | 9.90 | 4520 | 570.3503 | 15.57 | 6201 | 862.7958 | 0.81 |
| 1159 | 239.2364 | 21.34 | 2840 | 460.4229 | 10.14 | 4521 | 570.3508 | 15.47 | 6202 | 864.4110 | 11.20 |
| 1160 | 240.1678 | 17.51 | 2841 | 460.4275 | 8.04 | 4522 | 571.2523 | 8.46 | 6203 | 865.4065 | 11.20 |
| 1161 | 240.4296 | 17.60 | 2842 | 460.5774 | 10.07 | 4523 | 571.2754 | 8.24 | 6204 | 867.2679 | 10.92 |
| 1162 | 240.4323 | 17.74 | 2843 | 460.5922 | 9.90 | 4524 | 571.2772 | 7.82 | 6205 | 867.3964 | 10.93 |
| 1163 | 241.0321 | 0.89 | 2844 | 461.1016 | 0.95 | 4525 | 571.2892 | 7.92 | 6206 | 867.5418 | 10.90 |
| 1164 | 241.0344 | 0.90 | 2845 | 462.2611 | 8.90 | 4526 | 571.2926 | 8.00 | 6207 | 869.4290 | 11.74 |
| 1165 | 241.1534 | 18.86 | 2846 | 462.2686 | 9.31 | 4527 | 571.2953 | 8.01 | 6208 | 869.9912 | 8.46 |
| 1166 | 241.1573 | 18.38 | 2847 | 462.2696 | 7.68 | 4528 | 571.3494 | 18.53 | 6209 | 869.9951 | 8.33 |
| 1167 | 241.1609 | 18.27 | 2848 | 462.2711 | 9.72 | 4529 | 571.4883 | 8.46 | 6210 | 870.0078 | 8.34 |
| 1168 | 241.1616 | 18.25 | 2849 | 462.2719 | 9.37 | 4530 | 571.5039 | 8.29 | 6211 | 870.4951 | 8.47 |
| 1169 | 241.1650 | 18.17 | 2850 | 462.2742 | 9.32 | 4531 | 571.7943 | 8.01 | 6212 | 870.5029 | 8.34 |
| 1170 | 241.1660 | 18.27 | 2851 | 462.2761 | 7.95 | 4532 | 572.1708 | 0.98 | 6213 | 870.5152 | 8.33 |
| 1171 | 241.1715 | 14.47 | 2852 | 462.8437 | 8.82 | 4533 | 572.9998 | 8.46 | 6214 | 870.7899 | 0.83 |
| 1172 | 241.1727 | 17.61 | 2853 | 462.8578 | 0.84 | 4534 | 573.0057 | 8.33 | 6215 | 870.9774 | 8.34 |
| 1173 | 241.1735 | 14.49 | 2854 | 462.9943 | 0.92 | 4535 | 573.0061 | 8.24 | 6216 | 870.9987 | 8.47 |
| 1174 | 241.1752 | 14.60 | 2855 | 463.0273 | 0.79 | 4536 | 573.0061 | 8.37 | 6217 | 871.7688 | 10.07 |
| 1175 | 242.1747 | 14.58 | 2856 | 463.0471 | 8.82 | 4537 | 573.0066 | 7.56 | 6218 | 872.0681 | 9.84 |
| 1176 | 242.1755 | 14.49 | 2857 | 465.1507 | 0.95 | 4538 | 573.0090 | 8.34 | 6219 | 872.0955 | 10.03 |
| 1177 | 242.1757 | 14.35 | 2858 | 465.3097 | 10.97 | 4539 | 573.0093 | 8.01 | 6220 | 872.4433 | 10.04 |
| 1178 | 242.1770 | 14.50 | 2859 | 466.2035 | 15.64 | 4540 | 573.0097 | 8.40 | 6221 | 872.8291 | 0.76 |
| 1179 | 242.1792 | 17.64 | 2860 | 466.2409 | 10.00 | 4541 | 573.0097 | 7.14 | 6222 | 872.8331 | 0.80 |
| 1180 | 242.1794 | 14.60 | 2861 | 466.2478 | 7.47 | 4542 | 573.0097 | 8.47 | 6223 | 873.3331 | 0.79 |
| 1181 | 242.1794 | 14.60 | 2862 | 466.2488 | 8.84 | 4543 | 573.0098 | 8.19 | 6224 | 873.8316 | 0.78 |
| 1182 | 242.2851 | 15.55 | 2863 | 466.2514 | 8.81 | 4544 | 573.3398 | 7.99 | 6225 | 873.9031 | 11.41 |
| 1183 | 242.2858 | 12.99 | 2864 | 466.2566 | 8.65 | 4545 | 573.3403 | 8.21 | 6226 | 874.4118 | 11.41 |
| 1184 | 242.2858 | 12.70 | 2865 | 466.3184 | 15.86 | 4546 | 573.3406 | 8.47 | 6227 | 875.8233 | 4.95 |
| 1185 | 242.2880 | 13.01 | 2866 | 466.5865 | 8.81 | 4547 | 573.3409 | 7.56 | 6228 | 875.8269 | 7.46 |
| 1186 | 242.9260 | 0.78 | 2867 | 466.5886 | 8.65 | 4548 | 573.3429 | 8.29 | 6229 | 876.9292 | 5.73 |
| 1187 | 242.9266 | 0.84 | 2868 | 466.5900 | 8.84 | 4549 | 573.3434 | 8.33 | 6230 | 877.4546 | 8.91 |
| 1188 | 242.9283 | 0.76 | 2869 | 466.9191 | 8.84 | 4550 | 573.3438 | 8.24 | 6231 | 877.4559 | 9.41 |
| 1189 | 243.1553 | 9.58 | 2870 | 467.0143 | 8.85 | 4551 | 573.3438 | 8.37 | 6232 | 880.8134 | 0.80 |
| 1190 | 243.1664 | 14.49 | 2871 | 467.2591 | 8.64 | 4552 | 573.3439 | 7.16 | 6233 | 880.8219 | 0.80 |
| 1191 | 243.1666 | 14.35 | 2872 | 467.2612 | 8.85 | 4553 | 573.4024 | 21.52 | 6234 | 880.9665 | 8.33 |
| 1192 | 243.1698 | 14.62 | 2873 | 467.3156 | 15.84 | 4554 | 573.6709 | 8.34 | 6235 | 880.9751 | 8.46 |
| 1193 | 243.1704 | 14.61 | 2874 | 467.7621 | 8.59 | 4555 | 573.6715 | 7.16 | 6236 | 881.3232 | 0.83 |
| 1194 | 243.1720 | 17.62 | 2875 | 467.7927 | 6.11 | 4556 | 573.6773 | 8.29 | 6237 | 881.4943 | 8.34 |
| 1195 | 243.1725 | 14.52 | 2876 | 468.0098 | 8.61 | 4557 | 573.6786 | 8.46 | 6238 | 881.4943 | 8.46 |
| 1196 | 243.2858 | 12.70 | 2877 | 468.3069 | 14.29 | 4558 | 573.6813 | 8.01 | 6239 | 881.9937 | 8.49 |
| 1197 | 243.2858 | 12.69 | 2878 | 468.3082 | 14.16 | 4559 | 573.6816 | 8.37 | 6240 | 882.4656 | 9.17 |
| 1198 | 243.2874 | 12.79 | 2879 | 468.3084 | 14.20 | 4560 | 574.0129 | 7.14 | 6241 | 882.4704 | 9.18 |
| 1199 | 243.2875 | 15.52 | 2880 | 468.3090 | 14.39 | 4561 | 574.0155 | 8.34 | 6242 | 882.8014 | 9.18 |
| 1200 | 243.2901 | 12.99 | 2881 | 468.3090 | 14.34 | 4562 | 574.0195 | 8.39 | 6243 | 882.8058 | 9.08 |
| 1201 | 243.2904 | 13.07 | 2882 | 468.3095 | 17.11 | 4563 | 575.3039 | 8.50 | 6244 | 883.1400 | 11.15 |
| 1202 | 244.1691 | 17.62 | 2883 | 468.3097 | 14.47 | 4564 | 575.3082 | 7.69 | 6245 | 883.1445 | 9.17 |
| 1203 | 245.0742 | 0.99 | 2884 | 468.3101 | 14.36 | 4565 | 576.5616 | 11.86 | 6246 | 883.2603 | 11.14 |
| 1204 | 245.1852 | 7.39 | 2885 | 468.3125 | 17.38 | 4566 | 576.8054 | 11.86 | 6247 | 883.2718 | 10.93 |
| 1205 | 245.1854 | 7.51 | 2886 | 468.3125 | 14.28 | 4567 | 576.8466 | 0.75 | 6248 | 883.4125 | 11.15 |
| 1206 | 245.1868 | 7.72 | 2887 | 468.3133 | 17.27 | 4568 | 577.2245 | 18.27 | 6249 | 883.4144 | 10.90 |
| 1207 | 245.1878 | 7.28 | 2888 | 469.3107 | 14.34 | 4569 | 577.2555 | 15.67 | 6250 | 883.4292 | 11.20 |
| 1208 | 245.2235 | 19.09 | 2889 | 469.3117 | 14.36 | 4570 | 577.2617 | 17.50 | 6251 | 883.4673 | 9.17 |
| 1209 | 246.1712 | 7.02 | 2890 | 469.3139 | 17.35 | 4571 | 577.2645 | 17.58 | 6252 | 883.4679 | 9.18 |
| 1210 | 246.1712 | 7.03 | 2891 | 469.3908 | 19.41 | 4572 | 578.1884 | 0.95 | 6253 | 883.4840 | 9.17 |
| 1211 | 247.1430 | 8.29 | 2892 | 470.2617 | 9.36 | 4573 | 578.2619 | 15.69 | 6254 | 883.4853 | 9.34 |
| 1212 | 247.1464 | 12.21 | 2893 | 470.2647 | 11.69 | 4574 | 578.2687 | 17.57 | 6255 | 883.5550 | 11.15 |
| 1213 | 247.1637 | 18.98 | 2894 | 470.3139 | 17.38 | 4575 | 578.2720 | 17.50 | 6256 | 883.5563 | 10.92 |
| 1214 | 247.1641 | 6.12 | 2895 | 470.5982 | 9.36 | 4576 | 578.3060 | 8.82 | 6257 | 883.5675 | 11.20 |
| 1215 | 247.1649 | 5.81 | 2896 | 470.8861 | 0.70 | 4577 | 579.1866 | 0.96 | 6258 | 883.6742 | 10.93 |
| 1216 | 247.1651 | 5.93 | 2897 | 471.2209 | 9.50 | 4578 | 579.2588 | 17.58 | 6259 | 883.6892 | 11.15 |
| 1217 | 247.1659 | 18.95 | 2898 | 471.2210 | 9.54 | 4579 | 579.2627 | 17.46 | 6260 | 883.6933 | 11.18 |
| 1218 | 247.1670 | 5.51 | 2899 | 471.2221 | 9.45 | 4580 | 579.2629 | 17.57 | 6261 | 883.7133 | 9.40 |
| 1219 | 247.1670 | 5.28 | 2900 | 471.2228 | 8.00 | 4581 | 579.4370 | 21.33 | 6262 | 883.7178 | 9.15 |
| 1220 | 247.1675 | 6.00 | 2901 | 471.2230 | 9.40 | 4582 | 580.2681 | 17.57 | 6263 | 883.8117 | 9.18 |
| 1221 | 247.1675 | 5.17 | 2902 | 471.2249 | 9.42 | 4583 | 580.4425 | 19.37 | 6264 | 883.8156 | 9.07 |
| 1222 | 247.1677 | 6.36 | 2903 | 471.2257 | 8.14 | 4584 | 581.4200 | 18.90 | 6265 | 883.8205 | 10.92 |
| 1223 | 247.1679 | 21.23 | 2904 | 471.3601 | 13.11 | 4585 | 581.4209 | 15.69 | 6266 | 883.8222 | 11.23 |
| 1224 | 247.1680 | 21.33 | 2905 | 471.3619 | 16.26 | 4586 | 581.4340 | 18.84 | 6267 | 883.8359 | 11.15 |
| 1225 | 247.1681 | 21.31 | 2906 | 471.3638 | 13.38 | 4587 | 582.4243 | 18.88 | 6268 | 883.9911 | 11.15 |
| 1226 | 247.1681 | 19.04 | 2907 | 471.3676 | 13.56 | 4588 | 582.8590 | 0.74 | 6269 | 884.1368 | 11.23 |
| 1227 | 247.1688 | 5.65 | 2908 | 471.3676 | 13.21 | 4589 | 582.8653 | 0.81 | 6270 | 886.4366 | 8.99 |
| 1228 | 247.1766 | 16.30 | 2909 | 471.3695 | 16.24 | 4590 | 582.8657 | 0.83 | 6271 | 888.8000 | 0.74 |
| 1229 | 247.1799 | 13.57 | 2910 | 471.3728 | 13.14 | 4591 | 582.9054 | 9.67 | 6272 | 890.7782 | 7.50 |
| 1230 | 247.1811 | 13.20 | 2911 | 471.7313 | 8.49 | 4592 | 583.1077 | 8.84 | 6273 | 891.9658 | 8.49 |
| 1231 | 247.1811 | 13.16 | 2912 | 471.7344 | 8.47 | 4593 | 583.1125 | 9.64 | 6274 | 892.4674 | 8.31 |
| 1232 | 247.1838 | 19.15 | 2913 | 471.7374 | 10.17 | 4594 | 583.2602 | 16.40 | 6275 | 892.4840 | 8.49 |
| 1233 | 247.1845 | 13.39 | 2914 | 471.7375 | 8.44 | 4595 | 583.3067 | 9.68 | 6276 | 895.4966 | 9.18 |
| 1234 | 248.1251 | 11.87 | 2915 | 471.7376 | 8.46 | 4596 | 583.3191 | 8.87 | 6277 | 895.5193 | 8.92 |
| 1235 | 248.1276 | 11.74 | 2916 | 471.7393 | 8.32 | 4597 | 583.5159 | 9.66 | 6278 | 895.5273 | 9.28 |
| 1236 | 248.1279 | 14.10 | 2917 | 471.7416 | 8.25 | 4598 | 583.7067 | 9.66 | 6279 | 896.5244 | 9.29 |
| 1237 | 248.1296 | 11.53 | 2918 | 471.7529 | 8.44 | 4599 | 583.9154 | 9.83 | 6280 | 896.7855 | 0.84 |
| 1238 | 248.1737 | 16.28 | 2919 | 471.7620 | 8.43 | 4600 | 584.1060 | 9.81 | 6281 | 899.6321 | 15.79 |
| 1239 | 251.0020 | 17.10 | 2920 | 471.7638 | 8.32 | 4601 | 584.2626 | 16.37 | 6282 | 899.6383 | 17.44 |
| 1240 | 251.1262 | 16.96 | 2921 | 472.0526 | 8.40 | 4602 | 584.3075 | 9.76 | 6283 | 899.7434 | 10.33 |
| 1241 | 252.1208 | 17.30 | 2922 | 472.0568 | 8.49 | 4603 | 584.3092 | 11.89 | 6284 | 899.9305 | 10.38 |
| 1242 | 253.0987 | 11.82 | 2923 | 472.2256 | 9.53 | 4604 | 584.3114 | 9.87 | 6285 | 899.9699 | 10.60 |
| 1243 | 253.9926 | 1.43 | 2924 | 472.2383 | 7.76 | 4605 | 584.3127 | 11.92 | 6286 | 900.0870 | 10.38 |
| 1244 | 255.2095 | 16.82 | 2925 | 472.2395 | 7.23 | 4606 | 584.3137 | 11.85 | 6287 | 900.0945 | 10.33 |
| 1245 | 255.2111 | 16.86 | 2926 | 472.2400 | 8.43 | 4607 | 584.5019 | 9.84 | 6288 | 900.2504 | 10.34 |
| 1246 | 255.2111 | 16.84 | 2927 | 472.2401 | 8.44 | 4608 | 584.7718 | 12.09 | 6289 | 900.4831 | 10.58 |
| 1247 | 255.2126 | 20.40 | 2928 | 472.2415 | 7.72 | 4609 | 584.7787 | 10.26 | 6290 | 900.6311 | 15.80 |
| 1248 | 255.2137 | 16.92 | 2929 | 472.2417 | 7.13 | 4610 | 585.2678 | 13.41 | 6291 | 900.7977 | 0.76 |
| 1249 | 255.2294 | 17.83 | 2930 | 472.2417 | 7.78 | 4611 | 585.3154 | 11.92 | 6292 | 903.1787 | 10.04 |
| 1250 | 255.2315 | 17.89 | 2931 | 472.2418 | 7.53 | 4612 | 585.3157 | 11.74 | 6293 | 903.3231 | 10.07 |
| 1251 | 255.2328 | 18.10 | 2932 | 472.2418 | 8.32 | 4613 | 586.3028 | 18.86 | 6294 | 903.8173 | 0.76 |
| 1252 | 255.9433 | 21.69 | 2933 | 472.2419 | 10.18 | 4614 | 586.3089 | 11.91 | 6295 | 906.8326 | 0.81 |
| 1253 | 255.9776 | 0.89 | 2934 | 472.2420 | 6.78 | 4615 | 586.3118 | 18.80 | 6296 | 906.8354 | 0.75 |
| 1254 | 256.0974 | 15.27 | 2935 | 472.2421 | 7.71 | 4616 | 586.3172 | 15.68 | 6297 | 907.3430 | 0.79 |
| 1255 | 257.1503 | 0.99 | 2936 | 472.2423 | 8.01 | 4617 | 587.3131 | 18.87 | 6298 | 907.8241 | 0.82 |
| 1256 | 257.1522 | 0.95 | 2937 | 472.2428 | 6.56 | 4618 | 587.3522 | 8.67 | 6299 | 907.8310 | 0.76 |
| 1257 | 257.1653 | 14.52 | 2938 | 472.2433 | 6.97 | 4619 | 589.3054 | 7.71 | 6300 | 908.4942 | 9.13 |
| 1258 | 257.1687 | 17.71 | 2939 | 472.2434 | 6.95 | 4620 | 589.3069 | 7.17 | 6301 | 908.4978 | 9.04 |
| 1259 | 257.1727 | 17.60 | 2940 | 472.2435 | 7.84 | 4621 | 589.3077 | 9.40 | 6302 | 908.8356 | 0.86 |
| 1260 | 257.2467 | 21.27 | 2941 | 472.2435 | 7.68 | 4622 | 589.3078 | 7.76 | 6303 | 908.9953 | 9.04 |
| 1261 | 258.1150 | 1.01 | 2942 | 472.2444 | 7.75 | 4623 | 589.3084 | 7.64 | 6304 | 909.0044 | 9.13 |
| 1262 | 258.1538 | 0.97 | 2943 | 472.2445 | 7.33 | 4624 | 589.3086 | 7.68 | 6305 | 909.5061 | 9.02 |
| 1263 | 258.2499 | 21.27 | 2944 | 472.2446 | 7.79 | 4625 | 589.3091 | 7.50 | 6306 | 909.5072 | 9.15 |
| 1264 | 258.9013 | 0.83 | 2945 | 472.2457 | 8.02 | 4626 | 589.3091 | 8.00 | 6307 | 914.7905 | 0.80 |
| 1265 | 258.9972 | 0.68 | 2946 | 472.2466 | 7.66 | 4627 | 589.3095 | 6.98 | 6308 | 915.5563 | 8.72 |
| 1266 | 258.9983 | 0.62 | 2947 | 472.2938 | 17.85 | 4628 | 589.3095 | 6.66 | 6309 | 915.5568 | 8.59 |
| 1267 | 261.1290 | 7.59 | 2948 | 472.3044 | 15.82 | 4629 | 589.3156 | 6.82 | 6310 | 916.0517 | 8.61 |
| 1268 | 261.1291 | 7.73 | 2949 | 472.3064 | 17.42 | 4630 | 590.0475 | 7.75 | 6311 | 916.0600 | 8.68 |
| 1269 | 261.1295 | 7.77 | 2950 | 472.3672 | 16.27 | 4631 | 590.0481 | 7.78 | 6312 | 916.0605 | 8.59 |
| 1270 | 262.0127 | 2.43 | 2951 | 472.4358 | 8.02 | 4632 | 590.0491 | 8.02 | 6313 | 916.5470 | 8.61 |
| 1271 | 263.0137 | 0.87 | 2952 | 472.4375 | 7.14 | 4633 | 590.0497 | 7.85 | 6314 | 918.4745 | 8.70 |
| 1272 | 263.0893 | 15.30 | 2953 | 472.4389 | 6.54 | 4634 | 590.0518 | 7.53 | 6315 | 918.4766 | 9.81 |
| 1273 | 263.0900 | 12.83 | 2954 | 472.4408 | 7.79 | 4635 | 590.0546 | 7.76 | 6316 | 918.4807 | 8.94 |
| 1274 | 263.0925 | 12.67 | 2955 | 472.4410 | 7.78 | 4636 | 590.0560 | 7.68 | 6317 | 918.4879 | 8.04 |
| 1275 | 263.2306 | 18.94 | 2956 | 472.4421 | 7.65 | 4637 | 590.2849 | 6.97 | 6318 | 918.4885 | 8.18 |
| 1276 | 263.2340 | 19.09 | 2957 | 472.4427 | 7.84 | 4638 | 590.2978 | 7.76 | 6319 | 918.4902 | 9.90 |
| 1277 | 263.2363 | 19.00 | 2958 | 472.4428 | 7.66 | 4639 | 590.3026 | 8.02 | 6320 | 918.4949 | 10.06 |
| 1278 | 263.2363 | 18.21 | 2959 | 472.4443 | 6.92 | 4640 | 590.3032 | 7.66 | 6321 | 918.4955 | 9.87 |
| 1279 | 263.2374 | 18.47 | 2960 | 472.4444 | 7.71 | 4641 | 590.3088 | 7.68 | 6322 | 918.6018 | 10.65 |
| 1280 | 263.2384 | 19.15 | 2961 | 472.4444 | 6.75 | 4642 | 590.3118 | 7.69 | 6323 | 918.7591 | 10.66 |
| 1281 | 263.2385 | 18.24 | 2962 | 472.4446 | 7.50 | 4643 | 590.3120 | 15.11 | 6324 | 918.8099 | 8.94 |
| 1282 | 263.2407 | 18.98 | 2963 | 472.4468 | 7.33 | 4644 | 590.3124 | 7.80 | 6325 | 918.8182 | 10.04 |
| 1283 | 264.1356 | 5.53 | 2964 | 472.6370 | 7.79 | 4645 | 590.3194 | 18.14 | 6326 | 918.8183 | 8.38 |
| 1284 | 265.0156 | 1.02 | 2965 | 472.6373 | 6.76 | 4646 | 590.3229 | 15.02 | 6327 | 918.8252 | 8.69 |
| 1285 | 265.1547 | 7.11 | 2966 | 472.6381 | 6.57 | 4647 | 590.3244 | 15.14 | 6328 | 918.8262 | 8.18 |
| 1286 | 265.1560 | 7.33 | 2967 | 472.6389 | 6.95 | 4648 | 590.5420 | 7.69 | 6329 | 918.8283 | 9.89 |
| 1287 | 265.1561 | 7.35 | 2968 | 472.6389 | 7.68 | 4649 | 590.5444 | 7.75 | 6330 | 918.8319 | 8.41 |
| 1288 | 265.1570 | 7.19 | 2969 | 472.6399 | 7.75 | 4650 | 590.5457 | 7.51 | 6331 | 918.8341 | 8.04 |
| 1289 | 265.2470 | 17.66 | 2970 | 472.6399 | 7.76 | 4651 | 590.5459 | 8.02 | 6332 | 918.8357 | 9.81 |
| 1290 | 265.2493 | 17.78 | 2971 | 472.6403 | 7.53 | 4652 | 590.5516 | 7.79 | 6333 | 918.9472 | 10.66 |
| 1291 | 265.2514 | 19.43 | 2972 | 472.6425 | 7.35 | 4653 | 590.5576 | 7.84 | 6334 | 919.1012 | 10.66 |
| 1292 | 265.2515 | 17.88 | 2973 | 472.6433 | 7.13 | 4654 | 590.5583 | 7.76 | 6335 | 919.1478 | 9.81 |
| 1293 | 265.2518 | 19.67 | 2974 | 472.6443 | 8.02 | 4655 | 590.7948 | 7.76 | 6336 | 919.1564 | 8.18 |
| 1294 | 265.2526 | 19.72 | 2975 | 472.6451 | 7.84 | 4656 | 591.2643 | 9.49 | 6337 | 919.1575 | 9.89 |
| 1295 | 265.2538 | 17.95 | 2976 | 472.6464 | 7.78 | 4657 | 591.3206 | 15.02 | 6338 | 919.1601 | 8.96 |
| 1296 | 265.2539 | 18.03 | 2977 | 472.6776 | 8.90 | 4658 | 591.3211 | 18.16 | 6339 | 919.1622 | 8.07 |
| 1297 | 265.2539 | 19.59 | 2978 | 472.7354 | 8.32 | 4659 | 591.3228 | 15.10 | 6340 | 919.1725 | 8.73 |
| 1298 | 265.2548 | 19.68 | 2979 | 472.7459 | 8.44 | 4660 | 591.3244 | 12.08 | 6341 | 919.2551 | 10.66 |
| 1299 | 267.1653 | 21.59 | 2980 | 472.8383 | 7.84 | 4661 | 591.7720 | 9.49 | 6342 | 919.4769 | 8.94 |
| 1300 | 267.1672 | 6.19 | 2981 | 472.8383 | 7.68 | 4662 | 591.8112 | 10.39 | 6343 | 919.4890 | 8.18 |
| 1301 | 267.1680 | 6.61 | 2982 | 472.8394 | 7.79 | 4663 | 591.8113 | 8.38 | 6344 | 919.4941 | 8.17 |
| 1302 | 267.1682 | 15.68 | 2983 | 472.8405 | 8.02 | 4664 | 591.8142 | 10.51 | 6345 | 919.4966 | 8.05 |
| 1303 | 267.1690 | 18.74 | 2984 | 472.8446 | 7.58 | 4665 | 591.8166 | 10.48 | 6346 | 919.5009 | 8.69 |
| 1304 | 267.1708 | 15.41 | 2985 | 472.8453 | 7.75 | 4666 | 592.3124 | 8.79 | 6347 | 919.5036 | 9.90 |
| 1305 | 267.1751 | 15.49 | 2986 | 472.8485 | 7.76 | 4667 | 592.3145 | 10.45 | 6348 | 919.8115 | 9.90 |
| 1306 | 267.6475 | 15.60 | 2987 | 473.0448 | 7.76 | 4668 | 592.3190 | 10.49 | 6349 | 919.8241 | 10.06 |
| 1307 | 267.6480 | 15.48 | 2988 | 473.3002 | 17.43 | 4669 | 592.3413 | 18.54 | 6350 | 919.8277 | 8.17 |
| 1308 | 267.6495 | 15.66 | 2989 | 473.3134 | 15.82 | 4670 | 592.3629 | 10.15 | 6351 | 919.8316 | 8.96 |
| 1309 | 269.0887 | 5.10 | 2990 | 474.8304 | 0.65 | 4671 | 592.3635 | 10.68 | 6352 | 920.1709 | 9.90 |
| 1310 | 269.0892 | 5.43 | 2991 | 474.8304 | 21.69 | 4672 | 592.3677 | 9.94 | 6353 | 920.4870 | 9.86 |
| 1311 | 269.0914 | 5.11 | 2992 | 474.8319 | 0.64 | 4673 | 592.3681 | 9.82 | 6354 | 921.6238 | 15.80 |
| 1312 | 269.0915 | 5.40 | 2993 | 476.1929 | 6.84 | 4674 | 592.3728 | 12.05 | 6355 | 922.8012 | 0.78 |
| 1313 | 269.9936 | 0.90 | 2994 | 476.2765 | 18.99 | 4675 | 592.3755 | 10.32 | 6356 | 923.7979 | 0.82 |
| 1314 | 271.1096 | 11.83 | 2995 | 476.3036 | 10.57 | 4676 | 592.3755 | 11.99 | 6357 | 924.2693 | 17.28 |
| 1315 | 271.1856 | 13.66 | 2996 | 476.3086 | 8.65 | 4677 | 592.3767 | 12.06 | 6358 | 924.2705 | 17.00 |
| 1316 | 271.1879 | 13.14 | 2997 | 476.3093 | 9.24 | 4678 | 592.3929 | 11.20 | 6359 | 925.2798 | 19.18 |
| 1317 | 271.1897 | 13.29 | 2998 | 476.3095 | 8.94 | 4679 | 593.3635 | 8.90 | 6360 | 925.8146 | 9.89 |
| 1318 | 271.1904 | 16.37 | 2999 | 476.3098 | 9.01 | 4680 | 593.3645 | 9.74 | 6361 | 926.1456 | 9.87 |
| 1319 | 271.2431 | 19.28 | 3000 | 476.3118 | 10.61 | 4681 | 593.3652 | 9.34 | 6362 | 926.1509 | 10.15 |
| 1320 | 272.0890 | 15.99 | 3001 | 476.3123 | 8.83 | 4682 | 593.3674 | 7.95 | 6363 | 926.1567 | 8.20 |
| 1321 | 272.1974 | 8.65 | 3002 | 476.3126 | 8.84 | 4683 | 593.3676 | 7.68 | 6364 | 926.4928 | 9.89 |
| 1322 | 272.9443 | 0.74 | 3003 | 477.3076 | 9.22 | 4684 | 593.3721 | 12.06 | 6365 | 926.5427 | 8.73 |
| 1323 | 273.1624 | 13.80 | 3004 | 477.3096 | 10.61 | 4685 | 593.3744 | 9.83 | 6366 | 926.5443 | 8.68 |
| 1324 | 273.1654 | 9.72 | 3005 | 478.2850 | 15.18 | 4686 | 593.3770 | 7.79 | 6367 | 926.5458 | 8.79 |
| 1325 | 274.1882 | 4.55 | 3006 | 478.2899 | 18.37 | 4687 | 593.9354 | 8.55 | 6368 | 926.5534 | 8.59 |
| 1326 | 274.1892 | 4.78 | 3007 | 478.2903 | 18.20 | 4688 | 593.9478 | 7.15 | 6369 | 926.8308 | 9.91 |
| 1327 | 274.2741 | 11.27 | 3008 | 478.2913 | 15.31 | 4689 | 594.1176 | 8.57 | 6370 | 927.0353 | 8.79 |
| 1328 | 274.2745 | 11.14 | 3009 | 478.2942 | 15.22 | 4690 | 594.3589 | 11.74 | 6371 | 927.0514 | 8.59 |
| 1329 | 274.2748 | 11.48 | 3010 | 478.2944 | 15.21 | 4691 | 594.8159 | 9.51 | 6372 | 927.5583 | 8.59 |
| 1330 | 274.2760 | 11.60 | 3011 | 478.2946 | 15.08 | 4692 | 594.8164 | 8.13 | 6373 | 931.2274 | 21.44 |
| 1331 | 274.2767 | 13.79 | 3012 | 478.2978 | 15.27 | 4693 | 594.8171 | 9.10 | 6374 | 931.4607 | 10.01 |
| 1332 | 274.9766 | 0.62 | 3013 | 478.3229 | 15.97 | 4694 | 594.8177 | 9.27 | 6375 | 931.4748 | 9.90 |
| 1333 | 275.0670 | 16.23 | 3014 | 478.3254 | 18.76 | 4695 | 594.8201 | 7.76 | 6376 | 931.8102 | 9.84 |
| 1334 | 275.2754 | 11.48 | 3015 | 478.3273 | 18.85 | 4696 | 594.8203 | 8.50 | 6377 | 933.1500 | 9.90 |
| 1335 | 275.2771 | 11.27 | 3016 | 478.3275 | 15.48 | 4697 | 594.8205 | 9.42 | 6378 | 933.4818 | 9.90 |
| 1336 | 275.2775 | 11.64 | 3017 | 478.3284 | 18.87 | 4698 | 594.8207 | 7.80 | 6379 | 933.8131 | 9.86 |
| 1337 | 275.2797 | 13.79 | 3018 | 478.3286 | 15.64 | 4699 | 594.8218 | 8.34 | 6380 | 936.1262 | 9.87 |
| 1338 | 275.2799 | 11.14 | 3019 | 478.3336 | 18.92 | 4700 | 594.8229 | 9.35 | 6381 | 936.4600 | 10.12 |
| 1339 | 276.1165 | 1.33 | 3020 | 478.3373 | 15.68 | 4701 | 594.8238 | 9.45 | 6382 | 936.4748 | 9.90 |
| 1340 | 276.1191 | 1.34 | 3021 | 478.3386 | 15.72 | 4702 | 594.8246 | 8.82 | 6383 | 936.7919 | 10.04 |
| 1341 | 276.1340 | 6.36 | 3022 | 478.7259 | 6.57 | 4703 | 595.0644 | 7.76 | 6384 | 936.8072 | 9.90 |
| 1342 | 276.6332 | 15.74 | 3023 | 478.7266 | 7.49 | 4704 | 595.0654 | 9.27 | 6385 | 936.8100 | 8.20 |
| 1343 | 276.6375 | 15.66 | 3024 | 478.7277 | 7.53 | 4705 | 595.0666 | 9.36 | 6386 | 937.1267 | 9.90 |
| 1344 | 276.6377 | 18.84 | 3025 | 478.7285 | 7.93 | 4706 | 595.0669 | 9.38 | 6387 | 937.5376 | 8.61 |
| 1345 | 276.6389 | 15.46 | 3026 | 478.7294 | 7.64 | 4707 | 595.0678 | 8.49 | 6388 | 937.5417 | 8.64 |
| 1346 | 277.1726 | 19.23 | 3027 | 478.7299 | 7.26 | 4708 | 595.0684 | 9.07 | 6389 | 937.8173 | 0.75 |
| 1347 | 277.1751 | 16.19 | 3028 | 479.2259 | 6.57 | 4709 | 595.0705 | 9.51 | 6390 | 938.0384 | 8.64 |
| 1348 | 277.1806 | 15.92 | 3029 | 479.2298 | 7.49 | 4710 | 595.0706 | 8.02 | 6391 | 938.0389 | 8.59 |
| 1349 | 278.1720 | 7.55 | 3030 | 479.2940 | 18.34 | 4711 | 595.0718 | 7.80 | 6392 | 938.5357 | 8.59 |
| 1350 | 279.1591 | 16.25 | 3031 | 479.2942 | 15.18 | 4712 | 595.0719 | 8.84 | 6393 | 938.7679 | 0.84 |
| 1351 | 279.1599 | 19.68 | 3032 | 479.3021 | 14.97 | 4713 | 595.0746 | 9.52 | 6394 | 938.7886 | 0.77 |
| 1352 | 279.1601 | 16.50 | 3033 | 479.3229 | 15.97 | 4714 | 595.0764 | 8.34 | 6395 | 940.8117 | 0.78 |
| 1353 | 279.1607 | 19.61 | 3034 | 479.3306 | 18.90 | 4715 | 595.0781 | 9.47 | 6396 | 940.8167 | 0.84 |
| 1354 | 279.1630 | 19.72 | 3035 | 479.3312 | 15.67 | 4716 | 595.3132 | 9.09 | 6397 | 941.3143 | 0.84 |
| 1355 | 279.1653 | 9.06 | 3036 | 480.2791 | 7.04 | 4717 | 595.3161 | 7.80 | 6398 | 942.4593 | 8.44 |
| 1356 | 279.1696 | 8.29 | 3037 | 480.2794 | 7.13 | 4718 | 595.3178 | 9.36 | 6399 | 942.9351 | 5.53 |
| 1357 | 279.1711 | 7.78 | 3038 | 480.2797 | 6.97 | 4719 | 595.3184 | 9.35 | 6400 | 942.9470 | 5.05 |
| 1358 | 279.2312 | 17.64 | 3039 | 480.2813 | 7.06 | 4720 | 595.3190 | 7.76 | 6401 | 943.9347 | 5.05 |
| 1359 | 279.2313 | 18.76 | 3040 | 480.2814 | 6.68 | 4721 | 595.3192 | 9.45 | 6402 | 944.9453 | 8.04 |
| 1360 | 279.2337 | 17.63 | 3041 | 480.2835 | 7.24 | 4722 | 595.3217 | 9.51 | 6403 | 944.9526 | 5.04 |
| 1361 | 279.2343 | 21.37 | 3042 | 480.2835 | 6.50 | 4723 | 595.3219 | 8.01 | 6404 | 945.2011 | 8.04 |
| 1362 | 279.2345 | 17.49 | 3043 | 480.2843 | 6.77 | 4724 | 595.3231 | 8.84 | 6405 | 945.2014 | 21.21 |
| 1363 | 279.2347 | 17.58 | 3044 | 480.2851 | 6.57 | 4725 | 595.3235 | 9.27 | 6406 | 945.4537 | 8.05 |
| 1364 | 279.2349 | 17.55 | 3045 | 480.3061 | 15.85 | 4726 | 595.3259 | 8.49 | 6407 | 946.2010 | 21.17 |
| 1365 | 279.2357 | 15.48 | 3046 | 480.3074 | 16.17 | 4727 | 595.3276 | 8.34 | 6408 | 947.1991 | 21.17 |
| 1366 | 280.0861 | 16.63 | 3047 | 480.3079 | 19.51 | 4728 | 595.3518 | 12.11 | 6409 | 948.5129 | 8.61 |
| 1367 | 280.0915 | 0.98 | 3048 | 480.3173 | 16.15 | 4729 | 595.5617 | 8.34 | 6410 | 948.5163 | 8.75 |
| 1368 | 280.2362 | 17.64 | 3049 | 480.3232 | 14.61 | 4730 | 595.5696 | 9.35 | 6411 | 948.8046 | 0.82 |
| 1369 | 281.0807 | 16.06 | 3050 | 480.3235 | 14.50 | 4731 | 595.5702 | 9.47 | 6412 | 949.0212 | 8.61 |
| 1370 | 281.2434 | 19.05 | 3051 | 480.3256 | 14.76 | 4732 | 595.5702 | 8.49 | 6413 | 949.3100 | 0.79 |
| 1371 | 281.2435 | 19.06 | 3052 | 480.3259 | 14.62 | 4733 | 595.5703 | 7.76 | 6414 | 952.1611 | 0.78 |
| 1372 | 281.2451 | 18.46 | 3053 | 480.3309 | 17.60 | 4734 | 595.5716 | 8.85 | 6415 | 956.7849 | 0.78 |
| 1373 | 281.2458 | 19.20 | 3054 | 480.3382 | 17.65 | 4735 | 595.5725 | 9.36 | 6416 | 961.4856 | 10.09 |
| 1374 | 281.2461 | 19.00 | 3055 | 481.2440 | 10.11 | 4736 | 595.5729 | 9.51 | 6417 | 968.7949 | 0.74 |
| 1375 | 281.2462 | 18.95 | 3056 | 481.2575 | 10.59 | 4737 | 595.5782 | 7.74 | 6418 | 972.1023 | 8.81 |
| 1376 | 281.2482 | 19.15 | 3057 | 481.2589 | 10.61 | 4738 | 595.5788 | 7.89 | 6419 | 974.8125 | 0.79 |
| 1377 | 281.2483 | 18.98 | 3058 | 481.2598 | 8.84 | 4739 | 595.6501 | 10.59 | 6420 | 974.8161 | 1.02 |
| 1378 | 281.2485 | 19.10 | 3059 | 481.2626 | 9.24 | 4740 | 595.6560 | 10.64 | 6421 | 974.8191 | 0.76 |
| 1379 | 281.2509 | 18.24 | 3060 | 481.2629 | 8.94 | 4741 | 595.7992 | 8.26 | 6422 | 975.3241 | 0.84 |
| 1380 | 281.2522 | 18.38 | 3061 | 481.2650 | 8.65 | 4742 | 595.7994 | 8.10 | 6423 | 975.8203 | 0.75 |
| 1381 | 281.9953 | 0.97 | 3062 | 481.2780 | 7.04 | 4743 | 595.8020 | 9.94 | 6424 | 979.2594 | 10.74 |
| 1382 | 282.2523 | 19.10 | 3063 | 481.2832 | 6.77 | 4744 | 595.8068 | 9.80 | 6425 | 979.4625 | 10.74 |
| 1383 | 282.2794 | 18.40 | 3064 | 481.2900 | 6.82 | 4745 | 595.8070 | 9.99 | 6426 | 980.6156 | 10.42 |
| 1384 | 282.9037 | 0.69 | 3065 | 481.3130 | 19.51 | 4746 | 595.8078 | 7.76 | 6427 | 980.7883 | 10.51 |
| 1385 | 283.0406 | 2.21 | 3066 | 481.7520 | 10.09 | 4747 | 595.8081 | 8.47 | 6428 | 981.8048 | 10.96 |
| 1386 | 283.0656 | 18.59 | 3067 | 482.0454 | 8.79 | 4748 | 595.8084 | 9.97 | 6429 | 981.9539 | 6.97 |
| 1387 | 283.0943 | 6.20 | 3068 | 482.2667 | 10.59 | 4749 | 595.8157 | 10.02 | 6430 | 981.9616 | 10.93 |
| 1388 | 283.0974 | 6.40 | 3069 | 482.2993 | 8.79 | 4750 | 595.8169 | 7.88 | 6431 | 982.1445 | 10.95 |
| 1389 | 283.0975 | 6.37 | 3070 | 482.3178 | 17.27 | 4751 | 595.8173 | 9.51 | 6432 | 982.1528 | 10.92 |
| 1390 | 283.0977 | 5.94 | 3071 | 482.3219 | 17.47 | 4752 | 595.8185 | 9.52 | 6433 | 982.2992 | 10.96 |
| 1391 | 283.0992 | 5.77 | 3072 | 482.3224 | 14.94 | 4753 | 595.8187 | 8.84 | 6434 | 982.4716 | 10.93 |
| 1392 | 283.0998 | 6.51 | 3073 | 482.3230 | 14.95 | 4754 | 595.8192 | 9.27 | 6435 | 982.7855 | 0.81 |
| 1393 | 283.1014 | 5.76 | 3074 | 482.3239 | 14.78 | 4755 | 595.8226 | 8.81 | 6436 | 982.8086 | 0.78 |
| 1394 | 283.1026 | 5.99 | 3075 | 482.3248 | 17.44 | 4756 | 595.8252 | 9.45 | 6437 | 982.9619 | 6.97 |
| 1395 | 283.1027 | 5.98 | 3076 | 482.3253 | 14.97 | 4757 | 595.8261 | 7.74 | 6438 | 983.0785 | 8.82 |
| 1396 | 283.1605 | 1.11 | 3077 | 482.3255 | 18.07 | 4758 | 595.9219 | 0.89 | 6439 | 983.5785 | 8.79 |
| 1397 | 283.1730 | 8.53 | 3078 | 482.3266 | 14.86 | 4759 | 595.9900 | 10.63 | 6440 | 988.1513 | 11.21 |
| 1398 | 283.1764 | 8.35 | 3079 | 482.3271 | 15.04 | 4760 | 595.9943 | 10.59 | 6441 | 988.4975 | 11.20 |
| 1399 | 283.2018 | 10.17 | 3080 | 482.3277 | 21.21 | 4761 | 596.0755 | 9.49 | 6442 | 989.4871 | 11.42 |
| 1400 | 283.2574 | 19.32 | 3081 | 482.3518 | 19.52 | 4762 | 596.0843 | 8.82 | 6443 | 989.6077 | 11.47 |
| 1401 | 283.2601 | 19.17 | 3082 | 482.3580 | 15.97 | 4763 | 596.3005 | 8.47 | 6444 | 990.7842 | 0.79 |
| 1402 | 283.2602 | 17.78 | 3083 | 482.3584 | 15.96 | 4764 | 596.3018 | 9.94 | 6445 | 991.6703 | 15.62 |
| 1403 | 283.2602 | 19.46 | 3084 | 482.3628 | 16.12 | 4765 | 596.3056 | 8.10 | 6446 | 991.6711 | 18.84 |
| 1404 | 283.2624 | 17.87 | 3085 | 482.3658 | 16.04 | 4766 | 596.3056 | 9.81 | 6447 | 991.6732 | 15.67 |
| 1405 | 283.2625 | 18.03 | 3086 | 482.5319 | 8.75 | 4767 | 596.3063 | 9.99 | 6448 | 991.6757 | 15.74 |
| 1406 | 283.2638 | 19.74 | 3087 | 482.7732 | 8.77 | 4768 | 596.3082 | 10.02 | 6449 | 991.6802 | 15.53 |
| 1407 | 283.2647 | 17.77 | 3088 | 483.3222 | 21.18 | 4769 | 596.3083 | 8.69 | 6450 | 992.6683 | 15.67 |
| 1408 | 283.2648 | 17.71 | 3089 | 483.3265 | 18.07 | 4770 | 596.3088 | 8.26 | 6451 | 992.6725 | 15.52 |
| 1409 | 283.2649 | 18.37 | 3090 | 483.3291 | 14.96 | 4771 | 596.3098 | 9.90 | 6452 | 992.6741 | 15.49 |
| 1410 | 283.2651 | 19.59 | 3091 | 483.3291 | 17.47 | 4772 | 596.3100 | 9.97 | 6453 | 992.6751 | 18.84 |
| 1411 | 283.2672 | 17.88 | 3092 | 483.3638 | 16.10 | 4773 | 596.3151 | 8.27 | 6454 | 992.6799 | 18.85 |
| 1412 | 284.9568 | 12.32 | 3093 | 484.0979 | 16.26 | 4774 | 596.3275 | 10.63 | 6455 | 992.6832 | 15.62 |
| 1413 | 284.9581 | 12.17 | 3094 | 484.0984 | 7.73 | 4775 | 596.8186 | 8.26 | 6456 | 992.6848 | 15.71 |
| 1414 | 284.9582 | 15.22 | 3095 | 484.2365 | 11.66 | 4776 | 596.8196 | 9.99 | 6457 | 992.6899 | 15.68 |
| 1415 | 284.9592 | 12.33 | 3096 | 485.9595 | 0.98 | 4777 | 597.2682 | 17.53 | 6458 | 993.6576 | 15.74 |
| 1416 | 284.9626 | 12.52 | 3097 | 486.1104 | 0.89 | 4778 | 597.7754 | 17.50 | 6459 | 993.6744 | 15.62 |
| 1417 | 284.9651 | 12.70 | 3098 | 486.4427 | 7.99 | 4779 | 597.9002 | 8.23 | 6460 | 993.6756 | 18.87 |
| 1418 | 284.9657 | 15.18 | 3099 | 486.4459 | 7.72 | 4780 | 598.1032 | 8.25 | 6461 | 993.6770 | 15.52 |
| 1419 | 285.0026 | 0.90 | 3100 | 486.4491 | 7.96 | 4781 | 598.2755 | 17.50 | 6462 | 993.6856 | 15.68 |
| 1420 | 285.9891 | 0.87 | 3101 | 486.4492 | 8.01 | 4782 | 598.3162 | 10.85 | 6463 | 993.6959 | 15.65 |
| 1421 | 286.1992 | 11.16 | 3102 | 486.4497 | 7.74 | 4783 | 598.3162 | 9.39 | 6464 | 994.0897 | 8.81 |
| 1422 | 286.9518 | 12.19 | 3103 | 486.4533 | 8.24 | 4784 | 598.3179 | 7.85 | 6465 | 994.5633 | 9.38 |
| 1423 | 286.9542 | 12.70 | 3104 | 486.4559 | 9.38 | 4785 | 598.3209 | 8.19 | 6466 | 994.5834 | 8.82 |
| 1424 | 286.9566 | 12.52 | 3105 | 486.5491 | 8.79 | 4786 | 598.3220 | 7.91 | 6467 | 994.6580 | 18.84 |
| 1425 | 286.9596 | 15.18 | 3106 | 486.5494 | 9.28 | 4787 | 598.3222 | 9.74 | 6468 | 994.6862 | 15.68 |
| 1426 | 286.9608 | 12.36 | 3107 | 486.5497 | 7.86 | 4788 | 598.3227 | 9.10 | 6469 | 994.6868 | 15.67 |
| 1427 | 288.1947 | 21.36 | 3108 | 486.5517 | 7.70 | 4789 | 598.3229 | 9.35 | 6470 | 994.6939 | 15.53 |
| 1428 | 288.2166 | 12.10 | 3109 | 486.5535 | 9.40 | 4790 | 598.3233 | 9.18 | 6471 | 995.9754 | 7.50 |
| 1429 | 288.2182 | 10.26 | 3110 | 486.5537 | 9.72 | 4791 | 598.3238 | 7.65 | 6472 | 996.4702 | 10.89 |
| 1430 | 288.2194 | 9.86 | 3111 | 486.5538 | 8.47 | 4792 | 598.3243 | 9.29 | 6473 | 996.9912 | 10.89 |
| 1431 | 288.6350 | 15.16 | 3112 | 486.5559 | 9.43 | 4793 | 598.3245 | 9.02 | 6474 | 998.2892 | 19.73 |
| 1432 | 288.9212 | 0.73 | 3113 | 486.5935 | 9.38 | 4794 | 598.3265 | 8.47 | 6475 | 998.7812 | 0.74 |
| 1433 | 289.1522 | 18.77 | 3114 | 486.6450 | 7.72 | 4795 | 598.3287 | 9.30 | 6476 | 1001.1880 | 8.67 |
| 1434 | 289.1556 | 18.63 | 3115 | 486.6455 | 7.71 | 4796 | 598.3316 | 8.81 | 6477 | 1001.4012 | 8.68 |
| 1435 | 289.2163 | 13.28 | 3116 | 486.6463 | 8.22 | 4797 | 598.5646 | 8.47 | 6478 | 1001.4092 | 8.32 |
| 1436 | 289.2167 | 16.41 | 3117 | 486.6514 | 8.08 | 4798 | 598.5704 | 7.91 | 6479 | 1001.4132 | 8.50 |
| 1437 | 290.0738 | 1.01 | 3118 | 486.6515 | 8.01 | 4799 | 598.5722 | 7.65 | 6480 | 1001.6056 | 8.32 |
| 1438 | 290.0772 | 1.11 | 3119 | 486.6525 | 8.24 | 4800 | 598.5728 | 8.79 | 6481 | 1001.6165 | 8.67 |
| 1439 | 290.0773 | 1.39 | 3120 | 486.6573 | 7.91 | 4801 | 598.5732 | 7.85 | 6482 | 1001.6185 | 8.50 |
| 1440 | 290.0780 | 1.07 | 3121 | 486.7727 | 8.51 | 4802 | 598.5752 | 9.18 | 6483 | 1001.7975 | 8.32 |
| 1441 | 290.2706 | 13.92 | 3122 | 486.7956 | 9.27 | 4803 | 598.5764 | 9.03 | 6484 | 1001.8105 | 8.50 |
| 1442 | 290.5776 | 1.08 | 3123 | 486.7962 | 9.40 | 4804 | 598.5772 | 9.30 | 6485 | 1001.8173 | 8.67 |
| 1443 | 290.5851 | 1.01 | 3124 | 486.7983 | 7.74 | 4805 | 598.5775 | 9.74 | 6486 | 1003.2476 | 19.76 |
| 1444 | 291.0710 | 5.13 | 3125 | 486.7986 | 9.37 | 4806 | 598.5784 | 9.39 | 6487 | 1005.2354 | 19.69 |
| 1445 | 291.0725 | 5.43 | 3126 | 486.8012 | 8.79 | 4807 | 598.5797 | 8.19 | 6488 | 1005.8026 | 0.77 |
| 1446 | 293.0919 | 11.82 | 3127 | 486.8088 | 9.74 | 4808 | 598.8179 | 8.79 | 6489 | 1006.4997 | 9.55 |
| 1447 | 294.0013 | 1.04 | 3128 | 486.8454 | 8.24 | 4809 | 598.8193 | 9.15 | 6490 | 1006.7618 | 0.84 |
| 1448 | 294.0037 | 1.14 | 3129 | 486.8476 | 7.98 | 4810 | 598.8193 | 10.84 | 6491 | 1008.7984 | 0.82 |
| 1449 | 294.2062 | 19.05 | 3130 | 486.8506 | 7.95 | 4811 | 598.8214 | 9.02 | 6492 | 1008.8186 | 0.74 |
| 1450 | 294.9373 | 0.82 | 3131 | 486.8516 | 8.24 | 4812 | 598.8232 | 9.35 | 6493 | 1009.3150 | 0.79 |
| 1451 | 294.9394 | 0.77 | 3132 | 486.8533 | 7.91 | 4813 | 598.8234 | 9.39 | 6494 | 1009.8068 | 0.76 |
| 1452 | 295.0088 | 1.01 | 3133 | 486.8534 | 7.99 | 4814 | 598.8237 | 9.18 | 6495 | 1010.8851 | 9.86 |
| 1453 | 295.0962 | 17.75 | 3134 | 486.8537 | 8.01 | 4815 | 598.8247 | 8.19 | 6496 | 1011.1591 | 15.64 |
| 1454 | 295.1585 | 7.71 | 3135 | 486.8698 | 0.70 | 4816 | 598.8251 | 7.85 | 6497 | 1011.2215 | 9.86 |
| 1455 | 295.1650 | 6.67 | 3136 | 487.0279 | 8.51 | 4817 | 598.8276 | 7.65 | 6498 | 1011.4796 | 10.90 |
| 1456 | 295.1884 | 17.55 | 3137 | 487.0324 | 8.66 | 4818 | 598.8291 | 9.30 | 6499 | 1011.5489 | 9.86 |
| 1457 | 295.1941 | 21.60 | 3138 | 487.0447 | 8.22 | 4819 | 598.8292 | 7.91 | 6500 | 1011.6245 | 10.89 |
| 1458 | 295.6502 | 5.36 | 3139 | 487.0476 | 9.37 | 4820 | 598.8294 | 9.11 | 6501 | 1011.6358 | 8.91 |
| 1459 | 295.6527 | 5.53 | 3140 | 487.0483 | 9.40 | 4821 | 598.8295 | 9.74 | 6502 | 1011.8014 | 8.92 |
| 1460 | 295.6528 | 4.99 | 3141 | 487.0515 | 9.43 | 4822 | 598.8303 | 8.47 | 6503 | 1011.8026 | 10.90 |
| 1461 | 295.6529 | 5.12 | 3142 | 487.0537 | 8.81 | 4823 | 598.8344 | 0.81 | 6504 | 1011.8906 | 9.88 |
| 1462 | 295.6530 | 5.00 | 3143 | 487.0539 | 8.24 | 4824 | 598.8379 | 0.84 | 6505 | 1011.9730 | 10.93 |
| 1463 | 296.2007 | 21.60 | 3144 | 487.0560 | 8.08 | 4825 | 598.9962 | 0.79 | 6506 | 1012.1438 | 10.92 |
| 1464 | 297.0754 | 17.79 | 3145 | 487.0560 | 8.01 | 4826 | 599.0688 | 8.20 | 6507 | 1012.3098 | 10.92 |
| 1465 | 297.1847 | 9.35 | 3146 | 487.0571 | 9.27 | 4827 | 599.0688 | 9.18 | 6508 | 1013.6514 | 18.80 |
| 1466 | 297.1854 | 8.88 | 3147 | 487.0671 | 9.74 | 4828 | 599.0700 | 9.03 | 6509 | 1013.6516 | 15.62 |
| 1467 | 297.1878 | 9.21 | 3148 | 487.2740 | 8.50 | 4829 | 599.0718 | 9.35 | 6510 | 1013.6620 | 19.01 |
| 1468 | 297.1896 | 9.34 | 3149 | 487.3094 | 9.38 | 4830 | 599.0754 | 8.47 | 6511 | 1013.6625 | 15.43 |
| 1469 | 297.1921 | 9.31 | 3150 | 487.3121 | 8.81 | 4831 | 599.0754 | 9.39 | 6512 | 1013.6666 | 15.74 |
| 1470 | 297.1959 | 9.72 | 3151 | 487.5608 | 8.79 | 4832 | 599.0776 | 9.30 | 6513 | 1013.6693 | 18.56 |
| 1471 | 299.0186 | 0.86 | 3152 | 487.9410 | 8.61 | 4833 | 599.0780 | 9.74 | 6514 | 1014.1534 | 15.52 |
| 1472 | 299.1118 | 12.25 | 3153 | 488.2769 | 8.59 | 4834 | 599.0804 | 7.82 | 6515 | 1014.1547 | 18.86 |
| 1473 | 299.1123 | 14.94 | 3154 | 488.2935 | 15.86 | 4835 | 599.0807 | 7.93 | 6516 | 1014.1573 | 15.64 |
| 1474 | 299.1594 | 19.05 | 3155 | 488.9602 | 0.89 | 4836 | 599.0840 | 8.81 | 6517 | 1014.1675 | 15.75 |
| 1475 | 299.1785 | 15.57 | 3156 | 489.2242 | 9.98 | 4837 | 599.2466 | 17.57 | 6518 | 1014.6386 | 15.79 |
| 1476 | 299.1874 | 6.01 | 3157 | 489.2316 | 5.38 | 4838 | 599.2471 | 15.67 | 6519 | 1014.6447 | 15.49 |
| 1477 | 299.5119 | 6.01 | 3158 | 489.2752 | 8.56 | 4839 | 599.2499 | 17.48 | 6520 | 1014.6552 | 15.65 |
| 1478 | 301.1201 | 11.87 | 3159 | 489.2811 | 8.39 | 4840 | 599.3136 | 9.41 | 6521 | 1014.6582 | 15.55 |
| 1479 | 301.1387 | 19.68 | 3160 | 489.2827 | 8.73 | 4841 | 599.3153 | 8.81 | 6522 | 1014.6591 | 18.90 |
| 1480 | 301.1400 | 19.73 | 3161 | 489.3039 | 15.86 | 4842 | 599.3159 | 11.76 | 6523 | 1014.6621 | 15.46 |
| 1481 | 301.1401 | 19.72 | 3162 | 489.6518 | 9.41 | 4843 | 599.3208 | 9.18 | 6524 | 1014.6622 | 15.64 |
| 1482 | 301.1406 | 16.27 | 3163 | 489.7613 | 10.28 | 4844 | 599.3232 | 9.34 | 6525 | 1014.6637 | 18.83 |
| 1483 | 301.1407 | 19.74 | 3164 | 489.7789 | 8.73 | 4845 | 599.3302 | 9.72 | 6526 | 1014.6700 | 15.57 |
| 1484 | 301.1411 | 16.41 | 3165 | 489.7839 | 8.56 | 4846 | 599.4330 | 18.56 | 6527 | 1015.6455 | 15.62 |
| 1485 | 301.1420 | 19.65 | 3166 | 490.0111 | 10.28 | 4847 | 599.4351 | 18.89 | 6528 | 1015.6468 | 15.55 |
| 1486 | 301.1441 | 16.52 | 3167 | 490.2607 | 10.30 | 4848 | 599.4371 | 18.92 | 6529 | 1015.6513 | 15.52 |
| 1487 | 301.1443 | 19.66 | 3168 | 490.2863 | 14.55 | 4849 | 599.4390 | 18.81 | 6530 | 1015.6544 | 18.85 |
| 1488 | 301.1455 | 19.75 | 3169 | 490.2869 | 17.34 | 4850 | 599.4406 | 15.48 | 6531 | 1015.6564 | 15.74 |
| 1489 | 301.2101 | 21.34 | 3170 | 490.2869 | 14.34 | 4851 | 599.4442 | 15.71 | 6532 | 1015.6578 | 18.97 |
| 1490 | 301.2112 | 19.15 | 3171 | 490.2873 | 14.31 | 4852 | 599.4444 | 15.68 | 6533 | 1015.6635 | 15.67 |
| 1491 | 301.2114 | 19.32 | 3172 | 490.2895 | 14.20 | 4853 | 599.4448 | 15.64 | 6534 | 1015.6898 | 15.68 |
| 1492 | 301.2132 | 19.30 | 3173 | 490.2900 | 14.39 | 4854 | 599.5660 | 9.18 | 6535 | 1016.6510 | 15.69 |
| 1493 | 301.2138 | 19.17 | 3174 | 490.2935 | 17.38 | 4855 | 599.5720 | 9.72 | 6536 | 1016.8016 | 0.80 |
| 1494 | 301.2139 | 16.84 | 3175 | 490.2941 | 14.29 | 4856 | 599.5742 | 8.82 | 6537 | 1017.3063 | 0.88 |
| 1495 | 301.2168 | 19.22 | 3176 | 491.0238 | 0.85 | 4857 | 599.5756 | 9.38 | 6538 | 1017.6766 | 19.28 |
| 1496 | 301.2177 | 20.40 | 3177 | 491.2904 | 17.36 | 4858 | 599.8223 | 9.38 | 6539 | 1017.6995 | 18.97 |
| 1497 | 301.2186 | 16.72 | 3178 | 492.3859 | 19.43 | 4859 | 600.2513 | 17.44 | 6540 | 1018.6851 | 18.97 |
| 1498 | 302.1046 | 11.89 | 3179 | 492.4965 | 8.75 | 4860 | 600.2518 | 17.60 | 6541 | 1018.6939 | 19.29 |
| 1499 | 302.1417 | 16.39 | 3180 | 492.5004 | 8.59 | 4861 | 600.3003 | 8.84 | 6542 | 1019.6232 | 15.75 |
| 1500 | 302.1431 | 19.69 | 3181 | 492.5012 | 8.97 | 4862 | 600.3042 | 9.13 | 6543 | 1019.6250 | 18.84 |
| 1501 | 302.1449 | 16.27 | 3182 | 492.7548 | 8.98 | 4863 | 600.3070 | 8.96 | 6544 | 1019.6282 | 15.65 |
| 1502 | 302.1450 | 19.67 | 3183 | 492.8850 | 0.73 | 4864 | 600.3184 | 10.61 | 6545 | 1020.1344 | 18.85 |
| 1503 | 302.1460 | 16.52 | 3184 | 492.9990 | 8.97 | 4865 | 600.3210 | 10.55 | 6546 | 1020.1409 | 15.67 |
| 1504 | 302.1474 | 19.75 | 3185 | 493.5278 | 11.18 | 4866 | 600.3216 | 10.57 | 6547 | 1020.1433 | 0.78 |
| 1505 | 302.1503 | 19.65 | 3186 | 493.5304 | 9.21 | 4867 | 600.4328 | 15.64 | 6548 | 1020.1468 | 15.49 |
| 1506 | 302.2032 | 8.90 | 3187 | 493.7746 | 10.93 | 4868 | 600.4345 | 18.90 | 6549 | 1020.6335 | 15.62 |
| 1507 | 302.2098 | 9.19 | 3188 | 493.7754 | 11.19 | 4869 | 600.4422 | 18.83 | 6550 | 1020.6372 | 18.91 |
| 1508 | 302.9187 | 17.80 | 3189 | 494.0262 | 11.18 | 4870 | 600.4525 | 15.43 | 6551 | 1021.1458 | 15.67 |
| 1509 | 303.0233 | 5.12 | 3190 | 494.2666 | 8.59 | 4871 | 600.6498 | 9.06 | 6552 | 1024.7816 | 0.75 |
| 1510 | 303.0244 | 5.34 | 3191 | 494.2668 | 8.66 | 4872 | 600.6597 | 10.55 | 6553 | 1029.6215 | 15.69 |
| 1511 | 303.0256 | 5.63 | 3192 | 494.3186 | 14.68 | 4873 | 600.6605 | 10.58 | 6554 | 1030.1325 | 11.16 |
| 1512 | 303.2313 | 21.18 | 3193 | 494.3214 | 17.60 | 4874 | 600.6640 | 10.61 | 6555 | 1030.3062 | 9.03 |
| 1513 | 303.2322 | 17.46 | 3194 | 494.3214 | 17.66 | 4875 | 600.9925 | 10.61 | 6556 | 1030.3138 | 10.95 |
| 1514 | 303.2340 | 19.17 | 3195 | 494.3219 | 14.83 | 4876 | 601.2474 | 17.57 | 6557 | 1030.3253 | 11.17 |
| 1515 | 303.5468 | 14.23 | 3196 | 494.3219 | 14.71 | 4877 | 601.3061 | 8.96 | 6558 | 1030.3255 | 11.15 |
| 1516 | 304.1612 | 5.27 | 3197 | 494.3221 | 17.68 | 4878 | 601.3098 | 8.82 | 6559 | 1030.3343 | 9.40 |
| 1517 | 304.1624 | 5.53 | 3198 | 494.3228 | 14.64 | 4879 | 601.3137 | 9.13 | 6560 | 1030.4627 | 11.06 |
| 1518 | 304.1627 | 5.00 | 3199 | 494.3233 | 17.78 | 4880 | 601.3279 | 10.61 | 6561 | 1030.4839 | 9.15 |
| 1519 | 304.1639 | 4.88 | 3200 | 494.3239 | 17.74 | 4881 | 602.3129 | 7.44 | 6562 | 1030.4840 | 11.15 |
| 1520 | 304.1647 | 5.10 | 3201 | 494.3249 | 14.76 | 4882 | 602.3130 | 7.63 | 6563 | 1030.4897 | 10.93 |
| 1521 | 304.1649 | 5.37 | 3202 | 494.3250 | 17.57 | 4883 | 602.3163 | 7.15 | 6564 | 1030.4903 | 11.11 |
| 1522 | 304.1651 | 5.06 | 3203 | 494.3257 | 14.61 | 4884 | 602.3165 | 7.46 | 6565 | 1030.4925 | 9.41 |
| 1523 | 304.1682 | 5.72 | 3204 | 494.3305 | 17.71 | 4885 | 603.2650 | 9.48 | 6566 | 1030.4932 | 11.20 |
| 1524 | 304.6644 | 5.53 | 3205 | 494.3307 | 17.73 | 4886 | 603.7925 | 9.31 | 6567 | 1030.4967 | 11.12 |
| 1525 | 304.6696 | 5.00 | 3206 | 494.7857 | 18.88 | 4887 | 603.7996 | 9.14 | 6568 | 1030.5010 | 9.04 |
| 1526 | 304.8934 | 0.73 | 3207 | 494.7981 | 15.76 | 4888 | 604.2913 | 9.34 | 6569 | 1030.6265 | 15.69 |
| 1527 | 304.9153 | 17.79 | 3208 | 494.8009 | 15.69 | 4889 | 604.2916 | 9.11 | 6570 | 1030.6442 | 11.11 |
| 1528 | 305.1546 | 7.70 | 3209 | 494.8036 | 15.52 | 4890 | 604.3160 | 10.76 | 6571 | 1030.6506 | 11.12 |
| 1529 | 305.1582 | 8.45 | 3210 | 494.8049 | 15.64 | 4891 | 604.6606 | 10.75 | 6572 | 1030.6515 | 11.15 |
| 1530 | 305.1585 | 8.53 | 3211 | 495.3215 | 17.80 | 4892 | 604.6999 | 8.82 | 6573 | 1030.6560 | 9.15 |
| 1531 | 305.1591 | 8.36 | 3212 | 495.3226 | 14.68 | 4893 | 604.7841 | 9.31 | 6574 | 1030.6568 | 10.92 |
| 1532 | 305.1749 | 13.36 | 3213 | 495.3227 | 14.83 | 4894 | 605.0434 | 8.81 | 6575 | 1030.6595 | 9.04 |
| 1533 | 305.1860 | 10.17 | 3214 | 495.3275 | 14.62 | 4895 | 606.0014 | 9.04 | 6576 | 1030.6602 | 9.40 |
| 1534 | 305.2389 | 18.47 | 3215 | 495.3290 | 14.71 | 4896 | 606.2877 | 11.92 | 6577 | 1030.6608 | 11.23 |
| 1535 | 305.2457 | 18.19 | 3216 | 495.3316 | 17.69 | 4897 | 606.2939 | 15.12 | 6578 | 1030.8106 | 9.18 |
| 1536 | 305.2493 | 18.05 | 3217 | 495.8087 | 18.81 | 4898 | 606.3417 | 9.04 | 6579 | 1030.8185 | 9.41 |
| 1537 | 306.1810 | 13.36 | 3218 | 496.2378 | 6.19 | 4899 | 606.6717 | 9.04 | 6580 | 1030.8198 | 10.92 |
| 1538 | 307.2256 | 13.41 | 3219 | 496.2766 | 18.89 | 4900 | 606.6734 | 9.15 | 6581 | 1030.8208 | 11.11 |
| 1539 | 307.2266 | 13.63 | 3220 | 496.2830 | 18.75 | 4901 | 606.8002 | 9.89 | 6582 | 1030.8224 | 9.03 |
| 1540 | 307.2277 | 16.42 | 3221 | 496.3033 | 21.34 | 4902 | 606.8586 | 0.69 | 6583 | 1030.8283 | 11.20 |
| 1541 | 307.2285 | 13.30 | 3222 | 496.3162 | 20.98 | 4903 | 607.0087 | 9.04 | 6584 | 1030.8318 | 11.12 |
| 1542 | 307.2288 | 13.28 | 3223 | 496.3225 | 20.82 | 4904 | 607.1258 | 10.94 | 6585 | 1030.9647 | 10.92 |
| 1543 | 307.2487 | 17.39 | 3224 | 496.3245 | 20.94 | 4905 | 607.2845 | 11.93 | 6586 | 1030.9732 | 11.20 |
| 1544 | 308.1029 | 1.19 | 3225 | 496.3257 | 20.91 | 4906 | 607.3138 | 5.77 | 6587 | 1030.9812 | 9.13 |
| 1545 | 308.1650 | 11.87 | 3226 | 496.3289 | 20.91 | 4907 | 607.3153 | 5.84 | 6588 | 1030.9866 | 9.15 |
| 1546 | 308.1924 | 21.59 | 3227 | 496.3292 | 21.02 | 4908 | 607.3166 | 5.29 | 6589 | 1030.9866 | 11.15 |
| 1547 | 308.2304 | 16.36 | 3228 | 496.3296 | 20.74 | 4909 | 607.3175 | 5.00 | 6590 | 1030.9906 | 9.41 |
| 1548 | 308.2318 | 16.49 | 3229 | 496.3350 | 20.97 | 4910 | 607.3184 | 5.36 | 6591 | 1030.9975 | 11.11 |
| 1549 | 308.2333 | 13.28 | 3230 | 496.3353 | 21.11 | 4911 | 607.3194 | 4.88 | 6592 | 1031.1414 | 10.92 |
| 1550 | 308.2334 | 13.51 | 3231 | 496.3362 | 15.42 | 4912 | 607.3200 | 4.64 | 6593 | 1031.1477 | 9.06 |
| 1551 | 309.1283 | 15.90 | 3232 | 496.3366 | 15.67 | 4913 | 607.3203 | 5.53 | 6594 | 1031.1491 | 9.41 |
| 1552 | 309.1710 | 11.87 | 3233 | 496.3379 | 15.72 | 4914 | 607.3205 | 4.76 | 6595 | 1031.1634 | 11.20 |
| 1553 | 309.1764 | 1.17 | 3234 | 496.3383 | 20.95 | 4915 | 607.3205 | 4.99 | 6596 | 1031.3134 | 11.14 |
| 1554 | 309.1795 | 1.14 | 3235 | 496.3385 | 21.15 | 4916 | 607.3206 | 5.52 | 6597 | 1031.4982 | 11.17 |
| 1555 | 309.2009 | 17.23 | 3236 | 496.3386 | 21.02 | 4917 | 607.3212 | 5.03 | 6598 | 1034.3091 | 11.20 |
| 1556 | 309.2010 | 20.93 | 3237 | 496.3387 | 21.20 | 4918 | 607.3218 | 5.10 | 6599 | 1035.6886 | 11.36 |
| 1557 | 309.2021 | 17.17 | 3238 | 496.3390 | 15.37 | 4919 | 607.3238 | 4.74 | 6600 | 1035.9123 | 11.34 |
| 1558 | 309.2061 | 20.99 | 3239 | 496.3392 | 15.48 | 4920 | 607.3239 | 10.96 | 6601 | 1036.0926 | 11.36 |
| 1559 | 309.2082 | 17.25 | 3240 | 496.3396 | 18.85 | 4921 | 607.3242 | 5.72 | 6602 | 1036.7715 | 0.76 |
| 1560 | 309.2108 | 17.38 | 3241 | 496.3399 | 18.51 | 4922 | 607.8103 | 9.37 | 6603 | 1039.6657 | 19.28 |
| 1561 | 309.2445 | 17.97 | 3242 | 496.3400 | 17.77 | 4923 | 607.8145 | 8.06 | 6604 | 1039.6762 | 18.26 |
| 1562 | 310.1776 | 1.15 | 3243 | 496.3401 | 15.64 | 4924 | 607.8184 | 8.22 | 6605 | 1039.6856 | 15.16 |
| 1563 | 310.9155 | 0.80 | 3244 | 496.3404 | 18.72 | 4925 | 608.0484 | 8.21 | 6606 | 1039.7814 | 0.78 |
| 1564 | 312.1957 | 6.11 | 3245 | 496.3405 | 18.73 | 4926 | 608.0535 | 7.99 | 6607 | 1040.6803 | 19.32 |
| 1565 | 312.1973 | 21.37 | 3246 | 496.3406 | 21.24 | 4927 | 608.0540 | 9.40 | 6608 | 1040.6956 | 18.23 |
| 1566 | 313.0296 | 0.77 | 3247 | 496.3421 | 21.30 | 4928 | 608.0546 | 7.74 | 6609 | 1041.6880 | 18.80 |
| 1567 | 313.1534 | 8.18 | 3248 | 496.3423 | 15.43 | 4929 | 608.2945 | 8.84 | 6610 | 1041.8095 | 8.07 |
| 1568 | 313.1539 | 10.03 | 3249 | 496.3428 | 15.22 | 4930 | 608.3112 | 8.01 | 6611 | 1042.5030 | 9.58 |
| 1569 | 313.1545 | 8.42 | 3250 | 496.3440 | 18.70 | 4931 | 608.3128 | 8.21 | 6612 | 1042.5054 | 11.45 |
| 1570 | 313.1545 | 8.47 | 3251 | 496.3443 | 15.26 | 4932 | 608.3198 | 5.05 | 6613 | 1042.7988 | 0.78 |
| 1571 | 313.1546 | 9.59 | 3252 | 496.3555 | 20.91 | 4933 | 608.3220 | 4.99 | 6614 | 1042.8073 | 0.79 |
| 1572 | 313.1550 | 9.85 | 3253 | 496.3952 | 18.84 | 4934 | 608.3224 | 5.00 | 6615 | 1042.8218 | 9.58 |
| 1573 | 313.1557 | 8.20 | 3254 | 496.4040 | 15.79 | 4935 | 608.3224 | 5.56 | 6616 | 1042.8383 | 11.39 |
| 1574 | 313.1562 | 9.83 | 3255 | 496.4081 | 18.99 | 4936 | 608.3233 | 5.36 | 6617 | 1043.3084 | 0.80 |
| 1575 | 313.1564 | 8.79 | 3256 | 496.4112 | 18.99 | 4937 | 608.3252 | 5.53 | 6618 | 1043.7037 | 19.32 |
| 1576 | 313.1568 | 9.96 | 3257 | 496.4333 | 19.03 | 4938 | 608.3278 | 4.88 | 6619 | 1043.8083 | 0.83 |
| 1577 | 313.1569 | 8.50 | 3258 | 497.2665 | 11.68 | 4939 | 608.3291 | 5.72 | 6620 | 1044.7020 | 19.31 |
| 1578 | 313.1570 | 9.88 | 3259 | 497.2772 | 18.86 | 4940 | 608.3802 | 11.41 | 6621 | 1047.7242 | 17.18 |
| 1579 | 313.1571 | 8.40 | 3260 | 497.2979 | 19.02 | 4941 | 608.3819 | 9.49 | 6622 | 1047.7372 | 17.15 |
| 1580 | 313.1578 | 8.23 | 3261 | 497.3005 | 19.03 | 4942 | 608.3882 | 9.38 | 6623 | 1047.7491 | 20.90 |
| 1581 | 313.1583 | 8.93 | 3262 | 497.3043 | 18.95 | 4943 | 608.3924 | 9.62 | 6624 | 1048.7264 | 20.90 |
| 1582 | 313.1595 | 8.42 | 3263 | 497.3349 | 20.95 | 4944 | 609.3256 | 8.32 | 6625 | 1048.7474 | 17.24 |
| 1583 | 313.1648 | 8.42 | 3264 | 497.3403 | 18.73 | 4945 | 609.3351 | 5.00 | 6626 | 1050.0550 | 10.93 |
| 1584 | 313.1670 | 8.42 | 3265 | 497.3404 | 21.25 | 4946 | 609.8049 | 8.59 | 6627 | 1050.5715 | 10.93 |
| 1585 | 313.2210 | 8.63 | 3266 | 497.3410 | 15.26 | 4947 | 610.3081 | 18.27 | 6628 | 1050.5724 | 11.22 |
| 1586 | 313.2762 | 18.62 | 3267 | 497.3414 | 21.11 | 4948 | 610.3112 | 8.58 | 6629 | 1050.8000 | 0.75 |
| 1587 | 314.0367 | 15.74 | 3268 | 497.3419 | 21.01 | 4949 | 610.6988 | 8.46 | 6630 | 1050.8018 | 0.80 |
| 1588 | 314.0368 | 0.90 | 3269 | 497.3423 | 15.62 | 4950 | 610.7004 | 8.59 | 6631 | 1058.7691 | 0.82 |
| 1589 | 314.0386 | 15.62 | 3270 | 497.3425 | 18.85 | 4951 | 610.7005 | 7.41 | 6632 | 1058.7814 | 0.79 |
| 1590 | 314.1551 | 8.18 | 3271 | 497.3425 | 15.49 | 4952 | 610.7009 | 7.95 | 6633 | 1061.6294 | 18.26 |
| 1591 | 314.1562 | 8.42 | 3272 | 497.3451 | 15.37 | 4953 | 610.7012 | 7.62 | 6634 | 1061.6664 | 15.14 |
| 1592 | 314.1576 | 8.17 | 3273 | 497.3452 | 15.48 | 4954 | 610.7019 | 7.27 | 6635 | 1062.5558 | 10.10 |
| 1593 | 314.1587 | 9.86 | 3274 | 497.3452 | 15.43 | 4955 | 610.7052 | 8.39 | 6636 | 1062.6695 | 15.18 |
| 1594 | 314.1587 | 9.59 | 3275 | 497.3453 | 15.41 | 4956 | 610.7064 | 8.79 | 6637 | 1062.6866 | 18.26 |
| 1595 | 314.1606 | 8.77 | 3276 | 497.3457 | 15.22 | 4957 | 610.7074 | 8.58 | 6638 | 1063.6659 | 15.21 |
| 1596 | 314.1734 | 9.96 | 3277 | 497.3458 | 15.67 | 4958 | 610.7086 | 7.80 | 6639 | 1063.6924 | 18.24 |
| 1597 | 314.1802 | 10.00 | 3278 | 497.3459 | 18.51 | 4959 | 610.7091 | 7.32 | 6640 | 1064.6909 | 18.22 |
| 1598 | 314.1851 | 10.05 | 3279 | 497.3464 | 18.72 | 4960 | 610.9853 | 10.93 | 6641 | 1065.6942 | 19.34 |
| 1599 | 315.0092 | 0.86 | 3280 | 497.3470 | 21.34 | 4961 | 610.9976 | 11.12 | 6642 | 1065.6960 | 16.06 |
| 1600 | 315.1347 | 14.59 | 3281 | 497.3488 | 15.71 | 4962 | 611.0306 | 8.79 | 6643 | 1066.6797 | 19.34 |
| 1601 | 315.1372 | 13.25 | 3282 | 497.3699 | 21.01 | 4963 | 611.0333 | 7.78 | 6644 | 1069.7224 | 20.91 |
| 1602 | 315.1938 | 15.26 | 3283 | 497.4527 | 19.27 | 4964 | 611.0361 | 8.74 | 6645 | 1069.7301 | 17.23 |
| 1603 | 315.1956 | 15.52 | 3284 | 497.4558 | 9.07 | 4965 | 611.0370 | 8.46 | 6646 | 1070.7283 | 20.93 |
| 1604 | 315.1960 | 15.39 | 3285 | 497.4628 | 18.32 | 4966 | 611.0389 | 8.61 | 6647 | 1070.7314 | 17.23 |
| 1605 | 315.1973 | 18.44 | 3286 | 497.4642 | 17.55 | 4967 | 611.0393 | 7.62 | 6648 | 1074.7412 | 0.80 |
| 1606 | 315.2014 | 6.52 | 3287 | 498.3382 | 18.70 | 4968 | 611.0400 | 7.27 | 6649 | 1076.7885 | 0.76 |
| 1607 | 315.2017 | 6.77 | 3288 | 498.3427 | 15.37 | 4969 | 611.0402 | 7.32 | 6650 | 1076.8041 | 0.79 |
| 1608 | 315.2020 | 7.00 | 3289 | 498.3428 | 15.48 | 4970 | 611.0426 | 7.95 | 6651 | 1077.2915 | 0.80 |
| 1609 | 315.2030 | 7.07 | 3290 | 498.3461 | 15.42 | 4971 | 611.0426 | 8.56 | 6652 | 1077.7864 | 0.82 |
| 1610 | 315.2041 | 7.08 | 3291 | 498.3465 | 18.84 | 4972 | 611.0456 | 7.41 | 6653 | 1079.8975 | 10.24 |
| 1611 | 315.2057 | 7.23 | 3292 | 498.3485 | 15.53 | 4973 | 611.2856 | 18.97 | 6654 | 1081.6535 | 18.80 |
| 1612 | 315.7102 | 21.61 | 3293 | 498.3485 | 15.55 | 4974 | 611.3299 | 10.92 | 6655 | 1084.7792 | 0.79 |
| 1613 | 316.2478 | 11.02 | 3294 | 498.3489 | 21.32 | 4975 | 611.3323 | 11.12 | 6656 | 1085.6657 | 15.17 |
| 1614 | 316.2478 | 13.42 | 3295 | 498.3491 | 18.51 | 4976 | 611.3672 | 8.66 | 6657 | 1085.7072 | 18.26 |
| 1615 | 316.2508 | 11.38 | 3296 | 498.3493 | 15.62 | 4977 | 611.3688 | 8.79 | 6658 | 1086.6540 | 18.24 |
| 1616 | 316.2520 | 11.17 | 3297 | 498.3494 | 15.69 | 4978 | 611.3698 | 8.59 | 6659 | 1087.7098 | 18.22 |
| 1617 | 316.2539 | 10.91 | 3298 | 498.3497 | 15.67 | 4979 | 611.3738 | 8.56 | 6660 | 1092.7873 | 0.78 |
| 1618 | 317.1738 | 21.58 | 3299 | 498.3499 | 15.23 | 4980 | 611.3739 | 7.42 | 6661 | 1095.5820 | 11.55 |
| 1619 | 317.2494 | 13.42 | 3300 | 498.3540 | 18.70 | 4981 | 611.3741 | 7.62 | 6662 | 1096.1049 | 11.55 |
| 1620 | 318.6774 | 7.15 | 3301 | 498.4630 | 19.27 | 4982 | 611.5338 | 9.38 | 6663 | 1096.5860 | 11.55 |
| 1621 | 319.0726 | 18.25 | 3302 | 498.8967 | 1.04 | 4983 | 611.7116 | 8.59 | 6664 | 1097.0904 | 11.55 |
| 1622 | 319.1257 | 11.89 | 3303 | 498.9000 | 0.86 | 4984 | 611.7903 | 9.72 | 6665 | 1102.3218 | 10.65 |
| 1623 | 319.2662 | 13.43 | 3304 | 498.9020 | 0.82 | 4985 | 611.8065 | 8.81 | 6666 | 1102.5226 | 10.63 |
| 1624 | 319.2679 | 11.38 | 3305 | 498.9021 | 0.78 | 4986 | 611.8087 | 9.37 | 6667 | 1104.9462 | 11.21 |
| 1625 | 319.2684 | 10.86 | 3306 | 498.9425 | 9.14 | 4987 | 612.0535 | 8.59 | 6668 | 1105.1244 | 11.21 |
| 1626 | 319.2685 | 11.30 | 3307 | 498.9432 | 9.17 | 4988 | 612.0625 | 9.38 | 6669 | 1105.3392 | 11.14 |
| 1627 | 319.2691 | 11.17 | 3308 | 498.9437 | 7.51 | 4989 | 612.8824 | 0.73 | 6670 | 1105.5371 | 11.21 |
| 1628 | 319.2693 | 13.42 | 3309 | 498.9469 | 9.08 | 4990 | 613.3390 | 9.62 | 6671 | 1105.7421 | 11.20 |
| 1629 | 319.2693 | 11.01 | 3310 | 498.9473 | 8.75 | 4991 | 613.3391 | 11.40 | 6672 | 1110.7842 | 0.78 |
| 1630 | 320.0760 | 18.22 | 3311 | 498.9494 | 9.09 | 4992 | 613.5561 | 8.23 | 6673 | 1110.7895 | 0.80 |
| 1631 | 320.2733 | 13.50 | 3312 | 498.9507 | 7.78 | 4993 | 613.5631 | 8.07 | 6674 | 1111.3028 | 0.81 |
| 1632 | 321.0364 | 15.31 | 3313 | 499.2797 | 9.14 | 4994 | 613.8112 | 8.23 | 6675 | 1111.8100 | 0.80 |
| 1633 | 321.0711 | 18.25 | 3314 | 499.2815 | 7.78 | 4995 | 613.8112 | 8.07 | 6676 | 1115.0432 | 8.46 |
| 1634 | 321.2447 | 19.22 | 3315 | 499.2841 | 8.77 | 4996 | 614.0593 | 8.23 | 6677 | 1115.0440 | 8.29 |
| 1635 | 322.0705 | 18.26 | 3316 | 499.3238 | 20.40 | 4997 | 614.0663 | 8.07 | 6678 | 1115.5480 | 8.29 |
| 1636 | 322.1592 | 11.17 | 3317 | 499.3414 | 15.48 | 4998 | 614.3040 | 8.07 | 6679 | 1115.5599 | 8.44 |
| 1637 | 322.1784 | 12.00 | 3318 | 499.3471 | 15.55 | 4999 | 614.3150 | 8.22 | 6680 | 1115.5660 | 8.46 |
| 1638 | 323.1340 | 5.54 | 3319 | 499.3515 | 15.67 | 5000 | 614.3440 | 17.19 | 6681 | 1116.0435 | 8.35 |
| 1639 | 323.1395 | 5.00 | 3320 | 499.3515 | 18.83 | 5001 | 614.3444 | 20.88 | 6682 | 1116.0654 | 8.46 |
| 1640 | 323.1868 | 12.00 | 3321 | 499.3544 | 18.85 | 5002 | 614.3530 | 9.82 | 6683 | 1118.7857 | 0.80 |
| 1641 | 324.1401 | 13.78 | 3322 | 499.6106 | 9.14 | 5003 | 614.3540 | 12.06 | 6684 | 1126.7581 | 0.80 |
| 1642 | 324.1402 | 16.72 | 3323 | 499.6145 | 9.17 | 5004 | 614.5590 | 8.25 | 6685 | 1140.0075 | 11.38 |
| 1643 | 324.1537 | 6.94 | 3324 | 499.6218 | 8.78 | 5005 | 614.5633 | 8.22 | 6686 | 1140.2113 | 11.36 |
| 1644 | 324.1539 | 6.91 | 3325 | 499.9044 | 0.81 | 5006 | 614.5697 | 8.07 | 6687 | 1142.0270 | 10.93 |
| 1645 | 324.1563 | 6.84 | 3326 | 499.9053 | 0.77 | 5007 | 616.1422 | 18.28 | 6688 | 1142.5228 | 10.93 |
| 1646 | 324.1591 | 6.64 | 3327 | 500.2719 | 18.37 | 5008 | 616.1465 | 17.65 | 6689 | 1144.7770 | 0.79 |
| 1647 | 324.1917 | 12.02 | 3328 | 500.2723 | 15.20 | 5009 | 616.1773 | 21.38 | 6690 | 1144.7787 | 0.81 |
| 1648 | 324.1921 | 5.59 | 3329 | 500.2785 | 17.60 | 5010 | 616.1773 | 16.40 | 6691 | 1144.7820 | 0.75 |
| 1649 | 325.1319 | 0.96 | 3330 | 500.9086 | 0.77 | 5011 | 616.1879 | 16.43 | 6692 | 1145.2987 | 0.76 |
| 1650 | 325.1442 | 16.74 | 3331 | 502.2874 | 15.27 | 5012 | 616.3988 | 11.18 | 6693 | 1152.1463 | 11.86 |
| 1651 | 325.2116 | 18.46 | 3332 | 502.2897 | 18.32 | 5013 | 616.8174 | 0.68 | 6694 | 1152.6326 | 10.09 |
| 1652 | 325.2128 | 18.24 | 3333 | 502.2903 | 15.21 | 5014 | 618.0278 | 8.59 | 6695 | 1152.6370 | 9.72 |
| 1653 | 325.2128 | 18.27 | 3334 | 502.2910 | 15.12 | 5015 | 618.3540 | 8.59 | 6696 | 1152.6550 | 11.89 |
| 1654 | 325.2131 | 18.32 | 3335 | 502.2938 | 15.24 | 5016 | 618.4563 | 8.05 | 6697 | 1152.7827 | 0.78 |
| 1655 | 325.2221 | 19.15 | 3336 | 502.2962 | 18.27 | 5017 | 618.7051 | 8.61 | 6698 | 1153.1483 | 11.89 |
| 1656 | 325.2331 | 13.28 | 3337 | 502.2964 | 15.34 | 5018 | 619.2478 | 17.52 | 6699 | 1153.6362 | 11.91 |
| 1657 | 325.2362 | 16.44 | 3338 | 502.2967 | 19.51 | 5019 | 621.2353 | 17.51 | 6700 | 1163.5939 | 11.89 |
| 1658 | 326.1838 | 1.73 | 3339 | 503.2889 | 18.33 | 5020 | 622.2826 | 8.96 | 6701 | 1166.3099 | 6.45 |
| 1659 | 326.2381 | 8.94 | 3340 | 503.2906 | 15.23 | 5021 | 622.8337 | 0.70 | 6702 | 1166.3130 | 4.97 |
| 1660 | 326.2436 | 9.29 | 3341 | 503.3025 | 10.95 | 5022 | 622.8465 | 0.71 | 6703 | 1166.7755 | 4.97 |
| 1661 | 326.2467 | 9.16 | 3342 | 503.9437 | 9.66 | 5023 | 623.0848 | 8.16 | 6704 | 1178.1513 | 11.14 |
| 1662 | 327.0068 | 13.77 | 3343 | 503.9448 | 8.16 | 5024 | 623.0881 | 10.19 | 6705 | 1178.3532 | 11.12 |
| 1663 | 327.0071 | 14.01 | 3344 | 503.9451 | 9.88 | 5025 | 623.0909 | 8.47 | 6706 | 1178.5714 | 11.18 |
| 1664 | 327.0087 | 14.22 | 3345 | 503.9461 | 9.41 | 5026 | 623.0923 | 8.76 | 6707 | 1178.7834 | 0.79 |
| 1665 | 327.0105 | 17.07 | 3346 | 503.9465 | 7.82 | 5027 | 623.0932 | 8.30 | 6708 | 1178.7886 | 0.75 |
| 1666 | 327.0108 | 17.10 | 3347 | 503.9471 | 9.59 | 5028 | 623.0942 | 10.04 | 6709 | 1179.2951 | 0.78 |
| 1667 | 327.0117 | 0.94 | 3348 | 503.9491 | 8.42 | 5029 | 623.0953 | 10.17 | 6710 | 1185.2484 | 5.00 |
| 1668 | 327.0128 | 0.91 | 3349 | 503.9499 | 8.92 | 5030 | 623.0982 | 8.09 | 6711 | 1185.2674 | 6.51 |
| 1669 | 327.0525 | 0.79 | 3350 | 504.1891 | 0.99 | 5031 | 623.3349 | 8.74 | 6712 | 1185.2735 | 7.04 |
| 1670 | 327.0723 | 18.61 | 3351 | 504.2772 | 8.06 | 5032 | 623.3419 | 8.16 | 6713 | 1185.7455 | 6.38 |
| 1671 | 327.0748 | 15.49 | 3352 | 504.2773 | 8.14 | 5033 | 623.3423 | 8.26 | 6714 | 1185.7825 | 5.00 |
| 1672 | 327.0840 | 15.61 | 3353 | 504.2794 | 9.80 | 5034 | 623.3438 | 10.07 | 6715 | 1185.7834 | 7.04 |
| 1673 | 327.1998 | 9.16 | 3354 | 504.2798 | 9.63 | 5035 | 623.3445 | 8.47 | 6716 | 1186.7662 | 0.79 |
| 1674 | 327.2012 | 8.07 | 3355 | 504.2798 | 9.69 | 5036 | 623.3447 | 8.09 | 6717 | 1192.7383 | 6.45 |
| 1675 | 327.2036 | 8.10 | 3356 | 504.2799 | 9.58 | 5037 | 623.3487 | 10.19 | 6718 | 1192.7496 | 4.98 |
| 1676 | 327.2048 | 7.85 | 3357 | 504.2816 | 8.42 | 5038 | 623.4444 | 18.23 | 6719 | 1193.2529 | 6.44 |
| 1677 | 327.2227 | 19.47 | 3358 | 504.2818 | 9.41 | 5039 | 623.5880 | 8.15 | 6720 | 1193.2680 | 5.01 |
| 1678 | 327.2263 | 19.71 | 3359 | 504.2822 | 7.82 | 5040 | 623.5880 | 8.08 | 6721 | 1194.7424 | 0.84 |
| 1679 | 327.2272 | 19.78 | 3360 | 504.2855 | 7.86 | 5041 | 623.5881 | 10.19 | 6722 | 1194.7723 | 0.81 |
| 1680 | 327.2273 | 19.76 | 3361 | 504.2962 | 17.43 | 5042 | 623.5909 | 10.14 | 6723 | 1196.1044 | 9.35 |
| 1681 | 327.2277 | 19.61 | 3362 | 504.3047 | 14.87 | 5043 | 623.5920 | 8.74 | 6724 | 1196.6524 | 9.37 |

**Supporting Information Table 3** Candidate metabolites for the LMIs listed in Tables 2–4.

| **Mass value (***m/z***)*** | **Retention time (min)** | **Table no.** | **Compound** | **Name** | | **Formula** | **Monoisotopic Mass** | **Adduct** | **Adduct**  m/z | **Delta (ppm)**** |
| --- | --- | --- | --- | --- | --- | --- | --- | --- | --- | --- |
| **283.2018** | **10.17** | **2** | HMDB0035695 | Vitamin A2 aldehyde | | C20H26O | 282.1984 | M+H | 283.2056 | 14 |
|  |  |  | HMDB0040643 | Juvocimene 1 | | C20H26O | 282.1984 | M+H | 283.2056 | 14 |
|  |  |  | HMDB0033630 | Lactapiperanol C | | C16H26O4 | 282.1831 | M+H | 283.1904 | 40 |
|  |  |  | HMDB0030360 | (+)-Aspidospermidine | | C19H26N2 | 282.2096 | M+H | 283.2169 | 53 |
|  |  |  | HMDB0030260 | (-)-Quebrachamine | | C19H26N2 | 282.2096 | M+H | 283.2169 | 53 |
|  |  |  | HMDB0060992 | 2-hydroxydesipramine | | C18H22N2O | 282.1732 | M+H | 283.1805 | 75 |
|  |  |  | HMDB0031799 | Malonoben | | C18H22N2O | 282.1732 | M+H | 283.1805 | 75 |
|  |  |  | HMDB0061822 | Hexaethylene glycol | | C12H26O7 | 282.1679 | M+H | 283.1751 | 94 |
|  |  |  |  |  | |  |  |  |  |  |
| **305.186** | **10.17** | **2** | HMDB0060379 | 3-Polyprenyl-4-hydroxy-5-methoxybenzoate | | C18H24O4 | 304.1675 | M+H | 305.1747 | 37 |
|  |  |  | HMDB0060352 | 2-Polyprenyl-3-methyl-5-hydroxy-6-methoxy-1,4-benzoquinone | | C18H24O4 | 304.1675 | M+H | 305.1747 | 37 |
|  |  |  | HMDB0000309 | 3a,16b-Dihydroxyandrostenone | | C19H28O3 | 304.2038 | M+H | 305.2111 | 82 |
|  |  |  | HMDB0060089 | w Hydroxy testosterone | | C19H28O3 | 304.2038 | M+H | 305.2111 | 82 |
|  |  |  | HMDB0060339 | 11beta,17beta-Dihydroxy-4-androsten-3-one | | C19H28O3 | 304.2038 | M+H | 305.2111 | 82 |
|  |  |  | HMDB0012654 | 2beta-Hydroxytestosterone | | C19H28O3 | 304.2038 | M+H | 305.2111 | 82 |
|  |  |  | HMDB0012533 | 11beta-Hydroxytestosterone | | C19H28O3 | 304.2038 | M+H | 305.2111 | 82 |
|  |  |  | HMDB0000322 | 16-Oxoandrostenediol | | C19H28O3 | 304.2038 | M+H | 305.2111 | 82 |
|  |  |  | HMDB0000324 | 3a,16a-Dihydroxyandrostenone | | C19H28O3 | 304.2038 | M+H | 305.2111 | 82 |
|  |  |  | HMDB0000352 | 16a-Hydroxydehydroisoandrosterone | | C19H28O3 | 304.2038 | M+H | 305.2111 | 82 |
|  |  |  | HMDB0000388 | 3a,16-Dihydroxyandrostenone | | C19H28O3 | 304.2038 | M+H | 305.2111 | 82 |
|  |  |  | HMDB0003956 | 7a-Hydroxytestosterone | | C19H28O3 | 304.2038 | M+H | 305.2111 | 82 |
|  |  |  | HMDB0004611 | 7a-Hydroxydehydroepiandrosterone | | C19H28O3 | 304.2038 | M+H | 305.2111 | 82 |
|  |  |  | HMDB0004624 | 7b-Hydroxydehydroepiandrosterone | | C19H28O3 | 304.2038 | M+H | 305.2111 | 82 |
|  |  |  | HMDB0006031 | 11-Ketoetiocholanolone | | C19H28O3 | 304.2038 | M+H | 305.2111 | 82 |
|  |  |  | HMDB0006259 | 6beta-Hydroxytestosterone | | C19H28O3 | 304.2038 | M+H | 305.2111 | 82 |
|  |  |  | HMDB0006769 | 19-Hydroxytestosterone | | C19H28O3 | 304.2038 | M+H | 305.2111 | 82 |
|  |  |  | HMDB0039589 | Ginsenoyne G | | C19H28O3 | 304.2038 | M+H | 305.2111 | 82 |
|  |  |  | HMDB0039735 | 10-Acetoxy-8-heptadecene-4,6-diyn-3-ol | | C19H28O3 | 304.2038 | M+H | 305.2111 | 82 |
|  |  |  | HMDB0031464 | cis-[8]-Shogaol | | C19H28O3 | 304.2038 | M+H | 305.2111 | 82 |
|  |  |  |  |  | |  |  |  |  |  |
| **394.2633** | **10.8** | **2** | HMDB0038618 | Acidissiminol | | C25H31NO3 | 393.2304 | M+H | 394.2377 | 65 |
|  |  |  |  |  | |  |  |  |  |  |
| **505.3229** | **11.56** | **2** | HMDB0015110 | Dipyridamole | | C24H40N8O4 | 504.3173 | M+H | 505.3245 | 3 |
|  |  |  | HMDB0033709 | Desglucocoroloside | | C29H44O7 | 504.3087 | M+H | 505.316 | 14 |
|  |  |  | HMDB0013021 | Neuromedin N (1-4) | | C26H40N4O6 | 504.2948 | M+H | 505.3021 | 41 |
|  |  |  | HMDB0040784 | (3beta,19alpha)-3,19,23,24-Tetrahydroxy-12-oleanen-28-oic acid | | C30H48O6 | 504.3451 | M+H | 505.3524 | 58 |
|  |  |  | HMDB0040772 | Isothankunic acid | | C30H48O6 | 504.3451 | M+H | 505.3524 | 58 |
|  |  |  | HMDB0035487 | (1beta,2alpha,3alpha)-1,2,3,24-Tetrahydroxy-12-oleanen-28-oic acid | | C30H48O6 | 504.3451 | M+H | 505.3524 | 58 |
|  |  |  | HMDB0035875 | Myrianthic acid | | C30H48O6 | 504.3451 | M+H | 505.3524 | 58 |
|  |  |  | HMDB0036670 | 6beta-Hydroxyasiatic acid | | C30H48O6 | 504.3451 | M+H | 505.3524 | 58 |
|  |  |  | HMDB0034500 | Protobassic acid | | C30H48O6 | 504.3451 | M+H | 505.3524 | 58 |
|  |  |  | HMDB0034518 | Theasapogenol E | | C30H48O6 | 504.3451 | M+H | 505.3524 | 58 |
|  |  |  | HMDB0035948 | Cyclopassifloic acid D | | C30H48O6 | 504.3451 | M+H | 505.3524 | 58 |
|  |  |  | HMDB0034501 | Tomentosic acid | | C30H48O6 | 504.3451 | M+H | 505.3524 | 58 |
|  |  |  | HMDB0029339 | Ceanothine B | | C29H36N4O4 | 504.2737 | M+H | 505.2809 | 83 |
|  |  |  | HMDB0029618 | Macrophorin D | | C28H40O8 | 504.2723 | M+H | 505.2796 | 86 |
|  |  |  | HMDB0034393 | Ixocarpalactone A | | C28H40O8 | 504.2723 | M+H | 505.2796 | 86 |
|  |  |  |  |  | |  |  |  |  |  |
| **505.3401** | **11.03** | **2** | HMDB0036670 | 6beta-Hydroxyasiatic acid | | C30H48O6 | 504.3451 | M+H | 505.3524 | 24 |
|  |  |  | HMDB0034500 | Protobassic acid | | C30H48O6 | 504.3451 | M+H | 505.3524 | 24 |
|  |  |  | HMDB0035487 | (1beta,2alpha,3alpha)-1,2,3,24-Tetrahydroxy-12-oleanen-28-oic acid | | C30H48O6 | 504.3451 | M+H | 505.3524 | 24 |
|  |  |  | HMDB0034501 | Tomentosic acid | | C30H48O6 | 504.3451 | M+H | 505.3524 | 24 |
|  |  |  | HMDB0035948 | Cyclopassifloic acid D | | C30H48O6 | 504.3451 | M+H | 505.3524 | 24 |
|  |  |  | HMDB0034518 | Theasapogenol E | | C30H48O6 | 504.3451 | M+H | 505.3524 | 24 |
|  |  |  | HMDB0040784 | (3beta,19alpha)-3,19,23,24-Tetrahydroxy-12-oleanen-28-oic acid | | C30H48O6 | 504.3451 | M+H | 505.3524 | 24 |
|  |  |  | HMDB0040772 | Isothankunic acid | | C30H48O6 | 504.3451 | M+H | 505.3524 | 24 |
|  |  |  | HMDB0035875 | Myrianthic acid | | C30H48O6 | 504.3451 | M+H | 505.3524 | 24 |
|  |  |  | HMDB0015110 | Dipyridamole | | C24H40N8O4 | 504.3173 | M+H | 505.3245 | 31 |
|  |  |  | HMDB0033709 | Desglucocoroloside | | C29H44O7 | 504.3087 | M+H | 505.316 | 48 |
|  |  |  | HMDB0013021 | Neuromedin N (1-4) | | C26H40N4O6 | 504.2948 | M+H | 505.3021 | 75 |
|  |  |  | HMDB0037780 | Ganoderiol G | | C31H52O5 | 504.3815 | M+H | 505.3888 | 96 |
|  |  |  |  |  | |  |  |  |  |  |
| **505.3405** | **10.93** | **2** | HMDB0036670 | 6beta-Hydroxyasiatic acid | | C30H48O6 | 504.3451 | M+H | 505.3524 | 23 |
|  |  |  | HMDB0034500 | Protobassic acid | | C30H48O6 | 504.3451 | M+H | 505.3524 | 23 |
|  |  |  | HMDB0035487 | (1beta,2alpha,3alpha)-1,2,3,24-Tetrahydroxy-12-oleanen-28-oic acid | | C30H48O6 | 504.3451 | M+H | 505.3524 | 23 |
|  |  |  | HMDB0034501 | Tomentosic acid | | C30H48O6 | 504.3451 | M+H | 505.3524 | 23 |
|  |  |  | HMDB0035948 | Cyclopassifloic acid D | | C30H48O6 | 504.3451 | M+H | 505.3524 | 23 |
|  |  |  | HMDB0034518 | Theasapogenol E | | C30H48O6 | 504.3451 | M+H | 505.3524 | 23 |
|  |  |  | HMDB0040784 | (3beta,19alpha)-3,19,23,24-Tetrahydroxy-12-oleanen-28-oic acid | | C30H48O6 | 504.3451 | M+H | 505.3524 | 23 |
|  |  |  | HMDB0040772 | Isothankunic acid | | C30H48O6 | 504.3451 | M+H | 505.3524 | 23 |
|  |  |  | HMDB0035875 | Myrianthic acid | | C30H48O6 | 504.3451 | M+H | 505.3524 | 23 |
|  |  |  | HMDB0015110 | Dipyridamole | | C24H40N8O4 | 504.3173 | M+H | 505.3245 | 32 |
|  |  |  | HMDB0033709 | Desglucocoroloside | | C29H44O7 | 504.3087 | M+H | 505.316 | 49 |
|  |  |  | HMDB0013021 | Neuromedin N (1-4) | | C26H40N4O6 | 504.2948 | M+H | 505.3021 | 76 |
|  |  |  | HMDB0037780 | Ganoderiol G | | C31H52O5 | 504.3815 | M+H | 505.3888 | 95 |
|  |  |  |  |  | |  |  |  |  |  |
| **527.3245** | **11.39** | **2** | HMDB0035386 | (24E)-3alpha-Acetoxy-15alpha-hydroxy-23-oxo-7,9(11),24-lanostatrien-26-oic acid | | C32H46O6 | 526.3294 | M+H | 527.3367 | 23 |
|  |  |  | HMDB0035387 | (24E)-15alpha-Acetoxy-3alpha-hydroxy-23-oxo-7,9(11),24-lanostatrien-26-oic acid | | C32H46O6 | 526.3294 | M+H | 527.3367 | 23 |
|  |  |  | HMDB0000572 | Desmosine | | C24H40N5O8 | 526.2877 | M+H | 527.295 | 56 |
|  |  |  | HMDB0000739 | Isodesmosine | | C24H40N5O8 | 526.2877 | M+H | 527.295 | 56 |
|  |  |  | HMDB0036673 | 3alpha-Acetomethoxy-11alpha-oxo-12-ursen-24-oic acid | | C33H50O5 | 526.3658 | M+H | 527.3731 | 92 |
|  |  |  |  |  | |  |  |  |  |  |
| **527.3246** | **11.03** | **2** | HMDB0035386 | (24E)-3alpha-Acetoxy-15alpha-hydroxy-23-oxo-7,9(11),24-lanostatrien-26-oic acid | | C32H46O6 | 526.3294 | M+H | 527.3367 | 23 |
|  |  |  | HMDB0035387 | (24E)-15alpha-Acetoxy-3alpha-hydroxy-23-oxo-7,9(11),24-lanostatrien-26-oic acid | | C32H46O6 | 526.3294 | M+H | 527.3367 | 23 |
|  |  |  | HMDB0000572 | Desmosine | | C24H40N5O8 | 526.2877 | M+H | 527.295 | 56 |
|  |  |  | HMDB0000739 | Isodesmosine | | C24H40N5O8 | 526.2877 | M+H | 527.295 | 56 |
|  |  |  | HMDB0036673 | 3alpha-Acetomethoxy-11alpha-oxo-12-ursen-24-oic acid | | C33H50O5 | 526.3658 | M+H | 527.3731 | 92 |
|  |  |  |  |  | |  |  |  |  |  |
| **555.2872** | **11.56** | **2** | HMDB0003828 | D-Pantethine | | C22H42N4O8S2 | 554.2444 | M+H | 555.2517 | 64 |
|  |  |  | HMDB0038934 | Ssioriside | | C27H38O12 | 554.2363 | M+H | 555.2436 | 79 |
|  |  |  |  |  | |  |  |  |  |  |
| **594.3589** | **11.74** | **2** | **No results for this query mass** | | | | | | | |
|  |  |  |  | |  |  |  |  |  |  |
| **599.3159** | **11.76** | **2** | HMDB0061693 | | 1-Oleoylglycerophosphoinositol | C27H51O12P | 598.3118 | M+H | 599.3191 | 5 |
|  |  |  | HMDB0000668 | | Hematoporphyrin IX | C34H38N4O6 | 598.2791 | M+H | 599.2864 | 49 |
|  |  |  | HMDB0039579 | | Sucrose monopalmitate | C28H54O13 | 598.3564 | M+H | 599.3637 | 80 |
|  |  |  |  | |  |  |  |  |  |  |
| **616.3988** | **11.18** | **2** | **No results for this query mass** | | | | | | | |
|  |  |  |  | |  |  |  |  |  |  |
| **638.3749** | **11.89** | **2** | HMDB0035264 | | Avenestergenin A1 | C38H55NO7 | 637.3979 | M+H | 638.4051 | 47 |
|  |  |  |  | |  |  |  |  |  |  |
| **643.3331** | **11.89** | **2** | HMDB0036707 | | Steviobioside | C32H50O13 | 642.3251 | M+H | 643.3324 | 1 |
|  |  |  |  | |  |  |  |  |  |  |
| **472.2419** | **10.18** | **3** | HMDB0060629 | | Hydroxydesmthyl doxepin glucuronide | C25H29NO8 | 471.1893 | M+H | 472.1966 | 96 |
|  |  |  | HMDB0060700 | | (E)-2-Hydroxydoxepin glucuronide | C25H29NO8 | 471.1893 | M+H | 472.1966 | 96 |
|  |  |  |  | |  |  |  |  |  |  |
| **496.3405** | **18.73** | **3** | HMDB0010382 | | LysoPC(16:0) | C24H50NO7P | 495.3325 | M+H | 496.3398 | 1 |
|  |  |  |  | |  |  |  |  |  |  |
| **497.3403** | **18.73** | **3** | HMDB0061702 | | 2-Palmitoylglycerophosphocholine | C24H51NO7P | 496.3403 | M+H | 497.3476 | 15 |
|  |  |  | HMDB0005772 | | Postin | C22H40N8O5 | 496.3122 | M+H | 497.3194 | 42 |
|  |  |  | HMDB0041331 | | (3b,16b,20R)-Pregn-5-ene-3,16,20-triol 3-glucoside | C27H44O8 | 496.3036 | M+H | 497.3109 | 59 |
|  |  |  | HMDB0010352 | | 3-alpha,20-alpha-Dihydroxy-5-beta-pregnane 3-glucuronide | C27H44O8 | 496.3036 | M+H | 497.3109 | 59 |
|  |  |  | HMDB0010318 | | Pregnanediol-3-glucuronide | C27H44O8 | 496.3036 | M+H | 497.3109 | 59 |
|  |  |  |  | |  |  |  |  |  |  |
| **498.3499** | **15.23** | **3** | **No results for this query mass** | | | | | | | |
|  |  |  |  | |  |  |  |  |  |  |
| **544.3365** | **18.15** | **3** | HMDB0010395 | | LysoPC(20:4(5Z,8Z,11Z,14Z)) | C28H50NO7P | 543.3325 | M+H | 544.3398 | 6 |
|  |  |  | HMDB0010396 | | LysoPC(20:4(8Z,11Z,14Z,17Z)) | C28H50NO7P | 543.3325 | M+H | 544.3398 | 6 |
|  |  |  |  | |  |  |  |  |  |  |
| **544.3384** | **14.98** | **3** | HMDB0010395 | | LysoPC(20:4(5Z,8Z,11Z,14Z)) | C28H50NO7P | 543.3325 | M+H | 544.3398 | 3 |
|  |  |  | HMDB0010396 | | LysoPC(20:4(8Z,11Z,14Z,17Z)) | C28H50NO7P | 543.3325 | M+H | 544.3398 | 3 |
|  |  |  |  | |  |  |  |  |  |  |
| **181.0714** | **7.73** | **4** | HMDB0001889 | | Theophylline | C7H8N4O2 | 180.0647 | M+H | 181.072 | 3 |
|  |  |  | HMDB0001860 | | Paraxanthine | C7H8N4O2 | 180.0647 | M+H | 181.072 | 3 |
|  |  |  | HMDB0002825 | | Theobromine | C7H8N4O2 | 180.0647 | M+H | 181.072 | 3 |
|  |  |  | HMDB0062473 | | Glycoprotein-phospho-D-mannose | C6H12O6 | 180.0634 | M+H | 181.0707 | 4 |
|  |  |  | HMDB0033704 | | L-Galactose | C6H12O6 | 180.0634 | M+H | 181.0707 | 4 |
|  |  |  | HMDB0034220 | | Levoinositol | C6H12O6 | 180.0634 | M+H | 181.0707 | 4 |
|  |  |  | HMDB0062202 | | 3(S)-hydroxy-all-cis-8,11,14,17-eicosatetraenoyl-CoA | C6H12O6 | 180.0634 | M+H | 181.0707 | 4 |
|  |  |  | HMDB0032222 | | Dihydroxyacetone (dimer) | C6H12O6 | 180.0634 | M+H | 181.0707 | 4 |
|  |  |  | HMDB0062538 | | Fructose-1P | C6H12O6 | 180.0634 | M+H | 181.0707 | 4 |
|  |  |  | HMDB0000516 | | Beta-D-Glucose | C6H12O6 | 180.0634 | M+H | 181.0707 | 4 |
|  |  |  | HMDB0000346 | | 3-Deoxyarabinohexonic acid | C6H12O6 | 180.0634 | M+H | 181.0707 | 4 |
|  |  |  | HMDB0000211 | | Myo-inositol | C6H12O6 | 180.0634 | M+H | 181.0707 | 4 |
|  |  |  | HMDB0000169 | | D-Mannose | C6H12O6 | 180.0634 | M+H | 181.0707 | 4 |
|  |  |  | HMDB0000143 | | D-Galactose | C6H12O6 | 180.0634 | M+H | 181.0707 | 4 |
|  |  |  | HMDB0000122 | | D-Glucose | C6H12O6 | 180.0634 | M+H | 181.0707 | 4 |
|  |  |  | HMDB0003418 | | D-Tagatose | C6H12O6 | 180.0634 | M+H | 181.0707 | 4 |
|  |  |  | HMDB0000660 | | D-Fructose | C6H12O6 | 180.0634 | M+H | 181.0707 | 4 |
|  |  |  | HMDB0062170 | | D-Glucopyranoside | C6H12O6 | 180.0634 | M+H | 181.0707 | 4 |
|  |  |  | HMDB0012326 | | L-Gulose | C6H12O6 | 180.0634 | M+H | 181.0707 | 4 |
|  |  |  | HMDB0006088 | | Scyllitol | C6H12O6 | 180.0634 | M+H | 181.0707 | 4 |
|  |  |  | HMDB0003449 | | Beta-D-Galactose | C6H12O6 | 180.0634 | M+H | 181.0707 | 4 |
|  |  |  | HMDB0003345 | | Alpha-D-Glucose | C6H12O6 | 180.0634 | M+H | 181.0707 | 4 |
|  |  |  | HMDB0001151 | | Allose | C6H12O6 | 180.0634 | M+H | 181.0707 | 4 |
|  |  |  | HMDB0001266 | | L-Sorbose | C6H12O6 | 180.0634 | M+H | 181.0707 | 4 |
|  |  |  | HMDB0061922 | | Alpha-D-Glucopyranoside | C6H12O6 | 180.0634 | M+H | 181.0707 | 4 |
|  |  |  | HMDB0012883 | | Adrenochrome o-semiquinone | C9H10NO3 | 180.0661 | M+H | 181.0733 | 11 |
|  |  |  | HMDB0038915 | | 12-Tridecene-4,6,8,10-tetraynal | C13H8O | 180.0575 | M+H | 181.0648 | 37 |
|  |  |  | HMDB0029430 | | Methionine sulfoximine | C5H12N2O3S | 180.0569 | M+H | 181.0641 | 40 |
|  |  |  | HMDB0041912 | | Isonicotinylglycine | C8H8N2O3 | 180.0535 | M+H | 181.0608 | 59 |
|  |  |  | HMDB0003269 | | Nicotinuric acid | C8H8N2O3 | 180.0535 | M+H | 181.0608 | 59 |
|  |  |  | HMDB0059766 | | Picolinoylglycine | C8H8N2O3 | 180.0535 | M+H | 181.0608 | 59 |
|  |  |  | HMDB0127492 | | 4-ethenyl-2,6-dimethoxyphenol | C10H12O3 | 180.0786 | M+H | 181.0859 | 80 |
|  |  |  | HMDB0135240 | | 3-methoxy-5-(prop-2-en-1-yl)benzene-1,2-diol | C10H12O3 | 180.0786 | M+H | 181.0859 | 80 |
|  |  |  | HMDB0133505 | | 4-(3,4-dihydroxyphenyl)butan-2-one | C10H12O3 | 180.0786 | M+H | 181.0859 | 80 |
|  |  |  | HMDB0129958 | | 2-(4-methoxyphenyl)propanoic acid | C10H12O3 | 180.0786 | M+H | 181.0859 | 80 |
|  |  |  | HMDB0060734 | | 3-(3-Hydroxyphenyl)-2-methylpropionic acid | C10H12O3 | 180.0786 | M+H | 181.0859 | 80 |
|  |  |  | HMDB0032174 | | Benzaldehyde glyceryl acetal | C10H12O3 | 180.0786 | M+H | 181.0859 | 80 |
|  |  |  | HMDB0012915 | | Coniferyl alcohol | C10H12O3 | 180.0786 | M+H | 181.0859 | 80 |
|  |  |  | HMDB0032128 | | 1-(2,4-Dihydroxyphenyl)-1-butanone | C10H12O3 | 180.0786 | M+H | 181.0859 | 80 |
|  |  |  | HMDB0011751 | | 3-Methoxybenzenepropanoic acid | C10H12O3 | 180.0786 | M+H | 181.0859 | 80 |
|  |  |  | HMDB0037602 | | 4-Hydroxy-2,6,6-trimethyl-3-oxo-1,4-cyclohexadiene-1-carboxaldehyde | C10H12O3 | 180.0786 | M+H | 181.0859 | 80 |
|  |  |  | HMDB0032574 | | Propylparaben | C10H12O3 | 180.0786 | M+H | 181.0859 | 80 |
|  |  |  | HMDB0037733 | | Propyl 2-furanacrylate | C10H12O3 | 180.0786 | M+H | 181.0859 | 80 |
|  |  |  | HMDB0029685 | | 4-Ethoxy-3-methoxybenzaldehyde | C10H12O3 | 180.0786 | M+H | 181.0859 | 80 |
|  |  |  | HMDB0032640 | | Ethyl p-anisate | C10H12O3 | 180.0786 | M+H | 181.0859 | 80 |
|  |  |  | HMDB0033761 | | 4-Methoxybenzyl acetate | C10H12O3 | 180.0786 | M+H | 181.0859 | 80 |
|  |  |  | HMDB0031240 | | 2-Phenyl-1,3-dioxolane-4-methanol | C10H12O3 | 180.0786 | M+H | 181.0859 | 80 |
|  |  |  | HMDB0131174 | | 3-(4-methoxyphenyl)propanoic acid | C10H12O3 | 180.0786 | M+H | 181.0859 | 80 |
|  |  |  |  | |  |  |  |  |  |  |
| **380.7359** | **7.03** | **4** | **No results for this query mass** | | | | | | | |
|  |  |  |  | |  |  |  |  |  |  |
| **381.733** | **6.94** | **4** | **No results for this query mass** | | | | | | | |
|  |  |  |  | |  |  |  |  |  |  |
| **630.3031** | **10.01** | **4** | **No results for this query mass** | | | | | | | |
|  |  |  |  | |  |  |  |  |  |  |
| **657.3503** | **10.32** | **4** | HMDB0034433 | | Riboflavine 2',3',4',5'-tetrabutanoate | C33H44N4O10 | 656.3057 | M+H | 657.313 | 57 |
|  |  |  |  | |  |  |  |  |  |  |
| **657.6682** | **10.32** | **4** | **No results for this query mass** | | | | | | | |
|  |  |  |  | |  |  |  |  |  |  |
| **712.9321** | **8.55** | **4** | **No results for this query mass** | | | | | | | |
|  |  |  |  | |  |  |  |  |  |  |
| **756.1579** | **9.99** | **4** | HMDB0031974 | | Malvidin 6-(6-p-coumaroylglucoside)-4-vinylphenol | C40H35O15 | 755.1976 | M+H | 756.2049 | 62 |
|  |  |  |  | |  |  |  |  |  |  |
| **756.3643** | **10.02** | **4** | **No results for this query mass** | | | | | | | |
|  |  |  |  | |  |  |  |  |  |  |
| **756.5703** | **10.01** | **4** | HMDB0007882 | | PC(14:0/20:3(8Z,11Z,14Z)) | C42H78NO8P | 755.5465 | M+H | 756.5538 | 22 |
|  |  |  | HMDB0008199 | | PC(18:3(9Z,12Z,15Z)/16:0) | C42H78NO8P | 755.5465 | M+H | 756.5538 | 22 |
|  |  |  | HMDB0008166 | | PC(18:3(6Z,9Z,12Z)/16:0) | C42H78NO8P | 755.5465 | M+H | 756.5538 | 22 |
|  |  |  | HMDB0008006 | | PC(16:1(9Z)/18:2(9Z,12Z)) | C42H78NO8P | 755.5465 | M+H | 756.5538 | 22 |
|  |  |  | HMDB0008329 | | PC(20:2(11Z,14Z)/14:1(9Z)) | C42H78NO8P | 755.5465 | M+H | 756.5538 | 22 |
|  |  |  | HMDB0008361 | | PC(20:3(5Z,8Z,11Z)/14:0) | C42H78NO8P | 755.5465 | M+H | 756.5538 | 22 |
|  |  |  | HMDB0008394 | | PC(20:3(8Z,11Z,14Z)/14:0) | C42H78NO8P | 755.5465 | M+H | 756.5538 | 22 |
|  |  |  | HMDB0007974 | | PC(16:0/18:3(6Z,9Z,12Z)) | C42H78NO8P | 755.5465 | M+H | 756.5538 | 22 |
|  |  |  | HMDB0007975 | | PC(16:0/18:3(9Z,12Z,15Z)) | C42H78NO8P | 755.5465 | M+H | 756.5538 | 22 |
|  |  |  | HMDB0007913 | | PC(14:1(9Z)/20:2(11Z,14Z)) | C42H78NO8P | 755.5465 | M+H | 756.5538 | 22 |
|  |  |  | HMDB0007881 | | PC(14:0/20:3(5Z,8Z,11Z)) | C42H78NO8P | 755.5465 | M+H | 756.5538 | 22 |
|  |  |  | HMDB0008134 | | PC(18:2(9Z,12Z)/16:1(9Z)) | C42H78NO8P | 755.5465 | M+H | 756.5538 | 22 |
|  |  |  | HMDB0009282 | | PE(20:1(11Z)/P-18:1(9Z)) | C43H82NO7P | 755.5829 | M+H | 756.5902 | 26 |
|  |  |  | HMDB0009313 | | PE(20:2(11Z,14Z)/P-18:0) | C43H82NO7P | 755.5829 | M+H | 756.5902 | 26 |
|  |  |  | HMDB0009576 | | PE(22:2(13Z,16Z)/P-16:0) | C43H82NO7P | 755.5829 | M+H | 756.5902 | 26 |
|  |  |  | HMDB0011357 | | PE(P-16:0/22:2(13Z,16Z)) | C43H82NO7P | 755.5829 | M+H | 756.5902 | 26 |
|  |  |  | HMDB0011414 | | PE(P-18:1(11Z)/20:1(11Z)) | C43H82NO7P | 755.5829 | M+H | 756.5902 | 26 |
|  |  |  | HMDB0011447 | | PE(P-18:1(9Z)/20:1(11Z)) | C43H82NO7P | 755.5829 | M+H | 756.5902 | 26 |
|  |  |  | HMDB0011382 | | PE(P-18:0/20:2(11Z,14Z)) | C43H82NO7P | 755.5829 | M+H | 756.5902 | 26 |
|  |  |  | HMDB0009281 | | PE(20:1(11Z)/P-18:1(11Z)) | C43H82NO7P | 755.5829 | M+H | 756.5902 | 26 |

*Mass value (*m/z*) information used when searching for candidate metabolites in the Human Metabolome Database (HMDB; http://www.hmdb.ca). Search conditions: **Mass tolerance ± 100 ppm and H^+^ adduct, only in the positive mode.

**Supporting Information Table 4** Differences in logarithmic peak areas (Fig. 5).

Groups with n < 10 were not considered. ns not significant, HRG high-risk group, PC pancreatic cancer, BTC biliary tract cancer, CRC colorectal cancer, OVC ovarian cancer. ^a^Mean ± standard deviation, ^b^mean age of all subjects in the two groups.

| **log_10_(496.3405 *m/z*, 18.73 min)** | | |  | **log_10_(544.3365 *m/z*, 18.15 min)** | | |
| --- | --- | --- | --- | --- | --- | --- |
| A) Total | | | | | | |
| Control  (n=100) | PC/BTC/CRC/OVC (n=220) | *p* |  | Control  (n=100) | PC/BTC/CRC/OVC (n=220) | *p* |
| 6.798 ± 0.431^a^ | 6.666 ± 0.289 | <.001 |  | 5.497 ± 0.417 | 5.431 ± 0.264 | <.001 |
| PC/BTC  (n=90) | Control/CRC/OVC (n=230) | *p* |  | PC/BTC  (n=90) | Control/CRC/OVC (n=230) | *p* |
| 6.664 ± 0.277 | 6.724 ± 0.367 | <.001 |  | 5.445 ± 0.276 | 5.454 ± 0.337 | .043 |
| Control (n=100) | PC/BTC (n=90) | *p* |  | Control (n=100) | PC/BTC (n=90) | *p* |
| 6.798 ± 0.431 | 6.664 ± 0.277 | <.001 |  | 5.497 ± 0.417 | 5.445 ± 0.276 | <.001 |
| PC (n=51) | BTC (n=39) | *p* |  | PC (n=51) | BTC (n=39) | *p* |
| 6.739 ± 0.249 | 6.567 ± 0.284 | .008 |  | 5.536 ± 0.271 | 5.326 ± 0.236 | .001 |
| PC/BTC-Cancers (n=54) | PC/BTC-HRG (n=36) | *p* |  | PC/BTC-Cancers (n=54) | PC/BTC-HRG  (n=36) | *p* |
| 6.606 ± 0.320 | 6.752 ± 0.163 | .018 |  | 5.409 ± 0.308 | 5.500 ± 0.212 | ns |
| PC-Cancers (n=40) | PC-HRG (n=11) | *p* |  | PC-Cancers (n=40) | PC-HRG (n=11) | *p* |
| 6.703 ± 0.255 | 6.868 ± 0.184 | ns |  | 5.492 ± 0.268 | 5.697 ± 0.226 | .025 |
| PC-Stage IV (n=26) | PC-HRG (n=11) | *p* |  | PC-Stage IV (n=26) | PC-HRG (n=11) | *p* |
| 6.672 ± 0.273 | 6.868 ± 0.184 | .036 |  | 5.478 ± 0.289 | 5.697 ± 0.226 | .032 |
| PC-Stage II (n=10) | PC-HRG (n=11) | *p* |  | PC-Stage II (n=10) | PC-HRG (n=11) | *p* |
| 6.780 ± 0.252 | 6.868 ± 0.184 | ns |  | 5.536 ± 0.269 | 5.697 ± 0.226 | ns |
| PC-Stage IV (n=26) | PC-Stage II (n=10) | *p* |  | PC-Stage IV (n=26) | PC-Stage II (n=10) | *p* |
| 6.672 ± 0.273 | 6.780 ± 0.252 | ns |  | 5.478 ± 0.289 | 5.536 ± 0.269 | ns |
| BTC-Cancers (n=14) | BTC-HRG (n=25) | *p* |  | BTC-Cancers (n=14) | BTC-HRG (n=25) | *p* |
| 6.328 ± 0.331 | 6.702 ± 0.126 | <.001 |  | 5.171 ± 0.296 | 5.413 ± 0.137 | .010 |
| BTC-Stage IV (n=11) | BTC-HRG  (n=25) | *p* |  | BTC-Stage IV (n=11) | BTC-HRG  (n=25) | *P* |
| 6.347 ± 0.348 | 6.702 ± 0.126 | .007 |  | 5.186 ± 0.247 | 5.413 ± 0.137 | .013 |
| B) Gender | | | | | | |
| Control  male (n=70) | PC/BTC/CRC  male (n=113) | *p* |  | Control  male (n=70) | PC/BTC/CRC  male (n=113) | *p* |
| 6.775 ± 0.466 | 6.682 ± 0.312 | <.001 |  | 5.488 ± 0.451 | 5.453 ± 0.277 | <.001 |
| Control  female (n=30) | PC/BTC/CRC/OVC female (n=107) | *p* |  | Control  female (n=30) | PC/BTC/CRC/OVC female (n=107) | *p* |
| 6.854 ± 0.335 | 6.648 ± 0.262 | <.001 |  | 5.518 ± 0.330 | 5.407 ± 0.248 | <.001 |
| PC/BTC  male (n=55) | Control /CRC  male (n=128) | *p* |  | PC/BTC  male (n=55) | Control /CRC  male (n=128) | *p* |
| 6.677 ± 0.291 | 6.735 ± 0.413 | .004 |  | 5.469 ± 0.277 | 5.466 ± 0.382 | ns |
| PC/BTC  female (n=35) | Control /CRC/OVC female (n=102) | *p* |  | PC/BTC  female (n=35) | Control /CRC/OVC female (n=102) | *p* |
| 6.644 ± 0.256 | 6.710 ± 0.301 | ns |  | 5.408 ± 0.274 | 5.440 ± 0.271 | ns |
| Control  male (n=70) | PC/BTC  male (n=55) | *p* |  | Control  male (n=70) | PC/BTC  male (n=55) | *p* |
| 6.775 ± 0.466 | 6.677 ± 0.291 | <.001 |  | 5.488 ± 0.451 | 5.469 ± 0.277 | .034 |
| Control  female (n=30) | PC/BTC  female (n=35) | *p* |  | Control  female (n=30) | PC/BTC  female (n=35) | *p* |
| 6.854 ± 0.335 | 6.644 ± 0.256 | <.001 |  | 5.518 ± 0.330 | 5.408 ± 0.274 | .001 |
| PC male (n=30) | BTC male (n=25) | *p* |  | PC male (n=30) | BTC male (n=25) | *p* |
| 6.775 ± 0.257 | 6.560 ± 0.291 | .004 |  | 5.579 ± 0.283 | 5.338 ± 0.207 | <.001 |
| PC female (n=21) | BTC female (n=14) |  |  | PC female (n=21) | BTC female (n=14) | *p* |
| 6.687 ± 0.235 | 6.580 ± 0.281 | ns |  | 5.476 ± 0.249 | 5.306 ± 0.288 | ns |
| PC/BTC-Cancers male (n=32) | PC/BTC-HRG  male (n=23) | *p* |  | PC/BTC-Cancers male (n=32) | PC/BTC-HRG  male (n=23) | *p* |
| 6.624 ± 0.342 | 6.751 ± 0.182 | ns |  | 5.443 ± 0.318 | 5.506 ± 0.208 | ns |
| PC/BTC-Cancers female (n=22) | PC/BTC-HRG female (n=13) | *p* |  | PC/BTC-Cancers female (n=22) | PC/BTC-HRG female (n=13) | *p* |
| 6.579 ± 0.290 | 6.755 ± 0.131 | .020 |  | 5.360 ± 0.293 | 5.488 ± 0.228 | ns |
| C) Age | | | | | | |
| Control  <59 years^b^ (n=55) | PC/BTC/CRC/OVC <59 years (n=94) | *p* |  | Control  <59 years (n=55) | PC/BTC/CRC/OVC <59 years (n=94) | *P* |
| 6.817 ± 0.435 | 6.695 ± 0.184 | <.001 |  | 5.527 ± 0.431 | 5.441 ± 0.188 | <.001 |
| Control  ≥59 years (n=45) | PC/BTC/CRC/OVC ≥59 years (n=125) | *p* |  | Control  ≥59 years (n=45) | PC/BTC/CRC/OVC ≥59 years (n=125) | *P* |
| 6.775 ± 0.430 | 6.644 ± 0.347 | <.001 |  | 5.460 ± 0.400 | 5.424 ± 0.310 | .005 |
| PC/BTC  <59 years (n=29) | Control/CRC/OVC <59 years (n=120) | *p* |  | PC/BTC  <59 years (n=29) | Control/CRC/OVC <59 years (n=120) | *P* |
| 6.679 ± 0.183 | 6.755 ± 0.328 | .010 |  | 5.419 ± 0.186 | 5.486 ± 0.324 | .018 |
| PC/BTC  ≥59 years (n=61) | Control/CRC/OVC ≥59 years (n=109) | *p* |  | PC/BTC  ≥59 years (n=61) | Control/CRC/OVC ≥59 years (n=109) | *p* |
| 6.658 ± 0.313 | 6.691 ± 0.405 | .032 |  | 5.458 ± 0.310 | 5.420 ± 0.350 | ns |
| Control  <59 years (n=55) | PC/BTC  <59 years (n=29) | *p* |  | Control  <59 years (n=55) | PC/BTC  <59 years (n=29) | *p* |
| 6.817 ± 0.435 | 6.679 ± 0.183 | <.001 |  | 5.527 ± 0.431 | 5.419 ± 0.186 | <.001 |
| Control  ≥59 years (n=45) | PC/BTC  ≥59 years (n=61) | *p* |  | Control  ≥59 years (n=45) | PC/BTC  ≥59 years (n=61) | *p* |
| 6.775 ± 0.430 | 6.658 ± 0.313 | <.001 |  | 5.460 ± 0.400 | 5.458 ± 0.310 | ns |
| PC  <63 years^b^ (n=18) | BTC  <63 years (n=22) | *p* |  | PC  <63 years (n=18) | BTC  <63 years (n=22) | *P* |
| 6.670 ± 0.250 | 6.678 ± 0.181 | ns |  | 5.438 ± 0.252 | 5.408 ± 0.188 | ns |
| PC  ≥63 years (n=33) | BTC  ≥63 years (n=17) | *p* |  | PC  ≥63 years (n=33) | BTC  ≥63 years (n=17) | *P* |
| 6.776 ± 0.245 | 6.424 ± 0.331 | <.001 |  | 5.590 ± 0.270 | 5.220 ± 0.255 | <.001 |
| PC/BTC-Cancers <63 years (n=17) | PC/BTC-HRG  <63 years (n=23) | *p* |  | PC/BTC-Cancers <63 years (n=17) | PC/BTC-HRG  <63 years (n=23) | *p* |
| 6.605 ± 0.274 | 6.726 ± 0.135 | ns |  | 5.379 ± 0.281 | 5.452 ± 0.152 | ns |
| PC/BTC-Cancers ≥63 years (n=37) | PC/BTC-HRG  ≥63 years (n=13) | *p* |  | PC/BTC-Cancers ≥63 years (n=37) | PC/BTC-HRG  ≥63 years (n=13) | *p* |
| 6.606 ± 0.342 | 6.798 ± 0.202 | ns |  | 5.422 ± 0.322 | 5.584 ± 0.278 | ns |

A group of n <10 was not considered. ns not significant, HRG high risk group, PC pancreatic cancer, BTC biliary tract cancer, CRC colorectal cancer, OVC ovarian cancer. ^a^ mean ± standard deviation, ^b^ the mean age of all subjects in the associated two groups.
