# Supplementary material for: Individualized metabolic profiling stratifies pancreatic and biliary tract cancer: a useful tool for innovative screening programs and predictive strategies in healthcare
Source: EPMA J. 2018 Aug 17;9(3):287–97. doi: 10.1007/s13167-018-0147-5 (PMC6107458; doi:10.1007/s13167-018-0147-5)
Supplement: Supplementary file 2 — (DOCX 554 kb) [file 13167_2018_147_MOESM2_ESM.docx]

| **A** |  |
| --- | --- |
| **B** |  |

**Supporting Information Fig. 1** Individual LMIs showing the highest performance for discriminating between the PC and BTC groups (**A**), and between the control/CRC/OVC groups (**B**). The mass peak areas of the LMIs were converted into logarithms. LMI, low-mass ion; PC, pancreatic cancer; BTC, biliary tract cancer; HRG, high‑risk group.

| **A** |  |
| --- | --- |
| **B** |  |

**Supporting Information Fig. 2** A mass ion of 527.3218 *m/z* at a retention time of 10.95 min showing perfect discrimination between the PC and BTC groups and the control, CRC, and OVC groups using normalized peaks (**A**). A sensitivity of 98.89% (one false-negative) and a specificity of 100% were achieved using non-normalized peaks (**B**). PC, pancreatic cancer; BTC, biliary tract cancer; CRC, colorectal cancer; OVC, ovarian cancer; HRG, high-risk group.

| **C** |  |
| --- | --- |
| **D** |  |

**Supporting Information Fig. 2** A mass ion of 749.4577 *m/z* at a retention time of 11.33 min showing perfect discrimination between the PC and BTC groups and the control, CRC and OVC groups using normalized peaks (**C**). A sensitivity of 100% and a specificity of 99.57% (one false-positive) were achieved using non-normalized peaks (**D**). PC, pancreatic cancer; BTC, biliary tract cancer; CRC, colorectal cancer; OVC, ovarian cancer; HRG, high-risk group.
